# Farming and the geography of nutrient production for human use: a transdisciplinary analysis

**DOI:** 10.1016/S2542-5196(17)30007-4

**Published:** 2017-04

**Authors:** Mario Herrero, Philip K Thornton, Brendan Power, Jessica R Bogard, Roseline Remans, Steffen Fritz, James S Gerber, Gerald Nelson, Linda See, Katharina Waha, Reg A Watson, Paul C West, Leah H Samberg, Jeannette van de Steeg, Eloise Stephenson, Mark van Wijk, Petr Havlík

**Affiliations:** a Commonwealth Scientific and Industrial Research Organisation, St Lucia, QLD, Australia; b CGIAR Research Program on Climate Change, Agriculture and Food Security, Nairobi, Kenya; c School of Public Health, University of Queensland, Herston, QLD, Australia; d Bioversity International, Heverlee, Belgium; e Faculty of Bioscience Engineering, Ghent University, Ghent, Belgium; f International Institute for Applied Systems Analysis, Laxenburg, Austria; g Institute on the Environment, University of Minnesota, Saint Paul, MN, USA; h University of Illinois, Champaign, Urbana, IL, USA; i Institute for Marine and Antarctic Studies, University of Tasmania, Taroona, TAS, Australia; j HAS University of Applied Sciences, International Food and Agribusiness, 's-Hertogenbosch, Netherlands; k Griffith School of Environment, Griffith University, Brisbane, QLD, Australia; l International Livestock Research Institute, Nairobi, Kenya

## Abstract

**Background:**

Information about the global structure of agriculture and nutrient production and its diversity is essential to improve present understanding of national food production patterns, agricultural livelihoods, and food chains, and their linkages to land use and their associated ecosystems services. Here we provide a plausible breakdown of global agricultural and nutrient production by farm size, and also study the associations between farm size, agricultural diversity, and nutrient production. This analysis is crucial to design interventions that might be appropriately targeted to promote healthy diets and ecosystems in the face of population growth, urbanisation, and climate change.

**Methods:**

We used existing spatially-explicit global datasets to estimate the production levels of 41 major crops, seven livestock, and 14 aquaculture and fish products. From overall production estimates, we estimated the production of vitamin A, vitamin B_12_, folate, iron, zinc, calcium, calories, and protein. We also estimated the relative contribution of farms of different sizes to the production of different agricultural commodities and associated nutrients, as well as how the diversity of food production based on the number of different products grown per geographic pixel and distribution of products within this pixel (Shannon diversity index [*H*]) changes with different farm sizes.

**Findings:**

Globally, small and medium farms (≤50 ha) produce 51–77% of nearly all commodities and nutrients examined here. However, important regional differences exist. Large farms (>50 ha) dominate production in North America, South America, and Australia and New Zealand. In these regions, large farms contribute between 75% and 100% of all cereal, livestock, and fruit production, and the pattern is similar for other commodity groups. By contrast, small farms (≤20 ha) produce more than 75% of most food commodities in sub-Saharan Africa, southeast Asia, south Asia, and China. In Europe, west Asia and north Africa, and central America, medium-size farms (20–50 ha) also contribute substantially to the production of most food commodities. Very small farms (≤2 ha) are important and have local significance in sub-Saharan Africa, southeast Asia, and south Asia, where they contribute to about 30% of most food commodities. The majority of vegetables (81%), roots and tubers (72%), pulses (67%), fruits (66%), fish and livestock products (60%), and cereals (56%) are produced in diverse landscapes (*H*>1·5). Similarly, the majority of global micronutrients (53–81%) and protein (57%) are also produced in more diverse agricultural landscapes (*H*>1·5). By contrast, the majority of sugar (73%) and oil crops (57%) are produced in less diverse ones (*H*≤1·5), which also account for the majority of global calorie production (56%). The diversity of agricultural and nutrient production diminishes as farm size increases. However, areas of the world with higher agricultural diversity produce more nutrients, irrespective of farm size.

**Interpretation:**

Our results show that farm size and diversity of agricultural production vary substantially across regions and are key structural determinants of food and nutrient production that need to be considered in plans to meet social, economic, and environmental targets. At the global level, both small and large farms have key roles in food and nutrition security. Efforts to maintain production diversity as farm sizes increase seem to be necessary to maintain the production of diverse nutrients and viable, multifunctional, sustainable landscapes.

**Funding:**

Commonwealth Scientific and Industrial Research Organisation, Bill & Melinda Gates Foundation, CGIAR Research Programs on Climate Change, Agriculture and Food Security and on Agriculture for Nutrition and Health funded by the CGIAR Fund Council, Daniel and Nina Carasso Foundation, European Union, International Fund for Agricultural Development, Australian Research Council, National Science Foundation, Gordon and Betty Moore Foundation, and Joint Programming Initiative on Agriculture, Food Security and Climate Change—Belmont Forum.

## Introduction

The Sustainable Development Goals (SDGs) provide a framework to monitor advances in human and ecosystems prosperity.^1^ Global food systems are central to the attainment of several of these largely interconnected goals. How food is produced and consumed is closely linked to the goals of ending poverty (SDG1), ending hunger and achieving food security and improved nutrition while promoting sustainable agriculture (SDG2), ensuring sustainable consumption and production patterns (SDG12), taking urgent action to combat climate change (SDG13), and sustainably using oceans (SDG14) and terrestrial ecosystems (SDG15).

**Research in context**

**Evidence before this study**

A substantial body of work exists on the topic of agricultural production and farm size. 570 million farmers are estimated to be responsible for the global food supply, with small farms contributing the majority of food production, especially in low-income and middle-income countries. Spatially explicit global mapping of plot sizes has supported this prevalence of small plots in many low-income and middle-income countries. Some of these analyses have been extended through estimation of the average size of agricultural areas (a proxy for average farm size) using spatial and statistical methods, yielding information on the contribution of different average agricultural areas to crop production, which varied significantly depending on crop type. This analysis, however, did not account for the distribution of different farm sizes across the same areas, nor for production of livestock and fish. Several studies have shown links between agriculture and dietary diversity, and diversity of national food supplies has been reported to have become more homogeneous over time, raising concerns about the evolution of global nutritional diversity, which is associated with many measures of human and ecosystems wellbeing, including child malnutrition. The structure of global food production, and diversity of food supply are key to debates on how food should be produced now and in the future, and are fundamental for the design of feasible responses to attaining global human and planetary health.

**Added value of this study**

Previously, spatial linking of the global structure of food production to its functional diversity and the provision of key nutrients for anthropogenic use has not been fully quantified. Since the land connects human beings to both food production and the environment, this information is essential for designing more sustainable food systems and for the attainment of many of the sustainable development goals. Our results show that both production and nutrient diversity diminish with increasing farm size and that, irrespective of farm size, more diverse areas produce more nutrients. Our study also incorporates the latest spatial and statistical data on crops, livestock, and fish products, which have seldom been included simultaneously in these types of analyses.

**Implications of all the available evidence**

Our results show that farm size and nutritional functional diversity are key factors for global nutrient production. This finding has crucial implications for food and nutritional security. The evidence also shows that both small and large farms have crucial importance on a global basis. Small farms are still essential to the provision of food and nutrients in low-income and middle-income countries, whereas surpluses from larger farms ensure the necessary trade balances to deal with scarcity in some parts of the world. Furthermore, agricultural diversity needs to be safeguarded when agricultural intensification practices are promoted, given that, historically, intensification has decreased the number of crops planted, especially as farm sizes increase. Management of the risks associated with agricultural diversity losses will be essential in efforts to attain the Sustainable Development Goals. The information presented will be useful in attempts to improve the sustainability of food production, especially in countries in which the dynamics of global change processes are causing profound changes to livelihoods, economies, and ecosystems.

Agriculture, livestock, and fisheries provide the basis of production for edible nutrients used by mankind, whether directly through food manufacturing and consumption, or indirectly to feed animals and fish or for energy or fibre production. These sectors are part of the global food systems and are responsible for maintaining millions of livelihoods, from farmers, retailers, farm advisers, and scientists, all the way to the consumers. Their importance in regulating environmental services mainly through land and water use, nutrient cycles, and climate regulation is also undeniable.^2^

The scale of the food production challenge is clear: some studies^3^ suggest that a 70% increase in food availability by the 2050s will be essential to keep up with the demand for food from an increasingly numerous and affluent population. Put another way, more food will need to be produced on the planet in the next 50 years than has been produced in the past 400 years,^4^ with the additional constraint of ensuring that key environmental planetary boundaries are not exceeded in the process.^2^

This increase in food availability alone will not guarantee human wellbeing. Additionally, food systems must also provide foods of high nutritional quality and diversity to support the needs for human health and nutrition,^5^ while other crucial challenges such as poverty reduction, equity, land tenure, education and health accessibility, and reductions in emissions are resolved simultaneously.

Diversity in the food species that contribute to a diet is associated with improved nutrient adequacy and food security.^6^, ^7^ However, the global diversity of national food supplies has been decreasing since 1960, with a steady increase in the importance of major cereals and oil crops^8^ relative to other commodities like fruits or vegetables. Agricultural systems change through time in response to a wide range of drivers, particularly intensification processes (ie, increasing production per unit of land, labour, or capital), which can often lead to specialisation of production in the pursuit of economic efficiencies.^9^ As efforts are made to increase food production, achieving a balance between intensification and diversity of production has become increasingly important from a nutritional perspective. Identification of the policy options and technological changes that can achieve this balance will depend on a more complete understanding of the geography of current food production, and how this might evolve as agricultural systems change in response to drivers of change such as population growth, urbanisation, and climate change.

Whether food is produced on small or large farms, with minimal or large amounts of external inputs, or whether crops are grown singly or in combination with other crops and livestock or fish, all forms of food production have associated societal, economic, and environmental costs and benefits, which spread from the farmer all the way to the consumer. Different methods of production will have different abilities to handle challenges such as dealing with climatic and economic risk, adapting and mitigating climate change, generating employment and livelihood options, and maintaining ecosystem services.

Our study is a small first step towards building consistent, global data for the study of these key issues. We aimed to estimate the relative contribution of farms of different sizes to the production of various agricultural commodities and associated nutrients, as well as analysing how the diversity of food and nutrient production changes with farm size. We also present high-resolution global maps of the production of several key nutrients, with underlying information on the crop or animal producing systems.

## Methods

### Study design

We estimated the relative contribution of farms of different sizes to the production of different agricultural commodities and nutrients and the associations between diversity of production and size of farm. We used existing, spatially explicit global datasets of location and production of major crops, livestock, and aquacultural products and estimated the production of essential nutrients. We allocated national food and nutrient production data for these commodities to farms of different sizes using a global dataset of field size coupled with non-spatial methods, and we calculated diversity metrics for vegetables, cereals, livestock, fish, sugar crops, pulses, roots and tubers, oil crops, and fibre crops.

We focused on estimating the production of dietary energy (calories) of seven essential nutrients: vitamin A, vitamin B_12_, folate, iron, zinc, calcium, and protein. This selection reflects nutrients of public health interest because of either existing widespread deficiencies (vitamin A, iron, and zinc) or because intakes are commonly low particularly in developing countries (vitamin B_12_, folate, and calcium). We also included calories and protein as essential macronutrients.

More detailed descriptions of the methods are available in the appendix. Our analysis included 161 countries (we excluded several small island states); the country list and allocation to regions are shown in the appendix.

### Data sources

We extracted production data for 41 crops in 2005 from the dataset of Ray and colleagues,^10^ which was based on the work of Monfreda and colleagues.^11^ For seven livestock products in 2005 we used data from Herrero and colleagues.^12^ For the 14 fish functional groups, we used data from Watson and colleagues.^13^ We used the fish data for the computation of the nutrient yield and diversity metrics only, as they could not be allocated to farm sizes. We sourced data on the nutrient compositions of the 62 commodities from the US Department of Agriculture (USDA) online database and adjusted for edible portions. To estimate farm size distributions, we used the data of Lowder and colleagues,^14^, ^15^ supplemented where needed with additional data for missing countries (appendix).

### Statistical analysis

To allocate agricultural production data to different farm sizes at a country level, we used a spatially explicit global dataset on field-size distribution.^16^ For each country, we calculated the relative proportion of four different field sizes: “very small” (≤0·5 ha), “small” (>0·5–2 ha), “medium” (>2–100 ha), and “large” (>100 ha) and imputed a plausible field-size distribution to the country's farm-size distribution in such a way that the national (non-spatial) farm-size areas matched the national (spatial) field-size areas. We used the resulting matrix of relative proportions to allocate all the fields of a certain size to farms of different sizes. We then allocated production to all farm sizes in relation to the ratio of relative production to relative area, first weighting field size by suitability class, using length of growing period as a proxy for general agricultural suitability. This allowed us to allocate production to a country's distribution of farm sizes, taking account of agricultural suitability within the country.

We calculated and mapped nutritional yield^17^ for all crops, livestock, and fish combined, expressed as the number of people whose annual recommended daily allowance (RDA) for different nutrients could be met from crop, livestock, and fish production per grid cell.^18^

We calculated three diversity metrics based on all of the crop, livestock, and fish products used in the analysis:^19^ the Shannon diversity index, *H*, which represents how many different types of foods are produced in a pixel and how evenly these different types are distributed; the species richness, *S*, a simple count of the number of commodities produced in each pixel; and the Modified Functional Attribute Diversity index (MFAD), the sum of pairwise distances between functional units; this index reflects the diversity in nutrient composition of foods produced in each pixel. Maps of *S* and MFAD are available in the appendix.

We did all analyses using the R open source statistical package (version 3.3.2).

### Role of the funding source

The funder of the study had no role in study design, data collection, data analysis, data interpretation, or writing of the report. The corresponding author had full access to all the data in the study and had final responsibility for the decision to submit for publication.

## Results

**Figure 1:**
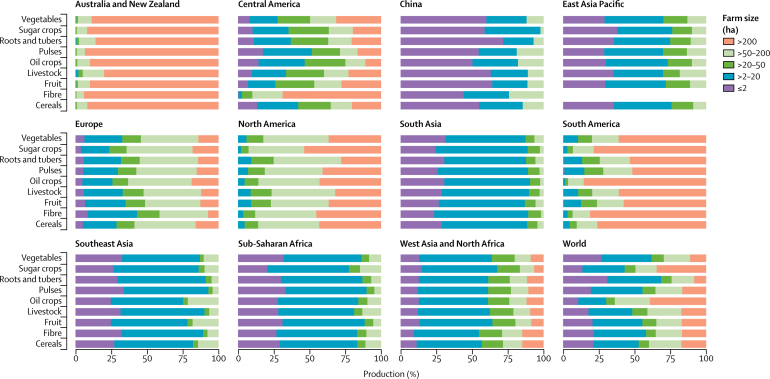
Production of key food groups by farm size

Our analyses show that globally, farms smaller than 50 ha produce between 51% and 77% of the volume of the major food groups for human consumption: cereals, fruits, pulses, roots and tubers, and vegetables (figure 1). Exceptions are sugar and oil crops, which tend to be produced on large farms (>50 ha) as large plantation crops, and livestock, of which 48% of global production is on small (≤20 ha) and medium (>20–50 ha) farms.

**Figure 2:**
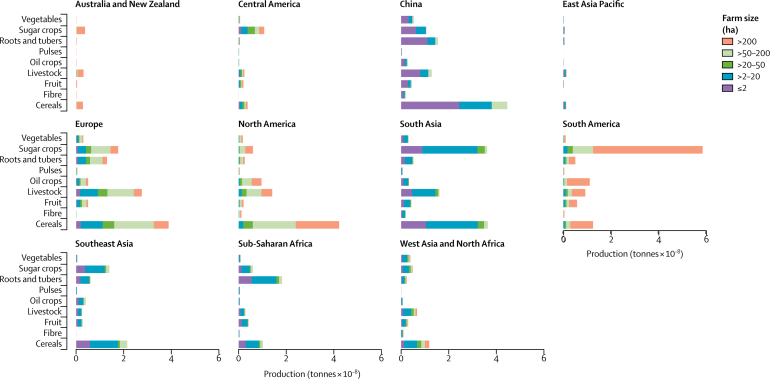
Distribution of production of key food commodity groups by farm size

Although these global numbers are important, they mask substantial regional differences in what food is produced and how it is produced (figure 1 and figure 2). Large farms (>50 ha) dominate production in North America, South America, and Australia and New Zealand. For example, in these regions large farms contribute approximately 75–100% of all cereal, livestock, and fruit production, and the pattern is similar for other commodity groups (appendix). By contrast, small farms (≤20 ha) produce more than 75% of most food commodities in sub-Saharan Africa, southeast Asia, south Asia, and China. A clear example of these structural differences is the production of cereals in Europe and North America compared with South Asia and China, where similar volumes of cereals are produced, but with very different production structures (large *vs* small farms; figure 2). Europe, west Asia and north Africa, and central America are different from other regions in that medium size farms (>20–50 ha) also contribute substantially to the production of most food commodities.

Very small farms (≤2 ha) are important and have local significance in sub-Saharan Africa, southeast Asia, and south Asia, where they contribute around 30% of most food commodities and where they are managed by millions of smallholder farmers. In China, such farms produce more than 50% of all food commodities (except for fibre crops), in particular fruits (64%), vegetables (60%), sugar crops (59%), roots and tubers (72%), and livestock (63%).

**Figure 3:**
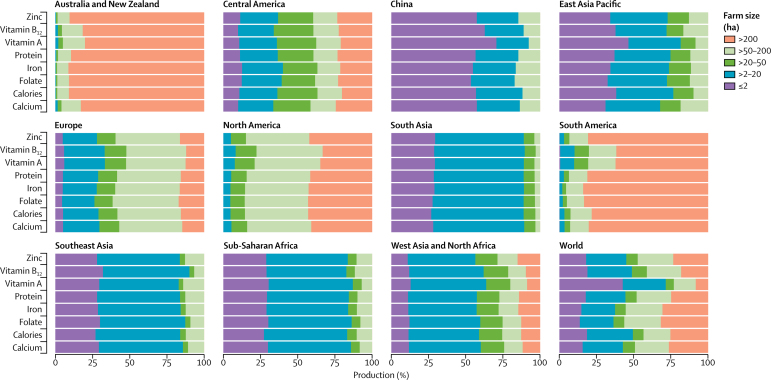
Distribution of nutrient production by farm size

The global patterns of nutrient production by farm size are similar to those of the production of food commodities (figure 3). With the exception of iron and folate, small (≤20 ha) and medium (>20–50 ha) farms supply 51–77% of the essential nutrients studied here. Notably, small farms (≤20 ha) provide 71% of global vitamin A production; vitamin A is supplied mainly from fruits and vegetables, some livestock, and orange-fleshed roots and tubers, which are produced mostly on these small farms.

A regional analysis (figure 3) shows that both small and large farms are vital to local nutrient production in each of the regions studied. Small farms (≤20 ha) produce most of the essential nutrients (>80%) in sub-Saharan Africa, southeast Asia, south Asia, China, and the rest of east Asia Pacific. Farms smaller than 2 ha produce more than 50% of all nutrients in China and are of key importance in south Asia, southeast Asia, sub-Saharan Africa, and east Asia Pacific, where they produce more than 25% of the nutrients. Farms larger than 50 ha contribute most of the nutrient production in Europe, North America, and South America, and Australia and New Zealand. In South America and Australia and New Zealand, very large farms (>200 ha) produce more than 50% of the nutrients.

**Figure 4:**
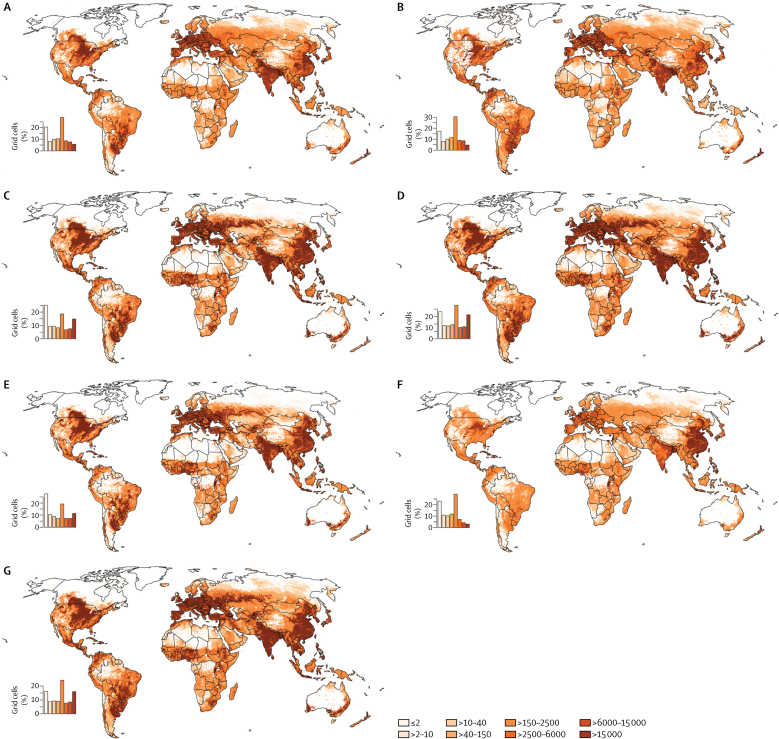
Global hotspots of nutritional yield Nutritional yield was calculated from 41 crops, seven livestock products, and 14 fish groups for (A) calcium, (B) folate, (C) iron, (D) protein, (E) vitamin A, (F) vitamin B_12_ and (G) zinc. The maps represent the number of people whose recommended daily allowance for each nutrient could be met, per grid cell. Maps for individual commodities are available in the appendix.

Areas of substantial nutrient production can be identified around the world. Figure 4 shows nutritional yields—ie, the number of people whose annual recommended allowances for each nutrient could be met from the aggregated nutrient production from crops, livestock, and fish combined per unit of land (grid cell). Although there are some differences for specific nutrients, the general overall patterns in the maps are similar, with parts of China, India, Europe, the North American Great Plains, southern Brazil and northern Argentina, east African highlands, and parts of west Africa being noticeable production areas. The lowest productivity is for vitamin A and vitamin B_12_, which are supplied in large quantities by fewer commodities (ie, roots and tubers for vitamin A and livestock and fish products for vitamin B_12_).

**Figure 5:**
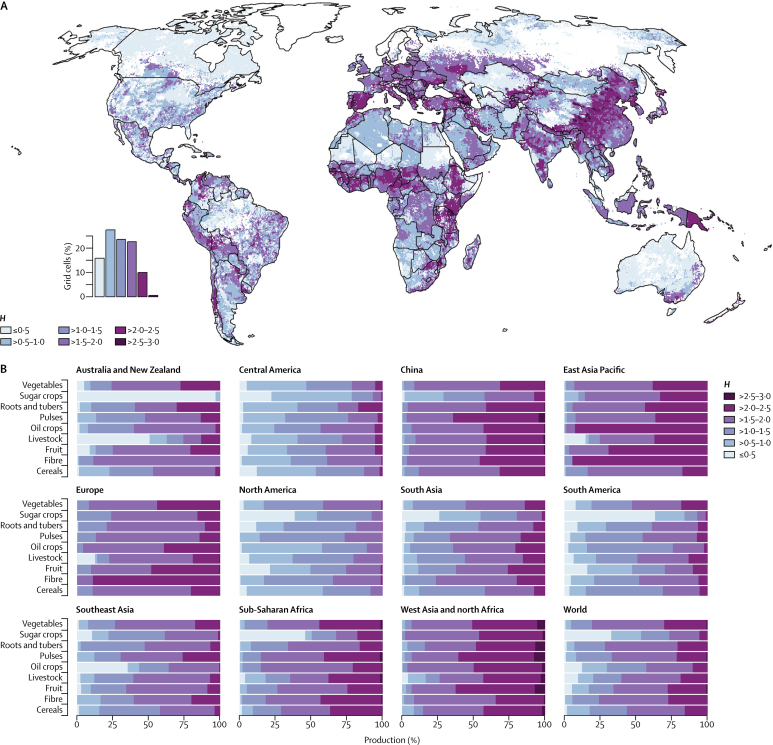
Diversity of global food production (A) Map of global diversity of food commodities. (B) Commodity group production by diversity category. Diversity is represented by the Shannon diversity index, *H*, which represents how many different types of foods are produced in a pixel and how evenly these different types are distributed. The higher the Shannon index, the higher the diversity.

Mapping agricultural diversity at grid level allows several trends to be identified (figure 5A). First, differences in diversity between regions are substantial, with higher diversity (*H*>1·5) in large parts of Europe, Africa, Asia, and the western part of South America, and lower diversity in large parts of Australia, North America, and South America. Second, overlaying the diversity data with the food and nutrient production data shows that, on a global level, farm areas with higher diversity (*H*>1·5) produce most of the vegetables (81%), fibre crops (76%), roots and tubers (72%), pulses (67%), fruits (66%), livestock (60%), and cereals (56%), although they occupy a smaller percentage of the grid cells than do the less diverse areas (figure 5). The exceptions are sugar crops (27%) and oil crops (43%), which are often grown in single crop plantations.

**Figure 6:**
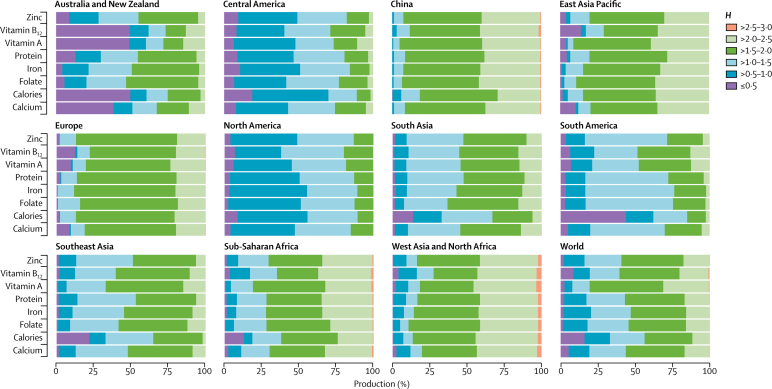
Production of nutrients by the diversity category Diversity is represented by the Shannon diversity index, *H*, which represents how many different types of foods are produced in a pixel and how evenly these different types are distributed. The higher the Shannon index, the higher the diversity.

Third, combining the diversity measures with spatially explicit plot sizes, which are highly correlated with farm size, shows that agricultural diversity (*H*) decreases as plot size increases (p<0·0001; appendix). In particular, areas with small and medium farms (≤50 ha) have larger diversity than do larger scale farms. These differences also translate into differences in nutrient production (figure 6). On a global level, areas with higher diversity of food commodities (higher *H*) produce more micronutrients than do areas with less diversity. This effect is particularly noticeable in places such as China, sub-Saharan Africa, east Asia Pacific, and west Asia and north Africa. In contrast with North America, in Europe, although production comes mostly from medium and large farms, it is not farm size, but the diversity of production that drives nutrient production in this region.

## Discussion

Our results show that the geography, structure, and diversity of farming matters significantly in the production of key nutrients for anthropogenic use. The production of global food commodities differs geographically and is governed by agroclimatic conditions, soil types, population density, and distance to markets. These factors, together with the competitiveness of the agricultural sector and alternative sources of employment, largely determine the structure of farming in the world. We show that both large and small farms have crucial roles in food and nutrient production and that this role largely depends on the region. Small farms are not only responsible for supporting millions of smallholders in low-income and middle-income countries, but also produce the majority of a very diverse set of commodities for human consumption, especially for poor people.^20^ By contrast, large farms can be less diverse, but their sheer sizes and productivity of fewer, easier to grow, high-yielding crops, ensure that there are tradeable surpluses of nutrients available to the parts of the world that need them most.^21^ This situation represents a marriage of convenience for global nutrient supply and for mankind's wellbeing. However, their environmental consequences remain to be more comprehensively studied than they have been to date.

To achieve nutrient adequacy, food diversity is an essential aspect of diet quality,^22^ and diversity in agricultural production systems can stimulate long-term productivity, stability, ecosystem services to and from agricultural lands, and resilience to shocks (eg, pests and diseases, climate, or price shocks).^23^ Our findings on diversity suggest that as farm sizes increase, a shift occurs in the type and intensity of crops grown. Species that are more suitable to be grown in smaller plots (eg, vegetables, fruits, and some roots and tubers) are reduced, whereas species that can be easily cultivated with mechanised techniques, such as cereals and sugar and oil crops, are maintained. By contrast, smaller plots also contain a broader mixture of crops and livestock.

The historical intensification of agriculture has yielded more but less diverse food and a reduction in the sources of key essential nutrients.^8^ Our data suggest that although most commodity groups are present across all farm sizes, there is a risk that numbers of species cultivated, particularly highly nutritious food groups, will decrease as farm sizes increase. Reversing of this trend is essential to safeguard the adaptive capacity of agriculture to maintain the supply of essential nutrients for human health. In low-income countries, the production of diverse commodities contributes to consumption diversity because trade is limited and most production is consumed locally.^19^ Production diversity is therefore part of a coping strategy that needs to be maintained. In high-income and middle-income countries, diversity of food can be obtained more easily from markets supplied by national or by international trade than in low-income countries, so production and supply diversity are not coupled. Incentives might be needed to manage diversity in such settings for risk management and long-term economic, health, and environmental benefits.^24^, ^25^

From a socioeconomic perspective, a shift in the typical development of small farms needs to occur to ensure that agricultural intensification in low-income and middle-income countries, which is usually promoted through the use of a few cereals and legumes, does not lead to reductions in agrobiodiversity. The number of species promoted needs to increase and investments and policy incentives to diversify agriculture to promote healthier diets and gender-sensitive agriculture needs to be pursued. This need has already been acknowledged in some parts of the world and successful examples of the promotion of diversified smallholder agriculture exist.^26^, ^27^, ^28^, ^29^ Similarly, nutritional quality must become a more prominent driving force in agriculture and food policy development and incentives such as price premiums or low-interest credits, certification, or guaranteed markets to promote the production of nutrient-rich foods including vegetables, fruits, perennial crops, livestock and fish species will need to be developed.

Our analysis focused on the production of a range of commodities for human use, and, as such, represents only one of the building blocks contributing to how nutrients are used. The food industry plays an essential part in how nutrients are transformed, packaged, and accessed by consumers. The industry is also pivotal in establishing production patterns in certain regions by the creation and promotion of markets for commodities of interest through large agribusiness companies. Policies, regulation, and effective public-private partnerships are and will be needed to ensure improved harmonisation of goals among the actors of the food chain to achieve human and ecosystems health.

Our study opens up new research opportunities to improve attempts to attain the SDG goals. Understanding of the structure of food and nutrient production in the world can help the targeting and prioritisation of research and investment actions to support the attainment of sustainable and equitable agricultural development together with healthier diets and healthier ecosystems. Our data provide the basis for the analysis of the effects of climate change on global and regional nutrient supply, or for projecting and incorporating scenarios of the consequences of farm size consolidation on food and nutrient supply in the future and the associated social and environmental costs, and for the investigation of nutrient yield gaps. Essential to advancement in this subject would be to link the results of our study to nutrient consumption data from disaggregated human population distributions, as well as to increase the number of nutrients included in the analysis (eg, adding essential fatty acids). Such work would enable the computation of specific dietary patterns and nutrient supply solutions to contribute to the SDGs.

Despite the importance of our findings, our study has also shown many inadequacies and data gaps that could guide further research of this topic. For example, we could not allocate aquacultural production to farm sizes, as a large proportion of aquaculture occurs in deltas or close to water bodies, which are difficult to allocate to terrestrial land use systems. Efforts to better map these systems are crucial. Advances have been made in the mapping of agricultural areas,^11^ plot size distributions,^16^ and the predominance of certain farm sizes.^30^ However, the development of high-resolution, global, continuous representations of farm size distributions remain elusive. Our analyses covered more than 85% of the global cropped area. However, we need to increase the number of mapped commodities, especially nutrient-rich foods that occupy small areas and contribute to dietary quality, particularly for women and children. Advances in crowdsourcing, remote sensing,^31^ and farm data collection will help to circumvent these problems^32^ in the future.
