# Supplementary material for: Farming and the geography of nutrient production for human use: a transdisciplinary analysis
Source: Lancet Planet Health. 2017 Apr;1(1):e33–42. doi: 10.1016/S2542-5196(17)30007-4 (PMC5483486; doi:10.1016/S2542-5196(17)30007-4)
Supplement: Supplementary appendix [file mmc1.pdf]

THE LANCET
Planetary Health

Supplementary appendix

This appendix formed part of the original submission and has been peer reviewed. We post it as supplied by the authors.

Supplement to: Herrero M, Thornton PK, Power B, et al. Farming and the geography of nutrient production for human use: a transdisciplinary analysis. *Lancet Planetary Health* 2017; **1:** e33–42.

Farming and the geography of nutrient production for human use: a transdisciplinary analysis

Supplementary material

Mario Herrero[1*], Philip K Thornton[1,2], Brendan Power[1], Jessica Bogard[1,3], Roseline Remans[4,5], Steffen Fritz[6], James N Gerber[7], Gerald Nelson[8], Linda See[6], Katharina Waha[1], Reg A. Watson[9], Paul C West[7], Leah H Samberg[7], Jeannette van de Steeg[10], Eloise Stephenson[1,11], Mark van Wijk[12], and Petr Havlík[6].

[1]Commonwealth Scientific and Industrial Research Organisation (CSIRO), 306 Carmody Road, St Lucia, QLD 4067, Australia.
[2]CGIAR Research Programme on Climate Change, Agriculture and Food Security (CCAFS), ILRI, Nairobi, Kenya.
[3]School of Public Health, University of Queensland, Herston Road, Herston, QLD 4006, Australia.
[4] Bioversity International, W. De Croylaan 42, 3001 Heverlee, Belgium.
[5] Faculty of Bioscience Engineering, Ghent University, Coupure Links 653, B-9000, Ghent, Belgium.
[6]International Institute for Applied Systems Analysis, Laxenburg, Austria.
[7]Institute on the Environment, University of Minnesota, 1954 Buford Avenue LES 325, Saint Paul, Minnesota 55108, US.
[8] University of Illinois, Champaign, Urbana, USA.
[9]Institute for Marine and Antarctic Studies, University of Tasmania, 15-21 Nubeena Crescent, Taroona, Tasmania 7053, Australia.
[10]HAS University of Applied Sciences, International Food and Agr-business, Onderwijsboulevard 221, 5223 DE ‘s-Hertogenbosch,The Netherlands.
[11]Griffith School of Environment, Griffith University, Nathan, Brisbane, QLD, 4111, Australia.
[12]International Livestock Research Institute, PO Box 30709, Nairobi, Kenya.

*Correspondence: Dr Mario Herrero, Commonwealth Scientific and Industrial Research Organisation (CSIRO), 306 Carmody Road, St Lucia, QLD 4067, Australia
Mario.Herrero@csiro.au; +61 7 3214 2538.

1. Detailed Methods

Global field size dataset: The field size data were collected through a crowdsourcing campaign using Geo-Wiki (http://www.geo-wiki.org), which is an application that involves the crowd in visual interpretation of very high resolution satellite imagery. During one campaign that was focused on human impact (See et al., 2015), volunteers were asked to interpret field sizes located in cropland areas in a 1 km^2 sample area using the following 4 categories: 1 (very small); 2 (small); 3 (medium) and 4 (large). The data gathered from this campaign consisted of 53,000 samples of 1 km^2 areas, of which around 36,000 were at unique locations. Of these unique locations, 13,963 samples contained cropland and therefore the size of the field. This sample data set of ordinal values was then interpolated using inverse weighted distance, treating the ordinal values as a continuum, to create a global map of field size at a 1 km^2 resolution, which was then clipped using the IIASA/IFPRI cropland map (Fritz et al., 2015). The global field size map can be downloaded from http://www.geo-wiki.org.

The field sizes have been roughly correlated to areas as follows: a field size of very small corresponds to fields that are smaller than 0.5 ha; small fields correspond to an area of 0.5 to 2 ha; medium fields to between 2 and 100 ha; and large fields are all fields larger than 100 ha. These field sizes were derived by measuring the size of fields found inside the 1 km^2 areas and by adjusting, in particular, the large field size based on comparisons of large fields in representative areas and using expert judgment from members of the GEOGLAM initiative (http://www.earthobservations.org/geoglam.php).

The field size map was validated in two ways. The first type of validation was to assess the reliability of the crowd. A random sample of the field size data collected during the campaign was provided to multiple experts and then correlated with the data obtained from the crowd. This resulted in a Spearman's rank correlation coefficient of 0.80, indicating an acceptable agreement. The second form of validation was to consider the effect of interpolation by randomly choosing a new set of samples from the interpolated map, comparing these to the field sizes determined by multiple experts from visual interpretation of very high resolution imagery. In total, 181 validation points with field sizes ranging from very small (1) to large (4) were used. The results indicate a Spearman's rank correlation coefficient of 0.78. Given that the global map was developed using only 13,963 points, this agreement is considered to be satisfactory. More details of the validation process can be found in Fritz et al. (2015).

Farm size distributions: Currently there are no farm-size distribution data for the globe. Lowder et al. (2014, 2016) compiled much information on the topic, drawing from census data. Two issues associated with these data are that the country coverage is incomplete, and that the information is based on many different years of census data (and some of the data are from the 1960s). The approach taken here was to calculate regional average farm size distributions for the regions of interest, update the number of farms to more recent values, where possible, and then use these regional distributions on a country-by-country basis to derive farm sizes on a national basis, on the assumption that the structure of agriculture in different countries of a region exhibits some essential similarities. In this way, a farm-size distribution can be estimated for any country, provided that data exist for the total number of farms in the country. The more recent the information on number of farms in the country, the better, given that farm size distribution in many countries may be changing over time for a variety of reasons. Out of the 163 countries in our analysis, data on number of farms for 138 countries were available in Lowder et al. (2014), and estimates for the remaining 25 were found from online sources, either from census data or, as a last resort, estimated from rural population and household size data. For the 167 countries that Lowder et al. (2014) found data for, the total number of farms was 569.6 million; this included several small island states excluded from our analysis. For our study's 163 countries, we calculated a total of 577.2 million farms. The countries in this analysis and their regional allocation are shown in Table S2.

To allocate national production statistics to the different farm sizes at a country level, we used the spatially explicit global dataset on field-size distribution described earlier (Fritz et al., 2015). This dataset is based on a 1-km resolution cropland percentage map for the baseline year 2005 overlaid with field size data collected via a crowdsourcing campaign. For each country, we calculated the relative proportion of four different field sizes, "very small" (<0.5 ha), "small" (0.5-2 ha), "medium" (2-100 ha) and "large" (> 100 ha). We then imputed a plausible field size distribution to the country's farm size distribution (using either national or regional data), using three simple rules, so that the national (non-spatial) farm size areas matched the national (spatial) field size areas:

- The distributions should be monotonically increasing, monotonically decreasing, or unimodal; i.e., smooth distributions rather than simply random selections.
- Any allocation reflects the assumption that the proportion of field sizes from 0.5-2 ha decreases as farm size increases.
- Any allocation reflects the assumption that the proportion of field sizes great than 100 ha increases as farm size increases from 100 ha upwards.

From the tables relating field sizes to farm sizes for different countries and regions, we then developed matrices of relative proportions which were used to allocate all the fields of a certain size to farms of different size. Spatially explicit national agricultural production data were then filtered through this field-size / farm-size matrix, resulting in production data being allocated to different farm sizes. We did this allocation on the basis of production rather than area, given that there are substantial differences between different agricultural production data sets in relation to actual areas cropped. To take some account of the differences in agro-ecological suitability within a country, we utilised a length of growing period dataset (Jones and Thornton 2009, 2013) to define the following five categories: LGP<60 days, 60-120 days, 120-180 days, 180-240 days, and >240 days per year. For each commodity, we calculated relative production per pixel. Each country's total production of a crop was then allocated by plot size, by farm size and by LGP zone, in relation to the ratio of relative production to relative area. This final step was done to take some account of areas with more or less suitability for the production of each commodity, as reflected in the production data; this results in a weighting of field sizes according to their relative production as spread across the country's farm sizes. This is an important element, because the methods we used do not allow us to say where farms of particular size are actually located; and a small farm in a low rainfall regime will not be as productive as a small farm in a higher rainfall regime, all other things being equal. Our estimates of farm-size distributions in countries where such data do not exist are plausible, but they would benefit from triangulation from other sources. A fully spatially-explicit farm size distribution dataset for the globe is yet to be developed.

An example of the calculation procedure is shown in Table 1. Relative production by plot size and by LGP zone is shown in Table 1A; this shows the proportion of national production of a crop broken down by plot size and by LGP zone. In this example, there are no pixels in LGP zones 4 or 5 and no pixels with a plot size > 100 ha. The proportion of national crop area is calculated on the same basis (results not shown in Table 1). Table 1B shows the allocation of plot sizes to farm sizes. Rows sum to unity, and the grey cells must be zero by definition. In the example, very small plot sizes are allocated to farm sizes as shown; thus 80 percent of the very small plots (<0.5 ha) are allocated to farm sizes < 1 ha, as are 20 percent of the small plots (0.5-2 ha). The distributions by plot size (down the column) follow the three rules given above. The allocation was done by trial-and-error so that the relative total area of each plot size category, as calculated across all farm sizes, matches the relative area of each plot size category calculated from the dataset of Fritz et al. (2015) for the country under consideration (data not shown in Table 1). The third step in the procedure is shown in Table 1C, where the relative production RP of the crop in plot size *i* and farm size *j* is calculated as:

$$RP_{i,j} = \left(\sum_{k=1}^{5} PROD_{i,k} / AREA_{i,k} \right) * FA_{i,k}$$

where *k* is the LGP zone, PROD is the proportion of national production (Table 1A), AREA is the proportion of national area, and FA is the imputed plot size proportion (Table 1B). These values of RP are then summed for each farm size and normalised to match total national production of the crop (columns 6 and 7 in Table 1C).

Table 1.
A. Relative production by plot size (Fritz et al., 2015) and LGP zone (Jones and Thornton, 2013).

**Relative Production**	**LGP 1 (<60 days)**	**LGP 2 (60-120)**	**LGP 3 (120-180)**	**LGP 4 (180-240)**
Plot size 1 (<0.5 ha)	0.210	0.050	0.000	0.000
Plot size 2 (0.5-2 ha)	0.080	0.230	0.280	0.000
Plot size 3 (2-100 ha)	0.000	0.150	0.000	0.000
Plot size 4 (>100 ha)	0.000	0.000	0.000	0.000

B. Imputed allocation of plot sizes to farm sizes (see text). Grey cells must be zero by definition.

Plot size by farm size matrix	Plot size 1 (<0.5 ha)	Plot size 2 (0.5-2 ha)	Plot size 3 (2-100 ha)	Plot size 4 (>100 ha)
Farm size < 1 ha	0.80	0.20	0.00	0.00
Farm size 1-2 ha	0.55	0.45	0.00	0.00
Farm size 2-5 ha	0.20	0.50	0.30	0.00

C. Relative production (from A above) allocated to the plot size by farm size matrix (B above).

Plot size by farm size matrix	Plot size 1 (<0.5 ha)	Plot size 2 (0.5-2 ha)	Plot size 3 (2-100 ha)	Plot size 4 (>100 ha)	Production by farm size (sum)	Normalised production by farm size
Farm size < 1 ha	0.883	0.804	0.000	0.000	1.687	0.245
Farm size 1-2 ha	0.607	1.809	0.000	0.000	2.416	0.351
Farm size 2-5 ha	0.221	2.010	0.540	0.000	2.771	0.403
**Total**					**6.874**	**1.000**

Crop production data: The majority of the EarthStat crop production data used here was developed for year 2000 (Monfreda et al, 2008), with updates for major crops (wheat, rice, maize, soy) to year 2005 (Ray et al, 2012). The yield and harvested area data for the year 2000 were derived as an average of crop census data from 1997-2003. A range of years was used because census data are not reported every year. In addition, the average reduces anomalies from short-term weather and market shocks. Census data were used from 206 countries, 150 of which had sub-national data at the state/province level, 73 of those 150 countries had data at the county/district level.

Monfreda et al. 2008 then combined these crop census data with a cropland area data layer (Ramankutty et al. 2008) to distribute yields and harvested area data within political units. The Ramankutty et al. 2008 cropland area was developed by combining agricultural census data with two satellite-derived land cover products (Friedl et al. 2002, Bartholome et al. 2005). Monfreda et al. 2008 distributed crop-specific harvested area data within political units proportional to total cropland area; yield data were distributed uniformly within political units.

More detailed methods for developing the crop production data are in Monfreda et al. 2008; additional details for the cropland area data are in Ramankutty et al. 2008. Details on the updates to the yield and harvested area data for wheat, rice, maize are available in the supplementary materials of Ray et al. (2012). The data are available at: www.EarthStat.org

Global livestock production: We updated the global livestock productivity dataset of Herrero et al. (2013) for 2000 to the base year 2005. We made all the calibrations by country rather than by region, hence adding significant additional detail to our estimates. Detailed information of the methods employed for constructing these datasets is presented in Herrero et al (2013). What follows is a summary of the methods.
A livestock systems classification based on an earlier effort by Sere and Steinfeld (1996) and recently updated by Robinson et al (2011) was used as the starting point. We differentiated 8 different types of livestock systems in 28 geographical regions of the world for this study. These are three types of each grazing and mixed systems, differentiated by agroecology: arid, humid and temperate/highland, and peri-urban and other systems. Details can be found in Robinson et al. (2011).

Numbers of animals for each of these systems and regions were estimated using the data of Robinson et al (2014) for the year 2005. For ruminants (cattle, sheep and goats), we disaggregated the dairy and beef cattle herds using livestock demographic data for total cattle, sheep and goats and the dairy females for each species, respectively, from FAOSTAT. We used herd dynamics models parameterised

for each region and production system using reproduction and mortality rates obtained from extensive literature reviews to estimate herd composition. We then subtracted the number of total dairy animals from the total number for each species. This procedure enabled us to have distinct herds for the production of milk and beef.

For monogastrics (pigs and poultry), we only differentiated two systems: smallholder and industrial production systems, since these are the most important ones and industrial systems exhibit most of the growth in meat production globally (Bruinsma 2003) The allocation of poultry, eggs and pork production was done on the basis of knowledge of the total product output from these two systems from national information from selected countries in the different regions, applied to the respective region. The numbers of animals contributing to the estimated animal production was also computed using herd dynamics models coupled with information on mortalities, reproduction and productivity for these two main systems for each region.

The calculations of biomass consumption and productivity estimations from different species in each region and system followed several stages. We first developed feasible diets for each species in each region and production system. The proportions of each feed in the diet of each species was obtained from extensive information available in the literature and from databases and feeding practice surveys at key research centres in the world (i.e. FAO, ILRI, NDDB). Data on feed quality was obtained from the databases containing regional feed composition data for each feed. We then estimated productivity. For ruminants, the information on the quantity and quality of the different feeds was then used to parameterise an IPCC tier 3 digestion and metabolism model (RUMINANT, Herrero et al 2002), as described in Herrero et al (2013) The model estimated productivity (milk, meat), methane emissions and manure and N excretion. For monogastrics, information on feed quality was used to estimate feed intake, productivity and feed use efficiency using standard nutrient requirements guidelines (NRC 2008).

All information on animal production (bovine milk, bovine meat, sheep and goat milk, sheep and goat meat, pork, poultry and eggs) and for grains as feed was harmonised with FAOSTAT's commodity balance sheets at national level following an iterative procedure restricted to deviate +/- 20% from the statistical data in FAOSTAT. More information of this process can be obtained in the supplementary information and in Havlik et al. (2013).

Fish production data and maps: Landings of marine and freshwater fishes, both wild caught and aquaculture production was mapped to 30-min spatial cells. Marine capture fisheries data was sourced from http://dx.doi.org/10.4226/77/58293083b0515 and this represents a harmonized and mapped compilation of global catch from 1950 to 2014 sourced in turn from the United Nations Food and Agriculture Organization's (FAO) Capture Production 1950-2014 dataset (Release date: March 2016 www.fao.org), International Committee for the Exploration of the Sea (ICES) 1950-2014 (www.ices.dk), Northwest Atlantic Fisheries Organisation (NAFO) Catch and Effort 1960-2014 (www.nafo.int), Southeast Atlantic (SEAFO) Capture Production 1975-2014 (Release date: June 2016) (www.seafo.org), General Fisheries Commission for the Mediterranean (GFCM) Capture production 1970-2014 (Release date: April 2016) (www.gfcm.org), Fishery Committee for the Eastern Central Atlantic (ECAF) Capture production 1970-2014 (Release date: May 2016) (www.fao.org/fishery/rfb/ecaf), Commission for the Conservation of Antarctic Marine Living Resources (CCAMLR) Statistical Bulletin 2016 Vol. 28 1970-2014 (www.ccamlr.org) and Sea Around Us project (SAUP) – records for FAO area 18 (Arctic) v1 1950 TO 2010 (extrapolated to 2014) (www.seaaroundus.org). See description in [1,2](and references therein) for fuller details of marine data treatment.

Aquaculture was sourced from FAO (Aquaculture Production (Quantities and values) 1950-2014 (Release date: March 2016 www.fao.org). Marine and brackish species aquaculture production was mapped to the reporting country's coastal cells equally. Both freshwater capture (from FAO capture sources) and freshwater species aquaculture production (from FAO's aquaculture source) were mapped to spatial cells within the reporting country which contained lakes, reservoirs or permanent rivers (codes 1-4 inclusive from the Global Lakes and Wetlands Database GLWD Level 3)[3]. Each reported tonnage was mapped equally to candidate cells.

Nutrient composition and nutrient requirements: The quantity of specific nutrients needed from the diet varies according to physiological requirements that vary by age, gender and for women, whether they are pregnant or lactating, and the extent to which a nutrient is utilized by the body, also known as

bioavailability. Where possible, we use the Recommended Dietary Allowance (RDA) which represents the average intake that would meet the needs of 97.5 percent of healthy individuals in a group. Where an RDA was not available for certain nutrients or population groups, we used Adequate Intake (AI), which is the level of intake assumed to be adequate for healthy individuals.
The measures used in this paper were developed by the US Institute of Medicine (*1*).
The nutrient requirements used in this paper are for a representative consumer, weighting each age and gender group by its population. We used the baseline population estimates for Shared Socioeconomic Pathway 2 (Fricko et al. 2016). The SSP population data set does not include the number of pregnant and lactating women so these were estimated by assuming that each is 20 percent of the number of children in the age group 0 to 4.

Calculation of diversity metrics: We calculated three diversity metrics based on all crop, livestock and fish products used in the analysis (Remans et al., 2014):

1. the Shannon diversity index, H, which reflects how many different types of foods are produced in a pixel, and how evenly these different types are distributed;
2. The species richness, S, a simple count of the number of commodities produced in each pixel;
3. the Modified Functional Attribute Diversity index (MFAD), the sum of pairwise distances between functional units in terms of nutrient composition for calcium, folate, iron, protein, vitamin A, vitamin B12 , zinc. This reflects the diversity in nutrient composition of foods produced in each pixel .

Shannon diversity metric (H)

$$H = \sum_{i=1}^{s} - (P_i * \ln P_i)$$

Where:

H = the Shannon diversity index
P_i = fraction of the entire population made up of species i
S = numbers of species encountered

Modified Functional Attribute Diversity (MFAD)

$$MFAD = \frac{\sum_{i=1}^{S}\sum_{j=1}^{S} d_{ij}}{N}$$

Where:

MFAD = Modified Functional Attribute Diversity
d_{ij} = Euclidean distance between functional traits of species
S = numbers of species encountered
∑ = sum from species 1 to species S
N=total number of # functional units

Table S1. Countries in the analysis and their regional allocation: Central America & the Caribbean (CAM), East Asia & the Pacific (EAP), Europe (EUR), North America (NAM), South Asia (SA), South America (SAM), Southeast Asia (SEA),sub-Saharan Africa (SSA), West Asia-North Africa (WANA). Other allocations: Australia and New Zealand (AUS) and China (CHN).

Australia	AUS	Lithuania	EUR	Cambodia	SEA	Rwanda	SSA
Belize	CAM	Luxembourg	EUR	Indonesia	SEA	Senegal	SSA
Costa Rica	CAM	Macedonia	EUR	Laos	SEA	Sierra Leone	SSA
Cuba	CAM	Montenegro	EUR	Malaysia	SEA	Somalia	SSA
Dominica	CAM	Netherlands	EUR	Myanmar	SEA	South Africa	SSA
Dominican Rep	CAM	Norway	EUR	Philippines	SEA	Swaziland	SSA
El Salvador	CAM	Poland	EUR	South Korea	SEA	Tanzania	SSA
Grenada	CAM	Portugal	EUR	Thailand	SEA	Togo	SSA
Guatemala	CAM	Romania	EUR	Viet Nam	SEA	Uganda	SSA
Haiti	CAM	Russia	EUR	Angola	SSA	Zambia	SSA
Honduras	CAM	Serbia	EUR	Benin	SSA	Zimbabwe	SSA
Jamaica	CAM	Slovakia	EUR	Botswana	SSA	Algeria	WANA
Mexico	CAM	Slovenia	EUR	Burkina Faso	SSA	Armenia	WANA
Nicaragua	CAM	Spain	EUR	Burundi	SSA	Azerbaijan	WANA
Panama	CAM	Sweden	EUR	Cameroon	SSA	Cyprus	WANA
Saint Lucia	CAM	Switzerland	EUR	Central Afr Rep	SSA	Egypt	WANA
St Vincent & Gren	CAM	Ukraine	EUR	Chad	SSA	Georgia	WANA
Trinidad & Tobago	CAM	UK	EUR	Congo	SSA	Iran	WANA
China	CHN	Canada	NAM	Côte d'Ivoire	SSA	Iraq	WANA
Japan	EAP	USA	NAM	Dem Republic Congo	SSA	Israel	WANA
Mongolia	EAP	New Zealand	AUS	Djibouti	SSA	Jordan	WANA
Papua New Guinea	EAP	Afghanistan	SA	Equatorial Guinea	SSA	Kazakhstan	WANA
Albania	EUR	Bangladesh	SA	Eritrea	SSA	Kuwait	WANA
Austria	EUR	Bhutan	SA	Ethiopia	SSA	Kyrgyzstan	WANA
Belarus	EUR	India	SA	Gabon	SSA	Lebanon	WANA
Belgium	EUR	Nepal	SA	Gambia	SSA	Libya	WANA
Bosnia & Herzegovina	EUR	Pakistan	SA	Ghana	SSA	Morocco	WANA
Bulgaria	EUR	Sri Lanka	SA	Guinea	SSA	Oman	WANA
Croatia	EUR	Argentina	SAM	Guinea-Bissau	SSA	Palestine	WANA
Czech Republic	EUR	Bolivia	SAM	Kenya	SSA	Qatar	WANA
Denmark	EUR	Brazil	SAM	Lesotho	SSA	Saudi Arabia	WANA
Estonia	EUR	Chile	SAM	Liberia	SSA	Sudan (former)	WANA
Finland	EUR	Colombia	SAM	Madagascar	SSA	Syria	WANA
France	EUR	Ecuador	SAM	Malawi	SSA	Tajikistan	WANA
Germany	EUR	Guyana	SAM	Mali	SSA	Tunisia	WANA
Greece	EUR	Paraguay	SAM	Mauritania	SSA	Turkey	WANA
Hungary	EUR	Peru	SAM	Mauritius	SSA	Turkmenistan	WANA
Iceland	EUR	Suriname	SAM	Mozambique	SSA	United Arab Emirates	WANA
Ireland	EUR	Uruguay	SAM	Namibia	SSA	Uzbekistan	WANA
Italy	EUR	Venezuela	SAM	Niger	SSA	Yemen	WANA
Latvia	EUR	Brunei Darussalam	SEA	Nigeria	SSA		

Table S2 - Food groups and commodities

Group	Commodities
Fruit	apple, banana, date, grapefruit, grape, orange, pineapple, plantain, citrusnes*, fruitnes*
Cereals	barley, maize, millet, oats, rice, rye, sorghum, wheat, cerealnes*
Pulses	bean, pea, pulsenes*
Roots & tubers	cassava, potato, sweetpotato, yam, rootnes*
Oil crops	coconut, olive, oilpalm, rapeseed, sesame, soybean, sunflower, oilseednes*
Fiber	cotton
Vegetables	onion, tomato, vegetablenes*
Sugar crops	sugarbeet, sugarcane
Livestock	Bovine meat, bovine milk, sheep and goat meat, sheep and goat milk, pork, eggs, poultry meat
Fish	Aquaculture, ClamOyster, DemersalFish, FreshwaterAmphibian, FreshwaterCrustacean, FreshwaterFish, FreshwaterMollusc, FreshwaterReptile, LobsterCrab, Plants, Shrimp, SmallPelagic, Squid, TunaSwordfish

* nes = ‘not elsewhere specified’, as used by FAO and mapped by EARTHStat (see p4).

2. Supplementary tables figures

Nutrient production maps

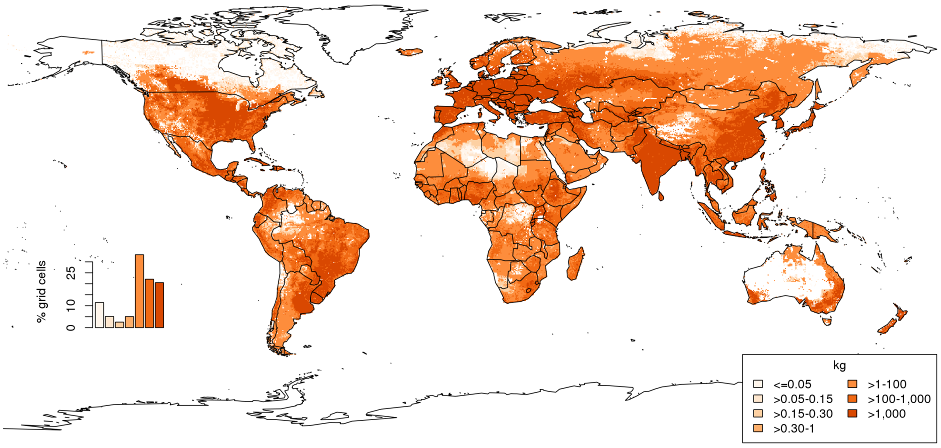

Figure S1. The spatial distribution of global calcium production (kg)

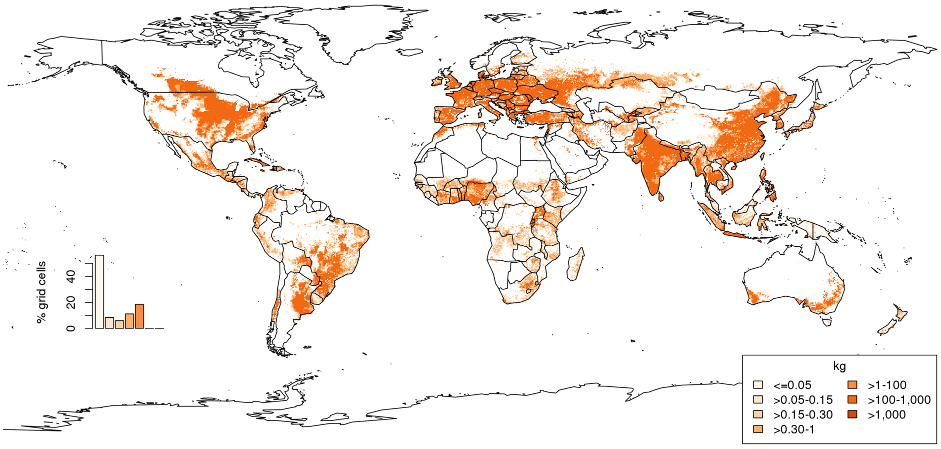

Figure S2. The spatial distribution of global folate production (kg)

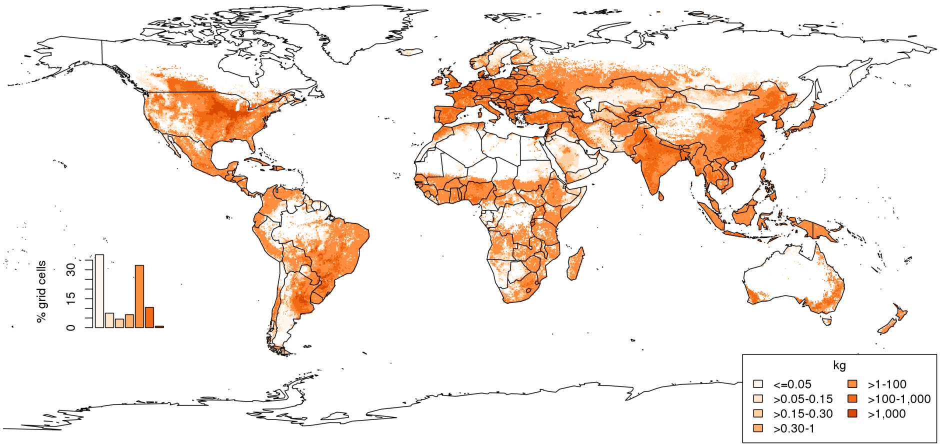

Figure S3. The spatial distribution of global iron production (kg)

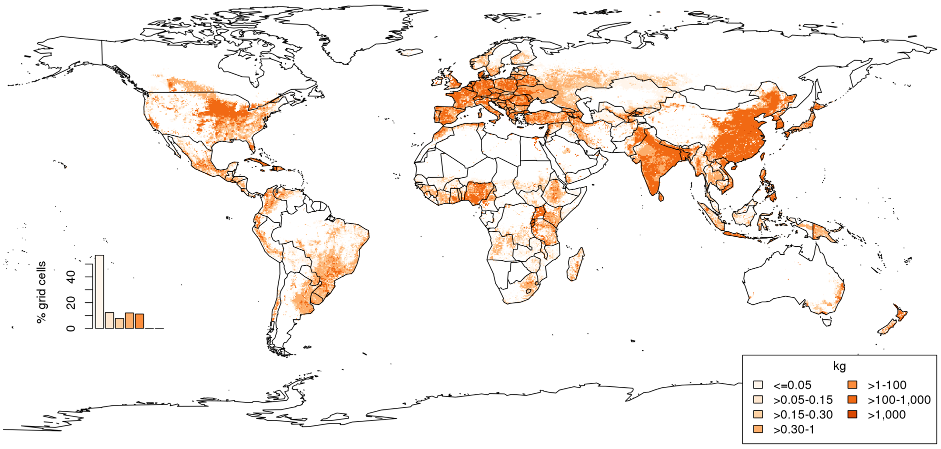

Figure S4. The spatial distribution of global vitamin A production (kg)

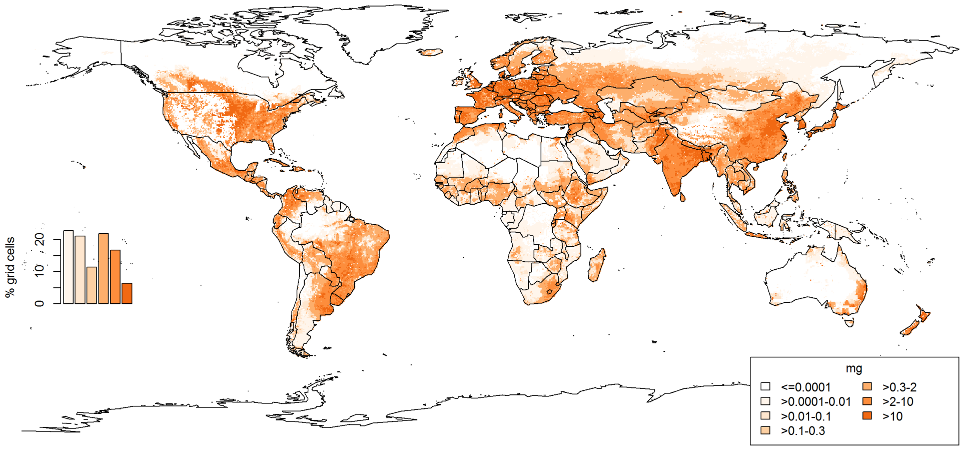

Figure S5. The spatial distribution of global vitamin B12 production (mg)

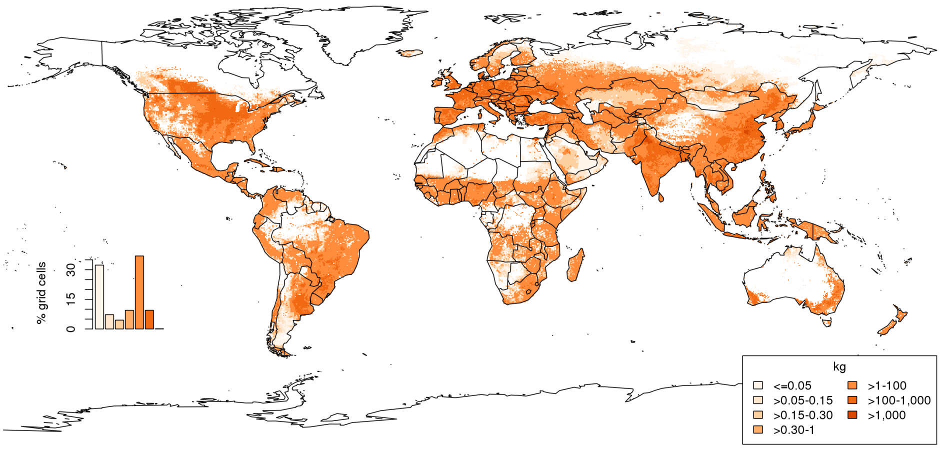

Figure S6. The spatial distribution of global zinc production (kg)

Nutritional diversity maps and tables

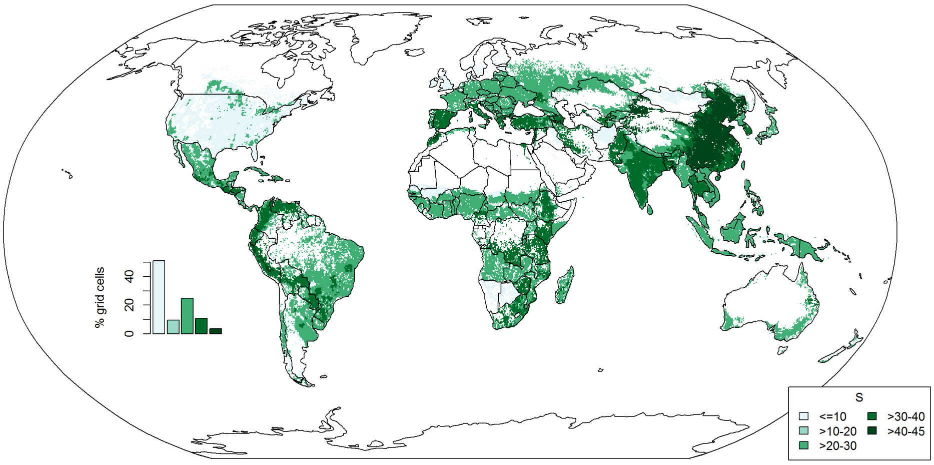

Figure S7. Global species richness (S) of food commodities

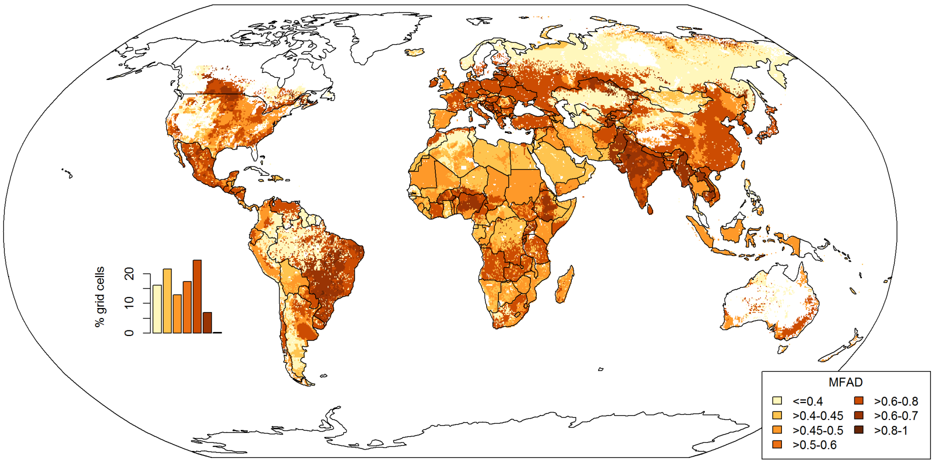

Figure S8. Global modified functional attribute diversity (MFAD) of food commodities

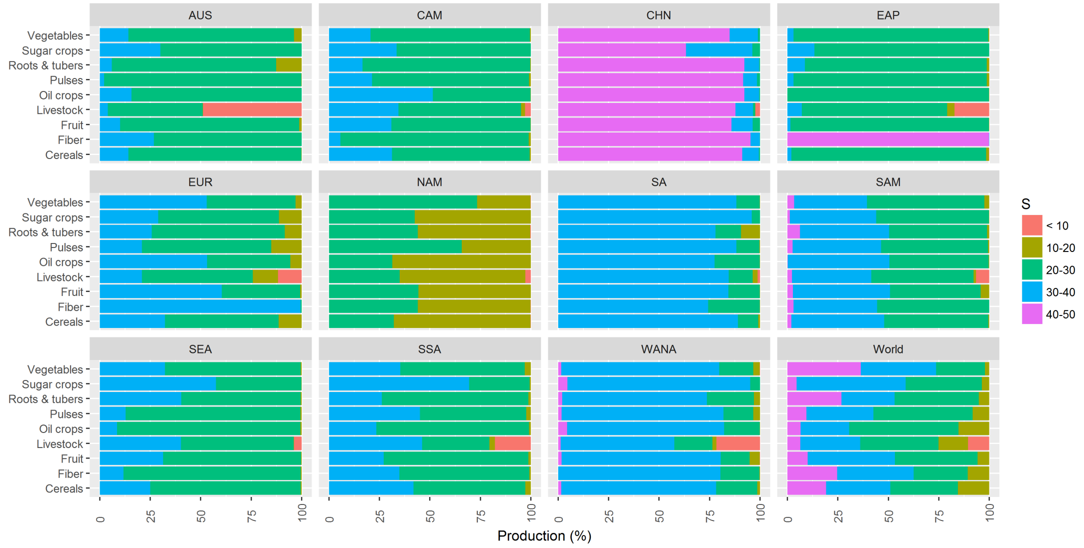

Figure S9. The regional relative production of different food commodity groups by species richness category

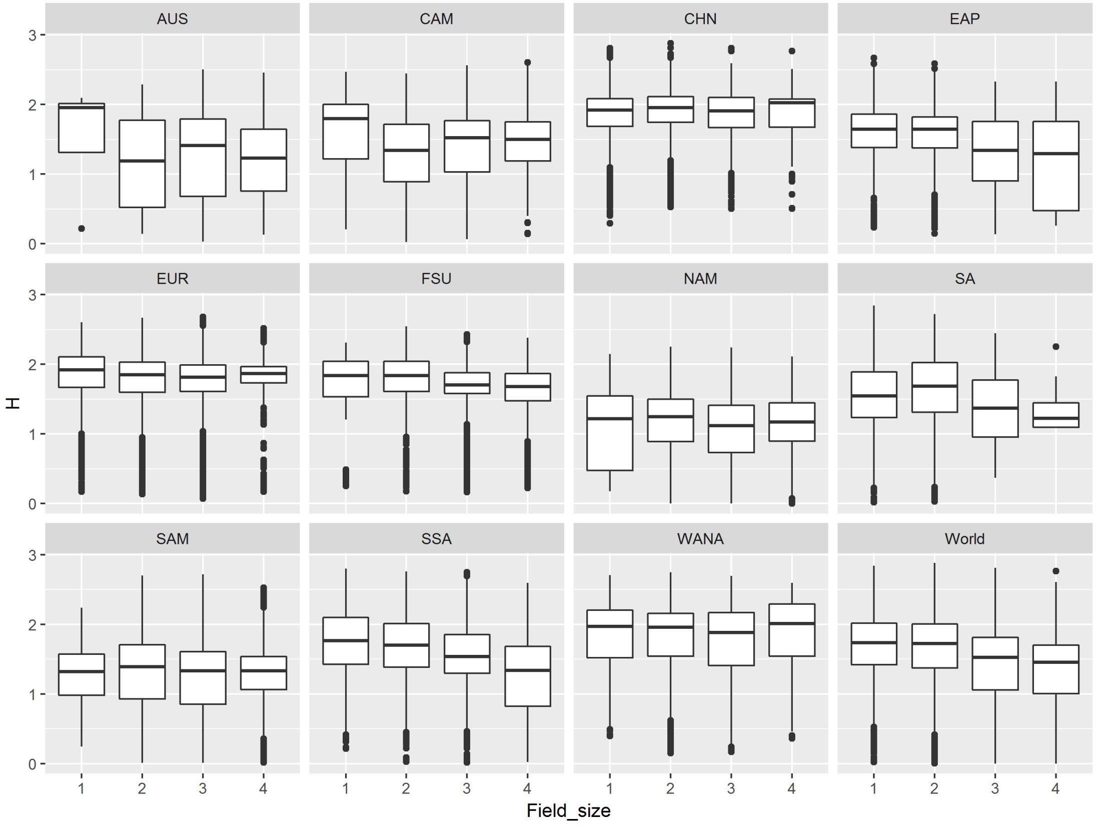

Figure S10. Boxplots of Shannon index by plot size by region. (1=<0.5 ha; 2=0.5-2 ha, 3=2-100 ha, 4= >100 ha)

3. Results data

	Calcium production (T)				
	**< 2 ha**	**2 - 20 ha**	**20 - 50 ha**	**50 - 200 ha**	**> 200 ha**
**Afghanistan**					
Cereals	340.87	840.42	108.94	364.96	0
Fiber	0.84	26.87	4.77	0.87	0
Fruit	4.81	28.34	4.5	0.84	0
Livestock	874.37	1141.23	105.16	18.59	0
Oil crops	132.86	169.06	14.89	2.72	0
Pulses	2.94	6.18	0.75	1.51	0
Roots & tubers	6.97	15.29	1.89	2.88	0
Sugar crops	0.43	0.84	0.1	0.02	0
Vegetables	0	0	0	0	0
**Albania**					
Cereals	18.7	59.09	15.17	15.22	0.71
Fiber	0.07	0.38	0.21	0.3	0.01
Fruit	1.77	6.98	2.73	3.51	0.16
Livestock	252.21	764.56	174.16	156.41	7.26
Oil crops	3.14	11.93	4.42	5.56	0.26
Pulses	5.85	17.12	3.46	2.7	0.13
Roots & tubers	2.3	7.61	2.19	2.4	0.11
Sugar crops	0.94	2.93	0.73	0.71	0.03
Vegetables	4.77	14.69	3.52	3.32	0.15
**Algeria**					
Cereals	144.17	536.12	158.86	182.1	246.97
Fiber	0.01	0.02	0.01	0.01	0.01
Fruit	63	260.17	88.08	48.93	20.78
Livestock	329.32	1228.11	363.15	332.93	370.95
Oil crops	33.99	103.38	25.43	39.25	63.74
Pulses	0.36	1.31	0.38	0.46	0.66
Roots & tubers	24.99	80.61	21.23	21.47	27.03
Sugar crops	0	0	0	0	0
Vegetables	29.8	112.5	33.9	25.98	23.13
**Angola**					
Cereals	14.58	28.6	3.13	1.82	0
Fiber	0.93	2.17	0.27	0.29	0
Fruit	12.59	24.28	2.65	1.95	0
Livestock	99.91	137.32	10.12	24.79	0
Oil crops	11.41	20.55	2.08	1.47	0
Pulses	32.61	75.17	9.24	9.22	0
Roots & tubers	375.68	872.3	107.92	77.61	0
Sugar crops	1.15	2.59	0.32	0.26	0
Vegetables	0.37	0.84	0.1	0.09	0
**Argentina**					

Cereals	4.23	100.11	153.22	812.62	5999.89
Fiber	1.31	22.33	24.26	66.41	356.18
Fruit	0.24	8.04	14.59	80.76	518.97
Livestock	71.57	1148.31	1127.6	2130.61	8080.54
Oil crops	44.44	1233.36	2102.55	12889.39	102048.76
Pulses	0.49	11.76	18.76	72	261.77
Roots & tubers	0.8	15.62	20.62	61.26	171.38
Sugar crops	0.58	13	19.99	66.59	149.2
Vegetables	0.11	3.21	5.51	28.41	174.33
**Armenia**					
Cereals	8.69	63.99	26.41	13.94	2.4
Fiber	0	0	0	0	0
Fruit	2.05	15.18	6.27	3.31	0.57
Livestock	83.48	434.06	159.22	82.34	14.15
Oil crops	0	0	0	0	0
Pulses	0.41	3.03	1.25	0.66	0.11
Roots & tubers	3.8	28.07	11.59	6.12	1.06
Sugar crops	0.26	1.39	0.54	0.27	0.05
Vegetables	2.65	21.12	8.86	4.71	0.81
**Australia**					
Cereals	0	14.99	64.65	604.94	8330.92
Fiber	0	1.48	6.71	65.36	1155.78
Fruit	0	0.59	2.7	24.89	325.75
Livestock	0	26.74	103.33	957.65	11173.54
Oil crops	0	7.03	29.91	270.29	2852.85
Pulses	0	0.2	0.93	9.09	162.04
Roots & tubers	0	0.33	1.34	11.95	108.69
Sugar crops	0	0.48	3.02	26.8	340.86
Vegetables	0	0.2	0.85	7.64	76.31
**Austria**					
Cereals	94.18	368.44	161.92	289.41	34.87
Fiber	0	0	0	0	0
Fruit	6.59	24.06	9.23	14.91	1.63
Livestock	159.17	1096.18	796.92	1642.47	201.51
Oil crops	57.13	218.88	92.87	162.91	19.42
Pulses	2.76	11.11	5.08	9.16	1.1
Roots & tubers	7.76	27.4	9.91	15.76	1.77
Sugar crops	39.88	143.25	54.22	90.16	10.61
Vegetables	2.3	9.26	4.23	7.63	0.91
**Azerbaijan**					
Cereals	97.93	314.79	84.76	67.34	67.56
Fiber	22.03	70.78	19.04	15.17	15.27
Fruit	2.96	9.5	2.55	2.02	2.01
Livestock	237.64	750.94	194.47	186.48	225.67
Oil crops	1.1	3.55	0.96	0.76	0.76
Pulses	2.07	7.81	2.38	1.7	1.37

Roots & tubers	14.64	46.57	12.39	9.92	10.07
Sugar crops	2.19	7.11	1.94	1.5	1.46
Vegetables	13.08	41.63	11.15	7.12	4.95
**Bangladesh**					
Cereals	2958.03	6554.35	812.54	1531.72	0
Fiber	13.21	29.56	3.71	3.99	0
Fruit	13.04	28.99	3.6	8.44	0
Livestock	1294.28	2306.44	253.38	452.65	0
Oil crops	203.79	561.05	75.88	140.09	0
Pulses	19.74	45.49	5.73	12.59	0
Roots & tubers	128.78	269.99	32.55	96.87	0
Sugar crops	14.84	31.1	3.76	9.32	0
Vegetables	42.76	91.76	11.21	21.53	0
**Belarus**					
Cereals	131.02	497.43	207.27	600.31	210.03
Fiber	0	0	0	0	0
Fruit	0.37	3.26	2.63	8.97	3.09
Livestock	548.58	2094.19	875.44	2626.61	954.41
Oil crops	37.92	144.01	60.23	172.47	59.36
Pulses	10.72	40.8	17.07	48.94	16.92
Roots & tubers	13.92	124.98	101.39	377.02	140.43
Sugar crops	7.3	68.33	56.75	221.05	84.73
Vegetables	4.61	18.06	7.81	20.86	6.69
**Belgium**					
Cereals	41.88	172.12	82.77	306.51	123.49
Fiber	0	0	0	0	0
Fruit	1.66	6.04	2.39	6.52	2.15
Livestock	94.53	672.1	497.56	1799.36	660.29
Oil crops	0.17	4.9	5.56	51.9	28.4
Pulses	0.15	0.61	0.29	0.74	0.2
Roots & tubers	33.82	112.99	35.82	61.76	12.52
Sugar crops	43.47	182.52	88.95	291.88	107.6
Vegetables	2.87	10.76	4.41	11.4	3.54
**Belize**					
Cereals	0.14	1.27	3.32	1.41	0.63
Fiber	0	0	0	0	0
Fruit	1.63	14.68	38.6	16.49	7.61
Livestock	0.5	1.34	1.69	0.8	0.54
Oil crops	0.03	0.24	0.64	0.27	0.13
Pulses	0.11	1.01	2.71	1.22	0.71
Roots & tubers	0	0.03	0.07	0.03	0.02
Sugar crops	0.22	2	5.29	2.32	1.19
Vegetables	0	0.01	0.03	0.01	0.01
**Benin**					
Cereals	24.82	49.44	5.55	0.03	0
Fiber	56.16	178.76	25.59	0.14	0

Fruit	4.67	7.86	0.75	0	0
Livestock	19.52	32.12	2.99	0.01	0
Oil crops	48.37	65.57	5	0.01	0
Pulses	48.75	83.9	8.34	0.03	0
Roots & tubers	299.44	374.93	23.62	0.03	0
Sugar crops	0.16	0.29	0.03	0	0
Vegetables	5.34	9.3	0.94	0	0
**Bhutan**					
Cereals	11.55	16.54	1.53	0.31	0
Fiber	0	0	0	0	0
Fruit	4.61	6.74	0.63	0.13	0
Livestock	20.06	29.14	2.7	0.55	0
Oil crops	1.55	2.31	0.22	0.04	0
Pulses	2.51	3.11	0.27	0.05	0
Roots & tubers	2.14	2.95	0.27	0.05	0
Sugar crops	0.05	0.07	0.01	0	0
Vegetables	0.05	0.06	0.01	0	0
**Bolivia (Plurinational State of)**					
Cereals	1.37	22.59	23.15	48.17	174.84
Fiber	0.31	5.38	5.99	16.46	79.91
Fruit	0.47	7.29	6.84	9.88	20.93
Livestock	4.71	74.66	71.9	111.05	209.99
Oil crops	3.62	86.28	133.32	620.2	3740.69
Pulses	0.4	6.28	6.02	9.71	25.82
Roots & tubers	1.51	23.52	21.8	28.91	42.75
Sugar crops	0.1	1.89	2.4	8.53	46.13
Vegetables	0.11	1.81	1.77	3.23	11.38
**Bosnia and Herzegovina**					
Cereals	16.91	64.48	27.8	51.09	6.56
Fiber	0	0	0	0	0
Fruit	0.45	1.87	0.91	1.77	0.23
Livestock	98.61	366.71	144.6	235.17	25.47
Oil crops	3.07	12.91	6.55	13.16	1.79
Pulses	1.85	7.06	3.04	5.49	0.68
Roots & tubers	3.92	14.75	6.23	11.34	1.45
Sugar crops	0	0	0	0	0
Vegetables	1.1	4.22	1.81	3.27	0.41
**Botswana**					
Cereals	0.51	2.33	0.37	1.12	0
Fiber	0.39	1.44	0.22	0.36	0
Fruit	0.01	0.17	0.03	0.01	0
Livestock	8.51	60.67	10.41	48.7	0
Oil crops	0.25	1.1	0.18	0.63	0
Pulses	0.23	1	0.16	0.83	0
Roots & tubers	0	0	0	0	0

Sugar crops	0	0	0	0	0
Vegetables	0.01	0.06	0.01	0.02	0
**Brazil**					
Cereals	14.4	261.89	312.27	1011.33	5508.62
Fiber	0.89	29.37	53.46	368.55	3107.16
Fruit	39.25	615.12	575.3	949.29	3699.11
Livestock	207.49	3304.91	3215.65	5821.2	20986.3
Oil crops	41.32	1171.94	2021	14533.91	131852.45
Pulses	38.98	607.66	565.06	818.43	2057.83
Roots & tubers	35.45	560.53	537.19	846.95	2005.11
Sugar crops	2.37	67.67	117.31	623.34	4022.89
Vegetables	4.72	74.73	71.63	115.61	309.35
**Brunei Darussalam**					
Cereals	0	0.21	0.03	0.01	0
Fiber	0	0	0	0	0
Fruit	0	0.2	0.03	0.01	0
Livestock	0	3.57	0.49	0.23	0
Oil crops	0	0.02	0	0	0
Pulses	0	0	0	0	0
Roots & tubers	0	0.31	0.04	0.02	0
Sugar crops	0	0	0	0	0
Vegetables	0	0	0	0	0
**Bulgaria**					
Cereals	46.35	264.29	173.19	601.14	215.87
Fiber	0.26	0.69	0.08	0.01	0
Fruit	3.52	12.05	4.15	9.37	2.73
Livestock	178.24	642.14	243.44	620.64	198.39
Oil crops	11.46	80.83	60.7	254.84	103.69
Pulses	0.59	2.61	1.37	4.41	1.55
Roots & tubers	2.31	10.02	5.42	12.03	2.02
Sugar crops	0.04	0.45	0.39	1.41	0.49
Vegetables	1.08	4.45	2.15	7.13	2.65
**Burkina Faso**					
Cereals	93.05	184.08	20.28	49.82	0
Fiber	161.98	300.21	32.7	121.76	0
Fruit	0.04	0.09	0.01	0.03	0
Livestock	166.38	223.23	16.58	30.68	0
Oil crops	25.12	87.97	13.13	107.54	0
Pulses	2.47	3.59	0.29	0.77	0
Roots & tubers	4.3	8.99	1.03	4.7	0
Sugar crops	0.3	1.63	0.27	2.31	0
Vegetables	0.24	0.52	0.06	0.14	0
**Burundi**					
Cereals	18.88	22.74	1.17	0	0
Fiber	2.21	2.66	0.14	0	0
Fruit	23.21	27.96	1.44	0	0

Livestock	19.93	24.04	1.27	0	0
Oil crops	3.35	4.03	0.2	0	0
Pulses	122.9	148.03	7.58	0.01	0
Roots & tubers	118.59	142.87	7.36	0.01	0
Sugar crops	0.53	0.63	0.03	0	0
Vegetables	0	0	0	0	0
**Cambodia**					
Cereals	492.98	1178.57	83.1	34.25	0
Fiber	0.09	0.16	0.01	0	0
Fruit	7.66	15.32	0.92	0.36	0
Livestock	19.28	35.22	1.72	0.61	0
Oil crops	314.05	528.41	27.08	10.3	0
Pulses	24.2	40.66	1.95	0.71	0
Roots & tubers	78.89	140.69	7.41	2.8	0
Sugar crops	0.7	1.07	0.04	0.02	0
Vegetables	0	0	0	0	0
**Cameroon**					
Cereals	68.39	105.22	9.1	5.74	0
Fiber	100.86	167.63	15.94	5.57	0
Fruit	27.99	43.06	3.68	6.27	0
Livestock	141.3	172.39	11.54	0.01	0
Oil crops	36.08	57.4	5.22	2.04	0
Pulses	137.94	167.39	9.51	0.02	0
Roots & tubers	128.76	228.1	22.82	82.22	0
Sugar crops	3.92	5.96	0.5	1.35	0
Vegetables	25.08	37.2	3.03	1.33	0
**Canada**					
Cereals	5.32	1191.03	1828.75	5466.7	5776.75
Fiber	0	0	0	0	0
Fruit	0	2.09	4.1	13.69	8.03
Livestock	7.8	1611.46	2252.92	3937.39	2286.05
Oil crops	14	3155.9	4832.41	13421.74	13161.21
Pulses	0.46	107.76	171.7	559.48	612.13
Roots & tubers	0.22	51.27	83.94	187.07	113.24
Sugar crops	0.01	2.58	4.1	31.62	53.71
Vegetables	0.03	9.11	18.11	54.63	34.81
**Central African Republic**					
Cereals	9.83	13.91	1.04	0	0
Fiber	1.3	1.57	0.08	0	0
Fruit	4.83	7.23	0.61	0	0
Oil crops	164.94	222.67	15.9	0.04	0
Pulses	2.55	4.77	0.51	0	0
Roots & tubers	56.02	72.01	4.69	0.01	0
Sugar crops	0.35	0.53	0.04	0	0
Vegetables	0	0	0	0	0
**Chad**					

Cereals	89.55	151.87	14.81	63.18	0
Fiber	43.47	81.44	8.7	13.97	0
Fruit	1.97	4.04	0.46	2.7	0
Livestock	112.5	157.22	12.63	22.25	0
Oil crops	115.75	183.91	16.81	41.15	0
Pulses	33.93	59.53	5.97	18.91	0
Roots & tubers	27.34	52.17	5.65	15.46	0
Sugar crops	1.14	2.13	0.23	0.39	0
Vegetables	1.5	2.38	0.21	0.95	0
**Chile**					
Cereals	4.43	72.46	74.03	150.83	530.04
Fiber	0	0	0	0	0
Fruit	1.9	30.78	30.76	59.62	213.27
Livestock	6.98	144.57	203.47	661.59	2003.5
Oil crops	0.82	13.3	13.38	26.2	91.85
Pulses	0.38	5.88	5.46	8.39	28.78
Roots & tubers	0.4	6.77	7.44	18.12	69.51
Sugar crops	1.49	24.14	24.28	47.55	166.69
Vegetables	0.99	16.03	16.12	31.57	110.7
**China**					
Cereals	56900.2	28457.28	0	10728.29	0
Fiber	8851.42	6376.87	0	4855.71	0
Fruit	2043.07	884.56	0	477.12	0
Livestock	38023.53	17124.77	0	7018.04	0
Oil crops	41081.43	24420.54	0	14306.24	0
Pulses	1364.74	673.42	0	470.26	0
Roots & tubers	17964.06	5267.84	0	1738.33	0
Sugar crops	896.51	790.83	0	293.44	0
Vegetables	4274.95	2002.14	0	849.49	0
**Colombia**					
Cereals	1.51	31.75	45.16	149.76	473.21
Fiber	0.99	15.83	15.37	25.59	66.44
Fruit	1.4	22.98	23.58	46.63	138.23
Livestock	56.71	906.57	885.13	1558.61	4859.03
Oil crops	0.84	13.58	13.45	28.57	140.29
Pulses	1.4	23.13	23.98	46.17	105.33
Roots & tubers	3.4	54.72	54.28	96.84	264.73
Sugar crops	2.84	45.98	46.26	83.14	197.05
Vegetables	0.91	14.48	14.09	23.63	61.15
**Congo**					
Cereals	0.58	0.81	0.06	0.01	0
Fiber	0	0	0	0	0
Fruit	1.17	3.05	0.4	0.27	0
Livestock	0.83	1.59	0.17	0.64	0
Oil crops	1.05	1.73	0.16	0.25	0
Pulses	2.08	3.55	0.34	0.61	0

Roots & tubers	47.43	87.84	9.09	3.43	0
Sugar crops	1.93	3.02	0.27	0.47	0
Vegetables	0.15	0.23	0.02	0.02	0
**Costa Rica**					
Cereals	0.07	3.67	13.86	13.03	22.09
Fiber	0	0.05	0.18	0.17	0.29
Fruit	0.38	21.39	80.92	76	128.69
Livestock	4.18	85.93	292.19	244.04	392.07
Oil crops	0	0.23	0.88	0.82	1.38
Pulses	0.01	0.85	3.27	3.12	5.31
Roots & tubers	0.02	1.6	6.13	5.73	9.49
Sugar crops	0.05	3.06	11.58	10.88	18.42
Vegetables	0.01	0.87	3.31	3.15	5.35
**Croatia**					
Cereals	37.16	135.27	53.61	155.48	54.29
Fiber	0	0	0	0	0
Fruit	2.33	7.87	2.65	6.86	2.35
Livestock	52.56	226.29	113.04	471.05	206.13
Oil crops	47.64	165.9	60.33	168.38	58.79
Pulses	0.49	1.76	0.69	1.98	0.69
Roots & tubers	2.49	8.62	3.07	8.29	2.86
Sugar crops	16.17	58.08	22.43	64.62	22.7
Vegetables	0.73	2.65	1.03	2.96	1.03
**Cuba**					
Cereals	13.99	31.65	30.21	21.98	34.32
Fiber	0	0	0	0	0
Fruit	9.48	21.5	20.74	20.68	45.64
Livestock	48.36	125.31	159.95	104.26	131.81
Oil crops	0.88	1.99	1.9	1.38	2.16
Pulses	12.29	27.79	26.53	19.3	30.14
Roots & tubers	20.93	47.31	45.1	32.83	51.3
Sugar crops	12.2	27.59	26.34	19.16	29.92
Vegetables	9.17	20.74	19.8	14.4	22.49
**Cyprus**					
Cereals	0	0	0	0	0
Fiber	0	0	0	0	0
Fruit	0.03	0.13	0.05	0.02	0
Livestock	30.6	141.55	52.5	25.47	4.51
Oil crops	0	0.01	0	0	0
Pulses	0	0.01	0	0	0
Roots & tubers	0	0	0	0	0
Sugar crops	0	0	0	0	0
Vegetables	0	0	0	0	0
**Czech Republic**					
Cereals	165.81	619.37	261.7	799.85	281.5
Fiber	0	0	0	0	0

Fruit	1.36	5.12	2.09	4.4	1
Livestock	233.81	925.97	413.6	1361.61	519.69
Oil crops	186.22	711.93	313.77	991.33	353.51
Pulses	2.79	9.75	3.59	9.39	3.01
Roots & tubers	3.56	17.82	11.25	33.57	9.56
Sugar crops	37.51	128.18	44.58	131.42	49.42
Vegetables	1.26	4.4	1.63	4.23	1.35
**Democratic Republic of the Congo**					
Cereals	46.49	75.6	6.96	19.73	0
Fiber	8.83	16.47	1.75	4.78	0
Fruit	29.03	50.46	4.98	15.29	0
Livestock	5.39	10.5	1.12	0.86	0
Oil crops	28.13	48.12	4.66	10.3	0
Pulses	45.7	74.74	6.81	17.4	0
Roots & tubers	582.46	1092.69	115.39	295.36	0
Sugar crops	4.34	7.96	0.83	2.57	0
Vegetables	4.34	7.94	0.83	2.57	0
**Denmark**					
Cereals	65.04	541.01	418.64	1408.72	489.6
Fiber	0	0	0	0	0
Fruit	0.04	0.33	0.26	0.86	0.3
Livestock	157.7	1267.42	958.63	2633.05	723.94
Oil crops	28.2	234.47	181.41	606.95	209.77
Pulses	0.27	2.28	1.77	5.96	2.07
Roots & tubers	3.02	25.25	19.59	66.06	22.97
Sugar crops	6.71	55.79	43.18	145.09	50.34
Vegetables	0.29	2.38	1.85	6.22	2.16
**Djibouti**					
Cereals	0	0	0	0	0
Fiber	0	0	0	0	0
Fruit	0	0	0	0	0
Livestock	0	0	0	0	0
Oil crops	0	0	0	0	0
Pulses	0	0	0	0	0
Roots & tubers	0	0	0	0	0
Sugar crops	0	0	0	0	0
Vegetables	0	0	0	0	0
**Dominica**					
Cereals	0	0	0	0	0
Fiber	0	0	0	0	0
Fruit	0.03	0.42	1.29	0.82	0.96
Livestock	0.08	0.91	2.7	1.61	1.71
Oil crops	0.01	0.08	0.26	0.17	0.19
Pulses	0	0	0	0	0
Roots & tubers	0.02	0.24	0.71	0.44	0.49

Sugar crops	0	0.01	0.02	0.01	0.01
Vegetables	0	0	0.01	0.01	0.01
**Ecuador**					
Cereals	6.02	93.73	87.06	117.24	185.94
Fiber	0.03	0.44	0.45	0.85	1.72
Fruit	3.45	52.49	46.55	52.24	79.18
Livestock	56.7	892.91	846.68	1277.74	2790.97
Oil crops	0.21	4.86	7.51	27.2	89.04
Pulses	0.27	4.15	3.9	5.49	9.51
Roots & tubers	0.29	4.77	5.04	10.05	22.02
Sugar crops	0.57	9.11	9	15.7	40.03
Vegetables	0.09	1.34	1.22	1.49	2.06
**Egypt**					
Cereals	590.67	2732.37	1013.49	491.63	87.15
Fiber	70.9	327.98	121.65	59.01	10.46
Fruit	184.98	855.67	317.39	153.96	27.29
Livestock	793.64	3671.28	1361.75	660.56	117.09
Oil crops	97.12	449.25	166.64	80.83	14.33
Pulses	8.65	40	14.84	7.2	1.28
Roots & tubers	38.92	180.06	66.79	32.4	5.74
Sugar crops	83.16	384.67	142.68	69.21	12.27
Vegetables	122.86	568.33	210.8	102.26	18.13
**El Salvador**					
Cereals	7.86	19.33	20.74	8.75	3.82
Fiber	0.24	0.54	0.47	0.2	0.09
Fruit	3.16	7.05	6.04	2.55	1.11
Livestock	118.44	237.84	141.06	59.54	25.98
Oil crops	7.08	15.18	11.53	4.87	2.12
Pulses	4.93	21.58	43.35	18.3	7.98
Roots & tubers	0.6	1.22	0.78	0.33	0.14
Sugar crops	9.77	19.51	11.32	4.78	2.08
Vegetables	0.61	1.27	0.88	0.37	0.16
**Equatorial Guinea**					
Cereals	0	0	0	0	0
Fiber	0	0	0	0	0
Fruit	0.68	0.82	0.04	0	0
Livestock	0.11	0.13	0.01	0	0
Oil crops	0.22	0.26	0.01	0	0
Pulses	0	0	0	0	0
Roots & tubers	10.18	12.24	0.6	0	0
Sugar crops	0	0	0	0	0
Vegetables	0	0	0	0	0
**Eritrea**					
Cereals	21.05	40.68	4.35	0.02	0
Fiber	0	0	0	0	0
Fruit	0	0	0	0	0

Livestock	48.66	87.51	8.96	0.04	0
Oil crops	29.95	74.09	9.45	0.05	0
Pulses	2.04	5.06	0.65	0	0
Roots & tubers	0.24	0.6	0.08	0	0
Sugar crops	0	0	0	0	0
Vegetables	0	0	0	0	0
**Estonia**					
Cereals	3.29	30.18	24.77	135.11	63.99
Fiber	0	0	0	0	0
Fruit	0	0.03	0.03	0.17	0.08
Livestock	8.1	109.29	105.85	437.69	166.52
Oil crops	3.29	30.73	25.49	147.27	71.4
Pulses	0.03	0.29	0.24	1.38	0.67
Roots & tubers	0.2	1.89	1.56	8.77	4.21
Sugar crops	0	0	0	0	0
Vegetables	0.01	0.11	0.09	0.5	0.24
**Ethiopia**					
Cereals	768.96	1530.21	170.12	188.72	0
Fiber	24.68	46.54	4.93	8.34	0
Fruit	6.4	12.05	1.28	2.16	0
Livestock	1080.33	1787.45	168.35	2.31	0
Oil crops	437.29	788.83	80.45	129.99	0
Pulses	131.91	174.33	11.51	7.57	0
Roots & tubers	50.21	94.52	10.01	16.89	0
Sugar crops	7.4	13.95	1.48	2.5	0
Vegetables	12.69	23.92	2.54	4.29	0
**Finland**					
Cereals	45.25	354.88	275.81	703.19	157.62
Fiber	0	0	0	0	0
Fruit	0.01	0.04	0.03	0.09	0.02
Livestock	147.03	793.88	495.59	1209.59	273.8
Oil crops	10.12	78.58	60.72	154.82	34.9
Pulses	0.1	0.79	0.61	1.55	0.35
Roots & tubers	1.72	13.67	10.72	27.97	6.51
Sugar crops	3.44	26.7	20.63	52.61	11.86
Vegetables	0.23	1.79	1.39	3.53	0.8
**France**					
Cereals	494.87	2743.44	1834.99	7875.87	3246.85
Fiber	0	0	0	0	0
Fruit	41.68	215.31	121.85	290.46	74.29
Livestock	2942.28	10999.81	4586.42	10378.36	2499.82
Oil crops	418.22	2094.41	1303.45	6510.16	2953.52
Pulses	20.67	92.4	49.37	158.13	54.63
Roots & tubers	5.95	79.84	79.43	321.64	117.66
Sugar crops	18.97	323.19	346.14	2158.11	1024.15
Vegetables	6.16	27.67	14.81	46.97	16.11

**Gabon**					
Cereals	0.78	1.02	0.07	0	0
Fiber	0	0	0	0	0
Fruit	2.32	3.22	0.25	0	0
Livestock	1.49	3.08	0.36	0	0
Oil crops	2.45	3.19	0.23	0	0
Pulses	0.04	0.05	0	0	0
Roots & tubers	23.51	31.54	2.41	0.01	0
Sugar crops	0.84	1.21	0.1	0	0
Vegetables	0.01	0.02	0	0	0
**Gambia**					
Cereals	6.94	11.67	1.13	0	0
Fiber	0.19	0.3	0.03	0	0
Fruit	0	0	0	0	0
Livestock	2.91	7.46	0.98	0.01	0
Oil crops	9.03	15.02	1.45	0.01	0
Pulses	0.36	0.58	0.05	0	0
Roots & tubers	0.48	0.69	0.06	0	0
Sugar crops	0	0	0	0	0
Vegetables	0	0	0	0	0
**Georgia**					
Cereals	7.26	38.3	14.2	7.32	1.26
Fiber	0	0	0	0	0
Fruit	2.22	14.28	5.76	2.98	0.52
Livestock	95.15	480.76	173.6	89.8	15.42
Oil crops	2.46	12.91	4.77	2.46	0.42
Pulses	1.88	9.78	3.61	1.86	0.32
Roots & tubers	2.58	13.94	5.22	2.7	0.46
Sugar crops	0	0	0	0	0
Vegetables	1.51	8.03	2.99	1.54	0.27
**Germany**					
Cereals	477.12	2614.56	1647.66	5750.04	2110.09
Fiber	0	0	0	0	0
Fruit	26.59	83.63	22.55	39.52	10.45
Livestock	1034.52	6184.28	4181.46	16670.74	6679.56
Oil crops	507.67	2699.02	1697.14	6848.88	2779.98
Pulses	5.32	24.08	12.76	43.66	16.38
Roots & tubers	31.74	189.78	126.34	470.03	181.07
Sugar crops	79.77	632.17	473.4	1342.98	390.28
Vegetables	4.58	20.17	10.27	32.85	11.83
**Ghana**					
Cereals	64.68	95.99	7.78	0.03	0
Fiber	6.81	12.31	1.27	0.01	0
Fruit	84.62	115.95	8.45	0.02	0
Livestock	27.33	36.3	2.47	0.01	0
Oil crops	7.63	13.88	1.44	0.01	0

Pulses	82.8	99.9	4.97	0	0
Roots & tubers	784.39	1086.69	81.44	0.21	0
Sugar crops	0.62	0.75	0.05	0	0
Vegetables	9.15	15.27	1.46	0.01	0
**Greece**					
Cereals	92.23	370.5	161.83	265.69	26.82
Fiber	107.81	430.02	191.68	335.98	38.6
Fruit	41.87	159.36	63.96	100.53	9.87
Livestock	396.42	1401.49	494.94	734.82	72.15
Oil crops	193.88	750.47	303.68	460.96	40.79
Pulses	3.09	11.35	4.23	6.21	0.55
Roots & tubers	7.54	31.18	14.41	25.01	2.74
Sugar crops	23.8	91.22	37.42	60.74	6.31
Vegetables	20.61	77.01	30.16	47.48	4.8
**Grenada**					
Cereals	0	0	0	0	0
Fiber	0	0	0	0	0
Fruit	0	0	0	0	0
Livestock	0	0	0	0	0
Oil crops	0	0	0	0	0
Pulses	0	0	0	0	0
Roots & tubers	0	0	0	0	0
Sugar crops	0	0	0	0	0
Vegetables	0	0	0	0	0
**Guatemala**					
Cereals	3.96	13.02	22.97	15.04	18.11
Fiber	0.21	0.77	1.45	0.84	0.85
Fruit	5.83	21.26	39.95	23.21	23.83
Livestock	44.46	134.79	210.47	119.61	118.66
Oil crops	24.32	89.25	168.33	96.97	98.28
Pulses	10.32	31.93	52.55	33.65	39.46
Roots & tubers	2.13	7.79	14.66	8.47	8.62
Sugar crops	9.4	34.7	65.77	37.98	38.65
Vegetables	2.72	9.78	18.24	10.73	11.24
**Guinea**					
Cereals	147.46	255.07	25.44	0.1	0
Fiber	13.3	27.1	3.08	0.01	0
Fruit	6.48	13.27	1.51	0.01	0
Livestock	51.6	88.79	8.77	0.03	0
Oil crops	3.02	4.34	0.36	0	0
Pulses	3.72	12.52	1.82	0.01	0
Roots & tubers	61.93	116.28	12.43	0.05	0
Sugar crops	0.89	1.73	0.19	0	0
Vegetables	0	0	0	0	0
**Guinea-Bissau**					
Cereals	17.08	20.67	1.2	0	0

Fiber	2.33	2.82	0.17	0	0
Fruit	1.38	1.67	0.1	0	0
Livestock	12.46	15.09	0.9	0	0
Oil crops	1.45	1.76	0.1	0	0
Pulses	0.31	0.38	0.02	0	0
Roots & tubers	2.45	2.97	0.17	0	0
Sugar crops	0.02	0.03	0	0	0
Vegetables	0	0	0	0	0
**Guyana**					
Cereals	0.02	1.28	2.77	16.87	105.09
Fiber	0	0	0	0	0
Fruit	0	0.06	0.1	0.41	1.6
Livestock	0.06	1.55	2.51	9.63	32.57
Oil crops	0.01	0.22	0.35	1.29	4.05
Pulses	0	0	0.01	0.03	0.12
Roots & tubers	0	0.08	0.14	0.61	2.54
Sugar crops	0.03	0.71	1.23	5.35	22.94
Vegetables	0	0.01	0.01	0.04	0.15
**Haiti**					
Cereals	8.68	19.66	17.51	7.39	3.22
Fiber	0.22	0.5	0.44	0.19	0.08
Fruit	3.82	8.65	7.7	3.25	1.42
Livestock	20.01	41.65	28.53	12.04	5.25
Oil crops	6.67	15.07	13.32	5.62	2.45
Pulses	9.8	22.16	19.65	8.3	3.62
Roots & tubers	20.81	47	41.52	17.53	7.65
Sugar crops	1.64	3.73	3.35	1.41	0.62
Vegetables	0.26	0.6	0.55	0.23	0.1
**Honduras**					
Cereals	3.88	7.98	5.59	6.99	18.12
Fiber	0.18	0.37	0.28	0.31	0.76
Fruit	10.84	22.74	16.92	18.56	45.21
Livestock	122.72	247.89	159.9	130.28	245.75
Oil crops	1.44	2.98	2.14	2.45	6.09
Pulses	4.92	10.87	9.81	16.71	48.94
Roots & tubers	0.71	1.45	0.96	0.93	2.12
Sugar crops	4.98	10.5	8.01	9.61	24.5
Vegetables	1.78	3.72	2.71	2.95	7.14
**Hungary**					
Cereals	187.33	728.22	307.49	825.31	272.63
Fiber	0	0	0	0	0
Fruit	4.62	19.88	9.6	27.67	9.41
Livestock	177.54	744.47	343.84	869.32	262.22
Oil crops	109.5	456.86	215.51	668.48	241.99
Pulses	1.19	5.68	3.07	8.34	2.55
Roots & tubers	5.05	18.52	7.07	17.38	5.51

Sugar crops	32.69	117.51	43.97	95.52	25
Vegetables	4.33	14.27	4.37	10.13	3.4
**Iceland**					
Cereals	0	0	0	0	0
Fiber	0	0	0	0	0
Fruit	0	0	0	0	0
Livestock	0	0	0	0	0
Oil crops	0	0	0	0	0
Pulses	0	0	0	0	0
Roots & tubers	0	0	0	0	0
Sugar crops	0	0	0	0	0
Vegetables	0	0	0	0	0
**India**					
Cereals	20448.54	40417.96	4800.5	906.65	0
Fiber	2673.59	7896.64	1090.85	206.14	0
Fruit	579.08	1321.1	165.99	31.75	0
Livestock	34387.37	78076.96	9788.19	1872.97	0
Oil crops	15254.13	34744.01	4355.04	835.16	0
Pulses	1199.32	2995.35	388.17	74.65	0
Roots & tubers	1297.99	2288.6	255.58	48.23	0
Sugar crops	714.64	1896.16	253.98	47.85	0
Vegetables	1094.43	1889.83	204.94	39.79	0
**Indonesia**					
Cereals	5359.8	7544.23	287.82	2855.68	0
Fiber	1.47	2.59	0.14	1.34	0
Fruit	257.87	453.96	24.07	234.88	0
Livestock	782.44	1066.82	35.38	127.28	0
Oil crops	1211.51	1644.02	56.63	406.37	0
Pulses	108.08	190.16	10.07	98.45	0
Roots & tubers	1182.86	1686.41	61.42	219.58	0
Sugar crops	74.13	130.43	6.91	67.53	0
Vegetables	58.14	102.29	5.42	52.96	0
**Iran (Islamic Republic of)**					
Cereals	1684.78	3467.62	410.31	1351.16	0
Fiber	102.23	168.59	17.52	31.7	0
Fruit	408.4	914.19	112.51	298.85	0
Livestock	1681.41	5409.42	758.32	1816.85	0
Oil crops	197.72	1210.84	194.49	186.97	0
Pulses	66.42	149.28	18.46	53.93	0
Roots & tubers	83.7	168.28	19.61	120.65	0
Sugar crops	91.11	392.25	59.08	209.15	0
Vegetables	192.73	413.22	49.53	206.94	0
**Iraq**					
Cereals	193.51	626.05	166.77	94.21	41.99
Fiber	3.32	15.38	5.7	2.77	0.49
Fruit	36.87	158.39	54.93	29.57	10.57

Livestock	45.95	219.98	77.64	53.05	37.15
Oil crops	1.49	6.42	2.19	1.09	0.19
Pulses	1.3	4.45	1.24	0.68	0.25
Roots & tubers	6.38	33.09	11.96	8.45	6.12
Sugar crops	0.1	0.84	0.36	0.19	0.03
Vegetables	13.37	64.25	24.16	11.81	2.09
**Ireland**					
Cereals	22.57	146.72	104.23	323.92	101.78
Fiber	0	0	0	0	0
Fruit	0.08	0.53	0.37	1.14	0.37
Livestock	565.98	2381.01	1134.16	2148.7	319.58
Oil crops	1.97	12.5	8.68	27.19	8.75
Pulses	0.6	3.77	2.61	8.17	2.63
Roots & tubers	1.18	7.54	5.25	16.46	5.29
Sugar crops	3.16	31.14	26.57	84.77	26.23
Vegetables	0.09	0.58	0.4	1.25	0.4
**Israel**					
Cereals	12.09	73.52	29.28	15.03	2.62
Fiber	10.59	55.69	21.17	10.68	1.86
Fruit	24.62	111.1	39.43	19.57	3.41
Livestock	353.91	1748.03	641.83	324.11	56.34
Oil crops	7.82	38.76	14.44	7.22	1.26
Pulses	0.05	0.23	0.08	0.04	0.01
Roots & tubers	14.2	65.58	23.75	11.74	2.06
Sugar crops	0	0	0	0	0
Vegetables	14.6	65.96	23.53	11.64	2.04
**Italy**					
Cereals	140.29	870.19	598.48	1842.96	580.37
Fiber	0	0	0	0	0
Fruit	61.93	357.2	230.23	708.9	231.36
Livestock	699.41	4283.28	2893.16	6109.85	905.94
Oil crops	242.37	1024.65	497.82	1632.86	608.59
Pulses	1.6	8.7	5.38	13.88	3.61
Roots & tubers	10.91	46.56	23.36	63.52	18.69
Sugar crops	32.44	241.83	180.33	428.07	86.33
Vegetables	32.76	178.27	110.38	288	75.82
**Ivory Coast**					
Cereals	0	0	0	0	0
Fiber	0	0	0	0	0
Fruit	0	0	0	0	0
Livestock	7.28	37.46	6.13	3.57	0
Oil crops	0	0	0	0	0
Pulses	0	0	0	0	0
Roots & tubers	0	0	0	0	0
Sugar crops	0	0	0	0	0
Vegetables	0	0	0	0	0

**Jamaica**					
Cereals	0.03	0.05	0	0	0
Fiber	0	0	0	0	0
Fruit	13.27	22.3	1.89	0.8	0.35
Livestock	83.28	148.9	40.38	17.05	7.44
Oil crops	7.4	12.44	1.05	0.44	0.19
Pulses	0.2	0.33	0.03	0.01	0.01
Roots & tubers	8.67	14.59	1.29	0.55	0.24
Sugar crops	5.82	9.79	0.83	0.35	0.15
Vegetables	0.68	1.14	0.1	0.04	0.02
**Japan**					
Cereals	1215.03	1387.66	525.33	304.17	0
Fiber	0	0	0	0	0
Fruit	24.89	35.4	14.48	11.23	0
Livestock	3290.24	3920.21	1469.51	2501.81	0
Oil crops	195.36	253.61	100.92	81.58	0
Pulses	33.83	48.11	19.68	15.26	0
Roots & tubers	214.66	181.09	57.76	39.54	0
Sugar crops	156.64	213.83	86.36	66.67	0
Vegetables	89.81	127.71	52.24	40.51	0
**Jordan**					
Cereals	2.08	9.63	3.57	1.73	0.31
Fiber	0	0	0	0	0
Fruit	2.57	11.88	4.41	2.14	0.38
Livestock	52.87	244.59	90.72	44.01	7.8
Oil crops	10.91	50.47	18.72	9.08	1.61
Pulses	0	0	0	0	0
Roots & tubers	1.55	7.16	2.66	1.29	0.23
Sugar crops	0	0	0	0	0
Vegetables	6.65	30.78	11.42	5.54	0.98
**Kazakhstan**					
Cereals	76.12	1350.4	637.51	1277.43	2203.12
Fiber	21.53	163.53	69.41	81.65	111.24
Fruit	1.07	5.96	2.35	1.33	0.55
Livestock	364.6	2545.94	1061.99	969.9	1068.08
Oil crops	15.26	178.5	80.98	102.02	142.61
Pulses	0.42	4.74	2.14	3.27	5.1
Roots & tubers	8.48	81.52	36.02	40.42	52.47
Sugar crops	1.17	18.67	8.74	6.33	4.48
Vegetables	8.4	54.33	22.27	16.33	13.37
**Kenya**					
Cereals	75.1	157.6	18.21	66.82	0
Fiber	5.1	11	1.29	5.1	0
Fruit	18.48	40.48	4.81	17.67	0
Livestock	1084.16	2279.47	264.94	941.51	0
Oil crops	26.5	55.12	6.33	25.46	0

Pulses	148.1	294.84	32.86	97.67	0
Roots & tubers	101.25	219.23	25.81	94.01	0
Sugar crops	11.37	24.61	2.9	10.91	0
Vegetables	15.79	34.2	4.03	15.18	0
**Kuwait**					
Cereals	0	0.48	0.24	0.14	0.02
Fiber	0	0	0	0	0
Fruit	0	5.1	2.59	1.45	0.25
Livestock	0	42.03	21.34	11.95	2.04
Oil crops	0	0.02	0.01	0	0
Pulses	0	0	0	0	0
Roots & tubers	0	1.06	0.54	0.3	0.05
Sugar crops	0	0	0	0	0
Vegetables	0	4.34	2.2	1.23	0.21
**Kyrgyzstan**					
Cereals	27.08	138.21	49.7	67.06	99.21
Fiber	7	41.92	16.01	19.17	26.12
Fruit	0.55	2.86	1.04	1.39	2.05
Livestock	226.09	788.86	221.92	150.03	106.61
Oil crops	2.48	12.59	4.47	4.51	5.38
Pulses	2.54	39.99	18.7	10.18	1.83
Roots & tubers	8.04	40.69	14.58	19.97	29.82
Sugar crops	1.77	9.59	3.55	5.02	7.6
Vegetables	2.82	14.51	5.24	6.93	10.14
**Lao People's Democratic Republic**					
Cereals	274.18	459.89	22.26	8.16	0
Fiber	2.82	4.07	0.14	0.04	0
Fruit	6.7	13.32	0.81	0.33	0
Livestock	8.29	14.49	0.71	0.26	0
Oil crops	40.63	58.69	1.97	0.58	0
Pulses	2.81	5.09	0.26	0.1	0
Roots & tubers	18.29	29.86	1.43	0.53	0
Sugar crops	0.7	1.61	0.11	0.04	0
Vegetables	0	0	0	0	0
**Latvia**					
Cereals	8.21	67.53	51.9	220.68	92.91
Fiber	0	0	0	0	0
Fruit	0.04	0.31	0.23	0.89	0.36
Livestock	22.21	177.06	133.12	487.35	185.59
Oil crops	8.34	68.32	52.37	212.14	86.52
Pulses	0.03	0.27	0.21	0.78	0.3
Roots & tubers	1	8.26	6.38	27.93	11.96
Sugar crops	0.67	5.69	4.47	21.06	9.37
Vegetables	0.08	0.67	0.5	1.94	0.77
**Lebanon**					

Cereals	5.11	23.46	8.34	8.93	11.7
Fiber	0	0	0	0	0
Fruit	6.92	35.08	12.83	14.51	19.4
Livestock	24.92	124.08	45.59	59.11	86.98
Oil crops	8.98	43.03	15.54	8.65	3.22
Pulses	0.09	0.46	0.17	0.2	0.27
Roots & tubers	1.32	12.04	5.28	9.09	15
Sugar crops	0.44	2.33	0.87	1.07	1.5
Vegetables	2.58	14.14	5.4	6.07	8.09
**Lesotho**					
Cereals	2.18	5.39	0.69	0	0
Fiber	0	0	0	0	0
Fruit	0	0	0	0	0
Livestock	10.18	25.03	3.21	0.02	0
Oil crops	0	0	0	0	0
Pulses	1.53	2.69	0.27	0	0
Roots & tubers	2.01	5.91	0.82	0	0
Sugar crops	0	0	0	0	0
Vegetables	0	0	0	0	0
**Liberia**					
Cereals	14.53	32.87	3.97	0.02	0
Fiber	0	0	0	0	0
Fruit	2.13	5.1	0.64	0	0
Livestock	0.68	3.5	0.57	0	0
Oil crops	2.7	6.6	0.83	0	0
Pulses	0.27	0.66	0.08	0	0
Roots & tubers	20.83	50.1	6.27	0.03	0
Sugar crops	0.64	1.72	0.23	0	0
Vegetables	0.03	0.1	0.02	0	0
**Libya**					
Cereals	6.13	26.58	8.59	12.01	18.06
Fiber	0	0	0	0	0
Fruit	8.78	38.75	12.69	18.03	27.34
Livestock	17.83	143.8	61.4	60.66	71.06
Oil crops	10.5	46.32	15.17	21.55	32.68
Pulses	0.27	1.18	0.39	0.55	0.83
Roots & tubers	2.1	9.25	3.03	4.3	6.53
Sugar crops	0	0	0	0	0
Vegetables	4.69	20.68	6.77	9.62	14.59
**Lithuania**					
Cereals	34.46	175.52	105.05	348.71	122.78
Fiber	0	0	0	0	0
Fruit	0.33	1.28	0.56	1.57	0.51
Livestock	103.1	513.73	303	1022.14	365.23
Oil crops	23.61	132.1	83.84	286.85	103.97
Pulses	1.29	5.09	2.24	6.18	2.02

Roots & tubers	3.14	14.34	7.7	24.27	8.38
Sugar crops	1.51	15.49	13.47	47.99	16.64
Vegetables	0.28	1.11	0.49	1.36	0.45
**Luxembourg**					
Cereals	0	7.39	9.47	25.23	4.02
Fiber	0	0	0	0	0
Fruit	0	0.32	0.41	1.08	0.17
Livestock	0	52.28	66.97	178.41	28.41
Oil crops	0	7.29	9.34	24.88	3.96
Pulses	0	0.1	0.13	0.35	0.06
Roots & tubers	0	0.27	0.34	0.92	0.15
Sugar crops	0	0	0	0	0
Vegetables	0	0	0	0.01	0
**Madagascar**					
Cereals	438.63	530.96	30.9	0.02	0
Fiber	5.74	6.95	0.41	0	0
Fruit	18	21.8	1.28	0	0
Livestock	285.56	345.4	19.6	0.01	0
Oil crops	2.75	3.33	0.2	0	0
Pulses	48.22	58.28	3.26	0	0
Roots & tubers	268.17	324.54	18.77	0.01	0
Sugar crops	8.11	15.94	1.73	0.01	0
Vegetables	2.05	2.48	0.15	0	0
**Malawi**					
Cereals	50.47	74.38	6.51	9.22	0
Fiber	20.99	32.09	2.83	1.5	0
Fruit	6.41	10.36	0.99	0.64	0
Livestock	23.63	28.92	1.57	1.23	0
Oil crops	68.49	82.7	4.29	0.04	0
Pulses	58.99	80.23	6.36	2.66	0
Roots & tubers	219.39	308.01	24.94	17.72	0
Sugar crops	8.53	13.8	1.32	0.85	0
Vegetables	5.13	8.29	0.79	0.51	0
**Malaysia**					
Cereals	37.71	279.69	32.2	295.03	0
Fiber	0	0	0	0	0
Fruit	9.78	21.75	1.54	9.84	0
Livestock	67.33	232.47	20.2	61.11	0
Oil crops	9	19.85	1.4	8.91	0
Pulses	0	0	0	0	0
Roots & tubers	2.78	7.55	0.62	3.54	0
Sugar crops	1.18	2.4	0.15	1.16	0
Vegetables	0.95	2.06	0.14	0.91	0
**Mali**					
Cereals	180.31	303.49	29.47	0.11	0
Fiber	133.23	245.55	25.85	0.11	0

Fruit	3.36	4.84	0.41	0	0
Livestock	468.68	976.92	113.74	0.53	0
Oil crops	18.17	25.71	2.18	0.01	0
Pulses	0.04	0.05	0	0	0
Roots & tubers	23.35	45.32	4.93	0.02	0
Sugar crops	1.01	2.1	0.25	0	0
Vegetables	4.56	10.93	1.37	0.01	0
**Mauritania**					
Cereals	10.36	20.36	2.2	0.01	0
Fiber	0	0	0	0	0
Fruit	4.01	7.4	0.76	0	0
Livestock	191.79	294.26	24.55	0.09	0
Oil crops	0	0	0	0	0
Pulses	3.39	4.94	0.38	0	0
Roots & tubers	0.39	0.64	0.06	0	0
Sugar crops	0	0	0	0	0
Vegetables	0	0	0	0	0
**Mauritius**					
Cereals	0	0	0	0	0
Fiber	0	0	0	0	0
Fruit	0	0	0	0	0
Livestock	0	0	0	0	0
Oil crops	0	0	0	0	0
Pulses	0	0	0	0	0
Roots & tubers	0	0	0	0	0
Sugar crops	0	0	0	0	0
Vegetables	0	0	0	0	0
**Mexico**					
Cereals	288.92	699.61	796.46	590.3	902.21
Fiber	0.14	7.12	26.8	86.96	288.22
Fruit	49.67	214.59	443.55	286.18	416.55
Livestock	1261.11	3098.87	3537.23	2408.15	3485.18
Oil crops	166.36	295.34	76.55	50.82	77.85
Pulses	280.3	524.28	219.56	140.33	194.83
Roots & tubers	15.57	37.56	41.83	28.53	39.58
Sugar crops	57.83	139.99	150.11	82.03	84.75
Vegetables	47.2	108.62	110.58	94.51	173.62
**Mongolia**					
Cereals	5.88	8.25	3.26	20.3	0
Fiber	0	0	0	0	0
Fruit	0	0	0	0	0
Livestock	159.11	155.01	51.97	151.09	0
Oil crops	0.59	13.68	7.14	4.21	0
Pulses	0.06	0.08	0.03	0.2	0
Roots & tubers	1.45	1.99	0.78	4.97	0
Sugar crops	0	0	0	0	0

Vegetables	0.09	0.13	0.05	0.32	0
**Montenegro**					
Cereals	0.28	0.96	0.29	0.33	0.02
Fiber	0	0	0	0	0
Fruit	0.89	3	0.89	0.98	0.05
Livestock	314.96	1124.4	376.46	450.39	20.93
Oil crops	0.2	0.62	0.14	0.13	0.01
Pulses	0.29	0.99	0.3	0.34	0.02
Roots & tubers	1.63	5.52	1.67	1.88	0.09
Sugar crops	0	0	0	0	0
Vegetables	0.41	1.4	0.42	0.47	0.02
**Morocco**					
Cereals	221.68	790.68	229.58	229.19	282.78
Fiber	0.03	0.11	0.03	0.03	0.03
Fruit	44.44	150.95	42.01	38.23	43.5
Livestock	349.82	1096.24	283.01	197.54	160.26
Oil crops	65.93	226.92	63.76	66.71	85.77
Pulses	3.52	12.96	3.84	3.3	3.47
Roots & tubers	19.55	65.78	18.13	15.8	17.18
Sugar crops	46.53	160.66	46.2	44.31	53.72
Vegetables	38.99	132.03	36.63	30.77	32.03
**Mozambique**					
Cereals	22.98	65.32	8.97	13.9	0
Fiber	18.22	28.28	2.45	101.72	0
Fruit	2.96	7.17	0.91	2.03	0
Livestock	23.12	81.7	12.11	0.07	0
Oil crops	62.57	130.2	15.03	45.44	0
Pulses	70.8	118.08	11.62	8.07	0
Roots & tubers	216.93	529.05	66.14	25.72	0
Sugar crops	4.25	14.77	2.2	0.01	0
Vegetables	5.6	10.31	1.1	2.98	0
**Myanmar**					
Cereals	2885.11	5264.61	289.27	111.29	0
Fiber	63.42	107.08	5.15	12.35	0
Fruit	4.7	7.9	0.37	0.13	0
Livestock	562.48	903.12	40.04	19.26	0
Oil crops	2547.55	4334.11	204.38	73.03	0
Pulses	1145.69	1921.36	90.64	32.61	0
Roots & tubers	30.55	51.98	2.46	0.88	0
Sugar crops	31.22	48.61	2.08	0.72	0
Vegetables	67.26	112.92	5.33	1.92	0
**Namibia**					
Cereals	2.43	7.98	1.18	1.39	0
Fiber	0.02	0.06	0.01	0.03	0
Fruit	0.26	1.01	0.16	0.55	0
Livestock	41.03	69.86	6.76	22.13	0

Oil crops	0.01	0.02	0	0.01	0
Pulses	0.71	2.55	0.39	1.36	0
Roots & tubers	0	0	0	0	0
Sugar crops	0	0	0	0	0
Vegetables	0.09	0.35	0.05	0.19	0
**Nepal**					
Cereals	568.81	1036.32	116	22.5	0
Fiber	0.01	0.02	0	0	0
Fruit	4.85	8.71	0.98	0.19	0
Livestock	577.87	1022.85	112.03	21.91	0
Oil crops	17.82	34.18	3.92	0.76	0
Pulses	13.66	23.91	2.62	0.51	0
Roots & tubers	55.36	100.88	11.27	2.19	0
Sugar crops	7.93	14.85	1.68	0.33	0
Vegetables	0	0	0	0	0
**Netherlands**					
Cereals	12.49	73.9	48.69	243.68	113.53
Fiber	0	0	0	0	0
Fruit	0.62	3.5	2.22	9.62	4.19
Livestock	333.73	2932.08	2363.65	6494.65	1679.28
Oil crops	0.03	2.07	2.51	16.32	7.82
Pulses	0.18	1.05	0.7	2.98	1.28
Roots & tubers	9.64	71.5	55.39	311.42	148.65
Sugar crops	18.77	107.31	69.27	339.93	156.97
Vegetables	8.45	47.73	30.2	131.92	57.64
**New Zealand**					
Cereals	0.16	4.8	7.27	36.64	186.66
Fiber	0	0	0	0	0
Fruit	0.03	0.87	1.31	6.62	33.73
Livestock	23.03	640.57	916.94	3867.73	12745.98
Oil crops	0.01	0.17	0.25	1.27	6.47
Pulses	0.01	0.23	0.35	1.77	9
Roots & tubers	0.03	0.98	1.48	7.47	38.04
Sugar crops	0	0	0	0	0
Vegetables	0.01	0.18	0.27	1.38	7.02
**Nicaragua**					
Cereals	17.96	36.97	26.63	17.48	21.11
Fiber	0.47	1.01	0.81	0.49	0.53
Fruit	3.94	8.49	6.98	4.19	4.51
Livestock	101.29	225.16	205.78	127.84	144.31
Oil crops	9.37	19.97	15.96	9.61	10.4
Pulses	53.78	100.44	41.58	22.29	19.96
Roots & tubers	2.62	5.57	4.42	2.65	2.84
Sugar crops	6.11	13.04	10.45	6.28	6.79
Vegetables	0.29	0.62	0.51	0.3	0.32
**Niger**					

Cereals	133.91	184.55	13.84	39.62	0
Fiber	4.56	6.05	0.43	0	0
Fruit	1.99	2.89	0.23	0.97	0
Livestock	414.94	564.08	41.38	58.76	0
Oil crops	160.26	220.22	16.44	35.13	0
Pulses	6.56	8.86	0.64	1.23	0
Roots & tubers	14.58	19.81	1.45	3.62	0
Sugar crops	1.02	1.25	0.07	0	0
Vegetables	29.59	40.38	2.97	7.66	0
**Nigeria**					
Cereals	1063.01	1839.58	180.79	79.41	0
Fiber	165.43	312.69	33.41	0.15	0
Fruit	32.75	64.38	7.11	9.48	0
Livestock	279.41	448.43	42.16	150.91	0
Oil crops	843.86	1557.43	162.61	100.45	0
Pulses	7.29	12.66	1.25	0.01	0
Roots & tubers	3202.04	6564.99	743.36	1031.93	0
Sugar crops	5	6.03	0.32	0	0
Vegetables	146.69	233.74	21.11	23.2	0
**Norway**					
Cereals	18.89	139.49	99.02	184.8	18.55
Fiber	0	0	0	0	0
Fruit	0.03	0.2	0.14	0.27	0.03
Livestock	40.47	454.37	411.36	926.63	124.27
Oil crops	1.16	8.55	6.05	11.25	1.12
Pulses	0	0	0	0	0
Roots & tubers	1.26	9.3	6.61	12.34	1.24
Sugar crops	0	0	0	0	0
Vegetables	0.05	0.38	0.27	0.5	0.05
**Oman**					
Cereals	0.25	1.17	0.43	0.21	0.04
Fiber	0	0	0	0	0
Fruit	18.08	83.61	31.01	15.04	2.67
Livestock	24.85	114.94	42.63	20.68	3.67
Oil crops	0	0	0	0	0
Pulses	0	0	0	0	0
Roots & tubers	0.07	0.34	0.13	0.06	0.01
Sugar crops	0	0	0	0	0
Vegetables	0.6	2.76	1.02	0.5	0.09
**Pakistan**					
Cereals	2696.26	6380.11	808.3	156.1	0
Fiber	1545.86	4018.29	525.07	101.84	0
Fruit	202.01	481.53	61.62	11.77	0
Livestock	12609.76	22065.85	2433.22	465.56	0
Oil crops	399.46	918.72	115.74	22.16	0
Pulses	71.67	161.6	20.14	3.87	0

Roots & tubers	54.04	116.48	14.18	2.75	0
Sugar crops	132.86	319.5	40.87	7.85	0
Vegetables	111.38	277.21	36.03	6.88	0
**Panama**					
Cereals	12.8	26.51	18.1	8.35	5.16
Fiber	0	0	0	0	0
Fruit	3.79	9.84	11.93	5.65	3.8
Livestock	23.22	65.26	88.64	40.64	24.68
Oil crops	0.34	0.89	1.12	0.55	0.42
Pulses	0.69	1.72	1.98	1.09	1.03
Roots & tubers	1.16	2.83	3.03	1.4	0.88
Sugar crops	1.37	4.48	7.21	3.08	1.41
Vegetables	0.84	2.15	2.52	1.18	0.76
**Papua New Guinea**					
Cereals	0.39	0.51	0.19	0.1	0
Fiber	0	0	0	0	0
Fruit	9.25	13.52	5.21	2.83	0
Livestock	5.11	3.84	0.95	0.42	0
Oil crops	19.85	29.03	11.19	6.08	0
Pulses	0.24	0.35	0.14	0.07	0
Roots & tubers	55.27	78.48	29.93	16.19	0
Sugar crops	1.01	1.47	0.57	0.31	0
Vegetables	0.01	0.02	0.01	0	0
**Paraguay**					
Cereals	0.63	11.64	14.26	49.41	284.84
Fiber	1.12	18.03	17.97	32.64	91.24
Fruit	0.2	3.72	4.57	14.48	69.09
Livestock	4.03	64.03	61.93	105.89	330.27
Oil crops	9.04	217.85	346.18	1711.53	11135.47
Pulses	0.86	13.53	12.93	19.86	42.12
Roots & tubers	1.35	25.02	30.6	100.03	516.65
Sugar crops	0.18	3.22	3.75	9.01	20.91
Vegetables	0.05	0.96	1.09	2.69	8.64
**Peru**					
Cereals	11.62	180.14	166.07	214.05	282.11
Fiber	2.95	45.72	42.12	53.89	67.38
Fruit	2.14	33.92	32.78	48.65	58.26
Livestock	28.08	432	392.16	474.91	587.15
Oil crops	0.67	10.3	9.48	12.09	15.02
Pulses	2.06	31.59	28.35	31.64	22.81
Roots & tubers	6.05	94.46	88.45	121.38	176.27
Sugar crops	1	15.47	14.32	18.73	24.76
Vegetables	1.99	30.66	27.91	33.92	38.35
**Philippines**					
Cereals	951.4	2163.58	155.9	1316.71	0
Fiber	0.68	1.53	0.11	0.74	0

Fruit	68.51	176.84	13.84	88.84	0
Livestock	130.07	276.01	16.85	18.07	0
Oil crops	213.63	551.51	43.18	277.33	0
Pulses	8.4	21.67	1.7	10.89	0
Roots & tubers	79.37	217.81	17.51	58.55	0
Sugar crops	77.49	202.18	15.11	21.72	0
Vegetables	7.34	18.96	1.48	9.52	0
**Poland**					
Cereals	353.6	1414.11	646.19	2171.97	831.52
Fiber	0	0	0	0	0
Fruit	8.34	31.16	12.59	34.12	11.35
Livestock	1320.97	4903.09	1962.56	5084.64	1608.9
Oil crops	97.41	645.71	483.11	2481.42	1126.1
Pulses	7.99	29.85	12.03	32.11	10.54
Roots & tubers	110.55	375.62	123.47	254.51	70.11
Sugar crops	65.74	308.27	173.45	667.19	266.58
Vegetables	17.85	65.09	25.25	69.07	23.56
**Portugal**					
Cereals	7.81	33.66	17.26	80.79	37.03
Fiber	0	0	0	0	0
Fruit	18.07	60.73	19.62	51.29	18.67
Livestock	340.37	1050.19	279.87	700.91	263.67
Oil crops	10.08	38.96	16.9	83.53	40.67
Pulses	0.3	1.13	0.47	1.96	0.89
Roots & tubers	7.69	26.98	9.09	13.69	2
Sugar crops	0.7	6.52	5.71	25.29	10.24
Vegetables	7.83	29.45	12.22	50.9	23.03
**Qatar**					
Cereals	0	0	0	0	0
Fiber	0	0	0	0	0
Fruit	0	0	0	0	0
Livestock	3.33	15.4	5.71	2.77	0.49
Oil crops	0	0	0	0	0
Pulses	0	0	0	0	0
Roots & tubers	0	0	0	0	0
Sugar crops	0	0	0	0	0
Vegetables	0	0	0	0	0
**Republic of Korea**					
Cereals	756.25	1040.41	32.11	8.96	0
Fiber	0	0	0	0	0
Fruit	22.73	31.2	0.96	0.27	0
Livestock	1282.29	1709.6	49.59	13.31	0
Oil crops	253.27	339.57	9.97	2.69	0
Pulses	4.56	6.26	0.19	0.05	0
Roots & tubers	53.82	72.7	2.17	0.59	0
Sugar crops	0	0	0	0	0

Vegetables	105.78	145.23	4.47	1.25	0
**Romania**					
Cereals	123.01	557.57	309.43	1168.62	454.88
Fiber	0	0	0	0	0
Fruit	3.31	16.82	10.77	50.93	22.17
Livestock	460.6	1936.65	960.68	3283.45	1231.14
Oil crops	58.9	304.04	195.93	899.53	385.54
Pulses	3.88	15.05	6.54	19.31	6.73
Roots & tubers	28.18	113.73	51.12	119.57	31.41
Sugar crops	8.65	32.38	13.1	39.39	14.51
Vegetables	7.66	32.21	16.28	59.19	22.91
**Russian Federation**					
Cereals	1137.85	4833.5	2433.95	11622.85	5397.19
Fiber	0	0	0	0	0
Fruit	3.02	16.56	10.91	67.76	33.96
Livestock	1310.35	7039.27	4451.64	18624.07	7753.59
Oil crops	224.64	1037.14	575.76	2823.78	1308.67
Pulses	21.49	91.65	45.15	177.97	75.6
Roots & tubers	235.56	924.96	404.55	1312.37	501.63
Sugar crops	158.63	599.36	259.53	1523.49	782.52
Vegetables	28	116.08	55.69	231.59	101.57
**Rwanda**					
Cereals	20.97	31.31	2.64	0.01	0
Fiber	0	0	0	0	0
Fruit	19.03	31.01	2.93	0.01	0
Livestock	90.29	124.03	9.12	0.02	0
Oil crops	35.52	45.3	2.89	0.01	0
Pulses	129.49	202.98	18.29	0.06	0
Roots & tubers	153.04	222.18	17.75	0.05	0
Sugar crops	0.25	0.42	0.04	0	0
Vegetables	2.05	2.46	0.12	0	0
**Saint Lucia**					
Cereals	0	0	0	0	0
Fiber	0	0	0	0	0
Fruit	0	0	0	0	0
Livestock	0	0	0	0	0
Oil crops	0	0	0	0	0
Pulses	0	0	0	0	0
Roots & tubers	0	0	0	0	0
Sugar crops	0	0	0	0	0
Vegetables	0	0	0	0	0
**Saint Vincent and the Grenadines**					
Cereals	0	0	0	0	0
Fiber	0	0	0	0	0
Fruit	0	0	0	0	0

Livestock	0	0	0	0	0
Oil crops	0	0	0	0	0
Pulses	0	0	0	0	0
Roots & tubers	0	0	0	0	0
Sugar crops	0	0	0	0	0
Vegetables	0	0	0	0	0
**Saudi Arabia**					
Cereals	137.72	529.68	170.95	83.11	14.46
Fiber	0	0	0	0	0
Fruit	94.1	336.31	100.25	49.19	8.43
Livestock	324.33	972.02	241.74	115.56	19.45
Oil crops	14.53	30.78	3.77	1.49	0.22
Pulses	0.35	1.39	0.46	0.22	0.04
Roots & tubers	6.26	23.3	7.43	3.56	0.62
Sugar crops	0	0	0	0	0
Vegetables	10.51	32.87	8.6	4.14	0.7
**Senegal**					
Cereals	35.72	81.55	10.09	0.05	0
Fiber	9.93	32.68	4.76	0.03	0
Fruit	4.68	6.79	0.57	0	0
Livestock	65.55	107.71	10.22	0.04	0
Oil crops	87.23	106.36	7.18	0.01	0
Pulses	0.04	0.05	0	0	0
Roots & tubers	17.32	21.16	1.3	0	0
Sugar crops	1.57	5.85	0.88	0	0
Vegetables	9.22	17.89	1.98	0.01	0
**Serbia**					
Cereals	91.5	334.6	130.18	336.65	108.15
Fiber	0	0	0	0	0
Fruit	5.76	18.71	5.66	12.99	4.21
Oil crops	92.36	360.34	157.85	449.85	150.55
Pulses	6.87	22.97	7.49	17.97	5.8
Roots & tubers	6.16	23.07	9.41	27.15	9.52
Sugar crops	50.48	159.18	44.67	103.81	35.81
Vegetables	4.42	15.41	5.45	12.86	3.92
**Sierra Leone**					
Cereals	66.73	146.48	17.56	0.08	0
Fiber	0	0	0	0	0
Fruit	0.16	0.5	0.07	0	0
Livestock	8.56	13.7	1.29	0	0
Oil crops	7.2	15.8	1.91	0.01	0
Pulses	4.57	13.64	1.92	0.01	0
Roots & tubers	60.4	144.4	18.11	0.09	0
Sugar crops	0.25	0.41	0.04	0	0
Vegetables	0.53	1.15	0.14	0	0
**Slovakia**					

Cereals	58.41	224.97	98.49	300.43	105.63
Fiber	0	0	0	0	0
Fruit	0.86	2.73	0.77	1.74	0.59
Livestock	37.52	256.97	186.71	660.76	238.44
Oil crops	68.36	255.89	108.31	329.78	115.75
Pulses	0.76	2.9	1.25	3.86	1.37
Roots & tubers	1.62	7.07	3.62	10.14	3.1
Sugar crops	10.08	42.9	21.79	65.86	21.58
Vegetables	0.84	3.16	1.33	4	1.41
**Slovenia**					
Cereals	13.23	44.37	13.12	14.6	0.68
Fiber	0	0	0	0	0
Fruit	2.47	8.63	2.78	3.25	0.15
Livestock	138.62	436.3	110.8	110.15	5.12
Oil crops	3.98	13.35	3.95	4.39	0.2
Pulses	0.49	1.66	0.52	0.59	0.03
Roots & tubers	1.66	5.77	1.83	2.12	0.1
Sugar crops	3.31	11.07	3.27	3.63	0.17
Vegetables	0.24	0.84	0.26	0.3	0.01
**Somalia**					
Cereals	10.58	15.78	1.27	0	0
Fiber	2.48	3.8	0.32	0	0
Fruit	4.14	6.32	0.53	0	0
Livestock	649.2	1290.14	144.35	0.66	0
Oil crops	184.91	282.61	23.58	0.08	0
Pulses	8.5	14.2	1.34	0.01	0
Roots & tubers	4.2	6.41	0.54	0	0
Sugar crops	0.69	1.05	0.09	0	0
Vegetables	0.65	0.99	0.08	0	0
**South Africa**					
Cereals	93.09	388.86	61.09	653.21	0
Fiber	3.68	20.06	3.33	11.21	0
Fruit	139.15	331.93	42.19	125.44	0
Livestock	721.42	1726.63	217.89	1233.13	0
Oil crops	133.43	465.47	68.95	366.71	0
Pulses	3.45	29.48	5.2	37.04	0
Roots & tubers	20.19	86.14	13.53	59.17	0
Sugar crops	11.42	113.02	20.23	59.55	0
Vegetables	15.15	60.82	9.42	41.35	0
**Spain**					
Cereals	286.48	1296.37	690.07	2329.04	859.51
Fiber	3.47	20.83	15.05	109.08	56.92
Fruit	111.19	424.33	177.46	556.46	209.97
Livestock	551.35	2702.78	1500.14	3907.37	1108.77
Oil crops	116.08	562.2	335.39	2004.43	996.63
Pulses	4.97	20.05	9.25	33.57	13.65

Roots & tubers	15.15	66.67	32.71	88.85	28.44
Sugar crops	18.51	116.65	83.13	367.32	154.94
Vegetables	37.29	150.58	69.43	252.17	102.52
**Sri Lanka**					
Cereals	243.99	461.43	52.87	151.55	0
Fiber	0	0	0	0	0
Fruit	3.54	7.81	0.97	3.39	0
Livestock	72.32	120.12	12.83	34.51	0
Oil crops	56.07	109.18	12.74	40.84	0
Pulses	2.25	5.09	0.63	2.82	0
Roots & tubers	10.74	23.29	2.84	9.67	0
Sugar crops	1.85	4.35	0.55	2.96	0
Vegetables	7.19	15.99	1.98	7.5	0
**Sudan (former)**					
Cereals	61.76	348.56	129.67	154.21	209.1
Fiber	29.29	111.04	33.37	40.15	56.15
Fruit	31.38	111.64	31.85	38.6	54.47
Livestock	1264.59	4618.51	1359.7	1182.65	1260.64
Oil crops	459	1376.43	329.86	359.4	480.17
Pulses	4.96	18.26	5.36	6.04	8.08
Roots & tubers	10.64	37.1	10.42	12.17	16.8
Sugar crops	8.89	31.26	8.83	9.91	13.29
Vegetables	36.12	126.95	35.85	40.25	53.99
**Suriname**					
Cereals	0	0.35	0.88	6.11	41.72
Fiber	0	0	0	0	0
Fruit	0	0.05	0.11	0.78	5.32
Livestock	0	0.08	0.19	1.32	9.01
Oil crops	0	0	0.01	0.06	0.39
Pulses	0	0	0	0	0.02
Roots & tubers	0	0.01	0.01	0.09	0.6
Sugar crops	0	0.01	0.02	0.13	0.88
Vegetables	0	0	0	0.01	0.06
**Swaziland**					
Cereals	0.8	1.68	0.2	0.06	0
Fiber	0.54	1.57	0.22	0.17	0
Fruit	4.36	9.63	1.16	0.29	0
Livestock	12.01	25.7	3	7.58	0
Oil crops	0	0	0	0	0
Pulses	0.38	1.02	0.14	0.03	0
Roots & tubers	0.34	0.67	0.07	0.02	0
Sugar crops	14.31	31.43	3.76	1.16	0
Vegetables	0.09	0.2	0.02	0.01	0
**Sweden**					
Cereals	42.34	344.83	263.16	755.13	219.58
Fiber	0	0	0	0	0

Fruit	0.03	0.23	0.19	0.54	0.15
Livestock	106.41	835.99	622.23	1753.04	507.19
Oil crops	17.82	134.84	97.56	266.21	75.41
Pulses	0.46	4.14	3.37	9.57	2.61
Roots & tubers	2.85	20.28	13.97	31.58	6.74
Sugar crops	11.57	80.02	53.72	110.67	19.49
Vegetables	0.18	1.6	1.3	3.7	1.01
**Switzerland**					
Cereals	14.73	79.96	52.25	112.15	15.28
Fiber	0	0	0	0	0
Fruit	1.16	7.2	5.1	10.95	1.45
Livestock	148.12	1277.37	1013.24	2084.51	247.44
Oil crops	15.51	63.25	30.77	60.96	8.26
Pulses	0.34	1.91	1.29	2.81	0.38
Roots & tubers	0.94	9.85	8.64	19.08	2.49
Sugar crops	3.69	40.63	36.49	81.79	10.9
Vegetables	0.13	0.73	0.49	1.07	0.15
**Syrian Arab Republic**					
Cereals	355.22	1011.42	239.29	148.41	103.64
Fiber	119.7	401.49	114.65	87.21	83.35
Fruit	30.14	102.26	29.5	22.68	21.94
Livestock	505.74	1759.32	516.78	390.82	365.38
Oil crops	93.19	299.48	81.81	61.89	59.12
Pulses	1.05	3.48	0.98	0.75	0.73
Roots & tubers	8.51	27.1	7.32	5.45	5.08
Sugar crops	29.3	88.24	22.59	12.92	7.13
Vegetables	17.8	60.66	17.62	12.84	11.51
**Tajikistan**					
Cereals	27.29	121.14	40.5	35.36	36.81
Fiber	45.66	200.94	66.86	59.14	62.63
Fruit	1.77	7.74	2.57	2.27	2.4
Livestock	59.1	277.06	94.53	110.18	148.31
Oil crops	0.56	2.55	0.88	0.69	0.63
Pulses	1.04	5.29	1.93	1.38	1.06
Roots & tubers	5.57	24.85	8.32	7.32	7.67
Sugar crops	0	0	0	0	0
Vegetables	6.57	28.78	9.55	8.43	8.9
**Thailand**					
Cereals	2183.9	5963.31	482.27	207.54	0
Fiber	2.57	7.72	0.67	0.29	0
Fruit	98.29	249.15	19.43	8.31	0
Livestock	336.58	1073.12	95.71	42.11	0
Oil crops	270.84	781.07	66.49	29.04	0
Pulses	52.54	104.74	6.46	2.59	0
Roots & tubers	569.81	2121.63	204.38	91.39	0
Sugar crops	151.54	351.07	25.42	10.68	0

Vegetables	6.19	15.7	1.22	0.52	0
**The former Yugoslav Republic of Macedonia**					
Cereals	9.01	47.6	29.27	58.53	7.25
Fiber	0	0	0	0	0
Fruit	1.85	9.4	5.61	11.13	1.38
Livestock	45.08	176.69	76.53	131.87	14.94
Oil crops	0.83	4.14	2.41	4.71	0.57
Pulses	0.58	2.94	1.75	3.44	0.42
Roots & tubers	1.02	5.45	3.28	6.22	0.7
Sugar crops	0.21	1.14	0.72	1.46	0.18
Vegetables	1.18	6.02	3.58	7.09	0.87
**Togo**					
Cereals	27.44	45.5	4.27	0.02	0
Fiber	22.56	27.28	1.55	0	0
Fruit	1.46	2.66	0.28	0	0
Livestock	8.7	12.97	1.08	0	0
Oil crops	4.71	9.28	1.04	0	0
Pulses	25.22	48.13	5.26	0.02	0
Roots & tubers	63.52	113.71	11.63	0.05	0
Sugar crops	0	0	0	0	0
Vegetables	0.21	0.31	0.03	0	0
**Trinidad and Tobago**					
Cereals	0	0.05	0.19	0.18	0.3
Fiber	0	0	0	0	0
Fruit	0	0.12	0.49	0.47	0.76
Livestock	0	0	0	0	0
Oil crops	0	0.09	0.35	0.33	0.54
Pulses	0	0	0.02	0.02	0.03
Roots & tubers	0	0.02	0.07	0.06	0.1
Sugar crops	0	0.46	1.85	1.76	2.89
Vegetables	0	0.01	0.06	0.05	0.09
**Tunisia**					
Cereals	70.56	287.18	91.38	91.35	110.8
Fiber	0.22	0.95	0.32	0.33	0.42
Fruit	20.96	63.09	15.73	12.57	12.84
Livestock	132.93	631.63	218.65	154.19	112.51
Oil crops	67.6	332.65	116.57	118.09	141.75
Pulses	0.84	3.69	1.23	1.3	1.65
Roots & tubers	3.68	12.31	3.3	4.55	6.92
Sugar crops	0	0	0	0	0
Vegetables	7.41	34.43	11.89	21.95	37.51
**Turkey**					
Cereals	1514.3	4916.6	1310.91	1131.28	1226.41
Fiber	316.82	1177.61	363.56	266.77	233.57
Fruit	158.77	474.84	114.26	97.31	104.53

Livestock	2186.69	7791.36	2239.65	1575.42	1218.02
Oil crops	272.97	892.72	239.05	176.82	154.78
Pulses	41.02	131.99	34.82	22.21	14.53
Roots & tubers	86.77	212.44	37.9	24.72	20.17
Sugar crops	359.88	962.3	198.46	123.45	82.68
Vegetables	221.39	673.62	166.5	124.37	112.52
**Turkmenistan**					
Cereals	21.65	361.99	170.12	210.98	289
Fiber	18.17	301.32	141.5	177.41	244.89
Fruit	0.68	11.27	5.29	6.63	9.15
Livestock	46.15	689.76	321.04	423.21	604.61
Oil crops	0	0	0	0	0
Pulses	0.09	1.44	0.68	0.85	1.17
Roots & tubers	0.32	6.96	3.33	3.61	4.43
Sugar crops	0.62	9.86	4.61	5.74	7.88
Vegetables	0.97	16.09	7.55	9.47	13.07
**Uganda**					
Cereals	77.15	131.52	12.91	0.05	0
Fiber	19.08	37.69	4.21	0.02	0
Fruit	63.23	119.69	12.94	0.06	0
Livestock	307.06	872.75	119.13	0.64	0
Oil crops	888.35	1177.29	85.64	0.18	0
Pulses	192.31	343.9	35.66	0.14	0
Roots & tubers	412.8	814.06	89.67	0.41	0
Sugar crops	7.65	14.59	1.59	0.01	0
Vegetables	13.66	26.07	2.84	0.01	0
**Ukraine**					
Cereals	666.38	2590.35	1129.83	3456.2	1241.94
Fiber	0	0	0	0	0
Fruit	4.92	19.34	8.57	28.03	10.64
Livestock	1251.76	4837.82	2075.34	5997.74	2067.83
Oil crops	412.84	1623.89	716.59	2252.99	831.54
Pulses	10.8	51.55	29.57	104.8	39
Roots & tubers	112.05	489.34	247.89	683.06	208.58
Sugar crops	114.03	516.95	275.04	972.85	374.34
Vegetables	21.33	82.11	35.32	117.58	45.61
**United Arab Emirates**					
Cereals	0	0.01	0	0	0
Fiber	0	0	0	0	0
Fruit	53.61	247.97	91.98	44.62	7.91
Livestock	1.4	6.5	2.41	1.17	0.21
Oil crops	0	0	0	0	0
Pulses	0	0	0	0	0
Roots & tubers	0.08	0.35	0.13	0.06	0.01
Sugar crops	0	0	0	0	0
Vegetables	1.22	5.64	2.09	1.02	0.18

**United Kingdom**					
Cereals	344.16	1702.21	979.5	2962.75	969.26
Fiber	0	0	0	0	0
Fruit	0.3	2.53	1.98	6.38	2.1
Livestock	726.74	4890.12	3224.51	7164.45	1559.36
Oil crops	283.66	1378.99	783.66	2285.23	716.27
Pulses	4.64	39.21	30.67	98.69	32.53
Roots & tubers	9.97	91.84	75.59	258.57	88.37
Sugar crops	16.27	152.19	126.35	469.42	172.9
Vegetables	1.85	15.6	12.2	39.25	12.94
**United Republic of Tanzania**					
Cereals	195.6	429.39	51.15	0.25	0
Fiber	55.18	153.39	20.82	0.11	0
Fruit	30.8	102.93	14.92	0.08	0
Livestock	600.74	1179.99	128.16	0.58	0
Oil crops	277.81	762.02	102.67	0.54	0
Pulses	314.37	596.8	63.51	0.28	0
Roots & tubers	260.31	750.17	103.55	0.55	0
Sugar crops	5.27	16.45	2.34	0.01	0
Vegetables	4.85	14.19	1.96	0.01	0
**United States of America**					
Cereals	3.67	1684.6	3469.68	16273.88	16708.33
Fiber	0.58	411.54	974.55	4940.39	5236.18
Fruit	1.19	264.63	386.05	1201.5	1363.18
Livestock	18.13	6842.56	12651.95	46950.74	37255.05
Oil crops	13.79	8864.11	20464.64	98445.59	99401.73
Pulses	0.15	76.52	163.8	746.28	722.61
Roots & tubers	0.44	164.82	288.39	935.9	556.14
Sugar crops	0.09	103.83	283.54	1698.15	2030.13
Vegetables	0.24	108.56	214.67	848.13	695.53
**Uruguay**					
Cereals	0.65	15.37	24.24	104.8	545.88
Fiber	0	0	0	0	0
Fruit	0.11	2.49	3.91	14.22	44.58
Livestock	2.31	55.64	88.74	360.6	1568.66
Oil crops	0.66	17.08	28.36	188.83	1655.54
Pulses	0.01	0.18	0.29	1.08	3.81
Roots & tubers	0.03	0.74	1.22	5.01	20.42
Sugar crops	0	0	0	0.12	1.91
Vegetables	0.01	0.27	0.44	1.85	7.77
**Uzbekistan**					
Cereals	158.5	945.73	367.25	339.36	374.35
Fiber	267.1	1593.49	618.76	571.71	630.58
Fruit	7.27	43.27	16.79	15.54	17.16
Livestock	362.97	2839.76	1188.83	862.7	644.89
Oil crops	3.37	20.09	7.8	7.11	7.73

Pulses	0.2	1.12	0.43	0.38	0.4
Roots & tubers	6.86	40.63	15.74	14.59	16.17
Sugar crops	0	0	0	0	0
Vegetables	18.85	112.02	43.45	40.2	44.44
**Venezuela (Bolivarian Republic of)**					
Cereals	1.37	24.91	29.71	83.9	325.16
Fiber	0.11	1.83	2	4.7	16.03
Fruit	0.71	12.23	13.45	31.56	103.22
Livestock	7.02	121.95	136.39	352.28	1415.54
Oil crops	1.72	29.3	31.98	74.83	257.73
Pulses	0.28	4.65	4.78	9.19	23.4
Roots & tubers	0.49	8.32	9.15	22.1	80.34
Sugar crops	0.43	7.3	7.99	18.75	63.7
Vegetables	0.31	5.36	5.87	13.78	46.98
**Viet Nam**					
Cereals	1366.17	5350.96	522.36	2989.49	0
Fiber	6.42	14.72	1.06	3.86	0
Fruit	69.28	158.96	11.43	36.8	0
Livestock	221.52	350.6	14.94	116.22	0
Oil crops	270.69	646.68	49.31	101.44	0
Pulses	67.8	146.32	10.17	13.86	0
Roots & tubers	442.61	801.83	44.54	73.56	0
Sugar crops	31.09	90.68	7.7	34.07	0
Vegetables	13.6	31.75	2.32	6.89	0
**Yemen**					
Cereals	16.58	75.95	25.95	13.4	2.29
Fiber	3.19	12.55	3.9	1.99	0.34
Fruit	8.52	40.92	14.26	7.42	1.26
Livestock	114.51	261.81	40.82	17.38	2.72
Oil crops	33.52	120.67	35.01	17.66	2.98
Pulses	1.08	4.27	1.33	0.68	0.11
Roots & tubers	2.77	11.87	3.88	2	0.34
Sugar crops	0	0	0	0	0
Vegetables	6.08	31.96	11.67	6.09	1.04
**Zambia**					
Cereals	25.24	60.08	7.67	12.61	0
Fiber	16.37	44.26	6.05	66.85	0
Fruit	0.33	0.54	0.05	0.1	0
Livestock	42.01	65.98	5.91	15.55	0
Oil crops	35.84	121.92	18	15.87	0
Pulses	2.09	3.48	0.34	0.8	0
Roots & tubers	61.68	87.98	6.65	4.79	0
Sugar crops	7.52	12.36	1.18	2.88	0
Vegetables	2.47	4.06	0.39	0.95	0
**Zimbabwe**					

Cereals	27.3	103.61	15.8	5.41	0
Fiber	24.93	149.27	25.07	13.8	0
Fruit	2.25	24.95	4.5	0.33	0
Livestock	168.7	293.58	29.89	0.23	0
Oil crops	62.59	143.03	17.89	7.64	0
Pulses	6.61	24.61	3.74	0.78	0
Roots & tubers	9.88	18.58	2.04	1.42	0
Sugar crops	8.66	20.45	2.6	1.98	0
Vegetables	0.03	1.86	0.36	0.01	0

	Folate production (T)				
	**< 2 ha**	**2 - 20 ha**	**20 - 50 ha**	**50 - 200 ha**	**> 200 ha**
**Afghanistan**					
Cereals	0.4	0.98	0.13	0.42	0
Fiber	0	0.06	0.01	0	0
Fruit	0	0.01	0	0	0
Livestock	0.04	0.06	0.01	0	0
Oil crops	0.02	0.03	0	0	0
Pulses	0.01	0.01	0	0	0
Roots & tubers	0.01	0.02	0	0	0
Sugar crops	0	0	0	0	0
Vegetables	0	0	0	0	0
**Albania**					
Cereals	0.03	0.09	0.02	0.02	0
Fiber	0	0	0	0	0
Fruit	0	0	0	0	0
Livestock	0.01	0.04	0.01	0.01	0
Oil crops	0	0	0	0	0
Pulses	0.02	0.05	0.01	0.01	0
Roots & tubers	0	0.01	0	0	0
Sugar crops	0.01	0.02	0	0	0
Vegetables	0.01	0.02	0	0	0
**Algeria**					
Cereals	0.15	0.56	0.17	0.19	0.26
Fiber	0	0	0	0	0
Fruit	0.02	0.1	0.03	0.02	0.01
Livestock	0.02	0.1	0.03	0.03	0.02
Oil crops	0.01	0.04	0.01	0.02	0.02
Pulses	0	0.01	0	0	0
Roots & tubers	0.03	0.11	0.03	0.03	0.04
Sugar crops	0	0	0	0	0
Vegetables	0.03	0.12	0.04	0.03	0.02
**Angola**					
Cereals	0.07	0.14	0.02	0.01	0
Fiber	0	0.01	0	0	0

Fruit	0.05	0.09	0.01	0.01	0
Livestock	0.01	0.01	0	0	0
Oil crops	0.01	0.02	0	0	0
Pulses	0.1	0.23	0.03	0.03	0
Roots & tubers	0.56	1.34	0.17	0.12	0
Sugar crops	0	0	0	0	0
Vegetables	0	0	0	0	0
**Argentina**					
Cereals	0.01	0.14	0.23	1.28	9.34
Fiber	0	0.05	0.06	0.15	0.83
Fruit	0	0.01	0.01	0.04	0.26
Livestock	0	0.06	0.06	0.14	0.6
Oil crops	0.06	1.71	2.92	17.77	139.94
Pulses	0	0.04	0.06	0.23	0.88
Roots & tubers	0	0.02	0.02	0.07	0.19
Sugar crops	0	0	0	0	0
Vegetables	0	0	0.01	0.03	0.18
**Armenia**					
Cereals	0.01	0.07	0.03	0.01	0
Fiber	0	0	0	0	0
Fruit	0	0	0	0	0
Livestock	0	0.03	0.01	0	0
Oil crops	0	0	0	0	0
Pulses	0	0.01	0	0	0
Roots & tubers	0.01	0.04	0.02	0.01	0
Sugar crops	0	0.01	0	0	0
Vegetables	0	0.03	0.01	0.01	0
**Australia**					
Cereals	0	0.02	0.07	0.68	8.89
Fiber	0	0	0.02	0.15	2.69
Fruit	0	0	0	0.01	0.18
Livestock	0	0	0.01	0.06	0.7
Oil crops	0	0.01	0.04	0.37	3.86
Pulses	0	0	0.01	0.05	1
Roots & tubers	0	0	0	0.02	0.14
Sugar crops	0	0	0	0	0
Vegetables	0	0	0	0.01	0.08
**Austria**					
Cereals	0.12	0.47	0.22	0.39	0.05
Fiber	0	0	0	0	0
Fruit	0	0.01	0	0.01	0
Livestock	0.01	0.07	0.05	0.09	0.01
Oil crops	0.08	0.32	0.13	0.23	0.03
Pulses	0.02	0.08	0.04	0.07	0.01
Roots & tubers	0.01	0.04	0.01	0.02	0
Sugar crops	0.27	0.98	0.37	0.61	0.07

Vegetables	0	0.01	0	0.01	0
**Azerbaijan**					
Cereals	0.11	0.36	0.1	0.08	0.08
Fiber	0.05	0.16	0.04	0.04	0.04
Fruit	0	0	0	0	0
Livestock	0.01	0.05	0.01	0.01	0.01
Oil crops	0	0.01	0	0	0
Pulses	0.01	0.03	0.01	0.01	0
Roots & tubers	0.02	0.06	0.02	0.01	0.01
Sugar crops	0.01	0.05	0.01	0.01	0.01
Vegetables	0.02	0.05	0.01	0.01	0.01
**Bangladesh**					
Cereals	0.96	2.07	0.25	0.5	0
Fiber	0.03	0.07	0.01	0.01	0
Fruit	0.04	0.08	0.01	0.02	0
Livestock	0.08	0.15	0.02	0.02	0
Oil crops	0.19	0.56	0.08	0.12	0
Pulses	0.06	0.14	0.02	0.04	0
Roots & tubers	0.16	0.33	0.04	0.12	0
Sugar crops	0	0	0	0	0
Vegetables	0.04	0.08	0.01	0.02	0
**Belarus**					
Cereals	0.14	0.52	0.22	0.63	0.22
Fiber	0	0	0	0	0
Fruit	0	0	0	0	0
Livestock	0.03	0.1	0.05	0.16	0.06
Oil crops	0.05	0.2	0.08	0.24	0.08
Pulses	0.04	0.15	0.06	0.18	0.06
Roots & tubers	0.02	0.17	0.14	0.5	0.19
Sugar crops	0.05	0.47	0.39	1.51	0.58
Vegetables	0.01	0.02	0.01	0.02	0.01
**Belgium**					
Cereals	0.06	0.25	0.11	0.36	0.14
Fiber	0	0	0	0	0
Fruit	0	0	0	0	0
Livestock	0.02	0.09	0.04	0.1	0.03
Oil crops	0	0.01	0.01	0.07	0.04
Pulses	0	0	0	0	0
Roots & tubers	0.05	0.15	0.05	0.08	0.02
Sugar crops	0.3	1.24	0.61	1.99	0.73
Vegetables	0	0.01	0.01	0.01	0
**Belize**					
Cereals	0	0	0	0	0
Fiber	0	0	0	0	0
Fruit	0	0.01	0.03	0.01	0.01
Livestock	0	0	0	0	0

Oil crops	0	0	0	0	0
Pulses	0	0	0.01	0	0
Roots & tubers	0	0	0	0	0
Sugar crops	0	0	0	0	0
Vegetables	0	0	0	0	0
**Benin**					
Cereals	0.08	0.13	0.01	0	0
Fiber	0.13	0.42	0.06	0	0
Fruit	0.01	0.01	0	0	0
Livestock	0	0	0	0	0
Oil crops	0.02	0.03	0	0	0
Pulses	0.15	0.26	0.03	0	0
Roots & tubers	0.45	0.57	0.04	0	0
Sugar crops	0	0	0	0	0
Vegetables	0.01	0.01	0	0	0
**Bhutan**					
Cereals	0.01	0.02	0	0	0
Fiber	0	0	0	0	0
Fruit	0	0.01	0	0	0
Livestock	0	0	0	0	0
Oil crops	0	0	0	0	0
Pulses	0.01	0.01	0	0	0
Roots & tubers	0	0	0	0	0
Sugar crops	0	0	0	0	0
Vegetables	0	0	0	0	0
**Bolivia (Plurinational State of)**					
Cereals	0	0.03	0.03	0.06	0.2
Fiber	0	0.01	0.01	0.04	0.19
Fruit	0	0.02	0.02	0.03	0.04
Livestock	0	0.01	0.01	0.02	0.03
Oil crops	0	0.12	0.18	0.85	5.13
Pulses	0	0.02	0.02	0.03	0.08
Roots & tubers	0	0.03	0.03	0.04	0.06
Sugar crops	0	0	0	0	0
Vegetables	0	0	0	0	0.01
**Bosnia and Herzegovina**					
Cereals	0.03	0.12	0.05	0.09	0.01
Fiber	0	0	0	0	0
Fruit	0	0	0	0	0
Livestock	0	0.02	0.01	0.01	0
Oil crops	0	0.02	0.01	0.02	0
Pulses	0.01	0.03	0.01	0.02	0
Roots & tubers	0.01	0.02	0.01	0.02	0
Sugar crops	0	0	0	0	0
Vegetables	0	0	0	0	0

**Botswana**					
Cereals	0	0	0	0	0
Fiber	0	0	0	0	0
Fruit	0	0	0	0	0
Livestock	0	0.01	0	0	0
Oil crops	0	0	0	0	0
Pulses	0	0	0	0	0
Roots & tubers	0	0	0	0	0
Sugar crops	0	0	0	0	0
Vegetables	0	0	0	0	0
**Brazil**					
Cereals	0.02	0.42	0.48	1.52	9.03
Fiber	0	0.07	0.12	0.86	7.24
Fruit	0.04	0.59	0.55	0.88	3.09
Livestock	0.02	0.26	0.26	0.52	1.81
Oil crops	0.06	1.59	2.74	19.68	178.5
Pulses	0.12	1.88	1.75	2.54	6.38
Roots & tubers	0.06	0.92	0.88	1.38	3.24
Sugar crops	0	0	0	0	0
Vegetables	0	0.07	0.07	0.13	0.4
**Brunei Darussalam**					
Cereals	0	0	0	0	0
Fiber	0	0	0	0	0
Fruit	0	0	0	0	0
Livestock	0	0	0	0	0
Oil crops	0	0	0	0	0
Pulses	0	0	0	0	0
Roots & tubers	0	0	0	0	0
Sugar crops	0	0	0	0	0
Vegetables	0	0	0	0	0
**Bulgaria**					
Cereals	0.06	0.34	0.22	0.77	0.28
Fiber	0	0	0	0	0
Fruit	0	0	0	0	0
Livestock	0.01	0.04	0.02	0.04	0.01
Oil crops	0.02	0.19	0.15	0.65	0.27
Pulses	0	0.01	0	0.02	0.01
Roots & tubers	0	0.01	0.01	0.02	0
Sugar crops	0	0	0	0.01	0
Vegetables	0	0.01	0	0.01	0
**Burkina Faso**					
Cereals	0.39	0.76	0.08	0.17	0
Fiber	0.38	0.7	0.08	0.28	0
Fruit	0	0	0	0	0
Livestock	0.02	0.02	0	0	0
Oil crops	0.01	0.02	0	0.01	0

Pulses	0.01	0.01	0	0	0
Roots & tubers	0	0	0	0	0
Sugar crops	0	0	0	0	0
Vegetables	0	0	0	0	0
**Burundi**					
Cereals	0.03	0.03	0	0	0
Fiber	0.01	0.01	0	0	0
Fruit	0.09	0.11	0.01	0	0
Livestock	0	0	0	0	0
Oil crops	0	0.01	0	0	0
Pulses	0.4	0.49	0.02	0	0
Roots & tubers	0.09	0.11	0.01	0	0
Sugar crops	0	0	0	0	0
Vegetables	0	0	0	0	0
**Cambodia**					
Cereals	0.17	0.38	0.03	0.01	0
Fiber	0	0	0	0	0
Fruit	0.01	0.02	0	0	0
Livestock	0.01	0.01	0	0	0
Oil crops	0.2	0.33	0.02	0.01	0
Pulses	0.08	0.13	0.01	0	0
Roots & tubers	0.13	0.23	0.01	0	0
Sugar crops	0	0	0	0	0
Vegetables	0	0	0	0	0
**Cameroon**					
Cereals	0.17	0.26	0.02	0.02	0
Fiber	0.23	0.39	0.04	0.01	0
Fruit	0.15	0.24	0.02	0.03	0
Livestock	0.01	0.01	0	0	0
Oil crops	0.01	0.02	0	0	0
Pulses	0.43	0.52	0.03	0	0
Roots & tubers	0.19	0.34	0.03	0.13	0
Sugar crops	0	0	0	0	0
Vegetables	0.03	0.05	0	0	0
**Canada**					
Cereals	0.01	1.25	1.98	6.4	6.85
Fiber	0	0	0	0	0
Fruit	0	0	0	0.01	0
Livestock	0	0.09	0.14	0.32	0.18
Oil crops	0.02	4.27	6.55	18.2	17.86
Pulses	0	0.7	1.09	3.43	3.76
Roots & tubers	0	0.07	0.11	0.25	0.15
Sugar crops	0	0.02	0.03	0.22	0.37
Vegetables	0	0.01	0.02	0.07	0.04
**Central African Republic**					

Cereals	0.02	0.03	0	0	0
Fiber	0	0	0	0	0
Fruit	0.01	0.02	0	0	0
Oil crops	0.02	0.02	0	0	0
Pulses	0.01	0.01	0	0	0
Roots & tubers	0.09	0.11	0.01	0	0
Sugar crops	0	0	0	0	0
Vegetables	0	0	0	0	0
**Chad**					
Cereals	0.22	0.4	0.04	0.2	0
Fiber	0.1	0.19	0.02	0.03	0
Fruit	0	0	0	0	0
Livestock	0.01	0.01	0	0	0
Oil crops	0.01	0.02	0	0	0
Pulses	0.1	0.18	0.02	0.06	0
Roots & tubers	0.04	0.07	0.01	0.02	0
Sugar crops	0	0	0	0	0
Vegetables	0	0	0	0	0
**Chile**					
Cereals	0.01	0.1	0.1	0.21	0.7
Fiber	0	0	0	0	0
Fruit	0	0.01	0.01	0.02	0.07
Livestock	0	0.02	0.02	0.05	0.14
Oil crops	0	0.02	0.02	0.03	0.11
Pulses	0	0.02	0.02	0.03	0.09
Roots & tubers	0	0.01	0.01	0.02	0.09
Sugar crops	0.01	0.16	0.17	0.32	1.14
Vegetables	0	0.02	0.02	0.04	0.14
**China**					
Cereals	53.11	24.91	0	13.43	0
Fiber	20.62	14.86	0	11.31	0
Fruit	1.55	0.84	0	0.26	0
Livestock	9.45	3.82	0	1.62	0
Oil crops	51.35	31.81	0	18.43	0
Pulses	5.16	2.53	0	1.78	0
Roots & tubers	10.93	3.34	0	1.14	0
Sugar crops	2.02	2.79	0	2	0
Vegetables	4.76	2.23	0	0.95	0
**Colombia**					
Cereals	0	0.02	0.03	0.09	0.39
Fiber	0	0.04	0.04	0.06	0.15
Fruit	0	0.07	0.07	0.16	0.48
Livestock	0	0.08	0.07	0.13	0.33
Oil crops	0	0.01	0.01	0.03	0.17
Pulses	0	0.08	0.08	0.16	0.37
Roots & tubers	0	0.08	0.08	0.14	0.41

Sugar crops	0	0	0	0	0.01
Vegetables	0	0.02	0.02	0.03	0.07
**Congo**					
Cereals	0	0.01	0	0	0
Fiber	0	0	0	0	0
Fruit	0	0.01	0	0	0
Livestock	0	0	0	0	0
Oil crops	0	0	0	0	0
Pulses	0.01	0.02	0	0	0
Roots & tubers	0.08	0.15	0.02	0.01	0
Sugar crops	0	0	0	0	0
Vegetables	0	0	0	0	0
**Costa Rica**					
Cereals	0	0	0	0	0.01
Fiber	0	0	0	0	0
Fruit	0	0.04	0.14	0.13	0.22
Livestock	0	0	0.01	0.02	0.03
Oil crops	0	0	0	0	0
Pulses	0	0	0.01	0.01	0.02
Roots & tubers	0	0	0.01	0.01	0.01
Sugar crops	0	0	0	0	0
Vegetables	0	0	0	0	0.01
**Croatia**					
Cereals	0.07	0.24	0.09	0.26	0.09
Fiber	0	0	0	0	0
Fruit	0	0	0	0	0
Livestock	0.01	0.02	0.01	0.03	0.01
Oil crops	0.07	0.23	0.08	0.23	0.08
Pulses	0	0.01	0	0.01	0
Roots & tubers	0	0.01	0	0.01	0
Sugar crops	0.11	0.4	0.15	0.44	0.15
Vegetables	0	0	0	0	0
**Cuba**					
Cereals	0.01	0.02	0.02	0.02	0.03
Fiber	0	0	0	0	0
Fruit	0.02	0.04	0.04	0.03	0.06
Livestock	0	0.01	0.02	0.01	0.03
Oil crops	0	0	0	0	0
Pulses	0.04	0.09	0.08	0.06	0.09
Roots & tubers	0.02	0.05	0.05	0.03	0.05
Sugar crops	0	0	0	0	0
Vegetables	0.01	0.03	0.03	0.02	0.03
**Cyprus**					
Cereals	0	0	0	0	0
Fiber	0	0	0	0	0
Fruit	0	0	0	0	0

Livestock	0	0.01	0	0	0
Oil crops	0	0	0	0	0
Pulses	0	0	0	0	0
Roots & tubers	0	0	0	0	0
Sugar crops	0	0	0	0	0
Vegetables	0	0	0	0	0
**Czech Republic**					
Cereals	0.18	0.68	0.29	0.87	0.31
Fiber	0	0	0	0	0
Fruit	0	0	0	0	0
Livestock	0.01	0.05	0.03	0.1	0.04
Oil crops	0.25	0.98	0.43	1.37	0.49
Pulses	0.02	0.07	0.02	0.06	0.02
Roots & tubers	0	0.02	0.02	0.04	0.01
Sugar crops	0.26	0.87	0.3	0.9	0.34
Vegetables	0	0	0	0	0
**Democratic Republic of the Congo**					
Cereals	0.08	0.15	0.01	0.04	0
Fiber	0.02	0.04	0	0.01	0
Fruit	0.08	0.13	0.01	0.04	0
Livestock	0	0	0	0	0
Oil crops	0.02	0.03	0	0	0
Pulses	0.14	0.23	0.02	0.05	0
Roots & tubers	0.96	1.81	0.19	0.49	0
Sugar crops	0	0	0	0	0
Vegetables	0	0.01	0	0	0
**Denmark**					
Cereals	0.06	0.54	0.42	1.4	0.49
Fiber	0	0	0	0	0
Fruit	0	0	0	0	0
Livestock	0.01	0.07	0.06	0.16	0.04
Oil crops	0.04	0.32	0.25	0.82	0.28
Pulses	0	0.01	0.01	0.04	0.01
Roots & tubers	0	0.03	0.03	0.09	0.03
Sugar crops	0.05	0.38	0.29	0.99	0.34
Vegetables	0	0	0	0.01	0
**Djibouti**					
Cereals	0	0	0	0	0
Fiber	0	0	0	0	0
Fruit	0	0	0	0	0
Livestock	0	0	0	0	0
Oil crops	0	0	0	0	0
Pulses	0	0	0	0	0
Roots & tubers	0	0	0	0	0
Sugar crops	0	0	0	0	0

Vegetables	0	0	0	0	0
**Dominica**					
Cereals	0	0	0	0	0
Fiber	0	0	0	0	0
Fruit	0	0	0	0	0
Livestock	0	0	0	0	0
Oil crops	0	0	0	0	0
Pulses	0	0	0	0	0
Roots & tubers	0	0	0	0	0
Sugar crops	0	0	0	0	0
Vegetables	0	0	0	0	0
**Ecuador**					
Cereals	0	0.05	0.05	0.07	0.13
Fiber	0	0	0	0	0
Fruit	0.01	0.2	0.18	0.2	0.3
Livestock	0	0.04	0.04	0.06	0.15
Oil crops	0	0.01	0.01	0.04	0.12
Pulses	0	0.01	0.01	0.02	0.03
Roots & tubers	0	0.01	0.01	0.01	0.03
Sugar crops	0	0	0	0	0
Vegetables	0	0	0	0	0
**Egypt**					
Cereals	0.63	2.92	1.08	0.53	0.09
Fiber	0.17	0.76	0.28	0.14	0.02
Fruit	0.09	0.43	0.16	0.08	0.01
Livestock	0.05	0.24	0.09	0.04	0.01
Oil crops	0.02	0.11	0.04	0.02	0
Pulses	0.03	0.12	0.05	0.02	0
Roots & tubers	0.04	0.2	0.07	0.04	0.01
Sugar crops	0.43	1.99	0.74	0.36	0.06
Vegetables	0.16	0.75	0.28	0.14	0.02
**El Salvador**					
Cereals	0.02	0.05	0.06	0.03	0.01
Fiber	0	0	0	0	0
Fruit	0	0.01	0.01	0	0
Livestock	0.01	0.02	0.02	0.01	0
Oil crops	0	0.01	0.01	0	0
Pulses	0.02	0.07	0.13	0.06	0.02
Roots & tubers	0	0	0	0	0
Sugar crops	0	0	0	0	0
Vegetables	0	0	0	0	0
**Equatorial Guinea**					
Cereals	0	0	0	0	0
Fiber	0	0	0	0	0
Fruit	0	0	0	0	0
Livestock	0	0	0	0	0

Oil crops	0	0	0	0	0
Pulses	0	0	0	0	0
Roots & tubers	0.01	0.01	0	0	0
Sugar crops	0	0	0	0	0
Vegetables	0	0	0	0	0
**Eritrea**					
Cereals	0.03	0.08	0.01	0	0
Fiber	0	0	0	0	0
Fruit	0	0	0	0	0
Livestock	0	0.01	0	0	0
Oil crops	0	0.01	0	0	0
Pulses	0	0.01	0	0	0
Roots & tubers	0	0	0	0	0
Sugar crops	0	0	0	0	0
Vegetables	0	0	0	0	0
**Estonia**					
Cereals	0	0.03	0.02	0.13	0.06
Fiber	0	0	0	0	0
Fruit	0	0	0	0	0
Livestock	0	0.01	0.01	0.02	0.01
Oil crops	0	0.04	0.03	0.2	0.1
Pulses	0	0	0	0.01	0
Roots & tubers	0	0	0	0.01	0.01
Sugar crops	0	0	0	0	0
Vegetables	0	0	0	0	0
**Ethiopia**					
Cereals	1.24	2.33	0.25	0.27	0
Fiber	0.06	0.11	0.01	0.02	0
Fruit	0.01	0.02	0	0	0
Livestock	0.06	0.1	0.01	0	0
Oil crops	0.09	0.14	0.01	0.01	0
Pulses	0.5	0.71	0.05	0.05	0
Roots & tubers	0.04	0.08	0.01	0.01	0
Sugar crops	0	0	0	0	0
Vegetables	0.01	0.02	0	0	0
**Finland**					
Cereals	0.04	0.31	0.24	0.62	0.14
Fiber	0	0	0	0	0
Fruit	0	0	0	0	0
Livestock	0.01	0.04	0.03	0.07	0.01
Oil crops	0.01	0.11	0.08	0.21	0.05
Pulses	0	0.01	0	0.01	0
Roots & tubers	0	0.02	0.01	0.04	0.01
Sugar crops	0.02	0.18	0.14	0.36	0.08
Vegetables	0	0	0	0	0
**France**					

Cereals	0.7	3.7	2.35	9.45	3.78
Fiber	0	0	0	0	0
Fruit	0.01	0.05	0.03	0.07	0.02
Livestock	0.14	0.6	0.29	0.67	0.15
Oil crops	0.62	3.08	1.88	9.18	4.14
Pulses	0.15	0.67	0.36	1.15	0.4
Roots & tubers	0.01	0.11	0.11	0.43	0.16
Sugar crops	0.13	2.2	2.36	14.7	6.98
Vegetables	0.01	0.03	0.02	0.06	0.02
**Gabon**					
Cereals	0	0	0	0	0
Fiber	0	0	0	0	0
Fruit	0.02	0.02	0	0	0
Livestock	0	0	0	0	0
Oil crops	0	0	0	0	0
Pulses	0	0	0	0	0
Roots & tubers	0.04	0.05	0	0	0
Sugar crops	0	0	0	0	0
Vegetables	0	0	0	0	0
**Gambia**					
Cereals	0.03	0.07	0.01	0	0
Fiber	0	0	0	0	0
Fruit	0	0	0	0	0
Livestock	0	0	0	0	0
Oil crops	0	0	0	0	0
Pulses	0	0	0	0	0
Roots & tubers	0	0	0	0	0
Sugar crops	0	0	0	0	0
Vegetables	0	0	0	0	0
**Georgia**					
Cereals	0.01	0.07	0.02	0.01	0
Fiber	0	0	0	0	0
Fruit	0	0	0	0	0
Livestock	0.01	0.03	0.01	0	0
Oil crops	0	0.02	0.01	0	0
Pulses	0.01	0.03	0.01	0.01	0
Roots & tubers	0	0.02	0.01	0	0
Sugar crops	0	0	0	0	0
Vegetables	0	0.01	0	0	0
**Germany**					
Cereals	0.57	3	1.82	6.27	2.29
Fiber	0	0	0	0	0
Fruit	0.01	0.02	0.01	0.01	0
Livestock	0.07	0.38	0.24	0.94	0.38
Oil crops	0.69	3.67	2.3	9.28	3.77
Pulses	0.04	0.18	0.09	0.32	0.12

Roots & tubers	0.04	0.25	0.17	0.63	0.24
Sugar crops	0.54	4.31	3.23	9.15	2.66
Vegetables	0	0.02	0.01	0.03	0.01
**Ghana**					
Cereals	0.17	0.24	0.02	0	0
Fiber	0.02	0.03	0	0	0
Fruit	0.22	0.3	0.02	0	0
Livestock	0.01	0.01	0	0	0
Oil crops	0.01	0.03	0	0	0
Pulses	0.25	0.31	0.02	0	0
Roots & tubers	1.23	1.7	0.13	0	0
Sugar crops	0	0	0	0	0
Vegetables	0.01	0.02	0	0	0
**Greece**					
Cereals	0.14	0.55	0.24	0.4	0.04
Fiber	0.25	1	0.45	0.78	0.09
Fruit	0.03	0.09	0.04	0.06	0.01
Livestock	0.02	0.07	0.03	0.04	0
Oil crops	0.01	0.02	0.01	0.01	0
Pulses	0.01	0.04	0.01	0.02	0
Roots & tubers	0.01	0.04	0.02	0.03	0
Sugar crops	0.16	0.62	0.25	0.41	0.04
Vegetables	0.03	0.11	0.04	0.06	0.01
**Grenada**					
Cereals	0	0	0	0	0
Fiber	0	0	0	0	0
Fruit	0	0	0	0	0
Livestock	0	0	0	0	0
Oil crops	0	0	0	0	0
Pulses	0	0	0	0	0
Roots & tubers	0	0	0	0	0
Sugar crops	0	0	0	0	0
Vegetables	0	0	0	0	0
**Guatemala**					
Cereals	0.01	0.04	0.08	0.05	0.07
Fiber	0	0	0	0	0
Fruit	0.02	0.06	0.1	0.06	0.06
Livestock	0.01	0.03	0.04	0.02	0.02
Oil crops	0.01	0.03	0.06	0.03	0.04
Pulses	0.03	0.1	0.16	0.1	0.12
Roots & tubers	0	0.01	0.02	0.01	0.01
Sugar crops	0	0	0	0	0
Vegetables	0	0.01	0.02	0.01	0.01
**Guinea**					
Cereals	0.14	0.29	0.03	0	0
Fiber	0.03	0.06	0.01	0	0

Fruit	0.03	0.06	0.01	0	0
Livestock	0.01	0.01	0	0	0
Oil crops	0	0	0	0	0
Pulses	0.01	0.03	0	0	0
Roots & tubers	0.09	0.16	0.02	0	0
Sugar crops	0	0	0	0	0
Vegetables	0	0	0	0	0
**Guinea-Bissau**					
Cereals	0.02	0.03	0	0	0
Fiber	0.01	0.01	0	0	0
Fruit	0	0	0	0	0
Livestock	0	0	0	0	0
Oil crops	0	0	0	0	0
Pulses	0	0	0	0	0
Roots & tubers	0	0.01	0	0	0
Sugar crops	0	0	0	0	0
Vegetables	0	0	0	0	0
**Guyana**					
Cereals	0	0	0	0	0.03
Fiber	0	0	0	0	0
Fruit	0	0	0	0	0
Livestock	0	0	0	0	0
Oil crops	0	0	0	0	0.01
Pulses	0	0	0	0	0
Roots & tubers	0	0	0	0	0
Sugar crops	0	0	0	0	0
Vegetables	0	0	0	0	0
**Haiti**					
Cereals	0.01	0.03	0.02	0.01	0
Fiber	0	0	0	0	0
Fruit	0.01	0.03	0.03	0.01	0
Livestock	0	0	0	0	0
Oil crops	0	0	0	0	0
Pulses	0.03	0.07	0.06	0.03	0.01
Roots & tubers	0.02	0.05	0.05	0.02	0.01
Sugar crops	0	0	0	0	0
Vegetables	0	0	0	0	0
**Honduras**					
Cereals	0.01	0.02	0.02	0.02	0.05
Fiber	0	0	0	0	0
Fruit	0.02	0.03	0.03	0.03	0.07
Livestock	0.01	0.01	0.01	0.01	0.03
Oil crops	0	0	0	0	0
Pulses	0.02	0.03	0.03	0.05	0.15
Roots & tubers	0	0	0	0	0
Sugar crops	0	0	0	0	0

Vegetables	0	0.01	0	0	0.01
**Hungary**					
Cereals	0.29	1.13	0.49	1.3	0.43
Fiber	0	0	0	0	0
Fruit	0	0.01	0	0.01	0
Livestock	0.01	0.05	0.03	0.08	0.02
Oil crops	0.19	0.81	0.39	1.21	0.43
Pulses	0.01	0.04	0.02	0.06	0.02
Roots & tubers	0.01	0.02	0.01	0.02	0.01
Sugar crops	0.22	0.8	0.3	0.65	0.17
Vegetables	0.01	0.02	0.01	0.01	0
**Iceland**					
Cereals	0	0	0	0	0
Fiber	0	0	0	0	0
Fruit	0	0	0	0	0
Livestock	0	0	0	0	0
Oil crops	0	0	0	0	0
Pulses	0	0	0	0	0
Roots & tubers	0	0	0	0	0
Sugar crops	0	0	0	0	0
Vegetables	0	0	0	0	0
**India**					
Cereals	18.54	33.22	3.75	0.71	0
Fiber	6.23	18.4	2.54	0.48	0
Fruit	1.02	2.35	0.3	0.06	0
Livestock	1.88	3.98	0.48	0.09	0
Oil crops	19.06	42.43	5.26	1.01	0
Pulses	4.02	9.94	1.28	0.25	0
Roots & tubers	1.81	3.11	0.34	0.06	0
Sugar crops	0	0	0	0	0
Vegetables	1.13	1.88	0.2	0.04	0
**Indonesia**					
Cereals	2.43	3.37	0.12	0.9	0
Fiber	0	0.01	0	0	0
Fruit	0.35	0.62	0.03	0.32	0
Livestock	0.27	0.35	0.01	0.01	0
Oil crops	1.82	2.54	0.09	0.71	0
Pulses	0.33	0.59	0.03	0.31	0
Roots & tubers	1.81	2.59	0.09	0.25	0
Sugar crops	0	0	0	0	0
Vegetables	0.06	0.1	0.01	0.05	0
**Iran (Islamic Republic of)**					
Cereals	1.93	3.76	0.43	1.51	0
Fiber	0.24	0.39	0.04	0.07	0
Fruit	0.19	0.42	0.05	0.15	0

Livestock	0.12	0.4	0.06	0.18	0
Oil crops	0.18	1.45	0.24	0.19	0
Pulses	0.2	0.46	0.06	0.16	0
Roots & tubers	0.11	0.22	0.03	0.16	0
Sugar crops	0.54	2.49	0.38	1.36	0
Vegetables	0.23	0.5	0.06	0.24	0
**Iraq**					
Cereals	0.21	0.67	0.18	0.1	0.04
Fiber	0.01	0.04	0.01	0.01	0
Fruit	0.01	0.04	0.02	0.01	0
Livestock	0.01	0.02	0.01	0.01	0.01
Oil crops	0	0.01	0	0	0
Pulses	0	0.01	0	0	0
Roots & tubers	0.01	0.04	0.02	0.01	0.01
Sugar crops	0	0.01	0	0	0
Vegetables	0.02	0.09	0.03	0.02	0
**Ireland**					
Cereals	0.02	0.13	0.09	0.29	0.09
Fiber	0	0	0	0	0
Fruit	0	0	0	0	0
Livestock	0.03	0.12	0.06	0.11	0.02
Oil crops	0	0.02	0.01	0.04	0.01
Pulses	0	0.01	0.01	0.03	0.01
Roots & tubers	0	0.01	0.01	0.02	0.01
Sugar crops	0.02	0.21	0.18	0.58	0.18
Vegetables	0	0	0	0	0
**Israel**					
Cereals	0.02	0.1	0.04	0.02	0
Fiber	0.02	0.13	0.05	0.02	0
Fruit	0.02	0.08	0.03	0.01	0
Livestock	0.03	0.14	0.05	0.02	0
Oil crops	0	0.02	0.01	0	0
Pulses	0	0	0	0	0
Roots & tubers	0.02	0.08	0.03	0.01	0
Sugar crops	0	0	0	0	0
Vegetables	0.02	0.08	0.03	0.01	0
**Italy**					
Cereals	0.2	1.29	0.89	2.58	0.75
Fiber	0	0	0	0	0
Fruit	0.03	0.18	0.11	0.32	0.09
Livestock	0.05	0.3	0.2	0.41	0.06
Oil crops	0.07	0.59	0.45	1	0.17
Pulses	0.01	0.04	0.03	0.07	0.02
Roots & tubers	0.01	0.06	0.03	0.08	0.02
Sugar crops	0.22	1.65	1.23	2.92	0.59
Vegetables	0.05	0.25	0.16	0.41	0.11

**Ivory Coast**					
Cereals	0	0	0	0	0
Fiber	0	0	0	0	0
Fruit	0	0	0	0	0
Livestock	0	0.01	0	0	0
Oil crops	0	0	0	0	0
Pulses	0	0	0	0	0
Roots & tubers	0	0	0	0	0
Sugar crops	0	0	0	0	0
Vegetables	0	0	0	0	0
**Jamaica**					
Cereals	0	0	0	0	0
Fiber	0	0	0	0	0
Fruit	0.01	0.02	0	0	0
Livestock	0	0.01	0	0	0
Oil crops	0.01	0.02	0	0	0
Pulses	0	0	0	0	0
Roots & tubers	0.01	0.02	0	0	0
Sugar crops	0	0	0	0	0
Vegetables	0	0	0	0	0
**Japan**					
Cereals	0.38	0.54	0.22	0.15	0
Fiber	0	0	0	0	0
Fruit	0.01	0.02	0.01	0.01	0
Livestock	0.72	0.54	0.15	0.15	0
Oil crops	0.26	0.34	0.14	0.11	0
Pulses	0.11	0.15	0.06	0.05	0
Roots & tubers	0.16	0.18	0.07	0.05	0
Sugar crops	1.01	1.43	0.59	0.45	0
Vegetables	0.09	0.12	0.05	0.04	0
**Jordan**					
Cereals	0	0.01	0	0	0
Fiber	0	0	0	0	0
Fruit	0	0.01	0	0	0
Livestock	0	0.02	0.01	0	0
Oil crops	0	0	0	0	0
Pulses	0	0	0	0	0
Roots & tubers	0	0.01	0	0	0
Sugar crops	0	0	0	0	0
Vegetables	0.01	0.04	0.02	0.01	0
**Kazakhstan**					
Cereals	0.08	1.55	0.73	1.53	2.66
Fiber	0.05	0.38	0.16	0.19	0.26
Fruit	0	0	0	0	0
Livestock	0.02	0.14	0.06	0.06	0.07
Oil crops	0.02	0.31	0.14	0.17	0.22

Pulses	0	0.03	0.02	0.02	0.04
Roots & tubers	0.01	0.11	0.05	0.05	0.07
Sugar crops	0.01	0.13	0.06	0.04	0.03
Vegetables	0.01	0.06	0.02	0.02	0.01
**Kenya**					
Cereals	0.2	0.42	0.05	0.17	0
Fiber	0.01	0.03	0	0.01	0
Fruit	0.05	0.1	0.01	0.05	0
Livestock	0.06	0.12	0.01	0.04	0
Oil crops	0.01	0.02	0	0.01	0
Pulses	0.46	0.91	0.1	0.3	0
Roots & tubers	0.1	0.23	0.03	0.1	0
Sugar crops	0	0	0	0	0
Vegetables	0.02	0.04	0.01	0.02	0
**Kuwait**					
Cereals	0	0	0	0	0
Fiber	0	0	0	0	0
Fruit	0	0	0	0	0
Livestock	0	0.01	0.01	0	0
Oil crops	0	0	0	0	0
Pulses	0	0	0	0	0
Roots & tubers	0	0	0	0	0
Sugar crops	0	0	0	0	0
Vegetables	0	0.01	0	0	0
**Kyrgyzstan**					
Cereals	0.03	0.18	0.06	0.09	0.13
Fiber	0.02	0.1	0.04	0.04	0.06
Fruit	0	0	0	0	0
Livestock	0.01	0.04	0.01	0.01	0.01
Oil crops	0.01	0.04	0.01	0.01	0.01
Pulses	0.01	0.12	0.06	0.03	0.01
Roots & tubers	0.01	0.05	0.02	0.03	0.04
Sugar crops	0.01	0.07	0.02	0.03	0.05
Vegetables	0	0.02	0.01	0.01	0.01
**Lao People's Democratic Republic**					
Cereals	0.11	0.19	0.01	0	0
Fiber	0.01	0.01	0	0	0
Fruit	0.01	0.02	0	0	0
Livestock	0	0.01	0	0	0
Oil crops	0.02	0.03	0	0	0
Pulses	0.01	0.01	0	0	0
Roots & tubers	0.02	0.03	0	0	0
Sugar crops	0	0	0	0	0
Vegetables	0	0	0	0	0
**Latvia**					

Cereals	0.01	0.07	0.06	0.24	0.1
Fiber	0	0	0	0	0
Fruit	0	0	0	0	0
Livestock	0	0.01	0.01	0.03	0.01
Oil crops	0.01	0.09	0.07	0.29	0.12
Pulses	0	0	0	0	0
Roots & tubers	0	0.01	0.01	0.04	0.02
Sugar crops	0	0.04	0.03	0.14	0.06
Vegetables	0	0	0	0	0
**Lebanon**					
Cereals	0.01	0.03	0.01	0.01	0.01
Fiber	0	0	0	0	0
Fruit	0.01	0.03	0.01	0.01	0.01
Livestock	0	0.01	0	0.01	0.02
Oil crops	0	0	0	0	0
Pulses	0	0	0	0	0
Roots & tubers	0	0.02	0.01	0.01	0.02
Sugar crops	0	0.02	0.01	0.01	0.01
Vegetables	0	0.02	0.01	0.01	0.01
**Lesotho**					
Cereals	0.01	0.01	0	0	0
Fiber	0	0	0	0	0
Fruit	0	0	0	0	0
Livestock	0	0	0	0	0
Oil crops	0	0	0	0	0
Pulses	0.01	0.01	0	0	0
Roots & tubers	0	0.01	0	0	0
Sugar crops	0	0	0	0	0
Vegetables	0	0	0	0	0
**Liberia**					
Cereals	0	0.01	0	0	0
Fiber	0	0	0	0	0
Fruit	0.01	0.02	0	0	0
Livestock	0	0	0	0	0
Oil crops	0	0.01	0	0	0
Pulses	0	0	0	0	0
Roots & tubers	0.03	0.08	0.01	0	0
Sugar crops	0	0	0	0	0
Vegetables	0	0	0	0	0
**Libya**					
Cereals	0.01	0.03	0.01	0.01	0.02
Fiber	0	0	0	0	0
Fruit	0	0.01	0	0.01	0.01
Livestock	0	0.02	0.01	0.01	0
Oil crops	0	0	0	0	0
Pulses	0	0.01	0	0	0

Roots & tubers	0	0.01	0	0.01	0.01
Sugar crops	0	0	0	0	0
Vegetables	0	0.02	0.01	0.01	0.02
**Lithuania**					
Cereals	0.03	0.17	0.11	0.35	0.12
Fiber	0	0	0	0	0
Fruit	0	0	0	0	0
Livestock	0.01	0.03	0.02	0.06	0.02
Oil crops	0.03	0.18	0.11	0.39	0.14
Pulses	0.01	0.02	0.01	0.03	0.01
Roots & tubers	0	0.02	0.01	0.03	0.01
Sugar crops	0.01	0.11	0.09	0.33	0.11
Vegetables	0	0	0	0	0
**Luxembourg**					
Cereals	0	0.01	0.01	0.03	0
Fiber	0	0	0	0	0
Fruit	0	0	0	0	0
Livestock	0	0	0	0.01	0
Oil crops	0	0.01	0.01	0.03	0.01
Pulses	0	0	0	0	0
Roots & tubers	0	0	0	0	0
Sugar crops	0	0	0	0	0
Vegetables	0	0	0	0	0
**Madagascar**					
Cereals	0.16	0.19	0.01	0	0
Fiber	0.01	0.02	0	0	0
Fruit	0.03	0.03	0	0	0
Livestock	0.02	0.02	0	0	0
Oil crops	0.01	0.01	0	0	0
Pulses	0.16	0.19	0.01	0	0
Roots & tubers	0.34	0.41	0.02	0	0
Sugar crops	0	0	0	0	0
Vegetables	0	0	0	0	0
**Malawi**					
Cereals	0.18	0.26	0.02	0.03	0
Fiber	0.05	0.07	0.01	0	0
Fruit	0.03	0.05	0.01	0	0
Livestock	0.01	0.01	0	0	0
Oil crops	0.09	0.11	0.01	0	0
Pulses	0.19	0.27	0.02	0.01	0
Roots & tubers	0.35	0.48	0.04	0.03	0
Sugar crops	0	0	0	0	0
Vegetables	0.01	0.01	0	0	0
**Malaysia**					
Cereals	0.01	0.09	0.01	0.09	0
Fiber	0	0	0	0	0

Fruit	0.02	0.04	0	0.02	0
Livestock	0.04	0.15	0.01	0.03	0
Oil crops	0.02	0.04	0	0.02	0
Pulses	0	0	0	0	0
Roots & tubers	0	0.01	0	0	0
Sugar crops	0	0	0	0	0
Vegetables	0	0	0	0	0
**Mali**					
Cereals	0.44	0.82	0.09	0	0
Fiber	0.31	0.57	0.06	0	0
Fruit	0.01	0.01	0	0	0
Livestock	0.02	0.04	0	0	0
Oil crops	0	0.01	0	0	0
Pulses	0	0	0	0	0
Roots & tubers	0.02	0.04	0	0	0
Sugar crops	0	0	0	0	0
Vegetables	0.01	0.01	0	0	0
**Mauritania**					
Cereals	0.01	0.02	0	0	0
Fiber	0	0	0	0	0
Fruit	0	0	0	0	0
Livestock	0.01	0.01	0	0	0
Oil crops	0	0	0	0	0
Pulses	0.01	0.02	0	0	0
Roots & tubers	0	0	0	0	0
Sugar crops	0	0	0	0	0
Vegetables	0	0	0	0	0
**Mauritius**					
Cereals	0	0	0	0	0
Fiber	0	0	0	0	0
Fruit	0	0	0	0	0
Livestock	0	0	0	0	0
Oil crops	0	0	0	0	0
Pulses	0	0	0	0	0
Roots & tubers	0	0	0	0	0
Sugar crops	0	0	0	0	0
Vegetables	0	0	0	0	0
**Mexico**					
Cereals	0.88	1.89	1.57	1.08	1.55
Fiber	0	0.02	0.06	0.2	0.67
Fruit	0.08	0.25	0.37	0.24	0.34
Livestock	0.16	0.4	0.49	0.28	0.3
Oil crops	0.14	0.25	0.1	0.06	0.09
Pulses	0.87	1.63	0.68	0.44	0.61
Roots & tubers	0.02	0.05	0.05	0.04	0.05
Sugar crops	0	0	0	0	0

Vegetables	0.05	0.12	0.13	0.11	0.21
**Mongolia**					
Cereals	0.01	0.01	0	0.03	0
Fiber	0	0	0	0	0
Fruit	0	0	0	0	0
Livestock	0.01	0.01	0	0.01	0
Oil crops	0	0.02	0.01	0.01	0
Pulses	0	0	0	0	0
Roots & tubers	0	0	0	0.01	0
Sugar crops	0	0	0	0	0
Vegetables	0	0	0	0	0
**Montenegro**					
Cereals	0	0	0	0	0
Fiber	0	0	0	0	0
Fruit	0	0	0	0	0
Livestock	0.02	0.07	0.02	0.03	0
Oil crops	0	0	0	0	0
Pulses	0	0	0	0	0
Roots & tubers	0	0.01	0	0	0
Sugar crops	0	0	0	0	0
Vegetables	0	0	0	0	0
**Morocco**					
Cereals	0.23	0.85	0.25	0.25	0.31
Fiber	0	0	0	0	0
Fruit	0.03	0.1	0.03	0.03	0.03
Livestock	0.04	0.12	0.03	0.02	0.01
Oil crops	0.01	0.02	0.01	0	0
Pulses	0.01	0.05	0.02	0.01	0.01
Roots & tubers	0.03	0.09	0.02	0.02	0.02
Sugar crops	0.31	1.07	0.31	0.29	0.36
Vegetables	0.04	0.15	0.04	0.03	0.03
**Mozambique**					
Cereals	0.07	0.19	0.03	0.02	0
Fiber	0.04	0.07	0.01	0.24	0
Fruit	0.01	0.02	0	0	0
Livestock	0	0.01	0	0	0
Oil crops	0.02	0.04	0	0.01	0
Pulses	0.21	0.34	0.03	0.02	0
Roots & tubers	0.32	0.79	0.1	0.01	0
Sugar crops	0	0	0	0	0
Vegetables	0.01	0.01	0	0	0
**Myanmar**					
Cereals	0.97	1.78	0.1	0.04	0
Fiber	0.15	0.25	0.01	0.03	0
Fruit	0.03	0.06	0	0	0
Livestock	0.08	0.11	0	0	0

Oil crops	0.72	1.18	0.05	0.02	0
Pulses	3.58	6	0.28	0.1	0
Roots & tubers	0.04	0.07	0	0	0
Sugar crops	0	0	0	0	0
Vegetables	0.06	0.09	0	0	0
**Namibia**					
Cereals	0.02	0.05	0.01	0	0
Fiber	0	0	0	0	0
Fruit	0	0	0	0	0
Livestock	0	0.01	0	0	0
Oil crops	0	0	0	0	0
Pulses	0	0.01	0	0	0
Roots & tubers	0	0	0	0	0
Sugar crops	0	0	0	0	0
Vegetables	0	0	0	0	0
**Nepal**					
Cereals	0.49	0.92	0.11	0.02	0
Fiber	0	0	0	0	0
Fruit	0.01	0.01	0	0	0
Livestock	0.03	0.06	0.01	0	0
Oil crops	0.02	0.05	0.01	0	0
Pulses	0.04	0.07	0.01	0	0
Roots & tubers	0.07	0.13	0.02	0	0
Sugar crops	0	0	0	0	0
Vegetables	0	0	0	0	0
**Netherlands**					
Cereals	0.02	0.09	0.06	0.29	0.13
Fiber	0	0	0	0	0
Fruit	0	0	0	0	0
Livestock	0.03	0.21	0.15	0.4	0.1
Oil crops	0	0	0	0.02	0.01
Pulses	0	0	0	0.01	0.01
Roots & tubers	0.01	0.1	0.07	0.42	0.2
Sugar crops	0.13	0.73	0.47	2.32	1.07
Vegetables	0.01	0.05	0.03	0.13	0.06
**New Zealand**					
Cereals	0	0.01	0.01	0.04	0.2
Fiber	0	0	0	0	0
Fruit	0	0	0	0	0.01
Livestock	0	0.03	0.04	0.18	0.62
Oil crops	0	0	0	0	0.01
Pulses	0	0	0	0.01	0.07
Roots & tubers	0	0	0	0.01	0.05
Sugar crops	0	0	0	0	0
Vegetables	0	0	0	0	0.01
**Nicaragua**					

Cereals	0.02	0.04	0.04	0.02	0.02
Fiber	0	0	0	0	0
Fruit	0	0.01	0.01	0.01	0.01
Livestock	0	0.01	0.01	0.01	0.01
Oil crops	0	0.01	0	0	0
Pulses	0.17	0.31	0.13	0.07	0.06
Roots & tubers	0	0.01	0.01	0	0
Sugar crops	0	0	0	0	0
Vegetables	0	0	0	0	0
**Niger**					
Cereals	0.93	1.29	0.1	0.28	0
Fiber	0.01	0.01	0	0	0
Fruit	0	0	0	0	0
Livestock	0.02	0.03	0	0	0
Oil crops	0.02	0.02	0	0	0
Pulses	0.02	0.03	0	0	0
Roots & tubers	0.01	0.02	0	0	0
Sugar crops	0	0	0	0	0
Vegetables	0.03	0.04	0	0.01	0
**Nigeria**					
Cereals	3.5	5.82	0.55	0.09	0
Fiber	0.39	0.73	0.08	0	0
Fruit	0.14	0.27	0.03	0.04	0
Livestock	0.06	0.12	0.01	0.12	0
Oil crops	0.72	1.33	0.14	0.14	0
Pulses	0.02	0.03	0	0	0
Roots & tubers	4.71	9.64	1.09	1.49	0
Sugar crops	0	0	0	0	0
Vegetables	0.17	0.26	0.02	0.02	0
**Norway**					
Cereals	0.02	0.13	0.09	0.17	0.02
Fiber	0	0	0	0	0
Fruit	0	0	0	0	0
Livestock	0	0.03	0.02	0.05	0.01
Oil crops	0	0.01	0.01	0.02	0
Pulses	0	0	0	0	0
Roots & tubers	0	0.01	0.01	0.02	0
Sugar crops	0	0	0	0	0
Vegetables	0	0	0	0	0
**Oman**					
Cereals	0	0	0	0	0
Fiber	0	0	0	0	0
Fruit	0	0.02	0.01	0	0
Livestock	0	0.01	0	0	0
Oil crops	0	0	0	0	0
Pulses	0	0	0	0	0

Roots & tubers	0	0	0	0	0
Sugar crops	0	0	0	0	0
Vegetables	0	0	0	0	0
**Pakistan**					
Cereals	2.98	7.01	0.89	0.17	0
Fiber	3.6	9.36	1.22	0.24	0
Fruit	0.11	0.27	0.03	0.01	0
Livestock	0.63	1.08	0.12	0.02	0
Oil crops	0.5	1.14	0.14	0.03	0
Pulses	0.24	0.54	0.07	0.01	0
Roots & tubers	0.07	0.15	0.02	0	0
Sugar crops	0.02	0.05	0.01	0	0
Vegetables	0.1	0.25	0.03	0.01	0
**Panama**					
Cereals	0	0.01	0.01	0.01	0
Fiber	0	0	0	0	0
Fruit	0.01	0.03	0.03	0.02	0.01
Livestock	0.01	0.01	0.01	0	0
Oil crops	0	0	0	0	0
Pulses	0	0.01	0.01	0	0
Roots & tubers	0	0	0	0	0
Sugar crops	0	0	0	0	0
Vegetables	0	0	0	0	0
**Papua New Guinea**					
Cereals	0	0	0	0	0
Fiber	0	0	0	0	0
Fruit	0.04	0.05	0.02	0.01	0
Livestock	0	0	0	0	0
Oil crops	0.04	0.05	0.02	0.01	0
Pulses	0	0	0	0	0
Roots & tubers	0.04	0.06	0.02	0.01	0
Sugar crops	0	0	0	0	0
Vegetables	0	0	0	0	0
**Paraguay**					
Cereals	0	0.03	0.04	0.09	0.47
Fiber	0	0.04	0.04	0.08	0.21
Fruit	0	0	0	0.01	0.06
Livestock	0	0.01	0.01	0.02	0.04
Oil crops	0.01	0.28	0.45	2.24	14.71
Pulses	0	0.04	0.04	0.06	0.14
Roots & tubers	0	0.04	0.05	0.16	0.84
Sugar crops	0	0	0	0	0
Vegetables	0	0	0	0	0.01
**Peru**					
Cereals	0.01	0.12	0.11	0.14	0.18
Fiber	0.01	0.11	0.1	0.13	0.16

Fruit	0	0.07	0.07	0.09	0.12
Livestock	0	0.06	0.05	0.05	0.04
Oil crops	0	0	0	0	0
Pulses	0.01	0.11	0.1	0.12	0.09
Roots & tubers	0.01	0.13	0.12	0.16	0.24
Sugar crops	0	0	0	0	0
Vegetables	0	0.03	0.03	0.03	0.03
**Philippines**					
Cereals	0.39	1.08	0.09	0.84	0
Fiber	0	0	0	0	0
Fruit	0.21	0.54	0.04	0.27	0
Livestock	0.08	0.17	0.01	0.01	0
Oil crops	0.4	1.02	0.08	0.51	0
Pulses	0.02	0.06	0	0.03	0
Roots & tubers	0.11	0.29	0.02	0.04	0
Sugar crops	0	0	0	0	0
Vegetables	0.01	0.02	0	0.01	0
**Poland**					
Cereals	0.42	1.67	0.75	2.52	0.97
Fiber	0	0	0	0	0
Fruit	0	0.02	0.01	0.02	0.01
Livestock	0.09	0.32	0.13	0.32	0.1
Oil crops	0.13	0.87	0.65	3.36	1.52
Pulses	0.03	0.1	0.04	0.1	0.03
Roots & tubers	0.15	0.5	0.16	0.34	0.09
Sugar crops	0.45	2.1	1.18	4.55	1.82
Vegetables	0.02	0.07	0.03	0.07	0.02
**Portugal**					
Cereals	0.02	0.06	0.03	0.1	0.04
Fiber	0	0	0	0	0
Fruit	0.01	0.02	0.01	0.03	0.01
Livestock	0.02	0.08	0.02	0.05	0.01
Oil crops	0	0	0	0.01	0
Pulses	0	0	0	0.01	0
Roots & tubers	0.01	0.03	0.01	0.02	0
Sugar crops	0	0.04	0.04	0.17	0.07
Vegetables	0.01	0.04	0.02	0.07	0.03
**Qatar**					
Cereals	0	0	0	0	0
Fiber	0	0	0	0	0
Fruit	0	0	0	0	0
Livestock	0	0	0	0	0
Oil crops	0	0	0	0	0
Pulses	0	0	0	0	0
Roots & tubers	0	0	0	0	0
Sugar crops	0	0	0	0	0

Vegetables	0	0	0	0	0
**Republic of Korea**					
Cereals	0.23	0.32	0.01	0	0
Fiber	0	0	0	0	0
Fruit	0.01	0.01	0	0	0
Livestock	0.17	0.23	0.01	0	0
Oil crops	0.25	0.33	0.01	0	0
Pulses	0.01	0.02	0	0	0
Roots & tubers	0.04	0.06	0	0	0
Sugar crops	0	0	0	0	0
Vegetables	0.1	0.14	0	0	0
**Romania**					
Cereals	0.19	0.86	0.48	1.85	0.73
Fiber	0	0	0	0	0
Fruit	0	0.01	0	0.01	0.01
Livestock	0.03	0.13	0.06	0.2	0.07
Oil crops	0.11	0.55	0.35	1.57	0.66
Pulses	0.02	0.06	0.03	0.08	0.03
Roots & tubers	0.04	0.15	0.07	0.16	0.04
Sugar crops	0.06	0.22	0.09	0.27	0.1
Vegetables	0.01	0.04	0.02	0.07	0.03
**Russian Federation**					
Cereals	1.25	5.32	2.71	13.25	6.2
Fiber	0	0	0	0	0
Fruit	0	0.01	0	0.03	0.01
Livestock	0.15	0.63	0.31	1.13	0.45
Oil crops	0.41	1.91	1.09	5.93	2.87
Pulses	0.15	0.64	0.32	1.28	0.55
Roots & tubers	0.31	1.23	0.54	1.75	0.67
Sugar crops	1.08	4.08	1.77	10.38	5.33
Vegetables	0.03	0.12	0.06	0.25	0.11
**Rwanda**					
Cereals	0.03	0.04	0	0	0
Fiber	0	0	0	0	0
Fruit	0.14	0.22	0.02	0	0
Livestock	0	0.01	0	0	0
Oil crops	0.05	0.06	0	0	0
Pulses	0.41	0.65	0.06	0	0
Roots & tubers	0.15	0.22	0.02	0	0
Sugar crops	0	0	0	0	0
Vegetables	0	0	0	0	0
**Saint Lucia**					
Cereals	0	0	0	0	0
Fiber	0	0	0	0	0
Fruit	0	0	0	0	0
Livestock	0	0	0	0	0

Oil crops	0	0	0	0	0
Pulses	0	0	0	0	0
Roots & tubers	0	0	0	0	0
Sugar crops	0	0	0	0	0
Vegetables	0	0	0	0	0
**Saint Vincent and the Grenadines**					
Cereals	0	0	0	0	0
Fiber	0	0	0	0	0
Fruit	0	0	0	0	0
Livestock	0	0	0	0	0
Oil crops	0	0	0	0	0
Pulses	0	0	0	0	0
Roots & tubers	0	0	0	0	0
Sugar crops	0	0	0	0	0
Vegetables	0	0	0	0	0
**Saudi Arabia**					
Cereals	0.18	0.68	0.22	0.11	0.02
Fiber	0	0	0	0	0
Fruit	0.02	0.08	0.02	0.01	0
Livestock	0.02	0.09	0.03	0.02	0
Oil crops	0	0	0	0	0
Pulses	0	0	0	0	0
Roots & tubers	0.01	0.03	0.01	0	0
Sugar crops	0	0	0	0	0
Vegetables	0.01	0.04	0.01	0.01	0
**Senegal**					
Cereals	0.15	0.3	0.03	0	0
Fiber	0.02	0.08	0.01	0	0
Fruit	0	0.01	0	0	0
Livestock	0.01	0.01	0	0	0
Oil crops	0.01	0.01	0	0	0
Pulses	0	0	0	0	0
Roots & tubers	0.03	0.03	0	0	0
Sugar crops	0	0	0	0	0
Vegetables	0.01	0.02	0	0	0
**Serbia**					
Cereals	0.17	0.61	0.24	0.63	0.21
Fiber	0	0	0	0	0
Fruit	0	0.01	0	0	0
Oil crops	0.14	0.56	0.24	0.69	0.23
Pulses	0.03	0.09	0.03	0.07	0.02
Roots & tubers	0.01	0.03	0.01	0.04	0.01
Sugar crops	0.34	1.08	0.3	0.71	0.24
Vegetables	0	0.02	0.01	0.01	0
**Sierra Leone**					

Cereals	0.03	0.06	0.01	0	0
Fiber	0	0	0	0	0
Fruit	0	0	0	0	0
Livestock	0	0	0	0	0
Oil crops	0	0	0	0	0
Pulses	0.01	0.03	0	0	0
Roots & tubers	0.09	0.22	0.03	0	0
Sugar crops	0	0	0	0	0
Vegetables	0	0	0	0	0
**Slovakia**					
Cereals	0.07	0.28	0.12	0.36	0.13
Fiber	0	0	0	0	0
Fruit	0	0	0	0	0
Livestock	0	0.02	0.01	0.04	0.01
Oil crops	0.1	0.38	0.16	0.49	0.17
Pulses	0.01	0.02	0.01	0.03	0.01
Roots & tubers	0	0.01	0	0.01	0
Sugar crops	0.07	0.29	0.15	0.45	0.15
Vegetables	0	0	0	0	0
**Slovenia**					
Cereals	0.02	0.07	0.02	0.02	0
Fiber	0	0	0	0	0
Fruit	0	0	0	0	0
Livestock	0.01	0.02	0.01	0.01	0
Oil crops	0.01	0.02	0.01	0.01	0
Pulses	0	0.01	0	0	0
Roots & tubers	0	0.01	0	0	0
Sugar crops	0.02	0.08	0.02	0.02	0
Vegetables	0	0	0	0	0
**Somalia**					
Cereals	0.02	0.03	0	0	0
Fiber	0.01	0.01	0	0	0
Fruit	0	0.01	0	0	0
Livestock	0.02	0.05	0	0	0
Oil crops	0.02	0.03	0	0	0
Pulses	0.03	0.04	0	0	0
Roots & tubers	0.01	0.01	0	0	0
Sugar crops	0	0	0	0	0
Vegetables	0	0	0	0	0
**South Africa**					
Cereals	0.13	0.77	0.13	1.59	0
Fiber	0.01	0.05	0.01	0.03	0
Fruit	0.09	0.22	0.03	0.09	0
Livestock	0.05	0.15	0.02	0.19	0
Oil crops	0.23	0.78	0.11	0.6	0
Pulses	0.01	0.09	0.02	0.12	0

Roots & tubers	0.03	0.11	0.02	0.08	0
Sugar crops	0	0	0	0	0
Vegetables	0.02	0.07	0.01	0.04	0
**Spain**					
Cereals	0.27	1.23	0.66	2.24	0.83
Fiber	0.01	0.05	0.04	0.25	0.13
Fruit	0.05	0.21	0.09	0.32	0.13
Livestock	0.1	0.34	0.13	0.26	0.06
Oil crops	0.03	0.14	0.08	0.37	0.17
Pulses	0.03	0.12	0.06	0.21	0.08
Roots & tubers	0.02	0.09	0.04	0.12	0.04
Sugar crops	0.13	0.79	0.57	2.5	1.06
Vegetables	0.05	0.19	0.09	0.32	0.13
**Sri Lanka**					
Cereals	0.07	0.14	0.02	0.05	0
Fiber	0	0	0	0	0
Fruit	0.02	0.04	0	0.02	0
Livestock	0.01	0.02	0	0	0
Oil crops	0.08	0.15	0.02	0.05	0
Pulses	0.01	0.02	0	0.01	0
Roots & tubers	0.01	0.03	0	0.01	0
Sugar crops	0	0	0	0	0
Vegetables	0.01	0.02	0	0.01	0
**Sudan (former)**					
Cereals	0.16	0.75	0.26	0.3	0.4
Fiber	0.07	0.26	0.08	0.09	0.13
Fruit	0.02	0.06	0.02	0.02	0.03
Livestock	0.05	0.2	0.06	0.05	0.06
Oil crops	0.05	0.16	0.04	0.04	0.06
Pulses	0.01	0.05	0.01	0.02	0.02
Roots & tubers	0.01	0.03	0.01	0.01	0.02
Sugar crops	0	0	0	0	0
Vegetables	0.03	0.12	0.03	0.04	0.05
**Suriname**					
Cereals	0	0	0	0	0.01
Fiber	0	0	0	0	0
Fruit	0	0	0	0	0.01
Livestock	0	0	0	0	0
Oil crops	0	0	0	0	0
Pulses	0	0	0	0	0
Roots & tubers	0	0	0	0	0
Sugar crops	0	0	0	0	0
Vegetables	0	0	0	0	0
**Swaziland**					
Cereals	0	0.01	0	0	0
Fiber	0	0	0	0	0

Fruit	0	0.01	0	0	0
Livestock	0	0	0	0	0
Oil crops	0	0	0	0	0
Pulses	0	0	0	0	0
Roots & tubers	0	0	0	0	0
Sugar crops	0	0	0	0	0
Vegetables	0	0	0	0	0
**Sweden**					
Cereals	0.04	0.35	0.27	0.77	0.23
Fiber	0	0	0	0	0
Fruit	0	0	0	0	0
Livestock	0.01	0.04	0.03	0.1	0.03
Oil crops	0.02	0.18	0.13	0.36	0.1
Pulses	0	0.03	0.02	0.07	0.02
Roots & tubers	0	0.03	0.02	0.04	0.01
Sugar crops	0.08	0.55	0.37	0.75	0.13
Vegetables	0	0	0	0	0
**Switzerland**					
Cereals	0.02	0.09	0.06	0.13	0.02
Fiber	0	0	0	0	0
Fruit	0	0	0	0	0
Livestock	0.01	0.06	0.05	0.1	0.01
Oil crops	0.02	0.09	0.04	0.08	0.01
Pulses	0	0.01	0.01	0.02	0
Roots & tubers	0	0.01	0.01	0.03	0
Sugar crops	0.03	0.28	0.25	0.56	0.07
Vegetables	0	0	0	0	0
**Syrian Arab Republic**					
Cereals	0.41	1.18	0.28	0.17	0.12
Fiber	0.28	0.94	0.27	0.2	0.19
Fruit	0.02	0.07	0.02	0.01	0.01
Livestock	0.04	0.12	0.03	0.02	0.01
Oil crops	0.01	0.02	0.01	0	0
Pulses	0	0.02	0	0	0
Roots & tubers	0.01	0.04	0.01	0.01	0.01
Sugar crops	0.2	0.6	0.15	0.09	0.05
Vegetables	0.02	0.08	0.02	0.02	0.02
**Tajikistan**					
Cereals	0.03	0.15	0.05	0.04	0.04
Fiber	0.11	0.47	0.16	0.14	0.15
Fruit	0	0	0	0	0
Livestock	0	0.01	0	0.01	0.01
Oil crops	0	0	0	0	0
Pulses	0	0.02	0.01	0.01	0
Roots & tubers	0.01	0.03	0.01	0.01	0.01
Sugar crops	0	0	0	0	0

Vegetables	0.01	0.03	0.01	0.01	0.01
**Thailand**					
Cereals	0.77	2.26	0.19	0.08	0
Fiber	0.01	0.02	0	0	0
Fruit	0.15	0.38	0.03	0.01	0
Livestock	0.07	0.34	0.04	0.02	0
Oil crops	0.25	0.78	0.07	0.03	0
Pulses	0.16	0.31	0.02	0.01	0
Roots & tubers	0.96	3.58	0.34	0.15	0
Sugar crops	0	0	0	0	0
Vegetables	0.01	0.02	0	0	0
**The former Yugoslav Republic of Macedonia**					
Cereals	0.01	0.06	0.03	0.07	0.01
Fiber	0	0	0	0	0
Fruit	0	0	0	0	0
Livestock	0	0.01	0	0.01	0
Oil crops	0	0	0	0	0
Pulses	0	0.01	0.01	0.01	0
Roots & tubers	0	0.01	0	0.01	0
Sugar crops	0	0.01	0	0.01	0
Vegetables	0	0.01	0	0.01	0
**Togo**					
Cereals	0.06	0.11	0.01	0	0
Fiber	0.05	0.06	0	0	0
Fruit	0	0	0	0	0
Livestock	0	0	0	0	0
Oil crops	0	0	0	0	0
Pulses	0.08	0.15	0.02	0	0
Roots & tubers	0.1	0.17	0.02	0	0
Sugar crops	0	0	0	0	0
Vegetables	0	0	0	0	0
**Trinidad and Tobago**					
Cereals	0	0	0	0	0
Fiber	0	0	0	0	0
Fruit	0	0	0	0	0
Livestock	0	0	0	0	0
Oil crops	0	0	0	0	0
Pulses	0	0	0	0	0
Roots & tubers	0	0	0	0	0
Sugar crops	0	0	0	0	0
Vegetables	0	0	0	0	0
**Tunisia**					
Cereals	0.08	0.32	0.1	0.1	0.12
Fiber	0	0	0	0	0
Fruit	0.01	0.02	0.01	0.01	0.01

Livestock	0.01	0.05	0.02	0.01	0.01
Oil crops	0	0.01	0	0	0
Pulses	0	0.02	0.01	0.01	0.01
Roots & tubers	0	0.02	0	0.01	0.01
Sugar crops	0	0	0	0	0
Vegetables	0.01	0.04	0.02	0.03	0.05
**Turkey**					
Cereals	1.68	5.39	1.42	1.24	1.38
Fiber	0.74	2.74	0.85	0.62	0.54
Fruit	0.08	0.25	0.06	0.05	0.05
Livestock	0.18	0.54	0.13	0.08	0.05
Oil crops	0.19	0.78	0.25	0.14	0.05
Pulses	0.13	0.41	0.11	0.07	0.05
Roots & tubers	0.12	0.28	0.05	0.03	0.03
Sugar crops	2.45	6.56	1.35	0.84	0.56
Vegetables	0.29	0.86	0.21	0.17	0.16
**Turkmenistan**					
Cereals	0.03	0.44	0.21	0.26	0.35
Fiber	0.04	0.7	0.33	0.41	0.57
Fruit	0	0	0	0	0
Livestock	0	0.04	0.02	0.03	0.04
Oil crops	0	0	0	0	0
Pulses	0	0.01	0.01	0.01	0.01
Roots & tubers	0	0.01	0	0	0.01
Sugar crops	0	0.07	0.03	0.04	0.05
Vegetables	0	0.02	0.01	0.01	0.02
**Uganda**					
Cereals	0.31	0.57	0.06	0	0
Fiber	0.04	0.09	0.01	0	0
Fruit	0.45	0.84	0.09	0	0
Livestock	0.02	0.05	0.01	0	0
Oil crops	0.38	0.58	0.05	0	0
Pulses	0.6	1.08	0.11	0	0
Roots & tubers	0.47	0.89	0.1	0	0
Sugar crops	0	0	0	0	0
Vegetables	0.01	0.02	0	0	0
**Ukraine**					
Cereals	0.78	3.02	1.3	3.95	1.41
Fiber	0	0	0	0	0
Fruit	0	0.01	0	0.01	0
Livestock	0.09	0.33	0.14	0.38	0.12
Oil crops	0.78	3.05	1.33	4.26	1.59
Pulses	0.07	0.34	0.19	0.69	0.25
Roots & tubers	0.15	0.65	0.33	0.91	0.28
Sugar crops	0.78	3.52	1.87	6.63	2.55
Vegetables	0.02	0.09	0.04	0.13	0.05

**United Arab Emirates**					
Cereals	0	0	0	0	0
Fiber	0	0	0	0	0
Fruit	0.01	0.06	0.02	0.01	0
Livestock	0	0.01	0	0	0
Oil crops	0	0	0	0	0
Pulses	0	0	0	0	0
Roots & tubers	0	0	0	0	0
Sugar crops	0	0	0	0	0
Vegetables	0	0.01	0	0	0
**United Kingdom**					
Cereals	0.37	1.84	1.06	3.21	1.05
Fiber	0	0	0	0	0
Fruit	0	0	0	0	0
Livestock	0.06	0.33	0.2	0.44	0.09
Oil crops	0.38	1.87	1.06	3.09	0.97
Pulses	0.02	0.14	0.11	0.35	0.12
Roots & tubers	0.01	0.12	0.1	0.34	0.12
Sugar crops	0.11	1.04	0.86	3.2	1.18
Vegetables	0	0.01	0.01	0.03	0.01
**United Republic of Tanzania**					
Cereals	0.3	0.79	0.1	0	0
Fiber	0.13	0.36	0.05	0	0
Fruit	0.12	0.33	0.04	0	0
Livestock	0.03	0.07	0.01	0	0
Oil crops	0.12	0.31	0.04	0	0
Pulses	0.98	1.87	0.2	0	0
Roots & tubers	0.33	1	0.14	0	0
Sugar crops	0	0	0	0	0
Vegetables	0.01	0.02	0	0	0
**United States of America**					
Cereals	0.01	3.64	7.79	36.43	36.61
Fiber	0	0.96	2.27	11.51	12.2
Fruit	0	0.17	0.23	0.7	0.92
Livestock	0	0.82	1.28	3.46	2.32
Oil crops	0.02	12.03	27.76	133.6	134.96
Pulses	0	0.33	0.66	2.8	2.56
Roots & tubers	0	0.21	0.36	1.17	0.69
Sugar crops	0	0.68	1.87	10.88	12.7
Vegetables	0	0.13	0.27	1.06	0.87
**Uruguay**					
Cereals	0	0.02	0.03	0.1	0.36
Fiber	0	0	0	0	0
Fruit	0	0	0	0.01	0.03
Livestock	0	0	0.01	0.02	0.1

Oil crops	0	0.03	0.04	0.27	2.28
Pulses	0	0	0	0	0.01
Roots & tubers	0	0	0	0	0.02
Sugar crops	0	0	0	0	0
Vegetables	0	0	0	0	0.01
**Uzbekistan**					
Cereals	0.2	1.18	0.46	0.42	0.47
Fiber	0.62	3.71	1.44	1.33	1.47
Fruit	0	0.01	0	0	0
Livestock	0.02	0.16	0.06	0.04	0.03
Oil crops	0	0.01	0	0	0
Pulses	0	0	0	0	0
Roots & tubers	0.01	0.05	0.02	0.02	0.02
Sugar crops	0	0	0	0	0
Vegetables	0.02	0.13	0.05	0.05	0.05
**Venezuela (Bolivarian Republic of)**					
Cereals	0	0.05	0.05	0.12	0.41
Fiber	0	0	0	0.01	0.04
Fruit	0	0.01	0.02	0.04	0.16
Livestock	0	0.03	0.03	0.04	0.1
Oil crops	0	0.01	0.01	0.03	0.11
Pulses	0	0.01	0.01	0.03	0.07
Roots & tubers	0	0.01	0.01	0.03	0.12
Sugar crops	0	0	0	0	0.01
Vegetables	0	0.01	0.01	0.01	0.05
**Viet Nam**					
Cereals	0.65	1.96	0.17	0.86	0
Fiber	0.01	0.03	0	0.01	0
Fruit	0.09	0.21	0.02	0.05	0
Livestock	0.07	0.11	0	0.03	0
Oil crops	0.3	0.73	0.06	0.11	0
Pulses	0.2	0.44	0.03	0.04	0
Roots & tubers	0.64	1.12	0.06	0.05	0
Sugar crops	0	0	0	0	0
Vegetables	0.01	0.03	0	0.01	0
**Yemen**					
Cereals	0.03	0.14	0.05	0.02	0
Fiber	0.01	0.03	0.01	0	0
Fruit	0.01	0.03	0.01	0	0
Livestock	0.01	0.03	0.01	0	0
Oil crops	0	0.01	0	0	0
Pulses	0	0.01	0	0	0
Roots & tubers	0	0.02	0.01	0	0
Sugar crops	0	0	0	0	0
Vegetables	0.01	0.03	0.01	0.01	0

**Zambia**					
Cereals	0.07	0.18	0.02	0.04	0
Fiber	0.04	0.1	0.01	0.16	0
Fruit	0	0	0	0	0
Livestock	0.01	0.01	0	0	0
Oil crops	0.05	0.17	0.02	0.02	0
Pulses	0	0.01	0	0	0
Roots & tubers	0.1	0.14	0.01	0.01	0
Sugar crops	0	0	0	0	0
Vegetables	0	0	0	0	0
**Zimbabwe**					
Cereals	0.06	0.25	0.04	0.02	0
Fiber	0.06	0.35	0.06	0.03	0
Fruit	0	0.03	0	0	0
Livestock	0.01	0.02	0	0	0
Oil crops	0.09	0.2	0.02	0.01	0
Pulses	0.02	0.08	0.01	0	0
Roots & tubers	0.02	0.03	0	0	0
Sugar crops	0	0	0	0	0
Vegetables	0	0	0	0	0

	**Iron production (T)**				
	**< 2 ha**	**2 - 20 ha**	**20 - 50 ha**	**50 - 200 ha**	**> 200 ha**
**Afghanistan**					
Cereals	35.15	86.1	11.13	36.5	0
Fiber	0.05	1.45	0.26	0.05	0
Fruit	0.13	0.96	0.16	0.03	0
Livestock	2.37	3.04	0.28	0.05	0
Oil crops	2.13	2.71	0.24	0.04	0
Pulses	0.1	0.21	0.03	0.05	0
Roots & tubers	0.45	0.99	0.12	0.19	0
Sugar crops	0.02	0.04	0	0	0
Vegetables	0	0	0	0	0
**Albania**					
Cereals	2.82	8.8	2.19	2.14	0.1
Fiber	0	0.02	0.01	0.02	0
Fruit	0.06	0.22	0.09	0.11	0.01
Livestock	0.5	1.4	0.24	0.14	0.01
Oil crops	0.13	0.48	0.17	0.21	0.01
Pulses	0.26	0.77	0.16	0.12	0.01
Roots & tubers	0.15	0.49	0.14	0.16	0.01
Sugar crops	0.05	0.15	0.04	0.04	0
Vegetables	0.07	0.25	0.08	0.09	0
**Algeria**					
Cereals	15.08	56.1	16.63	19.06	25.85
Fiber	0	0	0	0	0

Fruit	0.81	3.26	1.09	0.58	0.2
Livestock	1.42	5.74	1.81	1.39	1.22
Oil crops	1.46	4.51	1.13	1.69	2.71
Pulses	0.04	0.13	0.04	0.05	0.07
Roots & tubers	1.62	5.24	1.38	1.4	1.76
Sugar crops	0	0	0	0	0
Vegetables	0.42	1.74	0.56	0.38	0.25
**Angola**					
Cereals	6.58	13.24	1.48	0.84	0
Fiber	0.05	0.12	0.01	0.02	0
Fruit	0.62	1.18	0.13	0.09	0
Livestock	0.76	1.19	0.1	0.08	0
Oil crops	0.45	0.71	0.06	0.04	0
Pulses	1.48	3.4	0.42	0.42	0
Roots & tubers	6.97	15.97	1.96	1.44	0
Sugar crops	0.06	0.13	0.02	0.01	0
Vegetables	0.01	0.02	0	0	0
**Argentina**					
Cereals	0.56	14.91	24.86	138.4	995.79
Fiber	0.07	1.21	1.31	3.59	19.23
Fruit	0	0.11	0.25	1.58	10.47
Livestock	0.2	3.55	4.2	11.7	45.3
Oil crops	2.53	70.19	119.62	732.61	5795.23
Pulses	0.02	0.54	0.87	3.39	13.16
Roots & tubers	0.03	0.62	0.89	2.94	8.54
Sugar crops	0.03	0.65	1	3.33	7.46
Vegetables	0	0.06	0.09	0.43	2.47
**Armenia**					
Cereals	0.94	6.88	2.84	1.5	0.26
Fiber	0	0	0	0	0
Fruit	0.06	0.47	0.19	0.1	0.02
Livestock	0.17	0.88	0.33	0.17	0.03
Oil crops	0	0	0	0	0
Pulses	0.02	0.14	0.06	0.03	0.01
Roots & tubers	0.25	1.82	0.75	0.4	0.07
Sugar crops	0.01	0.07	0.03	0.01	0
Vegetables	0.06	0.46	0.19	0.1	0.02
**Australia**					
Cereals	0	1.68	7.21	67.1	891.57
Fiber	0	0.08	0.36	3.53	62.41
Fruit	0	0.01	0.05	0.49	7.09
Livestock	0	0.14	0.58	5.24	53.21
Oil crops	0	0.4	1.7	15.33	161.4
Pulses	0	0.02	0.09	0.89	17.21
Roots & tubers	0	0.02	0.08	0.75	6.66
Sugar crops	0	0.02	0.15	1.34	17.04

Vegetables	0	0	0.01	0.12	1.26
**Austria**					
Cereals	12.46	51.28	24.1	43.8	5.21
Fiber	0	0	0	0	0
Fruit	0.2	0.71	0.26	0.41	0.04
Livestock	0.57	3.17	2.01	3.95	0.47
Oil crops	3.29	12.57	5.31	9.3	1.11
Pulses	0.36	1.45	0.66	1.19	0.14
Roots & tubers	0.5	1.78	0.64	1.02	0.11
Sugar crops	1.99	7.16	2.71	4.51	0.53
Vegetables	0.03	0.11	0.05	0.09	0.01
**Azerbaijan**					
Cereals	10.75	34.55	9.3	7.39	7.42
Fiber	1.19	3.82	1.03	0.82	0.82
Fruit	0.08	0.26	0.07	0.05	0.05
Livestock	0.5	1.75	0.51	0.43	0.46
Oil crops	0.07	0.23	0.06	0.05	0.05
Pulses	0.11	0.49	0.17	0.11	0.07
Roots & tubers	0.95	3.03	0.81	0.64	0.65
Sugar crops	0.11	0.36	0.1	0.08	0.07
Vegetables	0.23	0.76	0.21	0.13	0.09
**Bangladesh**					
Cereals	94.54	205.91	25.34	49.26	0
Fiber	0.71	1.6	0.2	0.22	0
Fruit	0.47	1.05	0.13	0.3	0
Livestock	3.91	7.45	0.84	0.98	0
Oil crops	8.69	25.41	3.51	5.79	0
Pulses	0.91	2.08	0.26	0.57	0
Roots & tubers	7.64	15.98	1.93	5.82	0
Sugar crops	0.74	1.55	0.19	0.47	0
Vegetables	0.44	0.96	0.12	0.22	0
**Belarus**					
Cereals	14.04	53.35	22.24	64.36	22.5
Fiber	0	0	0	0	0
Fruit	0.01	0.07	0.05	0.18	0.06
Livestock	0.64	2.99	1.64	5.36	1.92
Oil crops	2.16	8.19	3.42	9.8	3.37
Pulses	0.59	2.26	0.94	2.7	0.93
Roots & tubers	0.9	8.12	6.59	24.51	9.13
Sugar crops	0.36	3.42	2.84	11.05	4.24
Vegetables	0.07	0.29	0.13	0.34	0.11
**Belgium**					
Cereals	6.76	25.19	10.35	34.05	13.16
Fiber	0	0	0	0	0
Fruit	0.03	0.12	0.05	0.13	0.04
Livestock	2.02	7.03	2.42	4.94	1.31

Oil crops	0.01	0.28	0.32	2.94	1.61
Pulses	0.01	0.06	0.03	0.08	0.02
Roots & tubers	2.2	7.34	2.33	4.01	0.81
Sugar crops	2.17	9.13	4.45	14.59	5.38
Vegetables	0.06	0.22	0.09	0.23	0.08
**Belize**					
Cereals	0.03	0.23	0.6	0.26	0.12
Fiber	0	0	0	0	0
Fruit	0.01	0.07	0.18	0.08	0.03
Livestock	0.01	0.02	0.04	0.02	0.02
Oil crops	0	0.02	0.04	0.02	0.01
Pulses	0	0.05	0.12	0.06	0.03
Roots & tubers	0	0	0	0	0
Sugar crops	0.01	0.1	0.26	0.12	0.06
Vegetables	0	0	0	0	0
**Benin**					
Cereals	9.93	15.91	1.48	0.01	0
Fiber	3.03	9.65	1.38	0.01	0
Fruit	0.07	0.12	0.01	0	0
Livestock	0.28	0.44	0.04	0	0
Oil crops	1.18	1.77	0.15	0	0
Pulses	2.18	3.75	0.37	0	0
Roots & tubers	7.02	8.79	0.55	0	0
Sugar crops	0.01	0.01	0	0	0
Vegetables	0.13	0.2	0.02	0	0
**Bhutan**					
Cereals	1.39	2.04	0.19	0.04	0
Fiber	0	0	0	0	0
Fruit	0.02	0.02	0	0	0
Livestock	0.04	0.06	0.01	0	0
Oil crops	0.09	0.13	0.01	0	0
Pulses	0.15	0.18	0.02	0	0
Roots & tubers	0.14	0.19	0.02	0	0
Sugar crops	0	0	0	0	0
Vegetables	0	0	0	0	0
**Bolivia (Plurinational State of)**					
Cereals	0.2	3.34	3.57	7.96	26.92
Fiber	0.02	0.29	0.32	0.89	4.32
Fruit	0.04	0.54	0.48	0.53	0.65
Livestock	0.05	0.79	0.82	1.6	3.45
Oil crops	0.21	4.9	7.57	35.23	212.47
Pulses	0.02	0.31	0.29	0.47	1.26
Roots & tubers	0.08	1.18	1.09	1.38	1.54
Sugar crops	0	0.09	0.12	0.43	2.31
Vegetables	0	0.03	0.03	0.06	0.24

**Bosnia and Herzegovina**					
Cereals	3.69	14.05	6.02	11	1.41
Fiber	0	0	0	0	0
Fruit	0.01	0.05	0.02	0.05	0.01
Livestock	0.1	0.41	0.21	0.4	0.05
Oil crops	0.17	0.73	0.37	0.75	0.1
Pulses	0.1	0.38	0.16	0.3	0.04
Roots & tubers	0.25	0.96	0.4	0.74	0.09
Sugar crops	0	0	0	0	0
Vegetables	0.02	0.06	0.03	0.05	0.01
**Botswana**					
Cereals	0.13	0.62	0.1	0.32	0
Fiber	0.02	0.08	0.01	0.02	0
Fruit	0	0	0	0	0
Livestock	0.08	0.42	0.07	0.24	0
Oil crops	0.02	0.07	0.01	0.04	0
Pulses	0.01	0.03	0.01	0.03	0
Roots & tubers	0	0	0	0	0
Sugar crops	0	0	0	0	0
Vegetables	0	0	0	0	0
**Brazil**					
Cereals	3.18	55.73	63	194.63	1139.9
Fiber	0.05	1.59	2.89	19.9	167.79
Fruit	0.27	4.19	4.01	6.55	18.99
Livestock	1.3	21.19	21.55	43.56	153.21
Oil crops	2.55	69.65	117.63	828.43	7482.5
Pulses	1.76	27.49	25.57	37.04	93.21
Roots & tubers	0.66	10.51	10.24	17.17	43.8
Sugar crops	0.12	3.38	5.87	31.17	201.14
Vegetables	0.06	0.98	1	1.99	6.79
**Brunei Darussalam**					
Cereals	0	0.01	0	0	0
Fiber	0	0	0	0	0
Fruit	0	0	0	0	0
Livestock	0	0.18	0.03	0.01	0
Oil crops	0	0	0	0	0
Pulses	0	0	0	0	0
Roots & tubers	0	0.01	0	0	0
Sugar crops	0	0	0	0	0
Vegetables	0	0	0	0	0
**Bulgaria**					
Cereals	5.76	32.27	20.94	74.2	27.15
Fiber	0.01	0.04	0	0	0
Fruit	0.13	0.43	0.15	0.33	0.09
Livestock	0.36	1.46	0.66	1.47	0.36
Oil crops	0.7	5.12	3.91	16.54	6.75

Pulses	0.03	0.13	0.07	0.23	0.08
Roots & tubers	0.15	0.65	0.35	0.78	0.13
Sugar crops	0	0.02	0.02	0.07	0.02
Vegetables	0.03	0.11	0.05	0.17	0.06
**Burkina Faso**					
Cereals	27.78	55.37	6.14	15.84	0
Fiber	8.75	16.21	1.77	6.58	0
Fruit	0	0	0	0	0
Livestock	1.43	1.99	0.15	0.33	0
Oil crops	0.58	1.71	0.24	1.66	0
Pulses	0.09	0.12	0.01	0.03	0
Roots & tubers	0.09	0.2	0.02	0.11	0
Sugar crops	0.02	0.08	0.01	0.12	0
Vegetables	0.01	0.01	0	0	0
**Burundi**					
Cereals	3.26	3.93	0.2	0	0
Fiber	0.12	0.14	0.01	0	0
Fruit	1.21	1.45	0.08	0	0
Livestock	0.16	0.19	0.01	0	0
Oil crops	0.19	0.23	0.01	0	0
Pulses	6.02	7.25	0.37	0	0
Roots & tubers	2.34	2.82	0.15	0	0
Sugar crops	0.03	0.03	0	0	0
Vegetables	0	0	0	0	0
**Cambodia**					
Cereals	17.91	39.25	2.57	1.04	0
Fiber	0	0.01	0	0	0
Fruit	0.09	0.19	0.01	0	0
Livestock	0.78	1.4	0.07	0.02	0
Oil crops	10.44	17.48	0.89	0.34	0
Pulses	1.09	1.84	0.09	0.03	0
Roots & tubers	1.34	2.39	0.13	0.05	0
Sugar crops	0.03	0.05	0	0	0
Vegetables	0	0	0	0	0
**Cameroon**					
Cereals	22.8	34.74	2.96	2.36	0
Fiber	5.45	9.05	0.86	0.3	0
Fruit	3.57	5.61	0.49	0.6	0
Livestock	1.27	1.56	0.1	0	0
Oil crops	0.87	1.39	0.13	0.05	0
Pulses	6.24	7.57	0.43	0	0
Roots & tubers	2.7	4.68	0.46	1.51	0
Sugar crops	0.2	0.3	0.02	0.07	0
Vegetables	0.55	0.81	0.06	0.03	0
**Canada**					
Cereals	0.56	130.19	206.38	653.95	689.59

Fiber	0	0	0	0	0
Fruit	0	0.05	0.1	0.33	0.18
Livestock	0.02	4.6	8.22	22.71	13.45
Oil crops	0.79	178.88	273.92	760.93	746.27
Pulses	0.05	12.11	18.76	58.78	64.57
Roots & tubers	0.01	3.33	5.46	12.16	7.36
Sugar crops	0	0.13	0.21	1.58	2.69
Vegetables	0	0.19	0.4	1.17	0.65
**Central African Republic**					
Cereals	2.4	3.19	0.22	0	0
Fiber	0.07	0.08	0	0	0
Fruit	0.2	0.33	0.03	0	0
Oil crops	2.46	3.32	0.24	0	0
Pulses	0.09	0.16	0.02	0	0
Roots & tubers	1.27	1.67	0.11	0	0
Sugar crops	0.02	0.03	0	0	0
Vegetables	0	0	0	0	0
**Chad**					
Cereals	14.96	27.3	2.85	12.3	0
Fiber	2.35	4.4	0.47	0.75	0
Fruit	0.03	0.06	0.01	0.04	0
Livestock	0.73	1.13	0.1	0.22	0
Oil crops	1.73	2.74	0.25	0.61	0
Pulses	1.5	2.62	0.26	0.83	0
Roots & tubers	0.7	1.34	0.14	0.41	0
Sugar crops	0.06	0.11	0.01	0.02	0
Vegetables	0.01	0.02	0	0.01	0
**Chile**					
Cereals	0.62	10.13	10.39	20.89	67.42
Fiber	0	0	0	0	0
Fruit	0.05	0.87	0.87	1.71	6.01
Livestock	0.09	1.4	1.4	2.59	7.88
Oil crops	0.04	0.72	0.72	1.41	4.96
Pulses	0.02	0.27	0.25	0.4	1.33
Roots & tubers	0.03	0.43	0.48	1.16	4.46
Sugar crops	0.07	1.21	1.21	2.38	8.33
Vegetables	0.02	0.31	0.32	0.62	2.17
**China**					
Cereals	5222.82	2655.24	0	1510.1	0
Fiber	477.98	344.35	0	262.21	0
Fruit	40.17	19.57	0	8.27	0
Livestock	590.92	238.99	0	102.38	0
Oil crops	2176.76	1329.89	0	775.85	0
Pulses	80.29	39.23	0	27.66	0
Roots & tubers	547.23	161.03	0	51.86	0
Sugar crops	44.83	39.54	0	14.67	0

Vegetables	71.62	33.54	0	14.23	0
**Colombia**					
Cereals	0.13	2.6	3.41	11.43	49.78
Fiber	0.05	0.85	0.83	1.38	3.59
Fruit	0.07	1.24	1.39	3.32	10.18
Livestock	0.23	3.7	3.63	6.22	15.43
Oil crops	0.05	0.76	0.75	1.58	7.61
Pulses	0.07	1.11	1.19	2.43	5.53
Roots & tubers	0.16	2.59	2.55	4.25	8.94
Sugar crops	0.14	2.3	2.31	4.16	9.85
Vegetables	0.01	0.22	0.22	0.37	0.95
**Congo**					
Cereals	0.2	0.3	0.03	0	0
Fiber	0	0	0	0	0
Fruit	0.1	0.24	0.03	0.03	0
Livestock	0.04	0.08	0.01	0.03	0
Oil crops	0.03	0.05	0	0.01	0
Pulses	0.15	0.26	0.02	0.04	0
Roots & tubers	0.82	1.51	0.16	0.06	0
Sugar crops	0.1	0.15	0.01	0.02	0
Vegetables	0	0.01	0	0	0
**Costa Rica**					
Cereals	0	0.13	0.5	0.47	0.79
Fiber	0	0	0.01	0.01	0.02
Fruit	0.01	0.47	1.77	1.66	2.81
Livestock	0.01	0.15	0.51	0.7	1.76
Oil crops	0	0.02	0.09	0.08	0.14
Pulses	0	0.04	0.15	0.14	0.24
Roots & tubers	0	0.05	0.2	0.18	0.31
Sugar crops	0	0.15	0.58	0.54	0.92
Vegetables	0	0.01	0.05	0.05	0.08
**Croatia**					
Cereals	7.54	27.21	10.56	29.84	10.28
Fiber	0	0	0	0	0
Fruit	0.08	0.26	0.09	0.22	0.08
Livestock	0.26	0.9	0.32	0.92	0.33
Oil crops	2.69	9.36	3.4	9.5	3.32
Pulses	0.02	0.09	0.03	0.1	0.03
Roots & tubers	0.16	0.56	0.2	0.54	0.19
Sugar crops	0.81	2.9	1.12	3.23	1.13
Vegetables	0.01	0.04	0.02	0.05	0.02
**Cuba**					
Cereals	1.33	3.01	2.88	2.09	3.27
Fiber	0	0	0	0	0
Fruit	0.31	0.7	0.68	0.51	0.83
Livestock	0.13	0.48	0.92	0.77	1.45

Oil crops	0.15	0.35	0.33	0.24	0.37
Pulses	0.56	1.26	1.2	0.87	1.36
Roots & tubers	0.54	1.21	1.15	0.84	1.31
Sugar crops	0.61	1.38	1.32	0.96	1.5
Vegetables	0.2	0.46	0.44	0.32	0.5
**Cyprus**					
Cereals	0	0	0	0	0
Fiber	0	0	0	0	0
Fruit	0	0.01	0	0	0
Livestock	0.1	0.47	0.17	0.08	0.01
Oil crops	0	0	0	0	0
Pulses	0	0	0	0	0
Roots & tubers	0	0	0	0	0
Sugar crops	0	0	0	0	0
Vegetables	0	0	0	0	0
**Czech Republic**					
Cereals	18.87	70.13	29.29	88.53	31.03
Fiber	0	0	0	0	0
Fruit	0.04	0.15	0.06	0.12	0.02
Livestock	0.27	1.73	1.25	4.08	1.31
Oil crops	10.57	40.44	17.83	56.35	20.09
Pulses	0.33	1.16	0.43	1.12	0.36
Roots & tubers	0.23	1.16	0.73	2.18	0.62
Sugar crops	1.88	6.41	2.23	6.57	2.47
Vegetables	0.02	0.06	0.02	0.05	0.02
**Democratic Republic of the Congo**					
Cereals	10.55	18.1	1.78	4.46	0
Fiber	0.48	0.89	0.09	0.26	0
Fruit	1.77	2.78	0.24	0.83	0
Livestock	0.3	0.56	0.06	0.04	0
Oil crops	1.03	1.71	0.16	0.26	0
Pulses	2.07	3.39	0.31	0.79	0
Roots & tubers	10.06	18.82	1.98	5.1	0
Sugar crops	0.22	0.4	0.04	0.13	0
Vegetables	0.06	0.11	0.01	0.04	0
**Denmark**					
Cereals	6.82	56.72	43.89	147.67	51.32
Fiber	0	0	0	0	0
Fruit	0	0.01	0.01	0.02	0.01
Livestock	0.42	3.42	2.6	8.56	2.93
Oil crops	1.6	13.29	10.28	34.4	11.89
Pulses	0.03	0.26	0.2	0.68	0.24
Roots & tubers	0.2	1.64	1.27	4.29	1.49
Sugar crops	0.34	2.79	2.16	7.25	2.52
Vegetables	0	0.03	0.02	0.07	0.02

**Djibouti**					
Cereals	0	0	0	0	0
Fiber	0	0	0	0	0
Fruit	0	0	0	0	0
Livestock	0	0	0	0	0
Oil crops	0	0	0	0	0
Pulses	0	0	0	0	0
Roots & tubers	0	0	0	0	0
Sugar crops	0	0	0	0	0
Vegetables	0	0	0	0	0
**Dominica**					
Cereals	0	0	0	0	0
Fiber	0	0	0	0	0
Fruit	0	0.01	0.02	0.01	0.02
Livestock	0	0	0.01	0	0
Oil crops	0	0.01	0.04	0.03	0.03
Pulses	0	0	0	0	0
Roots & tubers	0	0.01	0.02	0.01	0.01
Sugar crops	0	0	0	0	0
Vegetables	0	0	0	0	0
**Ecuador**					
Cereals	0.36	5.68	5.47	8.47	16
Fiber	0	0.02	0.02	0.05	0.09
Fruit	0.18	2.68	2.4	2.83	4.72
Livestock	0.08	1.24	1.21	2.03	5.26
Oil crops	0.01	0.3	0.45	1.58	5.13
Pulses	0.01	0.19	0.18	0.26	0.45
Roots & tubers	0.01	0.19	0.22	0.52	1.25
Sugar crops	0.03	0.46	0.45	0.78	2
Vegetables	0	0.04	0.03	0.04	0.06
**Egypt**					
Cereals	61.78	285.79	106.01	51.42	9.12
Fiber	3.83	17.71	6.57	3.19	0.56
Fruit	2.29	10.6	3.93	1.91	0.34
Livestock	2.75	12.7	4.71	2.29	0.41
Oil crops	2.84	13.13	4.87	2.36	0.42
Pulses	0.39	1.8	0.67	0.32	0.06
Roots & tubers	2.12	9.8	3.63	1.76	0.31
Sugar crops	4.16	19.23	7.13	3.46	0.61
Vegetables	2.74	12.67	4.7	2.28	0.4
**El Salvador**					
Cereals	3.11	7.71	8.4	3.55	1.55
Fiber	0.01	0.03	0.03	0.01	0
Fruit	0.06	0.13	0.12	0.05	0.02
Livestock	0.21	0.64	0.98	0.41	0.18
Oil crops	0.32	0.7	0.55	0.23	0.1

Pulses	0.22	0.98	1.96	0.83	0.36
Roots & tubers	0.01	0.03	0.02	0.01	0
Sugar crops	0.49	0.98	0.57	0.24	0.1
Vegetables	0.02	0.03	0.02	0.01	0
**Equatorial Guinea**					
Cereals	0	0	0	0	0
Fiber	0	0	0	0	0
Fruit	0.08	0.1	0	0	0
Livestock	0.01	0.01	0	0	0
Oil crops	0.04	0.04	0	0	0
Pulses	0	0	0	0	0
Roots & tubers	0.2	0.24	0.01	0	0
Sugar crops	0	0	0	0	0
Vegetables	0	0	0	0	0
**Eritrea**					
Cereals	3.96	8.12	0.91	0	0
Fiber	0	0	0	0	0
Fruit	0	0	0	0	0
Livestock	0.22	0.38	0.04	0	0
Oil crops	0.45	1.11	0.14	0	0
Pulses	0.08	0.19	0.02	0	0
Roots & tubers	0.02	0.04	0	0	0
Sugar crops	0	0	0	0	0
Vegetables	0	0	0	0	0
**Estonia**					
Cereals	0.34	3.13	2.57	13.99	6.62
Fiber	0	0	0	0	0
Fruit	0	0	0	0	0
Livestock	0.01	0.12	0.12	0.58	0.25
Oil crops	0.19	1.74	1.44	8.35	4.05
Pulses	0	0.04	0.03	0.18	0.09
Roots & tubers	0.01	0.12	0.1	0.57	0.27
Sugar crops	0	0	0	0	0
Vegetables	0	0	0	0.01	0
**Ethiopia**					
Cereals	127.95	227.43	22.88	19.9	0
Fiber	1.33	2.51	0.27	0.45	0
Fruit	0.13	0.25	0.03	0.04	0
Livestock	3.52	5.74	0.53	0.26	0
Oil crops	8.16	13.83	1.32	1.98	0
Pulses	7.77	11.23	0.87	0.92	0
Roots & tubers	1.69	3.18	0.34	0.57	0
Sugar crops	0.37	0.7	0.07	0.13	0
Vegetables	0.13	0.25	0.03	0.04	0
**Finland**					
Cereals	4.51	35.38	27.51	70.13	15.71

Fiber	0	0	0	0	0
Fruit	0	0	0	0	0
Livestock	0.21	1.42	1.02	2.26	0.39
Oil crops	0.57	4.45	3.44	8.77	1.98
Pulses	0.01	0.1	0.08	0.2	0.05
Roots & tubers	0.11	0.89	0.7	1.82	0.42
Sugar crops	0.17	1.34	1.03	2.63	0.59
Vegetables	0	0.03	0.02	0.06	0.01
**France**					
Cereals	73	378.36	236.18	934	370.73
Fiber	0	0	0	0	0
Fruit	1.4	7.29	4.14	9.67	2.4
Livestock	4.65	21.9	12.35	28.88	6.06
Oil crops	24.1	120.31	74.61	371.41	168.33
Pulses	2.63	11.77	6.29	20.14	6.96
Roots & tubers	0.39	5.19	5.16	20.91	7.65
Sugar crops	0.95	16.16	17.31	107.91	51.21
Vegetables	0.12	0.52	0.28	0.89	0.3
**Gabon**					
Cereals	0.37	0.49	0.04	0	0
Fiber	0	0	0	0	0
Fruit	0.44	0.6	0.05	0	0
Livestock	0.03	0.08	0.01	0	0
Oil crops	0.14	0.18	0.01	0	0
Pulses	0	0	0	0	0
Roots & tubers	0.56	0.74	0.06	0	0
Sugar crops	0.04	0.06	0.01	0	0
Vegetables	0	0	0	0	0
**Gambia**					
Cereals	1.69	3.23	0.35	0	0
Fiber	0.01	0.02	0	0	0
Fruit	0	0	0	0	0
Livestock	0.03	0.07	0.01	0	0
Oil crops	0.13	0.22	0.02	0	0
Pulses	0.01	0.02	0	0	0
Roots & tubers	0.01	0.01	0	0	0
Sugar crops	0	0	0	0	0
Vegetables	0	0	0	0	0
**Georgia**					
Cereals	1.49	7.81	2.89	1.49	0.26
Fiber	0	0	0	0	0
Fruit	0.07	0.46	0.19	0.1	0.02
Livestock	0.2	0.94	0.33	0.17	0.03
Oil crops	0.15	0.77	0.29	0.15	0.03
Pulses	0.09	0.44	0.16	0.08	0.01
Roots & tubers	0.17	0.91	0.34	0.18	0.03

Sugar crops	0	0	0	0	0
Vegetables	0.03	0.17	0.06	0.03	0.01
**Germany**					
Cereals	60.52	307.06	181.78	624.21	228.93
Fiber	0	0	0	0	0
Fruit	0.89	2.76	0.71	1.11	0.26
Livestock	3.36	16.98	9.92	36.27	14.16
Oil crops	28.81	153.08	96.22	388.26	157.59
Pulses	0.69	3.13	1.66	5.57	2.06
Roots & tubers	2.06	12.34	8.21	30.55	11.77
Sugar crops	3.99	31.61	23.67	67.15	19.51
Vegetables	0.05	0.22	0.11	0.33	0.12
**Ghana**					
Cereals	18.73	26.26	1.96	0.01	0
Fiber	0.37	0.66	0.07	0	0
Fruit	4.9	6.74	0.5	0	0
Livestock	0.61	0.9	0.07	0	0
Oil crops	1.32	2.41	0.25	0	0
Pulses	3.72	4.49	0.22	0	0
Roots & tubers	16.95	23.45	1.76	0	0
Sugar crops	0.03	0.04	0	0	0
Vegetables	0.19	0.32	0.03	0	0
**Greece**					
Cereals	14.7	58.12	25.02	41.54	4.36
Fiber	5.82	23.22	10.35	18.14	2.08
Fruit	0.49	1.93	0.82	1.29	0.12
Livestock	0.88	3.72	1.63	2.36	0.16
Oil crops	7.35	28.42	11.49	17.46	1.55
Pulses	0.14	0.52	0.19	0.28	0.03
Roots & tubers	0.49	2.01	0.93	1.61	0.18
Sugar crops	1.19	4.56	1.87	3.04	0.32
Vegetables	0.48	1.8	0.71	1.11	0.11
**Grenada**					
Cereals	0	0	0	0	0
Fiber	0	0	0	0	0
Fruit	0	0	0	0	0
Livestock	0	0	0	0	0
Oil crops	0	0	0	0	0
Pulses	0	0	0	0	0
Roots & tubers	0	0	0	0	0
Sugar crops	0	0	0	0	0
Vegetables	0	0	0	0	0
**Guatemala**					
Cereals	1.96	6.13	10.42	7.15	9.06
Fiber	0.01	0.04	0.08	0.05	0.05
Fruit	0.21	0.77	1.45	0.84	0.86

Livestock	0.49	1.37	1.91	1.01	0.89
Oil crops	0.58	2.13	4.03	2.32	2.35
Pulses	0.46	1.43	2.35	1.51	1.77
Roots & tubers	0.13	0.49	0.92	0.53	0.54
Sugar crops	0.47	1.73	3.29	1.9	1.93
Vegetables	0.05	0.18	0.34	0.2	0.21
**Guinea**					
Cereals	10.11	22.44	2.7	0.01	0
Fiber	0.72	1.46	0.17	0	0
Fruit	0.67	1.42	0.17	0	0
Livestock	0.47	0.75	0.07	0	0
Oil crops	0.19	0.34	0.04	0	0
Pulses	0.13	0.43	0.06	0	0
Roots & tubers	1.11	2.13	0.23	0	0
Sugar crops	0.04	0.09	0.01	0	0
Vegetables	0	0	0	0	0
**Guinea-Bissau**					
Cereals	1.63	1.97	0.12	0	0
Fiber	0.13	0.15	0.01	0	0
Fruit	0.08	0.1	0.01	0	0
Livestock	0.1	0.12	0.01	0	0
Oil crops	0.25	0.31	0.02	0	0
Pulses	0.01	0.01	0	0	0
Roots & tubers	0.04	0.05	0	0	0
Sugar crops	0	0	0	0	0
Vegetables	0	0	0	0	0
**Guyana**					
Cereals	0	0.04	0.08	0.5	3.1
Fiber	0	0	0	0	0
Fruit	0	0	0	0.01	0.03
Livestock	0	0.01	0.01	0.04	0.16
Oil crops	0	0.04	0.06	0.22	0.7
Pulses	0	0	0	0	0.02
Roots & tubers	0	0	0	0.01	0.05
Sugar crops	0	0.04	0.06	0.27	1.15
Vegetables	0	0	0	0	0
**Haiti**					
Cereals	1.62	3.66	3.24	1.37	0.6
Fiber	0.01	0.03	0.02	0.01	0
Fruit	0.24	0.54	0.48	0.2	0.09
Livestock	0.18	0.41	0.34	0.14	0.06
Oil crops	0.15	0.34	0.31	0.13	0.06
Pulses	0.44	1	0.89	0.38	0.16
Roots & tubers	0.46	1.04	0.92	0.39	0.17
Sugar crops	0.08	0.19	0.17	0.07	0.03
Vegetables	0	0.01	0.01	0	0

**Honduras**					
Cereals	1.64	3.32	2.19	2.69	6.91
Fiber	0.01	0.02	0.01	0.02	0.04
Fruit	0.18	0.38	0.28	0.31	0.74
Livestock	0.27	0.56	0.41	0.49	1.23
Oil crops	0.06	0.12	0.09	0.1	0.24
Pulses	0.22	0.49	0.44	0.76	2.21
Roots & tubers	0.02	0.04	0.03	0.03	0.08
Sugar crops	0.25	0.52	0.4	0.48	1.22
Vegetables	0.04	0.09	0.06	0.07	0.16
**Hungary**					
Cereals	31.45	124.11	53.42	141.62	45.95
Fiber	0	0	0	0	0
Fruit	0.13	0.59	0.3	0.88	0.3
Livestock	0.4	2.56	1.7	4.03	0.93
Oil crops	6.48	27.18	12.91	39.94	14.4
Pulses	0.14	0.68	0.37	1.01	0.31
Roots & tubers	0.33	1.2	0.46	1.13	0.36
Sugar crops	1.63	5.88	2.2	4.78	1.25
Vegetables	0.08	0.26	0.08	0.19	0.06
**Iceland**					
Cereals	0	0	0	0	0
Fiber	0	0	0	0	0
Fruit	0	0	0	0	0
Livestock	0	0	0	0	0
Oil crops	0	0	0	0	0
Pulses	0	0	0	0	0
Roots & tubers	0	0	0	0	0
Sugar crops	0	0	0	0	0
Vegetables	0	0	0	0	0
**India**					
Cereals	1549.83	2771.56	311.92	58.95	0
Fiber	144.37	426.42	58.91	11.13	0
Fruit	13.38	29.74	3.69	0.71	0
Livestock	47.53	95.99	11.31	2.2	0
Oil crops	832.99	1840.89	227.85	43.71	0
Pulses	60.72	149.42	19.27	3.7	0
Roots & tubers	60.13	115.31	13.46	2.55	0
Sugar crops	35.73	94.81	12.7	2.39	0
Vegetables	16.07	25.79	2.65	0.52	0
**Indonesia**					
Cereals	279.41	387.16	13.83	93	0
Fiber	0.08	0.14	0.01	0.07	0
Fruit	3.28	5.77	0.31	2.98	0
Livestock	15.58	21.82	0.78	1.09	0
Oil crops	110.12	166.12	7.07	60.79	0

Pulses	4.89	8.6	0.46	4.45	0
Roots & tubers	21.72	31.07	1.14	4.99	0
Sugar crops	3.71	6.52	0.35	3.38	0
Vegetables	0.81	1.42	0.08	0.73	0
**Iran (Islamic Republic of)**					
Cereals	176.93	353.83	41.2	140.78	0
Fiber	5.52	9.1	0.95	1.71	0
Fruit	5.28	11.88	1.46	3.89	0
Livestock	6.11	19.26	2.67	9.17	0
Oil crops	8.42	62.54	10.28	8.35	0
Pulses	2.97	6.68	0.83	2.41	0
Roots & tubers	5.44	10.94	1.27	7.84	0
Sugar crops	4.56	19.61	2.95	10.46	0
Vegetables	3.68	7.98	0.96	3.59	0
**Iraq**					
Cereals	21.07	66.55	17.24	9.7	4.28
Fiber	0.18	0.83	0.31	0.15	0.03
Fruit	0.54	2.3	0.8	0.43	0.15
Livestock	0.3	1.25	0.4	0.38	0.44
Oil crops	0.09	0.37	0.13	0.06	0.01
Pulses	0.06	0.2	0.06	0.03	0.01
Roots & tubers	0.41	2.15	0.78	0.55	0.4
Sugar crops	0.01	0.04	0.02	0.01	0
Vegetables	0.31	1.47	0.55	0.27	0.05
**Ireland**					
Cereals	2.36	15.36	10.91	33.91	10.65
Fiber	0	0	0	0	0
Fruit	0	0.01	0.01	0.02	0.01
Livestock	1.01	4.48	2.28	5.53	1.43
Oil crops	0.11	0.71	0.49	1.54	0.5
Pulses	0.03	0.2	0.14	0.44	0.14
Roots & tubers	0.08	0.49	0.34	1.07	0.34
Sugar crops	0.16	1.56	1.33	4.24	1.31
Vegetables	0	0.01	0.01	0.02	0.01
**Israel**					
Cereals	1.79	10.14	3.94	2.01	0.35
Fiber	0.57	3.01	1.14	0.58	0.1
Fruit	0.29	1.29	0.45	0.22	0.04
Livestock	2.16	7.97	2.5	1.21	0.21
Oil crops	0.34	1.66	0.62	0.31	0.05
Pulses	0	0.02	0.01	0	0
Roots & tubers	0.86	3.97	1.44	0.71	0.12
Sugar crops	0	0	0	0	0
Vegetables	0.31	1.39	0.5	0.25	0.04
**Italy**					
Cereals	21.42	142.5	100.76	270.96	71.1

Fiber	0	0	0	0	0
Fruit	1.03	6.31	4.23	14.94	5.54
Livestock	2.26	14.87	10.15	20.09	2.56
Oil crops	10.09	46.48	24.82	74.59	24.94
Pulses	0.13	0.72	0.45	1.14	0.3
Roots & tubers	0.7	2.99	1.49	4.06	1.2
Sugar crops	1.62	12.09	9.02	21.4	4.32
Vegetables	0.82	4.46	2.76	7.2	1.9
**Ivory Coast**					
Cereals	0	0	0	0	0
Fiber	0	0	0	0	0
Fruit	0	0	0	0	0
Livestock	0.16	0.92	0.15	0.05	0
Oil crops	0	0	0	0	0
Pulses	0	0	0	0	0
Roots & tubers	0	0	0	0	0
Sugar crops	0	0	0	0	0
Vegetables	0	0	0	0	0
**Jamaica**					
Cereals	0.02	0.03	0	0	0
Fiber	0	0	0	0	0
Fruit	0.11	0.19	0.02	0.01	0
Livestock	0.36	0.6	0.06	0.02	0.01
Oil crops	1.28	2.16	0.18	0.08	0.03
Pulses	0.02	0.03	0	0	0
Roots & tubers	0.25	0.42	0.04	0.02	0.01
Sugar crops	0.29	0.49	0.04	0.02	0.01
Vegetables	0.02	0.03	0	0	0
**Japan**					
Cereals	38.3	52.23	21.05	13.71	0
Fiber	0	0	0	0	0
Fruit	0.49	0.7	0.29	0.22	0
Livestock	32.19	22.43	6.08	5.53	0
Oil crops	11.07	14.37	5.72	4.62	0
Pulses	1.54	2.19	0.9	0.69	0
Roots & tubers	7.66	8.37	3.09	2.29	0
Sugar crops	7.83	10.69	4.32	3.33	0
Vegetables	1.17	1.67	0.68	0.53	0
**Jordan**					
Cereals	0.28	1.31	0.49	0.24	0.04
Fiber	0	0	0	0	0
Fruit	0.03	0.16	0.06	0.03	0.01
Livestock	0.22	1	0.37	0.18	0.03
Oil crops	0.41	1.89	0.7	0.34	0.06
Pulses	0	0	0	0	0
Roots & tubers	0.1	0.47	0.17	0.08	0.01

Sugar crops	0	0	0	0	0
Vegetables	0.17	0.76	0.28	0.14	0.02
**Kazakhstan**					
Cereals	8.18	142.19	67.03	133.17	229.07
Fiber	1.16	8.83	3.75	4.41	6.01
Fruit	0.03	0.16	0.06	0.04	0.01
Livestock	0.76	5.55	2.34	2.36	2.87
Oil crops	0.89	10.6	4.82	5.98	8.26
Pulses	0.05	0.59	0.27	0.41	0.64
Roots & tubers	0.55	5.3	2.34	2.63	3.41
Sugar crops	0.06	0.93	0.44	0.32	0.22
Vegetables	0.14	0.89	0.37	0.27	0.22
**Kenya**					
Cereals	24.56	50.98	5.85	21	0
Fiber	0.28	0.59	0.07	0.28	0
Fruit	0.64	1.4	0.17	0.61	0
Livestock	2.54	5.67	0.68	1.85	0
Oil crops	0.75	1.47	0.16	0.6	0
Pulses	6.69	13.32	1.48	4.41	0
Roots & tubers	4.21	9.19	1.09	3.97	0
Sugar crops	0.57	1.23	0.15	0.55	0
Vegetables	0.34	0.74	0.09	0.33	0
**Kuwait**					
Cereals	0	0.06	0.03	0.02	0
Fiber	0	0	0	0	0
Fruit	0	0.07	0.04	0.02	0
Livestock	0	0.86	0.44	0.24	0.04
Oil crops	0	0	0	0	0
Pulses	0	0	0	0	0
Roots & tubers	0	0.07	0.03	0.02	0
Sugar crops	0	0	0	0	0
Vegetables	0	0.09	0.05	0.03	0
**Kyrgyzstan**					
Cereals	3.51	17.81	6.39	8.65	12.82
Fiber	0.38	2.26	0.86	1.04	1.41
Fruit	0.01	0.07	0.02	0.03	0.05
Livestock	0.39	1.56	0.48	0.44	0.48
Oil crops	0.17	0.84	0.3	0.3	0.36
Pulses	0.12	1.83	0.85	0.46	0.08
Roots & tubers	0.52	2.64	0.95	1.3	1.94
Sugar crops	0.09	0.48	0.18	0.25	0.38
Vegetables	0.05	0.24	0.09	0.11	0.16
**Lao People's Democratic Republic**					
Cereals	12.15	20.98	1.04	0.38	0
Fiber	0.15	0.22	0.01	0	0

Fruit	0.12	0.24	0.01	0.01	0
Livestock	0.42	0.73	0.04	0.01	0
Oil crops	1.13	1.61	0.05	0.02	0
Pulses	0.11	0.2	0.01	0	0
Roots & tubers	0.38	0.66	0.04	0.01	0
Sugar crops	0.03	0.08	0.01	0	0
Vegetables	0	0	0	0	0
**Latvia**					
Cereals	0.84	6.94	5.34	22.77	9.6
Fiber	0	0	0	0	0
Fruit	0	0.01	0	0.02	0.01
Livestock	0.04	0.32	0.24	0.78	0.28
Oil crops	0.47	3.87	2.97	12.02	4.9
Pulses	0	0.02	0.02	0.06	0.02
Roots & tubers	0.06	0.54	0.41	1.82	0.78
Sugar crops	0.03	0.28	0.22	1.05	0.47
Vegetables	0	0.01	0.01	0.02	0.01
**Lebanon**					
Cereals	0.53	2.46	0.88	0.96	1.27
Fiber	0	0	0	0	0
Fruit	0.08	0.35	0.12	0.13	0.17
Livestock	0.11	0.67	0.26	0.56	1.01
Oil crops	0.33	1.6	0.58	0.32	0.11
Pulses	0.01	0.05	0.02	0.02	0.03
Roots & tubers	0.09	0.78	0.34	0.59	0.97
Sugar crops	0.02	0.12	0.04	0.05	0.08
Vegetables	0.06	0.33	0.12	0.13	0.16
**Lesotho**					
Cereals	0.81	1.82	0.22	0	0
Fiber	0	0	0	0	0
Fruit	0	0	0	0	0
Livestock	0.12	0.24	0.03	0	0
Oil crops	0	0	0	0	0
Pulses	0.08	0.15	0.02	0	0
Roots & tubers	0.13	0.38	0.05	0	0
Sugar crops	0	0	0	0	0
Vegetables	0	0	0	0	0
**Liberia**					
Cereals	0.42	0.94	0.11	0	0
Fiber	0	0	0	0	0
Fruit	0.09	0.26	0.04	0	0
Livestock	0.03	0.15	0.02	0	0
Oil crops	0.16	0.42	0.05	0	0
Pulses	0.01	0.02	0	0	0
Roots & tubers	0.37	0.88	0.11	0	0
Sugar crops	0.03	0.09	0.01	0	0

Vegetables	0	0	0	0	0
**Libya**					
Cereals	0.67	2.9	0.94	1.31	1.97
Fiber	0	0	0	0	0
Fruit	0.12	0.52	0.17	0.24	0.36
Livestock	0.18	1.29	0.54	0.39	0.3
Oil crops	0.39	1.74	0.57	0.81	1.23
Pulses	0.03	0.11	0.04	0.05	0.08
Roots & tubers	0.14	0.6	0.2	0.28	0.42
Sugar crops	0	0	0	0	0
Vegetables	0.07	0.3	0.1	0.14	0.21
**Lithuania**					
Cereals	3.6	18.43	11.07	36.67	12.89
Fiber	0	0	0	0	0
Fruit	0.01	0.03	0.01	0.03	0.01
Livestock	0.12	0.67	0.41	1.74	0.76
Oil crops	1.34	7.49	4.75	16.26	5.89
Pulses	0.1	0.41	0.18	0.5	0.16
Roots & tubers	0.2	0.93	0.5	1.58	0.54
Sugar crops	0.08	0.77	0.67	2.4	0.83
Vegetables	0	0.01	0	0.01	0
**Luxembourg**					
Cereals	0	0.78	1	2.66	0.42
Fiber	0	0	0	0	0
Fruit	0	0.01	0.01	0.04	0.01
Livestock	0	0.06	0.07	0.19	0.03
Oil crops	0	0.41	0.53	1.41	0.22
Pulses	0	0.01	0.01	0.03	0
Roots & tubers	0	0.02	0.02	0.06	0.01
Sugar crops	0	0	0	0	0
Vegetables	0	0	0	0	0
**Madagascar**					
Cereals	17.15	20.76	1.21	0	0
Fiber	0.31	0.38	0.02	0	0
Fruit	0.31	0.38	0.02	0	0
Livestock	1.48	1.8	0.1	0	0
Oil crops	0.47	0.57	0.03	0	0
Pulses	2.42	2.92	0.16	0	0
Roots & tubers	5.23	6.33	0.36	0	0
Sugar crops	0.41	0.8	0.09	0	0
Vegetables	0.04	0.05	0	0	0
**Malawi**					
Cereals	23.85	35.27	3.09	3.35	0
Fiber	1.13	1.73	0.15	0.08	0
Fruit	0.65	1.06	0.1	0.06	0
Livestock	0.48	0.6	0.03	0.07	0

Oil crops	3.89	4.7	0.24	0	0
Pulses	2.9	4	0.32	0.14	0
Roots & tubers	7.05	10.61	0.94	0.63	0
Sugar crops	0.43	0.69	0.07	0.04	0
Vegetables	0.07	0.11	0.01	0.01	0
**Malaysia**					
Cereals	1.37	8.77	0.98	8.87	0
Fiber	0	0	0	0	0
Fruit	0.25	0.56	0.04	0.26	0
Livestock	2.7	9.16	0.79	2.72	0
Oil crops	1.56	3.45	0.24	1.55	0
Pulses	0	0	0	0	0
Roots & tubers	0.05	0.14	0.01	0.07	0
Sugar crops	0.06	0.12	0.01	0.06	0
Vegetables	0.03	0.06	0	0.02	0
**Mali**					
Cereals	28.28	52.96	5.68	0.02	0
Fiber	7.19	13.26	1.4	0.01	0
Fruit	0.07	0.12	0.01	0	0
Livestock	1.42	2.81	0.32	0	0
Oil crops	0.35	0.63	0.07	0	0
Pulses	0	0	0	0	0
Roots & tubers	0.55	1.27	0.16	0	0
Sugar crops	0.05	0.1	0.01	0	0
Vegetables	0.09	0.21	0.03	0	0
**Mauritania**					
Cereals	1.48	2.18	0.17	0	0
Fiber	0	0	0	0	0
Fruit	0.06	0.1	0.01	0	0
Livestock	0.6	0.91	0.08	0	0
Oil crops	0	0	0	0	0
Pulses	0.23	0.36	0.03	0	0
Roots & tubers	0.01	0.02	0	0	0
Sugar crops	0	0	0	0	0
Vegetables	0	0	0	0	0
**Mauritius**					
Cereals	0	0	0	0	0
Fiber	0	0	0	0	0
Fruit	0	0	0	0	0
Livestock	0	0	0	0	0
Oil crops	0	0	0	0	0
Pulses	0	0	0	0	0
Roots & tubers	0	0	0	0	0
Sugar crops	0	0	0	0	0
Vegetables	0	0	0	0	0
**Mexico**					

Cereals	120.94	257.82	204.97	138.49	196.76
Fiber	0.01	0.38	1.45	4.7	15.56
Fruit	1.03	2.4	2.41	1.52	2.03
Livestock	8.78	21.97	25.47	14.54	15.65
Oil crops	7.34	14.25	7.31	4.12	4.89
Pulses	12.69	23.74	9.95	6.36	8.84
Roots & tubers	0.94	2.28	2.56	1.73	2.38
Sugar crops	2.89	7	7.51	4.1	4.24
Vegetables	0.72	1.76	2.11	1.81	3.28
**Mongolia**					
Cereals	0.61	0.85	0.34	2.1	0
Fiber	0	0	0	0	0
Fruit	0	0	0	0	0
Livestock	0.85	0.84	0.28	1	0
Oil crops	0.03	0.78	0.4	0.24	0
Pulses	0	0	0	0.01	0
Roots & tubers	0.09	0.13	0.05	0.32	0
Sugar crops	0	0	0	0	0
Vegetables	0	0	0	0	0
**Montenegro**					
Cereals	0.06	0.19	0.06	0.06	0
Fiber	0	0	0	0	0
Fruit	0.02	0.07	0.02	0.02	0
Livestock	0.99	3.49	1.14	1.34	0.06
Oil crops	0.01	0.02	0.01	0	0
Pulses	0.02	0.06	0.02	0.02	0
Roots & tubers	0.11	0.36	0.11	0.12	0.01
Sugar crops	0	0	0	0	0
Vegetables	0.01	0.03	0.01	0.01	0
**Morocco**					
Cereals	23.66	84.4	24.5	24.42	30.06
Fiber	0	0.01	0	0	0
Fruit	0.4	1.37	0.38	0.36	0.43
Livestock	2.17	6.71	1.71	1.09	0.73
Oil crops	2.51	8.6	2.41	2.5	3.2
Pulses	0.23	0.83	0.25	0.2	0.2
Roots & tubers	1.26	4.24	1.17	1.02	1.1
Sugar crops	2.33	8.03	2.31	2.22	2.69
Vegetables	0.66	2.2	0.61	0.5	0.5
**Mozambique**					
Cereals	9.47	25.53	3.44	3.57	0
Fiber	0.98	1.53	0.13	5.49	0
Fruit	0.07	0.2	0.03	0.05	0
Livestock	0.55	1.11	0.13	0	0
Oil crops	1.95	4.07	0.47	1.41	0
Pulses	3.08	5.02	0.48	0.28	0

Roots & tubers	3.93	9.41	1.17	0.54	0
Sugar crops	0.21	0.74	0.11	0	0
Vegetables	0.1	0.19	0.02	0.05	0
**Myanmar**					
Cereals	95.09	176.81	9.95	3.86	0
Fiber	3.42	5.78	0.28	0.67	0
Fruit	0.94	1.58	0.07	0.03	0
Livestock	5.01	7.69	0.32	0.55	0
Oil crops	51.89	86.59	3.97	1.4	0
Pulses	52.37	87.83	4.14	1.49	0
Roots & tubers	1.27	2.14	0.1	0.04	0
Sugar crops	1.56	2.43	0.1	0.04	0
Vegetables	0.61	1.03	0.05	0.02	0
**Namibia**					
Cereals	0.83	2.7	0.4	0.26	0
Fiber	0	0	0	0	0
Fruit	0.01	0.03	0.01	0.02	0
Livestock	0.32	0.53	0.05	0.15	0
Oil crops	0	0	0	0	0
Pulses	0.02	0.09	0.01	0.05	0
Roots & tubers	0	0	0	0	0
Sugar crops	0	0	0	0	0
Vegetables	0	0.01	0	0.01	0
**Nepal**					
Cereals	44.63	83.89	9.55	1.85	0
Fiber	0	0	0	0	0
Fruit	0.06	0.09	0.01	0	0
Livestock	1.66	2.99	0.33	0.06	0
Oil crops	1.01	1.94	0.22	0.04	0
Pulses	0.59	1.04	0.11	0.02	0
Roots & tubers	3.6	6.56	0.73	0.14	0
Sugar crops	0.4	0.74	0.08	0.02	0
Vegetables	0	0	0	0	0
**Netherlands**					
Cereals	1.5	9.11	6.04	27.41	12.19
Fiber	0	0	0	0	0
Fruit	0.01	0.07	0.04	0.19	0.08
Livestock	1.51	8.92	5.74	14.02	3.36
Oil crops	0	0.12	0.14	0.93	0.44
Pulses	0.01	0.08	0.05	0.23	0.1
Roots & tubers	0.63	4.65	3.6	20.24	9.66
Sugar crops	0.94	5.37	3.46	17	7.85
Vegetables	0.11	0.63	0.4	1.73	0.76
**New Zealand**					
Cereals	0.02	0.59	0.9	4.54	23.14
Fiber	0	0	0	0	0

Fruit	0	0.02	0.03	0.17	0.86
Livestock	0.03	0.81	1.18	5.35	23.32
Oil crops	0	0.01	0.01	0.07	0.37
Pulses	0	0.03	0.05	0.23	1.17
Roots & tubers	0	0.06	0.09	0.46	2.35
Sugar crops	0	0	0	0	0
Vegetables	0	0	0.01	0.04	0.19
**Nicaragua**					
Cereals	2.79	5.97	4.77	2.77	2.83
Fiber	0.03	0.05	0.04	0.03	0.03
Fruit	0.06	0.12	0.1	0.06	0.07
Livestock	0.22	0.52	0.61	0.44	0.59
Oil crops	0.2	0.44	0.35	0.21	0.23
Pulses	2.43	4.54	1.88	1.01	0.9
Roots & tubers	0.06	0.14	0.11	0.07	0.07
Sugar crops	0.31	0.65	0.52	0.31	0.34
Vegetables	0	0.01	0.01	0	0
**Niger**					
Cereals	42.02	58.31	4.42	13.09	0
Fiber	0.25	0.33	0.02	0	0
Fruit	0.03	0.04	0	0.01	0
Livestock	1.99	2.66	0.19	0.18	0
Oil crops	2.39	3.29	0.25	0.52	0
Pulses	0.29	0.4	0.03	0.06	0
Roots & tubers	0.31	0.42	0.03	0.08	0
Sugar crops	0.05	0.06	0	0	0
Vegetables	0.35	0.47	0.03	0.08	0
**Nigeria**					
Cereals	253.94	441.36	43.63	12.29	0
Fiber	8.93	16.89	1.8	0.01	0
Fruit	3.44	6.9	0.78	1.08	0
Livestock	6.09	11.45	1.23	5.31	0
Oil crops	34.15	63.11	6.59	5.91	0
Pulses	0.25	0.44	0.04	0	0
Roots & tubers	75.42	155.84	17.76	25.21	0
Sugar crops	0.25	0.3	0.02	0	0
Vegetables	2.72	3.81	0.29	0.21	0
**Norway**					
Cereals	1.9	14.04	9.97	18.62	1.87
Fiber	0	0	0	0	0
Fruit	0	0	0	0.01	0
Livestock	0.08	1	0.94	2.17	0.3
Oil crops	0.07	0.48	0.34	0.64	0.06
Pulses	0	0	0	0	0
Roots & tubers	0.08	0.6	0.43	0.8	0.08
Sugar crops	0	0	0	0	0

Vegetables	0	0.01	0.01	0.01	0
**Oman**					
Cereals	0.05	0.22	0.08	0.04	0.01
Fiber	0	0	0	0	0
Fruit	0.26	1.19	0.44	0.21	0.04
Livestock	0.04	0.18	0.07	0.03	0.01
Oil crops	0	0	0	0	0
Pulses	0	0	0	0	0
Roots & tubers	0	0.02	0.01	0	0
Sugar crops	0	0	0	0	0
Vegetables	0.01	0.06	0.02	0.01	0
**Pakistan**					
Cereals	255.4	600.1	75.99	14.62	0
Fiber	83.48	216.99	28.35	5.5	0
Fruit	1.64	3.83	0.49	0.09	0
Livestock	18.09	30.33	3.26	0.62	0
Oil crops	19.49	44.7	5.62	1.08	0
Pulses	3.63	8.22	1.03	0.2	0
Roots & tubers	3.49	7.51	0.91	0.18	0
Sugar crops	6.64	15.97	2.04	0.39	0
Vegetables	1.22	3.02	0.39	0.07	0
**Panama**					
Cereals	0.47	1.27	1.62	0.72	0.4
Fiber	0	0	0	0	0
Fruit	0.15	0.39	0.47	0.22	0.15
Livestock	0.46	0.91	0.51	0.23	0.13
Oil crops	0.02	0.06	0.08	0.04	0.03
Pulses	0.03	0.07	0.09	0.05	0.05
Roots & tubers	0.03	0.08	0.12	0.05	0.03
Sugar crops	0.07	0.22	0.36	0.15	0.07
Vegetables	0.01	0.03	0.03	0.02	0.01
**Papua New Guinea**					
Cereals	0.13	0.15	0.05	0.03	0
Fiber	0	0	0	0	0
Fruit	0.47	0.69	0.26	0.14	0
Livestock	0.29	0.22	0.06	0.03	0
Oil crops	3.45	5.04	1.94	1.05	0
Pulses	0.01	0.01	0	0	0
Roots & tubers	1.26	1.79	0.68	0.37	0
Sugar crops	0.05	0.07	0.03	0.02	0
Vegetables	0	0	0	0	0
**Paraguay**					
Cereals	0.25	4.23	4.45	10.69	50.02
Fiber	0.06	0.97	0.97	1.76	4.93
Fruit	0	0.02	0.03	0.07	0.29
Livestock	0.05	0.82	0.82	1.47	3.86

Oil crops	0.47	11.64	18.74	94.17	617.76
Pulses	0.04	0.62	0.59	0.92	2.01
Roots & tubers	0.02	0.43	0.52	1.7	8.8
Sugar crops	0.01	0.16	0.19	0.45	1.05
Vegetables	0	0.02	0.02	0.06	0.16
**Peru**					
Cereals	0.95	14.72	13.53	17.1	20.91
Fiber	0.16	2.47	2.27	2.91	3.64
Fruit	0.11	1.76	1.62	2.09	2.73
Livestock	0.22	3.34	2.98	3.29	2.72
Oil crops	0.03	0.47	0.43	0.55	0.68
Pulses	0.11	1.72	1.55	1.77	1.45
Roots & tubers	0.29	4.49	4.2	5.72	7.98
Sugar crops	0.05	0.77	0.72	0.94	1.24
Vegetables	0.02	0.37	0.34	0.38	0.37
**Philippines**					
Cereals	43.23	126.75	10.93	102.25	0
Fiber	0.04	0.08	0.01	0.04	0
Fruit	2.8	7.22	0.57	3.63	0
Livestock	7.15	15.55	0.99	1.44	0
Oil crops	37.03	95.59	7.48	48.04	0
Pulses	0.36	0.94	0.07	0.47	0
Roots & tubers	1.57	4.09	0.32	1.19	0
Sugar crops	3.87	10.11	0.76	1.09	0
Vegetables	0.12	0.32	0.03	0.16	0
**Poland**					
Cereals	37.9	152.38	70.23	241.99	94.22
Fiber	0	0	0	0	0
Fruit	0.17	0.62	0.25	0.68	0.23
Livestock	3.37	12.45	4.98	13.33	4.34
Oil crops	5.52	36.6	27.39	140.66	63.83
Pulses	0.41	1.53	0.62	1.65	0.54
Roots & tubers	7.19	24.42	8.03	16.54	4.56
Sugar crops	3.29	15.41	8.67	33.36	13.33
Vegetables	0.26	0.94	0.37	0.99	0.34
**Portugal**					
Cereals	1.81	7.02	3.03	10.68	4.35
Fiber	0	0	0	0	0
Fruit	0.5	1.63	0.48	0.89	0.24
Livestock	0.95	3.43	1.25	2.44	0.58
Oil crops	0.38	1.47	0.64	3.19	1.55
Pulses	0.01	0.05	0.02	0.09	0.04
Roots & tubers	0.49	1.71	0.57	0.81	0.09
Sugar crops	0.04	0.33	0.29	1.26	0.51
Vegetables	0.19	0.7	0.29	1.2	0.54
**Qatar**					

Cereals	0	0	0	0	0
Fiber	0	0	0	0	0
Fruit	0	0	0	0	0
Livestock	0.03	0.12	0.04	0.02	0
Oil crops	0	0	0	0	0
Pulses	0	0	0	0	0
Roots & tubers	0	0	0	0	0
Sugar crops	0	0	0	0	0
Vegetables	0	0	0	0	0
**Republic of Korea**					
Cereals	24.78	34.08	1.05	0.29	0
Fiber	0	0	0	0	0
Fruit	0.67	0.93	0.03	0.01	0
Livestock	9.24	12.71	0.39	0.11	0
Oil crops	11.2	14.91	0.43	0.12	0
Pulses	0.2	0.27	0.01	0	0
Roots & tubers	2.25	3.07	0.09	0.03	0
Sugar crops	0	0	0	0	0
Vegetables	1.27	1.74	0.05	0.01	0
**Romania**					
Cereals	20.39	92.16	50.98	196.37	77.66
Fiber	0	0	0	0	0
Fruit	0.1	0.5	0.33	1.61	0.71
Livestock	1.19	4.77	2.15	6.52	2.33
Oil crops	3.53	18.21	11.71	53.37	22.8
Pulses	0.25	0.98	0.44	1.22	0.41
Roots & tubers	1.83	7.39	3.32	7.77	2.04
Sugar crops	0.43	1.62	0.66	1.97	0.73
Vegetables	0.13	0.56	0.28	1.03	0.4
**Russian Federation**					
Cereals	119.77	511.85	259.69	1235.38	571.89
Fiber	0	0	0	0	0
Fruit	0.07	0.39	0.26	1.59	0.8
Livestock	6.26	24.79	11.33	39.29	15.24
Oil crops	13.44	62.23	34.72	174.44	81.68
Pulses	2.6	11.2	5.6	22.45	9.6
Roots & tubers	15.31	60.12	26.3	85.3	32.61
Sugar crops	7.93	29.97	12.98	76.17	39.13
Vegetables	0.44	1.83	0.88	3.65	1.6
**Rwanda**					
Cereals	3.74	6.17	0.59	0	0
Fiber	0	0	0	0	0
Fruit	3.69	6.06	0.58	0	0
Livestock	0.29	0.4	0.03	0	0
Oil crops	2.01	2.57	0.16	0	0
Pulses	6.07	9.52	0.86	0	0

Roots & tubers	4.89	6.98	0.55	0	0
Sugar crops	0.01	0.02	0	0	0
Vegetables	0.04	0.05	0	0	0
**Saint Lucia**					
Cereals	0	0	0	0	0
Fiber	0	0	0	0	0
Fruit	0	0	0	0	0
Livestock	0	0	0	0	0
Oil crops	0	0	0	0	0
Pulses	0	0	0	0	0
Roots & tubers	0	0	0	0	0
Sugar crops	0	0	0	0	0
Vegetables	0	0	0	0	0
**Saint Vincent and the Grenadines**					
Cereals	0	0	0	0	0
Fiber	0	0	0	0	0
Fruit	0	0	0	0	0
Livestock	0	0	0	0	0
Oil crops	0	0	0	0	0
Pulses	0	0	0	0	0
Roots & tubers	0	0	0	0	0
Sugar crops	0	0	0	0	0
Vegetables	0	0	0	0	0
**Saudi Arabia**					
Cereals	15.94	59.21	18.6	9.01	1.56
Fiber	0	0	0	0	0
Fruit	1.38	4.9	1.45	0.71	0.12
Livestock	0.98	5.21	1.94	1	0.17
Oil crops	0.22	0.46	0.06	0.02	0
Pulses	0.01	0.05	0.02	0.01	0
Roots & tubers	0.41	1.51	0.48	0.23	0.04
Sugar crops	0	0	0	0	0
Vegetables	0.24	0.75	0.2	0.1	0.02
**Senegal**					
Cereals	8.01	16.73	1.97	0.01	0
Fiber	0.54	1.76	0.26	0	0
Fruit	0.03	0.04	0	0	0
Livestock	0.54	1.26	0.16	0	0
Oil crops	1.32	1.62	0.11	0	0
Pulses	0	0	0	0	0
Roots & tubers	0.32	0.39	0.03	0	0
Sugar crops	0.08	0.29	0.04	0	0
Vegetables	0.15	0.3	0.03	0	0
**Serbia**					
Cereals	18.98	69.67	27.36	72.13	23.51

Fiber	0	0	0	0	0
Fruit	0.19	0.6	0.18	0.4	0.13
Oil crops	5.37	20.92	9.14	26.02	8.71
Pulses	0.43	1.45	0.48	1.16	0.37
Roots & tubers	0.4	1.5	0.61	1.76	0.62
Sugar crops	2.52	7.96	2.23	5.19	1.79
Vegetables	0.07	0.24	0.09	0.2	0.06
**Sierra Leone**					
Cereals	2.47	5.68	0.7	0	0
Fiber	0	0	0	0	0
Fruit	0.03	0.1	0.01	0	0
Livestock	0.11	0.15	0.01	0	0
Oil crops	0.12	0.26	0.03	0	0
Pulses	0.17	0.52	0.07	0	0
Roots & tubers	1.04	2.49	0.31	0	0
Sugar crops	0.01	0.02	0	0	0
Vegetables	0.01	0.03	0	0	0
**Slovakia**					
Cereals	7.62	29.17	12.64	38.22	13.38
Fiber	0	0	0	0	0
Fruit	0.03	0.09	0.02	0.05	0.02
Livestock	0.2	0.92	0.49	1.37	0.43
Oil crops	3.95	14.76	6.24	18.99	6.67
Pulses	0.09	0.34	0.15	0.45	0.16
Roots & tubers	0.11	0.46	0.24	0.66	0.2
Sugar crops	0.5	2.15	1.09	3.29	1.08
Vegetables	0.02	0.06	0.02	0.07	0.03
**Slovenia**					
Cereals	2.45	8.16	2.39	2.65	0.12
Fiber	0	0	0	0	0
Fruit	0.07	0.26	0.08	0.1	0
Livestock	0.31	1.01	0.27	0.28	0.01
Oil crops	0.22	0.74	0.22	0.24	0.01
Pulses	0.05	0.18	0.06	0.06	0
Roots & tubers	0.11	0.38	0.12	0.14	0.01
Sugar crops	0.17	0.55	0.16	0.18	0.01
Vegetables	0	0.01	0	0	0
**Somalia**					
Cereals	3.01	4.46	0.35	0	0
Fiber	0.13	0.21	0.02	0	0
Fruit	0.06	0.1	0.01	0	0
Livestock	1.25	2.31	0.25	0	0
Oil crops	2.82	4.31	0.36	0	0
Pulses	0.38	0.64	0.06	0	0
Roots & tubers	0.07	0.11	0.01	0	0
Sugar crops	0.03	0.05	0	0	0

Vegetables	0.01	0.01	0	0	0
**South Africa**					
Cereals	14.54	90.29	15.3	194.55	0
Fiber	0.2	1.08	0.18	0.61	0
Fruit	1.89	4.44	0.57	1.86	0
Livestock	3.66	11.14	1.57	12.84	0
Oil crops	7.89	27.39	4.05	21.46	0
Pulses	0.16	1.35	0.24	1.69	0
Roots & tubers	1.25	5.38	0.85	3.67	0
Sugar crops	0.57	5.65	1.01	2.98	0
Vegetables	0.25	0.97	0.15	0.55	0
**Spain**					
Cereals	33.29	152.27	81.88	271.1	98.21
Fiber	0.19	1.12	0.81	5.89	3.07
Fruit	2.28	8.31	3.17	8.36	2.81
Livestock	7.16	24.46	8.11	15.07	3.45
Oil crops	4.69	22.61	13.41	79.06	39.13
Pulses	0.53	2.14	0.99	3.58	1.46
Roots & tubers	0.97	4.28	2.1	5.68	1.81
Sugar crops	0.93	5.83	4.16	18.37	7.75
Vegetables	0.76	3.07	1.41	5.14	2.09
**Sri Lanka**					
Cereals	7.3	13.84	1.59	4.68	0
Fiber	0	0	0	0	0
Fruit	0.47	1.04	0.13	0.48	0
Livestock	0.72	1.03	0.1	0.07	0
Oil crops	7.38	13.72	1.57	4.2	0
Pulses	0.1	0.23	0.03	0.13	0
Roots & tubers	0.28	0.59	0.07	0.22	0
Sugar crops	0.09	0.22	0.03	0.15	0
Vegetables	0.09	0.19	0.02	0.09	0
**Sudan (former)**					
Cereals	15.24	81.42	29.63	34.42	46
Fiber	1.58	6	1.8	2.17	3.03
Fruit	0.44	1.61	0.47	0.57	0.81
Livestock	2.23	9.08	2.87	2.59	2.81
Oil crops	6.97	20.95	5.04	5.49	7.34
Pulses	0.19	0.72	0.21	0.24	0.32
Roots & tubers	0.39	1.36	0.38	0.45	0.63
Sugar crops	0.44	1.56	0.44	0.5	0.66
Vegetables	0.44	1.54	0.43	0.49	0.65
**Suriname**					
Cereals	0	0.01	0.03	0.17	1.19
Fiber	0	0	0	0	0
Fruit	0	0	0	0.02	0.13
Livestock	0	0	0	0.01	0.1

Oil crops	0	0	0	0.01	0.06
Pulses	0	0	0	0	0
Roots & tubers	0	0	0	0	0.01
Sugar crops	0	0	0	0.01	0.04
Vegetables	0	0	0	0	0
**Swaziland**					
Cereals	0.44	0.92	0.11	0.03	0
Fiber	0.03	0.08	0.01	0.01	0
Fruit	0.02	0.05	0.01	0	0
Livestock	0.08	0.18	0.02	0.05	0
Oil crops	0	0	0	0	0
Pulses	0.02	0.04	0.01	0	0
Roots & tubers	0.01	0.03	0	0	0
Sugar crops	0.72	1.57	0.19	0.06	0
Vegetables	0	0.01	0	0	0
**Sweden**					
Cereals	4.31	34.95	26.59	76.3	22.24
Fiber	0	0	0	0	0
Fruit	0	0	0	0.01	0
Livestock	0.18	1.43	1.09	3.53	1.2
Oil crops	1.01	7.64	5.53	15.09	4.27
Pulses	0.06	0.52	0.42	1.2	0.33
Roots & tubers	0.19	1.32	0.91	2.05	0.44
Sugar crops	0.58	4	2.69	5.53	0.97
Vegetables	0	0.02	0.02	0.05	0.01
**Switzerland**					
Cereals	1.75	9.45	6.19	13.34	1.83
Fiber	0	0	0	0	0
Fruit	0.03	0.2	0.14	0.3	0.04
Livestock	0.27	1.87	1.34	2.68	0.31
Oil crops	0.89	3.61	1.75	3.47	0.47
Pulses	0.04	0.25	0.17	0.37	0.05
Roots & tubers	0.06	0.64	0.56	1.24	0.16
Sugar crops	0.18	2.03	1.82	4.09	0.55
Vegetables	0	0.02	0.01	0.03	0
**Syrian Arab Republic**					
Cereals	37.95	107.82	25.42	15.78	11.04
Fiber	6.46	21.68	6.19	4.71	4.5
Fruit	0.25	0.89	0.27	0.21	0.21
Livestock	1.59	5.8	1.74	0.95	0.34
Oil crops	3.4	10.96	3	2.28	2.19
Pulses	0.08	0.26	0.07	0.06	0.05
Roots & tubers	0.55	1.76	0.48	0.35	0.33
Sugar crops	1.47	4.41	1.13	0.65	0.36
Vegetables	0.4	1.41	0.42	0.32	0.29
**Tajikistan**					

Cereals	3.03	13.5	4.52	3.94	4.09
Fiber	2.47	10.85	3.61	3.19	3.38
Fruit	0.05	0.23	0.08	0.07	0.07
Livestock	0.12	0.58	0.2	0.21	0.27
Oil crops	0.02	0.08	0.03	0.02	0.02
Pulses	0.06	0.3	0.11	0.08	0.07
Roots & tubers	0.36	1.62	0.54	0.48	0.5
Sugar crops	0	0	0	0	0
Vegetables	0.1	0.43	0.14	0.13	0.13
**Thailand**					
Cereals	81.76	246.05	21.15	9.22	0
Fiber	0.14	0.42	0.04	0.02	0
Fruit	1.94	4.9	0.38	0.16	0
Livestock	4.44	21.19	2.21	1	0
Oil crops	14.72	43.47	3.75	1.64	0
Pulses	2.31	4.57	0.28	0.11	0
Roots & tubers	9.82	36.1	3.46	1.55	0
Sugar crops	7.58	17.55	1.27	0.53	0
Vegetables	0.12	0.3	0.02	0.01	0
**The former Yugoslav Republic of Macedonia**					
Cereals	1.1	5.88	3.62	7.15	0.86
Fiber	0	0	0	0	0
Fruit	0.06	0.31	0.18	0.36	0.05
Livestock	0.1	0.36	0.14	0.19	0.01
Oil crops	0.04	0.18	0.1	0.2	0.02
Pulses	0.03	0.15	0.09	0.17	0.02
Roots & tubers	0.07	0.35	0.21	0.4	0.05
Sugar crops	0.01	0.06	0.04	0.07	0.01
Vegetables	0.02	0.12	0.07	0.14	0.02
**Togo**					
Cereals	7.66	14.12	1.48	0.01	0
Fiber	1.22	1.47	0.08	0	0
Fruit	0.01	0.03	0	0	0
Livestock	0.24	0.36	0.03	0	0
Oil crops	0.14	0.25	0.03	0	0
Pulses	1.14	2.17	0.24	0	0
Roots & tubers	1.54	2.7	0.27	0	0
Sugar crops	0	0	0	0	0
Vegetables	0.01	0.01	0	0	0
**Trinidad and Tobago**					
Cereals	0	0.01	0.03	0.03	0.04
Fiber	0	0	0	0	0
Fruit	0	0	0.01	0.01	0.02
Livestock	0	0	0	0	0
Oil crops	0	0.02	0.06	0.06	0.09

Pulses	0	0	0	0	0
Roots & tubers	0	0	0	0	0
Sugar crops	0	0.02	0.09	0.09	0.14
Vegetables	0	0	0	0	0
**Tunisia**					
Cereals	7.38	30.05	9.56	9.55	11.58
Fiber	0.01	0.05	0.02	0.02	0.02
Fruit	0.27	0.8	0.19	0.17	0.2
Livestock	0.45	2.38	0.88	0.61	0.44
Oil crops	2.56	12.58	4.41	4.46	5.36
Pulses	0.06	0.27	0.09	0.1	0.12
Roots & tubers	0.24	0.8	0.21	0.3	0.45
Sugar crops	0	0	0	0	0
Vegetables	0.15	0.72	0.25	0.52	0.92
**Turkey**					
Cereals	173.38	549.85	143.04	125.4	138.89
Fiber	17.11	63.59	19.63	14.41	12.61
Fruit	2.89	8.66	2.09	1.78	1.91
Livestock	7.52	20.24	4.26	2.56	1.6
Oil crops	11.37	39.07	11	7.57	5.76
Pulses	1.87	6	1.58	1.01	0.66
Roots & tubers	5.64	13.81	2.46	1.61	1.31
Sugar crops	17.99	48.11	9.92	6.17	4.13
Vegetables	4.74	14.25	3.46	2.79	2.8
**Turkmenistan**					
Cereals	2.2	36.74	17.27	21.43	29.37
Fiber	0.98	16.27	7.64	9.58	13.22
Fruit	0.02	0.39	0.18	0.23	0.32
Livestock	0.16	1.85	0.84	1.13	1.66
Oil crops	0	0	0	0	0
Pulses	0.01	0.19	0.09	0.11	0.15
Roots & tubers	0.02	0.45	0.22	0.23	0.29
Sugar crops	0.03	0.49	0.23	0.29	0.39
Vegetables	0.02	0.31	0.15	0.18	0.25
**Uganda**					
Cereals	24.32	42.54	4.31	0.02	0
Fiber	1.03	2.04	0.23	0	0
Fruit	11.93	22.17	2.36	0.01	0
Livestock	1.45	3.05	0.36	0	0
Oil crops	22.09	31.01	2.44	0.01	0
Pulses	8.83	15.88	1.65	0.01	0
Roots & tubers	8.53	16.53	1.8	0.01	0
Sugar crops	0.38	0.73	0.08	0	0
Vegetables	0.14	0.26	0.03	0	0
**Ukraine**					
Cereals	81.31	314.77	136.24	420.7	153.08

Fiber	0	0	0	0	0
Fruit	0.14	0.54	0.24	0.78	0.3
Livestock	3.07	11.26	4.44	11.71	3.8
Oil crops	24.91	97.83	43.09	135.93	50.33
Pulses	1.24	5.88	3.36	11.87	4.42
Roots & tubers	7.28	31.81	16.11	44.4	13.56
Sugar crops	5.7	25.85	13.75	48.64	18.72
Vegetables	0.37	1.43	0.62	2.05	0.8
**United Arab Emirates**					
Cereals	0	0	0	0	0
Fiber	0	0	0	0	0
Fruit	0.75	3.49	1.29	0.63	0.11
Livestock	0.06	0.28	0.1	0.05	0.01
Oil crops	0	0	0	0	0
Pulses	0	0	0	0	0
Roots & tubers	0	0.02	0.01	0	0
Sugar crops	0	0	0	0	0
Vegetables	0.03	0.15	0.06	0.03	0
**United Kingdom**					
Cereals	36.01	177.58	101.9	308.06	100.79
Fiber	0	0	0	0	0
Fruit	0.01	0.05	0.04	0.13	0.04
Livestock	2.83	14.92	8.54	18.9	4.24
Oil crops	16.08	78.16	44.42	129.52	40.6
Pulses	0.28	2.34	1.83	5.88	1.94
Roots & tubers	0.65	5.97	4.91	16.81	5.74
Sugar crops	0.81	7.61	6.32	23.47	8.65
Vegetables	0.02	0.17	0.13	0.42	0.14
**United Republic of Tanzania**					
Cereals	35.6	92.19	12.1	0.06	0
Fiber	2.98	8.28	1.12	0.01	0
Fruit	1.86	5.02	0.67	0	0
Livestock	2.08	4.06	0.44	0	0
Oil crops	6.92	18.72	2.51	0.01	0
Pulses	14.43	27.55	2.95	0.01	0
Roots & tubers	5.26	15.3	2.12	0.01	0
Sugar crops	0.26	0.82	0.12	0	0
Vegetables	0.1	0.3	0.04	0	0
**United States of America**					
Cereals	0.82	441.18	955.52	4474.47	4478.85
Fiber	0.03	22.22	52.63	266.78	282.75
Fruit	0.01	2.87	4.97	15.28	10.14
Livestock	0.2	51.49	79.73	205.38	132.7
Oil crops	0.78	502.55	1160.2	5581.69	5636.5
Pulses	0.01	5.22	10.32	43.43	39.03
Roots & tubers	0.03	10.29	17.78	57.29	33.75

Sugar crops	0	5.19	14.18	84.91	101.51
Vegetables	0	2.17	4.35	17.22	14.09
**Uruguay**					
Cereals	0.08	1.84	2.89	10.82	38.53
Fiber	0	0	0	0	0
Fruit	0	0.02	0.04	0.14	0.44
Livestock	0.01	0.24	0.4	1.7	8.2
Oil crops	0.04	0.98	1.63	10.78	94.08
Pulses	0	0.01	0.02	0.07	0.23
Roots & tubers	0	0.02	0.04	0.19	0.87
Sugar crops	0	0	0	0.01	0.1
Vegetables	0	0	0.01	0.03	0.13
**Uzbekistan**					
Cereals	16.44	98.06	38.08	35.19	38.82
Fiber	14.42	86.05	33.41	30.87	34.05
Fruit	0.23	1.38	0.54	0.5	0.55
Livestock	0.99	6.65	2.66	1.94	1.49
Oil crops	0.08	0.49	0.19	0.17	0.18
Pulses	0.01	0.05	0.02	0.02	0.02
Roots & tubers	0.45	2.64	1.02	0.95	1.05
Sugar crops	0	0	0	0	0
Vegetables	0.35	2.1	0.81	0.75	0.83
**Venezuela (Bolivarian Republic of)**					
Cereals	0.43	7.1	7.44	16.03	55.53
Fiber	0.01	0.1	0.11	0.25	0.87
Fruit	0.01	0.14	0.18	0.57	2.41
Livestock	0.16	2.48	2.3	3.27	7.51
Oil crops	0.05	0.83	0.91	2.13	7.49
Pulses	0.01	0.21	0.22	0.42	1.06
Roots & tubers	0.01	0.2	0.25	0.75	2.98
Sugar crops	0.02	0.36	0.4	0.94	3.18
Vegetables	0	0.07	0.08	0.19	0.65
**Viet Nam**					
Cereals	75.01	213.36	17.9	86.55	0
Fiber	0.35	0.8	0.06	0.21	0
Fruit	0.88	2.04	0.15	0.56	0
Livestock	8.47	12.77	0.5	4.16	0
Oil crops	14.9	36.05	2.78	5.67	0
Pulses	2.98	6.43	0.45	0.59	0
Roots & tubers	8.09	14.89	0.84	1.86	0
Sugar crops	1.55	4.53	0.38	1.7	0
Vegetables	0.12	0.29	0.02	0.06	0
**Yemen**					
Cereals	3.57	15.07	4.91	2.52	0.43
Fiber	0.17	0.68	0.21	0.11	0.02

Fruit	0.11	0.59	0.21	0.11	0.02
Livestock	0.93	2.27	0.42	0.19	0.03
Oil crops	0.5	1.8	0.52	0.26	0.04
Pulses	0.06	0.22	0.07	0.03	0.01
Roots & tubers	0.18	0.77	0.25	0.13	0.02
Sugar crops	0	0	0	0	0
Vegetables	0.09	0.49	0.18	0.09	0.02
**Zambia**					
Cereals	8.38	21.25	2.79	5.82	0
Fiber	0.88	2.39	0.33	3.61	0
Fruit	0	0	0	0	0
Livestock	0.57	1.04	0.11	0.29	0
Oil crops	2.04	6.93	1.02	0.91	0
Pulses	0.07	0.12	0.01	0.03	0
Roots & tubers	1.08	1.55	0.12	0.09	0
Sugar crops	0.38	0.62	0.06	0.14	0
Vegetables	0.04	0.06	0.01	0.01	0
**Zimbabwe**					
Cereals	6.11	30.09	4.88	1.49	0
Fiber	1.35	8.06	1.35	0.75	0
Fruit	0.03	0.19	0.03	0	0
Livestock	1.07	1.65	0.15	0.02	0
Oil crops	3.57	8.15	1.02	0.44	0
Pulses	0.3	1.11	0.17	0.03	0
Roots & tubers	0.17	0.47	0.06	0.03	0
Sugar crops	0.43	1.02	0.13	0.1	0
Vegetables	0	0.04	0.01	0	0

	Protein production (T)				
	**< 2 ha**	**2 - 20 ha**	**20 - 50 ha**	**50 - 200 ha**	**> 200 ha**
**Afghanistan**					
Cereals	136304.22	334841.34	43343.62	143436.68	0
Fiber	275.09	8755.42	1554.79	283.57	0
Fruit	280.42	1939.36	313.72	58.61	0
Livestock	42288.16	54714.06	4998.91	883.21	0
Oil crops	3048.64	3873.33	340.99	62.14	0
Pulses	612.8	1289.36	156.74	316	0
Roots & tubers	1172.52	2573.89	317.66	485.06	0
Sugar crops	17.69	52.14	7.06	1.38	0
Vegetables	0	0	0	0	0
**Albania**					
Cereals	9208.11	28830.2	7216.63	7087.2	329.54
Fiber	22.25	124.06	68.43	98.95	4.6
Fruit	115.59	455.3	178.13	229.43	10.67
Livestock	10560.98	31019.38	6359.35	5051.46	234.51
Oil crops	130.36	380.56	76.32	58.86	2.74

Pulses	886.29	2594.63	525.68	411.04	19.16
Roots & tubers	386.38	1280.38	369.1	403.73	18.78
Sugar crops	94.2	294.46	73.39	71.81	3.34
Vegetables	296.78	971.44	272.23	292.04	13.58
**Algeria**					
Cereals	56434.72	210089.05	62302.27	71415.54	96844.93
Fiber	1.71	7.94	2.69	2.44	2.61
Fruit	1986.63	7873.92	2585.66	1438.03	617.76
Livestock	20962.07	83286.52	25900.19	21002.16	20046.05
Oil crops	1528.43	5047.39	1344.9	1779.33	2658.1
Pulses	174.25	628.4	180.97	219.71	309.64
Roots & tubers	4206.64	13569.68	3573.56	3614.29	4550.18
Sugar crops	0	0	0	0	0
Vegetables	1759.22	6986.41	2179.6	1554.63	1204.29
**Angola**					
Cereals	17459.44	35009.58	3904.25	2350.17	0
Fiber	304.29	707.01	87.9	94.57	0
Fruit	2529.33	4807.82	518.62	374.7	0
Livestock	10096.07	16180.35	1454	1502.81	0
Oil crops	1097.33	1799.25	163.62	101.1	0
Pulses	4934.91	11376.79	1398.07	1395.02	0
Roots & tubers	31079.69	72562.9	9005.27	6531.3	0
Sugar crops	0	0	0	0	0
Vegetables	32.93	74.22	9.05	7.54	0
**Argentina**					
Cereals	1968.12	49781.45	80273.67	438317.12	3180646.12
Fiber	426.26	7278.69	7905.82	21642.86	116078.73
Fruit	8.27	348.83	681.06	4007.8	26035.76
Livestock	3669.56	62946.96	69194.06	170052.41	657423.88
Oil crops	6005.35	166222.29	283053.66	1725815.09	13597447.42
Pulses	76.52	1838.59	2936.73	11663.88	47255.63
Roots & tubers	83.25	1728.1	2448.18	7895.48	22503.62
Sugar crops	0	0	0	0	0
Vegetables	7.98	213.7	352.3	1732.56	10317.87
**Armenia**					
Cereals	3469.97	25532.9	10538.4	5562.26	959.28
Fiber	0	0	0	0	0
Fruit	128.84	954.93	394.39	208.44	35.93
Livestock	3507.75	18346.41	6770.22	3492.74	601.44
Oil crops	0	0	0	0	0
Pulses	63.64	472.54	195.39	103.22	17.79
Roots & tubers	639.27	4724.78	1950.2	1030.54	177.69
Sugar crops	26.44	139.98	53.93	26.99	4.74
Vegetables	200.98	1600.64	671.73	356.72	61.47
**Australia**					

Cereals	0	6214.79	26684.08	248588.6	3317842.8<br>5
Fiber	0	480.77	2187.49	21302.01	376669.46
Fruit	0	27.4	130.25	1213.15	17429.88
Livestock	0	2017.85	8085.67	73910.73	806407.04
Oil crops	0	937.06	3981.29	35944.12	375317.57
Pulses	0	88.01	417.32	4147.87	81727.13
Roots & tubers	0	53.33	217.04	1937.17	17241.83
Sugar crops	0	0	0	0	0
Vegetables	0	12.46	53.32	479.9	4926.59
**Austria**					
Cereals	42105.84	169759.36	77716.88	140332.8	16779.16
Fiber	0	0	0	0	0
Fruit	403.98	1449.52	535.07	840.49	89.85
Livestock	10972.53	66006.73	44133.7	88615.98	10685.35
Oil crops	8152.15	30894.7	12857.53	22317.16	2643.63
Pulses	1778.28	7155.27	3267.68	5900.03	706.61
Roots & tubers	1306.38	4611.64	1668.22	2653.33	297.46
Sugar crops	4012.89	14414.07	5455.42	9071.95	1067.27
Vegetables	123.8	498.44	227.7	410.69	49.07
**Azerbaijan**					
Cereals	39879.86	128191.85	34517.93	27423.78	27517
Fiber	7179.83	23066.91	6204.69	4943.44	4975.43
Fruit	166.19	532.17	142.78	112.74	112.36
Livestock	10143.6	33664	9262.5	8328.66	9484.78
Oil crops	264.82	853.4	230.44	182.96	183.34
Pulses	385.25	2011.43	728.88	458.72	243.27
Roots & tubers	2464.03	7839.67	2086.07	1670.15	1694.57
Sugar crops	220.16	715.54	195.39	151.15	146.48
Vegetables	883.26	2843.58	771.94	490.5	336.69
**Bangladesh**					
Cereals	773301.88	1706454.7	211183.13	400968.01	0
Fiber	4304.14	9633.37	1208.96	1299.91	0
Fruit	1812.46	4049.29	503.76	1130.84	0
Livestock	66153	122805.18	13738.54	19952.04	0
Oil crops	18733.41	55793.35	7748.47	12400.19	0
Pulses	3956.25	9081.29	1142.2	2460.99	0
Roots & tubers	19771.71	41381.8	4985.13	15063.46	0
Sugar crops	0	0	0	0	0
Vegetables	2165.32	4663.27	570.66	1076.21	0
**Belarus**					
Cereals	50882.73	193159.62	80466.33	233181.19	81632.95
Fiber	0	0	0	0	0
Fruit	15.93	142.57	115.43	398.44	138.63
Livestock	19534.79	81965.41	39285.19	121547.96	43308.7
Oil crops	5077.94	19292.73	8071.72	23094.99	7942.45

Pulses	2243.19	8549.55	3583.32	10232.08	3522.26
Roots & tubers	2343.18	21038.86	17066.81	63464.8	23639.7
Sugar crops	734.56	6875.58	5710.69	22243.58	8526.36
Vegetables	293.58	1151.25	497.99	1329.01	425.57
**Belgium**					
Cereals	21503.82	83090.99	36385.62	125827.05	49512.91
Fiber	0	0	0	0	0
Fruit	71.96	262.09	103.61	283.11	93.34
Livestock	32743.08	119412.32	45212.42	103129.33	29626.52
Oil crops	22.16	645.1	732.77	6836.67	3740.65
Pulses	69.36	294	146.81	372.04	98.28
Roots & tubers	5692.86	19020.32	6029.75	10395.83	2106.93
Sugar crops	4374.44	18366.18	8950.17	29370.21	10827.19
Vegetables	211.76	786.46	317.77	836.76	265.77
**Belize**					
Cereals	80	720.55	1895.69	810.59	375.91
Fiber	0	0	0	0	0
Fruit	51.46	462.74	1216.4	518.53	237.02
Livestock	125.51	396.14	650.73	378.44	389.26
Oil crops	3.73	33.59	88.37	37.77	17.46
Pulses	16.46	153.14	409.53	185.24	107.52
Roots & tubers	0.36	3.33	8.84	3.9	2.05
Sugar crops	0	0	0	0	0
Vegetables	0.12	1.08	2.84	1.23	0.6
**Benin**					
Cereals	25778.66	43849.53	4325.02	17.17	0
Fiber	18303.49	58259.07	8338.38	45.87	0
Fruit	183.51	312.89	30.41	0.12	0
Livestock	3310.38	5181.74	455.41	1.62	0
Oil crops	1999.3	3154.6	291.03	1	0
Pulses	7525.66	12953.42	1288.2	5.23	0
Roots & tubers	25918.15	32450.72	2043.13	2.74	0
Sugar crops	0	0	0	0	0
Vegetables	434.83	710.8	67.35	0.25	0
**Bhutan**					
Cereals	5151.19	7466.78	692.07	141.72	0
Fiber	0	0	0	0	0
Fruit	120.33	173.87	16.1	3.29	0
Livestock	888.67	1295.1	120.27	24.71	0
Oil crops	204.76	304.1	28.44	5.92	0
Pulses	599.53	740.63	64.23	11.4	0
Roots & tubers	360.25	496.15	45.09	8.87	0
Sugar crops	0	0	0	0	0
Vegetables	4.6	5.62	0.48	0.09	0
**Bolivia (Plurinational State of)**					

Cereals	663.18	11080.1	11690.86	25727.8	90725.85
Fiber	100.71	1752.94	1952.61	5364.83	26042.03
Fruit	86.88	1330.62	1195.79	1402.61	1921.32
Livestock	628.93	10390.54	10837.36	20998.9	46474.42
Oil crops	482.36	11500.18	17774.97	82688.79	498488.98
Pulses	68.1	1077.66	1033.24	1667.55	4456.19
Roots & tubers	215.62	3343.09	3087.74	3982.96	5022.09
Sugar crops	0	0	0	0	0
Vegetables	7.72	123.98	122.08	230.58	851.8
**Bosnia and Herzegovina**					
Cereals	10534.47	40087.24	17208.71	31524.91	4033.42
Fiber	0	0	0	0	0
Fruit	23.56	99.88	50.51	99.4	13.09
Livestock	3235.86	12579.75	5442.39	9568.23	1129.36
Oil crops	404.74	1702.31	864	1737.47	236.33
Pulses	375.38	1433.98	613.49	1104.5	137.33
Roots & tubers	659.29	2483.5	1048.57	1908.71	244.06
Sugar crops	0	0.04	0.04	0.11	0.02
Vegetables	65.63	251.37	108.1	195.32	24.36
**Botswana**					
Cereals	403.95	1873.71	300.51	940.88	0
Fiber	126.28	470.79	71.61	117.92	0
Fruit	0.18	3.96	0.74	0.2	0
Livestock	886.43	5361.97	897.44	3391.67	0
Oil crops	67.81	293.65	46.64	169.01	0
Pulses	47	209.07	33.27	173.18	0
Roots & tubers	0	0	0	0	0
Sugar crops	0	0	0	0	0
Vegetables	0.38	2.86	0.5	0.75	0
**Brazil**					
Cereals	9291.38	164651.56	189015.48	591017.89	3376316.91
Fiber	290.01	9570.34	17423.82	120110.7	1012623.41
Fruit	1482.25	23240.1	21814.27	34415.4	110374.53
Livestock	19428.93	317067.75	322036.67	647390.43	2262868.07
Oil crops	5596.2	156799.35	268532.63	1917983.73	17375051.67
Pulses	5900.74	91981.73	85543.38	123979.38	312120.06
Roots & tubers	3071.94	48728.86	46991.03	75888.21	185153.79
Sugar crops	0	0	0	0	0
Vegetables	263.49	4241.92	4193.93	7638.58	23699.22
**Brunei Darussalam**					
Cereals	0	53.81	7.36	3.48	0
Fiber	0	0	0	0	0
Fruit	0	9.72	1.33	0.63	0

Livestock	0	2198.98	300.92	142.08	0
Oil crops	0	4.96	0.68	0.32	0
Pulses	0	0	0	0	0
Roots & tubers	0	24.77	3.39	1.6	0
Sugar crops	0	0	0	0	0
Vegetables	0	0	0	0	0
**Bulgaria**					
Cereals	20377.68	114899.84	74810.32	262586.31	95286.75
Fiber	86	226.4	27.39	4.17	1.38
Fruit	250.73	857.89	293.46	658.07	190
Livestock	7884.92	30366.26	12704.62	30306.53	8601.91
Oil crops	2177.01	17467.72	13919.64	60068.31	24726.84
Pulses	113.53	495.03	255.24	828.01	294.59
Roots & tubers	388.34	1686.83	912.67	2025.8	340.52
Sugar crops	4.38	45.39	39.67	141.64	49.09
Vegetables	87.86	361.97	173.14	574.09	213.59
**Burkina Faso**					
Cereals	91196.56	179676.8	19730.22	46793.06	0
Fiber	52789.21	97837.29	10657.12	39682.34	0
Fruit	1.14	2.32	0.26	0.7	0
Livestock	17848.5	24499.36	1835.02	3714.79	0
Oil crops	1020.14	2672.24	352.6	2114.78	0
Pulses	515.72	750.4	59.54	159.87	0
Roots & tubers	242.52	534.29	63.43	299.89	0
Sugar crops	0	0	0	0	0
Vegetables	21.54	46.15	5.4	12.34	0
**Burundi**					
Cereals	10874.98	13096.95	667.58	0.3	0
Fiber	720.37	867.92	44.78	0.03	0
Fruit	5059.52	6096.11	314.75	0.23	0
Livestock	2082.82	2509.87	130.07	0.08	0
Oil crops	440.69	530.28	26.4	0.01	0
Pulses	21241.03	25586.33	1311.86	0.96	0
Roots & tubers	7515.12	9053.36	465.42	0.34	0
Sugar crops	0	0	0	0	0
Vegetables	0	0	0	0	0
**Cambodia**					
Cereals	133217.47	311320.37	21563.36	8845.82	0
Fiber	28.32	52.82	2.65	0.95	0
Fruit	444.23	888.36	53.3	20.99	0
Livestock	10869.92	19096.11	870.41	298.1	0
Oil crops	21009.85	35015.92	1777.59	675.05	0
Pulses	3662.79	6153.49	294.91	107.3	0
Roots & tubers	6614.18	11777.06	619.44	234.02	0
Sugar crops	0	0	0	0	0
Vegetables	0	0	0	0	0

**Cameroon**					
Cereals	64076.56	98104.35	8420.84	6139.05	0
Fiber	32869.04	54632.08	5195.27	1813.67	0
Fruit	8806.02	13770.35	1205.72	1702.67	0
Livestock	16831.29	20604.73	1356.44	1.53	0
Oil crops	1526.76	2439.31	221.93	89.31	0
Pulses	20893.26	25357.45	1441.38	4.41	0
Roots & tubers	10960.86	19334.55	1927.62	6851.21	0
Sugar crops	0	0	0	0	0
Vegetables	1931.14	2829.34	225.72	100.66	0
**Canada**					
Cereals	2040.12	468145.54	734106.03	2285159.85	2416195.81
Fiber	0	0	0	0	0
Fruit	0.23	106.24	209.05	689.2	391.21
Livestock	343.84	88503.97	147234.92	374718.62	242779.39
Oil crops	1844.47	415838.51	636889.77	1770723.68	1737647.24
Pulses	259.97	58188.68	89626.08	278524.05	306189.39
Roots & tubers	36.67	8630.26	14129.25	31489.53	19062.72
Sugar crops	1.11	259.26	412.69	3181.87	5404.29
Vegetables	1.93	680.62	1385.71	4118.12	2414.09
**Central African Republic**					
Cereals	7125.98	9733.36	694.8	1.72	0
Fiber	424.15	511.9	27.63	0.02	0
Fruit	609.62	1003.99	94.89	0.37	0
Oil crops	2999.35	4049.15	289.05	0.66	0
Pulses	531.94	995.91	106.35	0.46	0
Roots & tubers	4872.46	6276.8	410.55	0.72	0
Sugar crops	0	0	0	0	0
Vegetables	0	0	0	0	0
**Chad**					
Cereals	53767.2	96502.62	9938.8	42728.73	0
Fiber	14165.81	26540.09	2836.62	4552.64	0
Fruit	55.69	114.28	13.11	76.44	0
Livestock	9398.55	14090.77	1228.44	2523.49	0
Oil crops	2104.9	3344.39	305.73	748.35	0
Pulses	5342.1	9394.66	943.9	3014.53	0
Roots & tubers	2311.18	4442.95	484.12	1305.36	0
Sugar crops	0	0	0	0	0
Vegetables	71.67	113.89	10.23	45.23	0
**Chile**					
Cereals	2040.79	33393.77	34208.58	69105.02	230225.94
Fiber	0	0	0	0	0
Fruit	113.3	1834.54	1840.08	3587.5	12683.33
Livestock	1482.91	24387.53	25012.65	49488.08	145277.08

Oil crops	94.59	1535.46	1544.37	3024.22	10602.4
Pulses	58.75	919.67	864.64	1370.93	4525.03
Roots & tubers	65.55	1119.19	1230.95	3009.05	11553.92
Sugar crops	149.64	2429.07	2443.15	4784.24	16772.76
Vegetables	70.44	1143.46	1150.09	2252.03	7895.71
**China**					
Cereals	21668836	10935904.42	0	5143209.55	0
Fiber	2884677.93	2078222.21	0	1582475.46	0
Fruit	108048.96	57863.04	0	20630.57	0
Livestock	7800906.65	3138761.29	0	1350324.91	0
Oil crops	5024969.63	3102444.34	0	1799576.71	0
Pulses	316214.73	153704.69	0	108923.45	0
Roots & tubers	1424664.58	422933.48	0	138322.31	0
Sugar crops	29767.61	41202.31	0	29527.42	0
Vegetables	277725.35	130070.62	0	55187.65	0
**Colombia**					
Cereals	565.01	11493.54	15803.5	52762.45	195118.32
Fiber	324.27	5158.77	5008.18	8338.73	21653.65
Fruit	201.66	3444.17	3779.61	8674.38	26478.69
Livestock	3730.66	59779.79	58709.45	101673.7	272793.81
Oil crops	94.78	1528.1	1513.98	3270.84	16676.91
Pulses	229.35	3890.59	4229.68	8985.75	20372.16
Roots & tubers	461.99	7417.36	7328.29	12588.52	29847.81
Sugar crops	0.01	1.32	2.98	18.91	121.52
Vegetables	56.33	898.2	874.91	1469.52	3796.3
**Congo**					
Cereals	646.98	926.11	77.48	7.39	0
Fiber	0	0	0	0	0
Fruit	273.52	706.23	92.29	77.93	0
Livestock	500.43	930.15	96.08	320.93	0
Oil crops	36.31	60.17	5.66	8.79	0
Pulses	652.31	1096.41	104.44	174.8	0
Roots & tubers	4029.85	7463.83	772.34	291.72	0
Sugar crops	0	0	0	0	0
Vegetables	11.49	17.91	1.6	2.16	0
**Costa Rica**					
Cereals	17.54	987.37	3733.61	3508.95	5949.01
Fiber	0.28	15.74	59.54	55.92	94.69
Fruit	28.69	1623.62	6141.33	5768.17	9766.61
Livestock	168.17	3522.01	12014.24	13420.62	29508.55
Oil crops	0.73	33.41	124.62	115.32	193.92
Pulses	1.67	128.82	494.82	471.73	803.58
Roots & tubers	2.18	170.66	655.8	614.8	1023.38
Sugar crops	0	0	0	0	0
Vegetables	0.68	52.56	201.92	192.5	327.91
**Croatia**					

Cereals	22049.08	79770.96	31172.75	88838.77	30751.95
Fiber	0	0	0	0	0
Fruit	156.78	527.45	175.16	450.86	154.71
Livestock	4189.83	15738.91	6533.72	22340.93	8906.7
Oil crops	6401.82	22287.22	8099.65	22565.03	7866.6
Pulses	84.05	308.02	123.47	352.44	120.62
Roots & tubers	419.05	1451.27	516.77	1395.29	481.04
Sugar crops	1626.76	5844.07	2256.58	6502.03	2283.79
Vegetables	46.15	167.2	65.32	186.57	64.95
**Cuba**					
Cereals	5440.35	12306.55	11747	8545.37	13343.96
Fiber	0	0	0	0	0
Fruit	912.02	2079.52	2027.56	1619.63	2872.22
Livestock	2393.36	7784.89	13328.47	10610.99	18876.01
Oil crops	209.19	473.22	451.7	328.59	513.11
Pulses	1859.47	4206.29	4015.04	2920.75	4560.87
Roots & tubers	1699.47	3839.3	3653.21	2661.51	4162.97
Sugar crops	0	0	0	0	0
Vegetables	705.13	1595.06	1522.54	1107.57	1729.52
**Cyprus**					
Cereals	0	0	0	0	0
Fiber	0	0	0	0	0
Fruit	5.92	27.39	10.16	4.93	0.87
Livestock	2189.15	10126.7	3756.18	1822.07	322.99
Oil crops	0.02	0.1	0.04	0.02	0
Pulses	0.43	1.98	0.74	0.36	0.06
Roots & tubers	0	0	0	0	0
Sugar crops	0	0	0	0	0
Vegetables	0	0	0	0	0
**Czech Republic**					
Cereals	67878.02	252962.56	106325.99	323369.5	113612.7
Fiber	0	0	0	0	0
Fruit	78.28	300.55	125.55	251.08	51.03
Livestock	8677.02	43347.02	25879.46	83082.59	27907.72
Oil crops	24780.01	94897.46	41926.67	132593.11	47298.57
Pulses	1658.84	5798.33	2138.18	5585.11	1789.84
Roots & tubers	599.22	2999.78	1894.59	5651.24	1609.9
Sugar crops	3774.59	12898.39	4486.14	13224.25	4972.57
Vegetables	70.86	247.84	91.46	238.09	76
**Democratic Republic of the Congo**					
Cereals	30684.36	51965.23	5026.1	13124.41	0
Fiber	2878.71	5366.82	569.87	1559.08	0
Fruit	4417.48	7050.67	623.86	2045.96	0
Livestock	3160.76	6226.95	670.56	528.79	0
Oil crops	2164.59	3579.27	331.34	471.15	0

Pulses	7035.81	11508.65	1049.27	2699	0
Roots & tubers	49262.62	92464.99	9769.61	24921.52	0
Sugar crops	0	0	0	0	0
Vegetables	253.6	464.46	48.53	150.21	0
**Denmark**					
Cereals	25262.9	210142.78	162611.46	547209.48	190188.97
Fiber	0	0	0	0	0
Fruit	1.68	14.24	11.16	37.37	12.84
Livestock	9981.67	80999.91	61674.18	197864.17	66069.45
Oil crops	3715.44	30887.82	23897.07	79955.88	27634.11
Pulses	156.47	1303.84	1010.44	3407.24	1185.69
Roots & tubers	509.04	4250.71	3297.84	11119.45	3866.56
Sugar crops	674.75	5614.04	4345.13	14599.42	5065.64
Vegetables	15.07	125.69	97.45	328.53	114.28
**Djibouti**					
Cereals	0	0	0	0	0
Fiber	0	0	0	0	0
Fruit	0	0	0	0	0
Livestock	0	0	0	0	0
Oil crops	0	0	0	0	0
Pulses	0	0	0	0	0
Roots & tubers	0	0	0	0	0
Sugar crops	0	0	0	0	0
Vegetables	0	0	0	0	0
**Dominica**					
Cereals	0.12	1.54	4.65	2.9	3.29
Fiber	0	0	0	0	0
Fruit	2.46	32.07	97.96	62.67	73.4
Livestock	4.65	52.5	152.63	87.45	87.9
Oil crops	1.53	20.02	61.3	39.38	46.36
Pulses	0	0	0	0	0
Roots & tubers	1.57	19.17	57.37	35.15	38.95
Sugar crops	0	0	0	0	0
Vegetables	0.02	0.23	0.7	0.45	0.53
**Ecuador**					
Cereals	1906.38	29879.84	28150.5	40110.73	68728.41
Fiber	8.66	142.47	147.41	276.85	560.7
Fruit	714.96	10882.03	9635.76	10764.17	16531.69
Livestock	2217.96	35147.01	33701.32	53394.57	126391.44
Oil crops	29.12	658.6	1007.24	3614.51	11793.91
Pulses	42.71	669	629.3	896.09	1570.65
Roots & tubers	33.99	585.59	651.98	1454.58	3376.78
Sugar crops	0.4	6.44	6.29	10.27	21
Vegetables	7.66	117.84	107	131.02	181.52
**Egypt**					
Cereals	239498.86	1107888.98	410936.75	199339.9	35335.9

Fiber	23106.41	106887.09	39646.42	19231.95	3409.14
Fruit	6332.16	29291.7	10864.84	5270.39	934.25
Livestock	47170.43	218203.96	80935.93	39260.93	6959.57
Oil crops	3125.33	14457.36	5362.51	2601.28	461.11
Pulses	1323.17	6120.82	2270.33	1101.31	195.22
Roots & tubers	5483.22	25364.63	9408.22	4563.8	809
Sugar crops	6352.64	29386.44	10899.98	5287.43	937.27
Vegetables	9509.77	43990.9	16317.05	7915.18	1403.08
**El Salvador**					
Cereals	8138.38	20022.75	21497.95	9075.12	3958.38
Fiber	79.01	176.82	152.38	64.32	28.06
Fruit	180.53	403.47	346.4	146.23	63.78
Livestock	5066.19	12434.11	13287.84	5609.26	2446.71
Oil crops	508.41	1093.67	838.83	354.09	154.45
Pulses	746.36	3266.42	6561.23	2769.77	1208.08
Roots & tubers	55.41	114.34	75.85	32.02	13.97
Sugar crops	0	0	0	0	0
Vegetables	53.05	110.59	76.22	32.18	14.03
**Equatorial Guinea**					
Cereals	0	0	0	0	0
Fiber	0	0	0	0	0
Fruit	215.15	258.8	12.74	0	0
Livestock	52.67	63.35	3.12	0	0
Oil crops	51.15	61.53	3.03	0	0
Pulses	0	0	0	0	0
Roots & tubers	640.79	770.77	37.93	0.01	0
Sugar crops	0	0	0	0	0
Vegetables	0	0	0	0	0
**Eritrea**					
Cereals	13118.02	26890.25	3021.93	14.34	0
Fiber	0	0	0	0	0
Fruit	0	0	0	0	0
Livestock	3160.94	5501.7	545.32	2.26	0
Oil crops	544.57	1347.31	171.9	0.89	0
Pulses	444.54	1107.1	141.75	0.73	0
Roots & tubers	40.63	100.45	12.81	0.07	0
Sugar crops	0	0	0	0	0
Vegetables	0	0	0	0	0
**Estonia**					
Cereals	1248.26	11434.69	9382.13	51047.92	24152.21
Fiber	0	0	0	0	0
Fruit	0.16	1.5	1.25	7.27	3.53
Livestock	264.68	3875.98	3850	17283.62	6954.37
Oil crops	433.15	4047.83	3358.17	19399.97	9406.09
Pulses	19.85	184.29	152.39	868.2	418.69
Roots & tubers	34.48	318.16	262.16	1475.45	708.27

Sugar crops	0	0	0	0	0
Vegetables	0.82	7.62	6.32	36.03	17.36
**Ethiopia**					
Cereals	412045.12	762840.5	79738.44	76391.24	0
Fiber	8044.84	15167.68	1607.7	2718.33	0
Fruit	586.33	1104.1	116.89	197.46	0
Livestock	58365.63	95398.06	8867.76	2186.69	0
Oil crops	12405.15	19931.03	1787.17	2472.55	0
Pulses	30834.41	46385.04	3831.09	4568.05	0
Roots & tubers	4453.53	8373.27	885.66	1493.2	0
Sugar crops	0	0	0	0	0
Vegetables	643.66	1213.37	128.59	217.4	0
**Finland**					
Cereals	16142.13	126732.21	98555.95	251291.51	56308.87
Fiber	0	0	0	0	0
Fruit	0.23	1.82	1.44	3.73	0.84
Livestock	5847.15	35915.71	24379.49	56492.41	10998
Oil crops	1332.68	10351.86	7998.33	20394.56	4597.6
Pulses	65.37	507.78	392.33	1000.39	225.52
Roots & tubers	289.71	2301.75	1804.66	4708.48	1096.3
Sugar crops	345.94	2687.14	2076.21	5294.02	1193.45
Vegetables	15.18	118.11	91.36	232.97	52.47
**France**					
Cereals	239948.47	1275586.31	818191.69	3344512.79	1348526.6
Fiber	0	0	0	0	0
Fruit	2816.57	14672.38	8328.26	19504.52	4866.38
Livestock	119185.15	506313.35	254627.42	589741.62	130139.36
Oil crops	60121.57	296648.04	181377.79	889052.79	400953.58
Pulses	12980.36	58008.15	30991.6	99289.06	34307.08
Roots & tubers	1001.32	13440.48	13370.26	54142.5	19806.59
Sugar crops	1908.43	32521.22	34830.81	217159.98	103055.28
Vegetables	429.69	1929.09	1032.39	3274.07	1122.69
**Gabon**					
Cereals	897.26	1180.95	85.15	0.17	0
Fiber	0	0	0	0	0
Fruit	969.08	1326.69	103.54	0.26	0
Livestock	389.32	1001	131.09	0.67	0
Oil crops	322.74	419.77	30.41	0.05	0
Pulses	7.71	10.28	0.77	0	0
Roots & tubers	2042.87	2738.29	208.54	0.43	0
Sugar crops	0	0	0	0	0
Vegetables	1.03	1.9	0.2	0	0
**Gambia**					
Cereals	6100.08	11390.38	1215.83	5.3	0
Fiber	61.54	96.68	8.39	0.03	0
Fruit	0	0	0	0	0

Livestock	375.51	866.13	106.79	0.53	0
Oil crops	164.15	273.1	26.3	0.1	0
Pulses	75.21	120.3	11.16	0.04	0
Roots & tubers	40.48	58.76	4.71	0.01	0
Sugar crops	0	0	0	0	0
Vegetables	0	0	0	0	0
**Georgia**					
Cereals	4345.64	22830.11	8449.54	4356.59	750.72
Fiber	0	0	0	0	0
Fruit	144.06	933.41	377.15	195.28	33.91
Livestock	4316.73	20941.25	7421.05	3825.76	656.35
Oil crops	426.05	2238.42	828.01	427.13	73.58
Pulses	286.27	1492.07	550.67	283.61	48.87
Roots & tubers	434.63	2346.86	878.44	453.87	78.22
Sugar crops	0	0	0	0	0
Vegetables	113.33	602.55	224.38	115.69	19.96
**Germany**					
Cereals	208444.98	1094200.4	666671.47	2305263.91	845170.25
Fiber	0	0	0	0	0
Fruit	1795.85	5578.01	1442.13	2290.92	549.2
Livestock	70287.59	376492.23	232095.93	863052.69	336582.14
Oil crops	67308.35	356900.27	223942.74	903135.64	366547.34
Pulses	3425.94	15485.65	8196.8	27568.23	10195.09
Roots & tubers	5342.37	31946.61	21266.91	79121.6	30480.28
Sugar crops	8026.46	63612.55	47635.99	135137.01	39271.79
Vegetables	242.44	1043.31	516.68	1632.34	586.34
**Ghana**					
Cereals	53839.42	76535.6	5821.41	16.92	0
Fiber	2218.92	4012.1	414.31	1.79	0
Fruit	11721.87	16138.32	1198.25	2.95	0
Livestock	6351.14	9895.65	851.9	3.01	0
Oil crops	1813.7	3302.18	342.96	1.47	0
Pulses	12667.44	15299.89	763.43	0.37	0
Roots & tubers	67667.11	93660.77	7011.09	18.09	0
Sugar crops	0	0	0	0	0
Vegetables	677.52	1130.2	107.99	0.42	0
**Greece**					
Cereals	46971.42	186820.13	80876.42	133706.27	13828.87
Fiber	35136.63	140143.54	62468.73	109497.48	12579.38
Fruit	1531.53	5940.72	2443.97	3844.65	368.79
Livestock	18874.32	71633.19	27880.56	40794.47	3389.14
Oil crops	2394.74	9124.28	3644.06	5637.26	533.86
Pulses	485.95	1788.56	668.9	984.93	87.97
Roots & tubers	1256.79	5199.93	2406.72	4180.13	458.55
Sugar crops	2395.11	9178.92	3765.61	6112.14	635.44
Vegetables	1647.63	6155.71	2411.47	3797.59	384.12

**Grenada**					
Cereals	0	0	0	0	0
Fiber	0	0	0	0	0
Fruit	0	0	0	0	0
Livestock	0	0	0	0	0
Oil crops	0	0	0	0	0
Pulses	0	0	0	0	0
Roots & tubers	0	0	0	0	0
Sugar crops	0	0	0	0	0
Vegetables	0	0	0	0	0
**Guatemala**					
Cereals	4733.04	14993.99	25715.2	17436.6	21827
Fiber	68.42	251.04	473.43	272.81	276.68
Fruit	783.76	2868.42	5401.63	3121.39	3179.1
Livestock	6021.8	16712.24	23106.28	12721.96	11962.85
Oil crops	1019.8	3743.18	7061.54	4070.52	4129.83
Pulses	1579.78	4899.56	8079.86	5166.14	6046.58
Roots & tubers	348.32	1275.87	2403.7	1387.02	1409.63
Sugar crops	0	0	0	0	0
Vegetables	189.04	676.26	1256.86	743.99	785.73
**Guinea**					
Cereals	52498.13	101914.06	11207.19	50.31	0
Fiber	4333.44	8832.16	1005.32	4.68	0
Fruit	1591.66	3373.79	393.73	1.86	0
Livestock	5336.56	8770.8	822.55	3.12	0
Oil crops	248.98	463.41	49.31	0.21	0
Pulses	777.06	2613.46	380.1	2.11	0
Roots & tubers	4870.38	9096.27	967.98	4.12	0
Sugar crops	0	0	0	0	0
Vegetables	0	0	0	0	0
**Guinea-Bissau**					
Cereals	7364.95	8915.81	519.66	0.38	0
Fiber	758.52	919.1	54.82	0.05	0
Fruit	204.11	246.92	14.15	0.01	0
Livestock	1556.27	1881.17	105.71	0.08	0
Oil crops	345.71	418.21	23.94	0.02	0
Pulses	65.03	78.7	4.55	0	0
Roots & tubers	208.39	252.07	14.4	0.01	0
Sugar crops	0	0	0	0	0
Vegetables	0	0	0	0	0
**Guyana**					
Cereals	5.08	331.18	715.88	4340.93	26950.49
Fiber	0	0	0	0	0
Fruit	0.14	3.93	6.8	29.72	128.57
Livestock	4.37	115.38	193.44	794.51	3095.32
Oil crops	2.14	51.66	82.86	307.58	963.52

Pulses	0.09	2.46	4.24	18.39	78.76
Roots & tubers	0.25	6.82	11.66	49.86	208.4
Sugar crops	0	0	0	0	0
Vegetables	0.02	0.46	0.79	3.32	13.64
**Haiti**					
Cereals	5184.77	11726.14	10393.82	4387.77	1913.58
Fiber	72.29	163.26	144.17	60.86	26.54
Fruit	701.51	1583.43	1396.16	589.38	257.06
Livestock	2476.65	5502.67	4648.09	1962.12	855.83
Oil crops	194.41	440.34	391.84	165.42	72.15
Pulses	1482.52	3353.84	2974.56	1255.62	547.73
Roots & tubers	1583.72	3577.66	3161.34	1334.51	582.08
Sugar crops	0	0	0	0	0
Vegetables	13.52	31.39	29.7	12.54	5.47
**Honduras**					
Cereals	4121.22	8388.67	5622.85	6939.31	17879.4
Fiber	57.45	120.73	90.51	101.27	249.26
Fruit	745.84	1567.5	1174.71	1297.72	3172.01
Livestock	5797.74	11967.63	8476.95	8521.57	19259.56
Oil crops	95.18	199.31	147.52	169.95	425.85
Pulses	744.05	1645.12	1484.65	2528.23	7407.24
Roots & tubers	72.45	148.31	99.93	100.22	232.07
Sugar crops	0	0	0	0	0
Vegetables	141.89	295.7	214.61	232.45	562.29
**Hungary**					
Cereals	98094.19	385590.31	165186.68	440160.5	143718.79
Fiber	0	0	0	0	0
Fruit	273.53	1212.19	605.93	1788.9	615.37
Livestock	9456.67	52422.42	31509.18	74933.62	18366.61
Oil crops	17911.41	76534.82	37148.31	114068.18	40599.63
Pulses	698.87	3337.97	1801.17	4936.77	1524.13
Roots & tubers	849.87	3117.64	1189.48	2926.26	927.27
Sugar crops	3289.42	11824.57	4424.95	9611.46	2515.2
Vegetables	296.88	977.11	299.26	695.02	233.8
**Iceland**					
Cereals	0	0	0	0	0
Fiber	0	0	0	0	0
Fruit	0	0	0	0	0
Livestock	0	0	0	0	0
Oil crops	0	0	0	0	0
Pulses	0	0	0	0	0
Roots & tubers	0	0	0	0	0
Sugar crops	0	0	0	0	0
Vegetables	0	0	0	0	0
**India**					
Cereals	7248578.97	13790422.38	1605850.41	303447.66	0

Fiber	871323.48	2573515.72	355506.48	67179.59	0
Fruit	53509.38	121176.32	15174.25	2904.6	0
Livestock	1246453.29	2728066.18	335767.15	64580.03	0
Oil crops	1881219.87	4181876.69	518605.97	99565.15	0
Pulses	225269.29	549586.06	70674.12	13575.16	0
Roots & tubers	173653.45	321754.19	36906.02	6980.83	0
Sugar crops	0	0	0	0	0
Vegetables	66014.11	109577.6	11559.11	2254.3	0
**Indonesia**					
Cereals	1618724.91	2266129.27	84575.07	750124.79	0
Fiber	480.2	844.88	44.75	437.4	0
Fruit	15085.47	26580.82	1411.59	13738.26	0
Livestock	173754.8	254709	10125.13	14079.76	0
Oil crops	197230.76	282788.94	10967.66	87813.78	0
Pulses	16363.01	28789.69	1524.78	14904.75	0
Roots & tubers	98513.77	140784.08	5158.35	17713.63	0
Sugar crops	0	0	0	0	0
Vegetables	3399.64	5981.46	316.79	3096.67	0
**Iran (Islamic Republic of)**					
Cereals	676034.75	1368228.98	160414.43	539222.26	0
Fiber	33318.37	54944.44	5709.52	10330.11	0
Fruit	13830.57	30887.88	3797.3	10416.14	0
Livestock	100612.86	320547.4	44679.03	136033.67	0
Oil crops	18313.81	142646.94	23569.33	18522.95	0
Pulses	10238.39	22999.32	2842.79	8313.17	0
Roots & tubers	14089.93	28327.77	3301.45	20309.87	0
Sugar crops	7923.75	36752.23	5614.21	20037.59	0
Vegetables	13527.59	29214.27	3521.5	13724.35	0
**Iraq**					
Cereals	78218.03	250385.03	65876.91	37080.3	16309.25
Fiber	1083.43	5011.8	1858.97	901.76	159.85
Fruit	1125.53	4862.96	1695.84	905.38	311.15
Livestock	4095.62	17369.71	5722.24	4876.58	5017.45
Oil crops	288.88	1207.06	397.24	200.73	34.43
Pulses	196.6	673.64	187.69	103.46	37.84
Roots & tubers	1074.11	5570.89	2013.58	1421.75	1029.56
Sugar crops	10.25	84.3	36.32	19.01	3.31
Vegetables	1069.36	5061.54	1893.14	922.69	163.34
**Ireland**					
Cereals	8571.24	55768.04	39650.67	123201.18	38682.18
Fiber	0	0	0	0	0
Fruit	3.6	22.83	15.82	49.46	15.93
Livestock	23421.65	102764.36	51453.28	109778.2	22219.89
Oil crops	259.22	1647.05	1143.94	3581.35	1152.89
Pulses	117.9	747.05	517.49	1619.14	521.79

Roots & tubers	199.39	1269.93	884.27	2770.17	890.95
Sugar crops	317.69	3133.29	2673.97	8529.66	2639.21
Vegetables	5.66	35.88	24.86	77.79	25.07
**Israel**					
Cereals	6023.58	34959.17	13712.15	7009.66	1220.5
Fiber	3450.96	18148.72	6900.77	3481.59	607.64
Fruit	1115.99	4834.15	1674.53	828.86	144.37
Livestock	35319.4	139485.29	45718.05	22354.49	3888.36
Oil crops	501.24	2267.05	807.1	400.08	69.89
Pulses	23.43	115.67	42.84	21.47	3.75
Roots & tubers	2214.08	10278.9	3733.56	1845.62	323.6
Sugar crops	0	0	0	0	0
Vegetables	1086.29	4935.73	1766.64	874.01	152.88
**Italy**					
Cereals	69405.24	450183.69	315349.09	893417.71	253163.39
Fiber	0	0	0	0	0
Fruit	2686.84	16074.37	10604.85	35460.24	12555.89
Livestock	42519.28	274210.62	186975.98	377216.7	50214.17
Oil crops	8992.28	62855.63	45128.69	105484.26	21190.13
Pulses	588.08	3212.94	1983.11	5077.15	1310.01
Roots & tubers	1817.24	7732.53	3866.72	10520.81	3101.74
Sugar crops	3264.46	24334.29	18145.66	43074.73	8686.71
Vegetables	2736.05	14888.32	9218.78	24052.73	6332.23
**Ivory Coast**					
Cereals	0	0	0	0	0
Fiber	0	0	0	0	0
Fruit	0	0	0	0	0
Livestock	1701.5	9528.06	1579.58	530.62	0
Oil crops	0	0	0	0	0
Pulses	0	0	0	0	0
Roots & tubers	0	0	0	0	0
Sugar crops	0	0	0	0	0
Vegetables	0	0	0	0	0
**Jamaica**					
Cereals	39.94	67.15	5.69	2.4	1.05
Fiber	0	0	0	0	0
Fruit	573.94	964.54	80.36	33.92	14.8
Livestock	7186.15	12294.96	1705.89	720.08	314.11
Oil crops	1759.5	2958.28	250.59	105.79	46.13
Pulses	68.56	115.26	9.76	4.12	1.8
Roots & tubers	719.25	1212.26	111.96	47.26	20.61
Sugar crops	0	0	0	0	0
Vegetables	58.74	98.76	8.37	3.53	1.54
**Japan**					
Cereals	315826.26	377260.53	145303.66	87069.29	0
Fiber	0	0	0	0	0

Fruit	1133.65	1612.1	659.47	511.35	0
Livestock	387110.79	309425.07	94110.31	118673.73	0
Oil crops	25735.14	33408.28	13294.44	10747.17	0
Pulses	5175	7359.06	3010.42	2334.25	0
Roots & tubers	19862.58	21747.55	8042.5	5961.18	0
Sugar crops	14856.81	21127	8642.56	6701.35	0
Vegetables	5084.58	7230.48	2957.82	2293.46	0
**Jordan**					
Cereals	950.77	4398.11	1631.34	791.34	140.28
Fiber	0	0	0	0	0
Fruit	113.61	525.53	194.93	94.56	16.76
Livestock	3783.21	17500.63	6491.31	3148.85	558.18
Oil crops	104.63	484.01	179.53	87.09	15.44
Pulses	0	0	0	0	0
Roots & tubers	260.65	1205.73	447.23	216.94	38.46
Sugar crops	0	0	0	0	0
Vegetables	553.36	2559.78	949.47	460.57	81.64
**Kazakhstan**					
Cereals	30369.11	539093.19	254511.67	510297.83	880267.86
Fiber	7017.2	53296.02	22620.37	26609.37	36253.83
Fruit	58.91	328.36	129.44	73.09	30.24
Livestock	15866.01	114554.65	48124.97	46395.44	53850.34
Oil crops	2295.77	29578.2	13566.44	15905.97	21088.14
Pulses	257.14	2886.15	1302.8	1999.93	3123.9
Roots & tubers	1427.6	13722.15	6064.18	6804.14	8832.78
Sugar crops	118.12	1879.05	879.39	636.89	450.36
Vegetables	539.18	3487.86	1429.71	1048.54	858.81
**Kenya**					
Cereals	65146.25	135347.99	15525.12	56006.24	0
Fiber	1661.23	3584.7	421.69	1663.5	0
Fruit	2321.54	5128.5	612.8	2212.22	0
Livestock	49165.86	106137.06	12544.5	39828.83	0
Oil crops	1361.13	2586.24	277.67	985.85	0
Pulses	22471.03	44745.16	4987.45	14836.07	0
Roots & tubers	11513.65	25062.76	2963.77	10817.88	0
Sugar crops	0	0	0	0	0
Vegetables	1204.08	2607.8	307.63	1157.29	0
**Kuwait**					
Cereals	0	207.59	105.41	59	10.09
Fiber	0	0	0	0	0
Fruit	0	144.34	73.29	41.03	7.02
Livestock	0	8862.94	4500.56	2519.12	431
Oil crops	0	0.15	0.08	0.04	0.01
Pulses	0	0	0	0	0
Roots & tubers	0	178.28	90.53	50.67	8.67
Sugar crops	0	0	0	0	0

Vegetables	0	331.6	168.39	94.25	16.13
**Kyrgyzstan**					
Cereals	12122.13	61712.81	22165.75	29981.3	44422.21
Fiber	2281.46	13661.83	5219.23	6247.71	8512.28
Fruit	26.42	138.19	50.18	66.87	98.1
Livestock	8991.46	33483.15	9918.41	7569.93	6650.87
Oil crops	640.14	3248.87	1152.64	1153.43	1363.95
Pulses	390.49	6161.32	2880.87	1569.81	285.63
Roots & tubers	1354.21	6848.72	2453.68	3362.01	5019.88
Sugar crops	177.73	964.95	357.49	504.76	764.75
Vegetables	180.96	934.46	337.54	441.18	640.48
**Lao People's Democratic Republic**					
Cereals	78495.01	132870	6487.53	2383.27	0
Fiber	919.46	1326.01	44.49	13.04	0
Fruit	508.36	1007.57	61.54	24.56	0
Livestock	5329.73	8986.26	418.66	148.91	0
Oil crops	2158.59	3076.11	101.18	29.34	0
Pulses	500.61	921.33	48.73	18.25	0
Roots & tubers	1359.22	2166.83	100.65	36.74	0
Sugar crops	0	0	0	0	0
Vegetables	0	0	0	0	0
**Latvia**					
Cereals	3150.13	25952.06	19958	85158.31	35925.17
Fiber	0	0	0	0	0
Fruit	1.73	13.55	10.05	38.74	15.46
Livestock	953.73	7529.02	5622.09	19640.06	7209.47
Oil crops	1098.08	9000.4	6898.9	27946.04	11397.13
Pulses	11.2	90.27	68.43	257.34	99.78
Roots & tubers	167.81	1390.97	1073.96	4702.33	2013.59
Sugar crops	67.42	572.8	449.51	2118.89	942.81
Vegetables	4.62	37.12	28.04	107.89	42.63
**Lebanon**					
Cereals	2055.39	9435.08	3355.1	3573.95	4660.35
Fiber	0	0	0	0	0
Fruit	307.6	1355.9	459.42	508.93	673.86
Livestock	1696.75	9809.2	3784.11	7732.88	13641.81
Oil crops	86.77	416.22	150.35	85.76	35.91
Pulses	45.74	240.96	89.62	102.33	137.56
Roots & tubers	221.49	2026.48	888.76	1529.64	2524.76
Sugar crops	44.31	234.61	87.55	107.21	151.14
Vegetables	210.3	1119.01	422.38	455.95	591.45
**Lesotho**					
Cereals	2110.52	4800.74	586.45	2.85	0
Fiber	0	0	0	0	0
Fruit	0	0	0	0	0

Livestock	1560.66	3059.37	344.37	1.56	0
Oil crops	0	0	0	0	0
Pulses	295.02	585.7	65.95	0.3	0
Roots & tubers	337.88	994.53	138.37	0.74	0
Sugar crops	0	0	0	0	0
Vegetables	0	0	0	0	0
**Liberia**					
Cereals	3700.96	8370.15	1009.84	4.97	0
Fiber	0	0	0	0	0
Fruit	324.11	840.1	109.5	0.57	0
Livestock	430.81	1853.86	290.43	1.67	0
Oil crops	366.5	908.42	115.12	0.58	0
Pulses	56.31	137.08	17.14	0.09	0
Roots & tubers	1749.61	4173.03	519.52	2.61	0
Sugar crops	0	0	0	0	0
Vegetables	2.42	9.13	1.38	0.01	0
**Libya**					
Cereals	2447.16	10616.13	3434.51	4806.02	7228.84
Fiber	0	0	0	0	0
Fruit	254.98	1125.27	368.46	523.51	793.92
Livestock	2217.73	16222.41	6821.76	5216.67	4479.46
Oil crops	100.2	442.18	144.79	205.72	311.98
Pulses	119.87	529.03	173.22	246.12	373.24
Roots & tubers	352.76	1556.81	509.76	724.28	1098.38
Sugar crops	0	0	0	0	0
Vegetables	281.9	1244.05	407.35	578.77	877.72
**Lithuania**					
Cereals	13256.43	67958.71	40897.76	135888.72	47830.47
Fiber	0	0	0	0	0
Fruit	14.15	55.68	24.47	67.96	22.31
Livestock	3640.68	19048.74	11511.18	44319.37	17823.04
Oil crops	3110.64	17401.67	11044.2	37786.93	13695.63
Pulses	501.96	1975.42	868.62	2406.18	787.6
Roots & tubers	528.46	2414.31	1296.63	4085.28	1411.22
Sugar crops	152.14	1558.5	1355.08	4828.51	1674.19
Vegetables	13.97	54.9	24.13	67.24	22.12
**Luxembourg**					
Cereals	0	2881.38	3691.24	9833.77	1566.14
Fiber	0	0	0	0	0
Fruit	0	21.48	27.51	73.3	11.67
Livestock	0	1863.17	2386.83	6358.72	1012.7
Oil crops	0	960.5	1230.47	3278.07	522.07
Pulses	0	41.13	52.69	140.38	22.36
Roots & tubers	0	45.13	57.82	154.03	24.53
Sugar crops	0	0	0	0	0
Vegetables	0	0.1	0.13	0.33	0.05

**Madagascar**					
Cereals	120982.43	146452.75	8529.03	5.52	0
Fiber	1869.29	2263.7	133.02	0.09	0
Fruit	1345.63	1629.55	95.75	0.06	0
Livestock	23106.99	28060.15	1616.42	1.19	0
Oil crops	648.43	785.25	46.14	0.03	0
Pulses	8799.71	10628.01	584.66	0.36	0
Roots & tubers	20772.33	25130.55	1443.04	0.91	0
Sugar crops	0	0	0	0	0
Vegetables	152.33	184.47	10.84	0.01	0
**Malawi**					
Cereals	58318.13	86270.57	7572.17	8852.15	0
Fiber	6840.78	10457.05	921.98	489.24	0
Fruit	1864.89	3016.19	288.9	184.9	0
Livestock	5133.16	6426.09	376.23	975.62	0
Oil crops	9122.09	11042.49	577.34	10.25	0
Pulses	10260.62	14296.93	1168.66	534.11	0
Roots & tubers	24441.19	35551.12	3017.29	2081.01	0
Sugar crops	0	0	0	0	0
Vegetables	294.74	476.7	45.66	29.22	0
**Malaysia**					
Cereals	10201.65	72784.87	8324.48	76005.82	0
Fiber	0	0	0	0	0
Fruit	824.1	1858.88	133.26	851.35	0
Livestock	32555.36	108225.12	9165.13	40968.98	0
Oil crops	2140.1	4721.71	332.9	2119.7	0
Pulses	0	0	0	0	0
Roots & tubers	212.65	564.61	45.6	208.71	0
Sugar crops	0	0	0	0	0
Vegetables	83.21	181.14	12.63	80.49	0
**Mali**					
Cereals	108679.32	196025.85	20362.52	85.67	0
Fiber	43418.13	80024.84	8423.25	36.93	0
Fruit	317.42	548.57	55.46	0.22	0
Livestock	24767.74	49809.14	5671.74	25.95	0
Oil crops	544.53	1134.23	134.94	0.63	0
Pulses	9.02	11.46	0.77	0	0
Roots & tubers	1576.81	3615.1	441.75	2.19	0
Sugar crops	0	0	0	0	0
Vegetables	321.54	770.65	96.78	0.5	0
**Mauritania**					
Cereals	5484.41	9030.31	815.67	3.21	0
Fiber	0	0	0	0	0
Fruit	113.39	209.17	21.48	0.1	0
Livestock	9992.42	15250.46	1265.92	4.45	0
Oil crops	0	0	0	0	0

Pulses	1209.48	1882.55	159.86	0.56	0
Roots & tubers	34.05	56.08	5.13	0.02	0
Sugar crops	0	0	0	0	0
Vegetables	0	0	0	0	0
**Mauritius**					
Cereals	0	0	0	0	0
Fiber	0	0	0	0	0
Fruit	0	0	0	0	0
Livestock	0	0	0	0	0
Oil crops	0	0	0	0	0
Pulses	0	0	0	0	0
Roots & tubers	0	0	0	0	0
Sugar crops	0	0	0	0	0
Vegetables	0	0	0	0	0
**Mexico**					
Cereals	300111.33	660320	583676.83	408243.33	599129.6
Fiber	44.01	2319.27	8734.26	28339.33	93930.97
Fruit	4270.81	11005.06	13488.56	8737.85	12340.24
Livestock	123099.66	303397.92	343939.18	206971.56	246263.81
Oil crops	14465	27134.57	11370.72	6987.71	9757.62
Pulses	42485.08	79488.29	33355.06	21335.62	29649.92
Roots & tubers	2435.78	5907.63	6648.69	4506.64	6186.22
Sugar crops	0	0	0	0	0
Vegetables	2896.56	6933.17	7762.46	6642.61	12113.25
**Mongolia**					
Cereals	2349.7	3291	1299.61	8112.13	0
Fiber	0	0	0	0	0
Fruit	0	0	0	0	0
Livestock	11151.32	11026.7	3712.73	12308.17	0
Oil crops	78.31	1801.61	940.2	555.17	0
Pulses	11.96	16	6.22	41.2	0
Roots & tubers	243.37	334.83	131.48	837.18	0
Sugar crops	0	0	0	0	0
Vegetables	4.46	6.08	2.38	15.16	0
**Montenegro**					
Cereals	162.31	552	168.29	190.84	8.87
Fiber	0	0	0	0	0
Fruit	49.81	166.46	48.88	54.13	2.52
Livestock	19918.03	70489.09	23229.11	27555.02	1281.08
Oil crops	1.94	5.88	1.34	1.2	0.06
Pulses	73.27	249.09	75.88	86.01	4
Roots & tubers	274.78	929.77	280.47	315.98	14.69
Sugar crops	0	0	0	0	0
Vegetables	32.31	108.84	32.53	36.44	1.69
**Morocco**					
Cereals	88156.41	315166.65	91692.82	91607	113074.42

Fiber	11.27	36.56	9.82	8.9	10.25
Fruit	1507.13	5111.52	1419.91	1313.71	1520
Livestock	30174.58	92155.2	23082.4	14869.54	10296.44
Oil crops	1247.71	4048.13	1084.56	987.11	1137.85
Pulses	1001.14	3652.87	1071.32	874.63	858.67
Roots & tubers	3261.57	10974.57	3023.89	2631.65	2856.09
Sugar crops	4544.59	15740.87	4540.11	4353.04	5273.6
Vegetables	2541.11	8558.77	2362.83	1957.84	2006.96
**Mozambique**					
Cereals	24037.96	65058.77	8773.38	11356	0
Fiber	5936.46	9217.48	797.91	33150.41	0
Fruit	303.58	836.16	112.88	193.29	0
Livestock	7679.34	16067.3	1864.62	8.8	0
Oil crops	2626.95	5560.11	649.21	1902.47	0
Pulses	11397.49	19574.45	1977.66	1683.44	0
Roots & tubers	17504.54	42827.35	5360.48	1406.93	0
Sugar crops	0	0	0	0	0
Vegetables	378.73	698.73	74.51	202.71	0
**Myanmar**					
Cereals	762649.64	1399784.78	77512.09	29904.05	0
Fiber	20667.94	34896.49	1680	4024.16	0
Fruit	2038.2	3422.41	161.67	58.17	0
Livestock	74946.64	118318.24	5167.94	9658.27	0
Oil crops	89064.65	146949.66	6637.51	2330.87	0
Pulses	176546.83	296079.48	13967.33	5024.57	0
Roots & tubers	3742.35	6334.33	299.31	107.36	0
Sugar crops	0	0	0	0	0
Vegetables	3216.66	5400.32	255.02	91.79	0
**Namibia**					
Cereals	2727.62	8738.42	1277.85	784.62	0
Fiber	4.92	19.06	2.95	10.34	0
Fruit	17.64	67.47	10.4	36.88	0
Livestock	4176.36	6880.57	657.44	1870.46	0
Oil crops	1.4	5.39	0.83	2.93	0
Pulses	144.01	528.65	80.56	283.27	0
Roots & tubers	0	0	0	0	0
Sugar crops	0	0	0	0	0
Vegetables	8.05	30.79	4.74	16.83	0
**Nepal**					
Cereals	204539.36	377713.14	42588.47	8263.87	0
Fiber	3.89	7.46	0.86	0.17	0
Fruit	270.38	403.06	40.63	7.53	0
Livestock	29253.03	52204.5	5753.14	1122.81	0
Oil crops	2348.77	4503.97	516.18	100.75	0
Pulses	2205.97	3866.33	423.04	82.11	0
Roots & tubers	9319.14	16981.11	1897.71	369.09	0

Sugar crops	0	0	0	0	0
Vegetables	0	0	0	0	0
**Netherlands**					
Cereals	5381.85	32215.34	21245.89	100654.18	45743.71
Fiber	0	0	0	0	0
Fruit	26.88	151.9	96.26	417.99	181.91
Livestock	27257.91	181312.47	125951.39	314916.82	75319.91
Oil crops	4.39	272.26	330.32	2150.4	1029.88
Pulses	53.07	338.55	233.19	979.53	411.48
Roots & tubers	1622.94	12036.46	9323.86	52422.11	25022.98
Sugar crops	1889.1	10798.24	6969.89	34205.11	15795.44
Vegetables	479.88	2709.56	1714.71	7489.05	3271.95
**New Zealand**					
Cereals	68.97	2036.14	3085.69	15550.41	79216.4
Fiber	0	0	0	0	0
Fruit	1.58	46.68	70.74	356.51	1816.11
Livestock	815.98	22832.21	32863.29	141636.16	516588.41
Oil crops	0.74	21.9	33.18	167.22	851.84
Pulses	5.04	148.88	225.63	1137.06	5792.39
Roots & tubers	5.29	156.15	236.64	1192.53	6074.96
Sugar crops	0	0	0	0	0
Vegetables	0.54	15.88	24.06	121.26	617.71
**Nicaragua**					
Cereals	9355.52	19698.33	15111.28	9166.11	10021.17
Fiber	154.17	328.81	262.95	158.29	171.25
Fruit	199.09	430.4	358.82	214.15	228.87
Livestock	4594.35	10739.66	11476.51	7912.47	10081.14
Oil crops	334.08	712.62	570.24	343.3	371.43
Pulses	8139.28	15200.62	6292.52	3373.23	3021.61
Roots & tubers	257.01	547.25	434.82	260.54	280
Sugar crops	0	0	0	0	0
Vegetables	17.29	37.28	30.82	18.37	19.59
**Niger**					
Cereals	149303.24	207061.67	15698.89	45978.9	0
Fiber	1485.7	1970.23	139.52	0.32	0
Fruit	56.32	81.77	6.59	27.39	0
Livestock	28377.22	38091.94	2734.96	2981.33	0
Oil crops	2914.18	4004.68	299.02	638.82	0
Pulses	1166.58	1571.3	113.93	218.4	0
Roots & tubers	1076.83	1467.37	107.87	274.48	0
Sugar crops	0	0	0	0	0
Vegetables	1592.17	2165.34	157.51	400.09	0
**Nigeria**					
Cereals	856746.65	1474023.44	144203.3	40444.72	0
Fiber	53912.64	101905.58	10887.25	48.36	0
Fruit	7331	14731.77	1660.93	2307.95	0

Livestock	63944.34	114964.24	11952.06	47293.73	0
Oil crops	73086.38	134874.6	14069.36	13408.76	0
Pulses	1522.67	2642.07	260.44	1.06	0
Roots & tubers	274634.62	563307.32	63800.71	88461.99	0
Sugar crops	0	0	0	0	0
Vegetables	10130.33	14945.8	1224.11	1109.78	0
**Norway**					
Cereals	6899.36	50959.55	36183.35	67547.84	6785.11
Fiber	0	0	0	0	0
Fruit	1.19	8.73	6.18	11.48	1.14
Livestock	1855.72	21771.65	20063.16	45688.48	6207.61
Oil crops	153.34	1126.86	796.87	1481.76	147.71
Pulses	0	0	0	0	0
Roots & tubers	211.75	1565.07	1111.93	2077	208.85
Sugar crops	0	0	0	0	0
Vegetables	4.54	33.33	23.57	43.82	4.37
**Oman**					
Cereals	161.03	744.89	276.29	134.03	23.76
Fiber	0	0	0	0	0
Fruit	531.02	2456.45	911.14	441.98	78.35
Livestock	956.69	4425.5	1641.5	796.27	141.15
Oil crops	0	0	0	0	0
Pulses	0	0	0	0	0
Roots & tubers	12.38	57.26	21.24	10.3	1.83
Sugar crops	0	0	0	0	0
Vegetables	46.31	214.21	79.45	38.54	6.83
**Pakistan**					
Cereals	1041610.49	2456659.5	311174.17	59988.24	0
Fiber	503797.02	1309561.54	171120.29	33191	0
Fruit	5584.37	13233.19	1687.89	322.82	0
Livestock	466219.37	804233.46	87981.81	16814.96	0
Oil crops	48411.89	111051.75	13971.78	2676.37	0
Pulses	14157.77	32227.07	4038.6	774.63	0
Roots & tubers	9034.39	19439.62	2363.98	458.09	0
Sugar crops	332.64	780.11	99.12	18.94	0
Vegetables	5778.9	14348.08	1863.11	355.89	0
**Panama**					
Cereals	3480.33	7810.73	6863.37	3116.67	1833.54
Fiber	0	0	0	0	0
Fruit	533.94	1388.14	1682.18	797.08	535.66
Livestock	6282.73	12629.49	7599.96	3406.58	1914.3
Oil crops	33.89	88.58	108.69	52.44	37.05
Pulses	111.64	278.5	322.06	175.54	162.1
Roots & tubers	104.54	280.01	353.45	160.08	93.35
Sugar crops	0	0	0	0	0
Vegetables	49.21	124.77	144.04	66.68	41.79

**Papua New Guinea**					
Cereals	363.79	433.29	152.78	80.33	0
Fiber	0	0	0	0	0
Fruit	1943.32	2841.07	1095.69	594.92	0
Livestock	4743.38	3821.61	1021.16	471.8	0
Oil crops	4722.39	6903.97	2662.59	1445.68	0
Pulses	50.22	73.42	28.31	15.37	0
Roots & tubers	3573.94	5091.57	1944.29	1052.09	0
Sugar crops	0	0	0	0	0
Vegetables	1.17	1.71	0.66	0.36	0
**Paraguay**					
Cereals	633.76	10794.7	11754.25	31199.02	156320.16
Fiber	364.14	5875.58	5857.21	10638.27	29735.22
Fruit	7.84	137.26	156.37	428.4	1889.74
Livestock	603.16	9714.17	9648.84	17353.03	48513.22
Oil crops	1098.97	26974.88	43455.5	218522.97	1434091.03
Pulses	131.34	2076.86	1993.84	3126.08	6988.74
Roots & tubers	113.14	2093.11	2559.36	8366.28	43187.27
Sugar crops	0	0	0	0	0
Vegetables	4.19	72.83	82.31	197.51	584.63
**Peru**					
Cereals	4165.91	64536.95	59408.44	75908.03	96568.06
Fiber	961.99	14899.13	13725.66	17561.88	21959.95
Fruit	262.59	4085.19	3798.14	5030.84	6508.88
Livestock	3199.79	48967.46	43945.87	49754.46	45793.97
Oil crops	25.24	390.75	359.58	458.22	573.67
Pulses	420.75	6459.03	5835.25	6759.47	5909.85
Roots & tubers	813.95	12703.05	11892.36	16274.42	23158.27
Sugar crops	0	0	0	0	0
Vegetables	109.13	1676.32	1515.9	1785.18	1878.2
**Philippines**					
Cereals	274522.99	681492.34	52713.08	465243.46	0
Fiber	220.81	497.12	34.4	242.44	0
Fruit	10469.63	27024.25	2115.2	13576.22	0
Livestock	98044.25	222062.22	14665.36	18357.46	0
Oil crops	50770.05	131058.78	10259.3	65874.85	0
Pulses	1361.53	3514.39	275.07	1765.53	0
Roots & tubers	6333.66	17062.21	1356.42	3605.9	0
Sugar crops	0	0	0	0	0
Vegetables	480.3	1239.74	97.04	622.81	0
**Poland**					
Cereals	140396.65	563023.76	258404.58	880790.27	340486.51
Fiber	0	0	0	0	0
Fruit	361.24	1350.46	545.72	1478.34	491.65
Livestock	73214.04	273338.21	111201.84	301052.34	98659.46

Oil crops	12837.89	85130.15	63707.76	327057.9	148377.67
Pulses	1905.75	7124.8	2877.14	7682.97	2518.34
Roots & tubers	18609.6	63229.85	20784.62	42843.12	11801.44
Sugar crops	6614.77	31019.95	17453.57	67135.86	26824.24
Vegetables	1062.96	3882.59	1510.6	4117.02	1397.89
**Portugal**					
Cereals	5049.34	20216.84	9232.76	36119.79	15437.62
Fiber	0	0	0	0	0
Fruit	1091.39	3582.33	1084.73	2326.09	725.76
Livestock	19212.02	66584.17	22808.94	48027.96	13237.33
Oil crops	100.96	428.59	217.9	1262.6	634.35
Pulses	45.64	171.69	71.22	296.62	134.24
Roots & tubers	1261.78	4415.81	1477.08	2086.62	237.22
Sugar crops	70.54	654.63	574.44	2542.97	1029.28
Vegetables	629.74	2369.44	983.36	4095.02	1852.79
**Qatar**					
Cereals	0	0	0	0	0
Fiber	0	0	0	0	0
Fruit	0	0	0	0	0
Livestock	299.78	1386.76	514.37	249.52	44.23
Oil crops	0	0	0	0	0
Pulses	0	0	0	0	0
Roots & tubers	0	0	0	0	0
Sugar crops	0	0	0	0	0
Vegetables	0	0	0	0	0
**Republic of Korea**					
Cereals	197924.29	272270.36	8403.15	2345.55	0
Fiber	0	0	0	0	0
Fruit	1377.91	1891.8	58.19	16.22	0
Livestock	141630.37	193629.05	5905.53	1638.08	0
Oil crops	24779.77	32946.56	950.29	253.78	0
Pulses	740.09	1016.1	31.25	8.71	0
Roots & tubers	5827.3	7938.19	240.49	66.41	0
Sugar crops	0	0	0	0	0
Vegetables	5737.99	7877.97	242.31	67.54	0
**Romania**					
Cereals	63846.41	289093.56	160324.42	614324.15	241770.45
Fiber	0	0	0	0	0
Fruit	197.75	1028.65	674.48	3270.26	1437.37
Livestock	23783.14	98138.51	46653.81	148442.33	53724.14
Oil crops	10131.26	52391	33490.88	148683.21	62767.28
Pulses	1001.89	4031.43	1804.46	4930.28	1604.85
Roots & tubers	4743.81	19144.92	8605.11	20127.03	5287.57
Sugar crops	870.43	3258.45	1318.64	3963.32	1460.07
Vegetables	509.23	2140.87	1082.1	3925.65	1516.72
**Russian Federation**					

Cereals	446048.31	1900179.33	961221.42	4604401.1	2138888.12
Fiber	0	0	0	0	0
Fruit	149.62	818.92	539	3355.18	1682.94
Livestock	93089.65	417833.08	224067.98	845991.05	337028.07
Oil crops	38541.14	180280.17	102291.39	554352.86	267514.83
Pulses	12727.94	55010.15	27582.49	110899.47	47475.75
Roots & tubers	39652.1	155701.77	68098.55	220916.42	84440.61
Sugar crops	15962.17	60310.44	26115.35	153301.02	78740.68
Vegetables	1755.64	7277.72	3490.94	14518.71	6368.46
**Rwanda**					
Cereals	12627.15	20097.06	1836.61	6.56	0
Fiber	0	0	0	0	0
Fruit	7997.88	13138.81	1254.15	4.69	0
Livestock	5009.85	6859.07	495.48	1.25	0
Oil crops	4679.32	5967.08	380.22	0.66	0
Pulses	20848	32699.42	2947.92	10.22	0
Roots & tubers	14302.67	20502.66	1609.99	4.57	0
Sugar crops	0	0	0	0	0
Vegetables	154.54	185.89	9.15	0	0
**Saint Lucia**					
Cereals	0	0	0	0	0
Fiber	0	0	0	0	0
Fruit	0	0	0	0	0
Livestock	0	0	0	0	0
Oil crops	0	0	0	0	0
Pulses	0	0	0	0	0
Roots & tubers	0	0	0	0	0
Sugar crops	0	0	0	0	0
Vegetables	0	0	0	0	0
**Saint Vincent and the Grenadines**					
Cereals	0	0	0	0	0
Fiber	0	0	0	0	0
Fruit	0	0	0	0	0
Livestock	0	0	0	0	0
Oil crops	0	0	0	0	0
Pulses	0	0	0	0	0
Roots & tubers	0	0	0	0	0
Sugar crops	0	0	0	0	0
Vegetables	0	0	0	0	0
**Saudi Arabia**					
Cereals	59666.27	223685.29	70789.89	34332.04	5964.05
Fiber	0	0	0	0	0
Fruit	2773.28	9845.6	2919.98	1430.83	245.18
Livestock	17712.3	80514.43	27688.26	14158.36	2428.06
Oil crops	264.25	559.66	68.6	27.18	3.93

Pulses	73.32	290.43	95.51	46.67	8.13
Roots & tubers	1053.52	3922.59	1250.65	599.85	104.87
Sugar crops	0	0	0	0	0
Vegetables	818.07	2594.2	687.59	332.48	56.29
**Senegal**					
Cereals	28749.32	60549.68	7151.99	33.78	0
Fiber	3235.13	10651.34	1551.45	8.65	0
Fruit	190.24	256.82	19.24	0.05	0
Livestock	6830.56	14741.18	1758.29	8.47	0
Oil crops	1610.77	1979.84	135.48	0.17	0
Pulses	8.64	10.48	0.65	0	0
Roots & tubers	1391.27	1713.05	106.96	0.13	0
Sugar crops	0	0	0	0	0
Vegetables	594.39	1153.2	127.83	0.58	0
**Serbia**					
Cereals	55445.41	203366.97	79668.37	208890.33	67817.3
Fiber	0	0	0	0	0
Fruit	375.88	1210.47	359.02	819.05	267.12
Oil crops	13885.8	53769.62	23259.36	65855.25	22022.01
Pulses	1719.6	5848.7	1976.6	4745.57	1506.52
Roots & tubers	1037.77	3882.84	1583.54	4570.72	1602.55
Sugar crops	5079.62	16017.81	4494.52	10446.24	3602.99
Vegetables	278.44	969.52	342.39	802.39	242.49
**Sierra Leone**					
Cereals	18327.38	40952.56	4964.48	23.79	0
Fiber	0	0	0	0	0
Fruit	70.99	216.72	30.42	0.17	0
Livestock	1242.43	1763.77	142.43	0.41	0
Oil crops	144.99	318.06	38.44	0.18	0
Pulses	1022.8	3054.3	429.4	2.3	0
Roots & tubers	4919.94	11804.23	1482.18	7.58	0
Sugar crops	0	0	0	0	0
Vegetables	46.28	101.55	12.27	0.06	0
**Slovakia**					
Cereals	26082.56	99998.14	43437.75	131708.79	46197.89
Fiber	0	0	0	0	0
Fruit	58.29	182.08	49.05	106.72	36.13
Livestock	3198.51	16698.46	10033.94	31930.22	10939.09
Oil crops	9918.21	36967.14	15506.54	47176.83	16627.98
Pulses	442.78	1678.86	721.22	2218.06	786.69
Roots & tubers	273.15	1190.55	609.58	1707.32	521.6
Sugar crops	1014.46	4317.05	2192.48	6626.73	2171.91
Vegetables	57.76	216.28	90.36	271.25	95.63
**Slovenia**					
Cereals	7355.7	24588.64	7225.4	8005.39	372.04
Fiber	0	0	0	0	0

Fruit	151.88	532.53	172.47	202.64	9.42
Livestock	7029.38	22516.08	5985	6173.62	286.94
Oil crops	499.09	1671.89	493.62	548.52	25.49
Pulses	252.28	864.91	268.05	307.02	14.28
Roots & tubers	280.16	971.23	307.73	357.09	16.6
Sugar crops	332.62	1113.99	328.75	365.26	16.98
Vegetables	14.52	49.79	15.43	17.68	0.82
**Somalia**					
Cereals	8740.48	12972.98	1034.4	3.37	0
Fiber	809.76	1237.59	103.25	0.37	0
Fruit	207.64	317.34	26.47	0.09	0
Livestock	27052.56	52292.16	5732.41	25.71	0
Oil crops	3443.28	5262.46	439.02	1.55	0
Pulses	1287.08	2149.28	202.36	0.8	0
Roots & tubers	356.71	545.17	45.48	0.16	0
Sugar crops	0	0	0	0	0
Vegetables	30.91	47.25	3.94	0.01	0
**South Africa**					
Cereals	46508.73	256189.29	42586.45	519660.04	0
Fiber	1199.7	6538.16	1086.41	3653.14	0
Fruit	5595.83	13801.1	1794.58	5733.15	0
Livestock	52328.28	156242.94	21881.95	173918.99	0
Oil crops	21690.5	74076.65	10896.61	56850.46	0
Pulses	551.59	4545.85	798.9	5662.53	0
Roots & tubers	3237.24	13922.91	2191.28	9515.79	0
Sugar crops	0	0	0	0	0
Vegetables	965.47	3841.32	593.49	2361.18	0
**Spain**					
Cereals	115815.17	527879.96	282957.53	945828.18	345688.28
Fiber	1131.04	6789.58	4904.29	35548.77	18550.39
Fruit	5578.88	20752.72	8258.76	23655.65	8446.52
Livestock	113555.86	400020.36	141425.49	272337.78	62551.53
Oil crops	3876.78	18013.83	10160.88	51850.45	24351.41
Pulses	2555.36	10319.9	4758.83	17277.29	7022.31
Roots & tubers	2515.08	11078.11	5439.37	14713.5	4688.1
Sugar crops	1860.96	11732.7	8363.15	36953.26	15587.22
Vegetables	2725.8	11007.43	5075.36	18433.26	7494.27
**Sri Lanka**					
Cereals	62830.98	118912.35	13630.8	39340.64	0
Fiber	0	0	0	0	0
Fruit	1019.95	2264.82	280.28	1049.84	0
Livestock	9328.61	13627.11	1312.79	1516.43	0
Oil crops	10245.32	19104.35	2183.28	5891.82	0
Pulses	341.26	770.55	95.7	426.45	0
Roots & tubers	999.59	2132.08	257.4	844.95	0
Sugar crops	0	0	0	0	0

Vegetables	392.8	873.51	108.22	410.6	0
**Sudan (former)**					
Cereals	50268.17	267638.78	97270.97	113300.75	151803.63
Fiber	9546.36	36188.88	10874.17	13084.56	18299.9
Fruit	1235.6	4494.98	1307.94	1613.5	2299.36
Livestock	50576.88	192082.21	58091.56	51214.81	54978.19
Oil crops	8900.53	26976.97	6548.33	7152.78	9559.41
Pulses	909.79	3324.83	969.35	1092.48	1463.89
Roots & tubers	1028.31	3608.97	1018.49	1196.22	1656.34
Sugar crops	0	0	0	0	0
Vegetables	1969.1	6921.46	1954.68	2194.37	2943.32
**Suriname**					
Cereals	0	89.97	223.92	1556.18	10625.21
Fiber	0	0	0	0	0
Fruit	0	4.44	11.05	76.79	524.31
Livestock	0	12.48	31.07	215.93	1474.35
Oil crops	0	0.76	1.89	13.14	89.74
Pulses	0	0.03	0.09	0.6	4.12
Roots & tubers	0	0.39	0.98	6.8	46.45
Sugar crops	0	0	0	0	0
Vegetables	0	0.04	0.11	0.74	5.06
**Swaziland**					
Cereals	1051.33	2188.74	253.81	78.84	0
Fiber	174.7	510.66	71.2	54.93	0
Fruit	134.28	296.17	35.58	9.26	0
Livestock	1143.87	2416.1	280.16	754.14	0
Oil crops	0	0	0	0	0
Pulses	63.06	166.16	21.94	4.47	0
Roots & tubers	37.24	76.27	8.72	1.78	0
Sugar crops	0	0	0	0	0
Vegetables	8.17	17.88	2.14	0.59	0
**Sweden**					
Cereals	15920.23	129023.28	98136.63	281851.93	82276.91
Fiber	0	0	0	0	0
Fruit	1.12	10.05	8.17	23.22	6.34
Livestock	4476.63	36001.86	27241.14	82871.03	26154.93
Oil crops	2347.59	17763.21	12851.3	35068.76	9933.77
Pulses	283.33	2552.94	2075.35	5900.23	1609.55
Roots & tubers	479.28	3414.14	2352.23	5315.9	1134.66
Sugar crops	1163.93	8052.17	5405.81	11136.5	1960.86
Vegetables	9.92	89.34	72.63	206.49	56.33
**Switzerland**					
Cereals	6208.53	33680.36	22035.54	47411.4	6481.35
Fiber	0	0	0	0	0
Fruit	64.04	408.98	293.17	626.94	82.4
Livestock	6875.57	52801.31	39750.77	80354.2	9385.09

Oil crops	2136.76	8660.03	4175.66	8241.82	1115.94
Pulses	216.12	1228.96	832.85	1806.71	247.22
Roots & tubers	158.85	1658.53	1454.74	3211.3	419.93
Sugar crops	371.18	4088.58	3672.01	8230.24	1096.96
Vegetables	11.23	63.97	43.44	94.32	12.92
**Syrian Arab Republic**					
Cereals	143134.72	407414.37	96342.47	59774.56	41784.2
Fiber	39010.85	130845.62	37365.69	28422.42	27163.58
Fruit	952.81	3283.77	961.52	744.77	726.19
Livestock	26839.43	94494.6	27729.91	17819.63	12020.32
Oil crops	1399.26	4550.79	1259.06	949.14	900.14
Pulses	363.63	1214.32	345	263.85	254.1
Roots & tubers	1432.06	4562.45	1232.83	916.97	854.43
Sugar crops	2948.69	8879.42	2273.61	1300.01	717.3
Vegetables	1389.66	4827.03	1426.82	1059.22	973.2
**Tajikistan**					
Cereals	11321.26	50317.32	16832.91	14679.69	15260.93
Fiber	14880.75	65486.07	21788.5	19275.04	20411.2
Fruit	106.46	466.2	154.6	136.68	144.62
Livestock	2490.26	11745.54	4030	4496.06	5867.27
Oil crops	44.79	223.1	80.5	57.32	43.81
Pulses	302.28	1433.36	500.99	395.26	359.67
Roots & tubers	938.32	4182.61	1399.93	1231.44	1291.76
Sugar crops	0	0	0	0	0
Vegetables	400.85	1758.46	583.9	515.01	543.42
**Thailand**					
Cereals	594961.73	1670471.61	137602.74	59465.83	0
Fiber	836.33	2515.18	217.67	95.24	0
Fruit	6187.05	15647.38	1217.93	521.16	0
Livestock	63025.1	277021.18	28081.77	12645.37	0
Oil crops	26944.72	82849.92	7313.49	3218.02	0
Pulses	8302.39	16740.83	1047.7	421.82	0
Roots & tubers	48788.07	180854.95	17390.89	7773.89	0
Sugar crops	0	0	0	0	0
Vegetables	436.81	1108.34	86.46	37.01	0
**The former Yugoslav Republic of Macedonia**					
Cereals	3879.93	20608.56	12671.34	25150.47	3072.32
Fiber	0	0	0	0	0
Fruit	122	621.54	370.96	736.29	91.12
Livestock	1971.07	7517	3045.22	4868.2	493.92
Oil crops	60.8	282.95	152.05	283.79	32.89
Pulses	106.13	539.71	320.49	631.7	77.35
Roots & tubers	172.13	917.41	551.81	1046.66	118.2
Sugar crops	21.07	115.16	72.9	147.27	18.36
Vegetables	85.65	435.15	258.88	512.67	63.27

**Togo**					
Cereals	22587.15	39684.14	3957.33	16.39	0
Fiber	7351.08	8889.93	505.24	0.33	0
Fruit	73.08	142.18	15.68	0.07	0
Livestock	2874.86	4074.62	313.69	0.89	0
Oil crops	200.81	345.02	34.84	0.14	0
Pulses	3842.73	7318.06	798.42	3.54	0
Roots & tubers	5547.82	9917.58	1012.83	4.21	0
Sugar crops	0	0	0	0	0
Vegetables	18.19	26.94	2.37	0.01	0
**Trinidad and Tobago**					
Cereals	0	23.13	92.56	88.06	144.27
Fiber	0	0	0	0	0
Fruit	0	9.13	36.51	34.74	56.91
Livestock	0	0	0	0	0
Oil crops	0	20.72	82.9	78.86	129.21
Pulses	0	0.88	3.53	3.36	5.51
Roots & tubers	0	1.32	5.27	5.01	8.21
Sugar crops	0	0	0	0	0
Vegetables	0	1.23	4.91	4.67	7.65
**Tunisia**					
Cereals	27971.88	113742.92	36171.1	36182.94	43919.97
Fiber	70.15	308.37	102.69	108.67	137.42
Fruit	627.42	1938.74	496.72	464.07	556.45
Livestock	7397.41	37492.14	13597.19	9389.04	6667.92
Oil crops	817.34	3932.22	1364.79	1393.4	1688.94
Pulses	325.59	1431.23	476.56	504.51	638.13
Roots & tubers	619.18	2071.54	555.26	765.72	1164.55
Sugar crops	0	0	0	0	0
Vegetables	541.16	2550.19	887.71	1768.07	3096.95
**Turkey**					
Cereals	625556.76	2006697.65	528514.19	460599.07	505979.74
Fiber	103251.15	383783.15	118484.42	86941.86	76120.25
Fruit	7348.76	22009.24	5305.77	4518.49	4852.08
Livestock	124414.69	378325.68	93344.54	61279.37	43528.76
Oil crops	19745.71	77882.26	24643.69	13902.13	5598.65
Pulses	6382.51	20493.81	5394.63	3467.36	2312.66
Roots & tubers	14606.06	35761.51	6380.27	4161.13	3395.91
Sugar crops	36212.67	96831.23	19969.88	12421.88	8319.48
Vegetables	16697.84	50417.01	12318.41	9662.19	9377.5
**Turkmenistan**					
Cereals	8611.02	143936.02	67640.22	83925.56	114993.25
Fiber	5921.21	98199.4	46114.63	57818.88	79808.24
Fruit	47.32	784.74	368.52	461.94	637.51
Livestock	2489.47	32584.82	14970.23	19914.11	28700.99
Oil crops	0	0	0	0	0

Pulses	55.78	927.09	435.45	545.27	751.92
Roots & tubers	54.17	1170.88	560.27	607.5	745.79
Sugar crops	62.42	992.08	464.24	577.09	792.69
Vegetables	68.32	1133.8	532.46	667.44	921.02
**Uganda**					
Cereals	73204.05	129762.21	13271.46	54.17	0
Fiber	6219.23	12283.39	1371.12	6.16	0
Fruit	26358.07	49280.88	5276.53	23.26	0
Livestock	23910.42	52801.98	6373.47	30.97	0
Oil crops	42631.39	62660.2	5236.44	16.04	0
Pulses	29854.46	53911.69	5637.01	23.03	0
Roots & tubers	31302.67	60208.13	6510.14	29.28	0
Sugar crops	0	0	0	0	0
Vegetables	678.93	1295.63	141.05	0.61	0
**Ukraine**					
Cereals	285215.72	1105015.24	479233.06	1472007.46	532314.56
Fiber	0	0	0	0	0
Fruit	282.81	1111.41	492.38	1611.79	612.1
Livestock	62759.14	233087.87	93646.34	255963.49	85621.08
Oil crops	73489.36	287251.02	125767.4	401090.57	150039.43
Pulses	5960.13	28352.36	16200.28	57318.54	21331.91
Roots & tubers	18861.28	82373.04	41728.68	114981.73	35111.41
Sugar crops	11474.57	52017.61	27676.37	97893.24	37668.16
Vegetables	1418.33	5460.87	2348.69	7819.8	3033.26
**United Arab Emirates**					
Cereals	0.44	2.03	0.75	0.36	0.06
Fiber	0	0	0	0	0
Fruit	1516.21	7013.76	2601.54	1261.97	223.7
Livestock	647.11	2993.42	1110.32	538.6	95.47
Oil crops	0	0	0	0	0
Pulses	0	0	0	0	0
Roots & tubers	12.91	59.71	22.15	10.74	1.9
Sugar crops	0	0	0	0	0
Vegetables	107.34	496.54	184.18	89.34	15.84
**United Kingdom**					
Cereals	135535.41	668307.85	383574.74	1159366.17	379169.87
Fiber	0	0	0	0	0
Fruit	13.08	110.56	86.46	278.25	91.72
Livestock	51848.66	286671.21	168883.64	370500.51	81276.08
Oil crops	37367.97	181658.45	103233.78	301039.33	94356.47
Pulses	1496.02	12644.21	9888.35	31823.16	10489.41
Roots & tubers	1678.78	15459.42	12725.01	43525.58	14876
Sugar crops	1637.05	15314.3	12714.45	47235.74	17398.32
Vegetables	95.11	803.83	628.63	2023.09	666.84
**United Republic of Tanzania**					

Cereals	114441.56	282713.86	36177.01	184.25	0
Fiber	17982.97	49990	6786.15	35.9	0
Fruit	6431.31	17654.98	2366.94	12.44	0
Livestock	34412.6	66956.8	7212.18	32.53	0
Oil crops	13425.66	35952.95	4790.95	25.12	0
Pulses	49414.22	95177.67	10254.51	45.64	0
Roots & tubers	20574.05	60821.71	8485.46	45.6	0
Sugar crops	0	0	0	0	0
Vegetables	360.76	1066.31	148.17	0.79	0
**United States of America**					
Cereals	2329.91	1208449.74	2590627.52	12191574.64	12322160.29
Fiber	188.42	134119.5	317605.83	1610074.64	1706471.85
Fruit	38.48	9659.09	15087.58	45704.51	41791.46
Livestock	2941.65	802336.54	1289384.69	3592711.74	2414063.76
Oil crops	1822.29	1170286.44	2700874.65	12996661.04	13128417.92
Pulses	50.91	21777.02	41648.9	168959.24	146082.77
Roots & tubers	72.75	26652.44	46049.55	148349.98	87382.96
Sugar crops	7.72	10020.49	27625.81	160729.2	187613.9
Vegetables	17	7842.62	15641.77	61875.88	50666.28
**Uruguay**					
Cereals	278.34	6545.48	10293.97	41407.99	182206.78
Fiber	0	0	0	0	0
Fruit	3.64	85.21	133.85	485.88	1513.59
Livestock	149.68	3672.89	5929.6	24998.3	116154.59
Oil crops	94.3	2433.28	4026.28	25934.98	221405.84
Pulses	1.9	45.01	71.08	266.44	922.85
Roots & tubers	2.18	62.21	107.79	484.78	2252.78
Sugar crops	0	0	0	0	0
Vegetables	0.52	14.33	24.44	107.99	497.18
**Uzbekistan**					
Cereals	63788.55	380542.64	147768.53	136553.78	150645.97
Fiber	87047.13	519319.21	201654.18	186320.45	205505.61
Fruit	469.77	2797.99	1085.87	1004.74	1110.03
Livestock	17382.56	127684.33	52568.5	38288.23	28974.18
Oil crops	178.78	1068.54	414.85	358.54	365.37
Pulses	52.23	302.49	116.3	99.4	100.29
Roots & tubers	1154.73	6839.75	2650.11	2456.44	2721.31
Sugar crops	0	0	0	0	0
Vegetables	1307.09	7772.54	3015.09	2789.62	3083.05
**Venezuela (Bolivarian Republic of)**					
Cereals	1153.89	19560.28	21234.85	49795.66	179784.34
Fiber	34.98	597.22	653.35	1532.05	5224.56

Fruit	33.49	603.22	709.12	1956.95	7455.72
Livestock	2228.21	34835.09	32566.48	47704.09	115079.1
Oil crops	85.45	1453.82	1584.38	3712.43	12997.56
Pulses	43.16	708	728.48	1401.37	3576.56
Roots & tubers	43.77	780.99	907.06	2456.41	9391.52
Sugar crops	1.14	19.41	21.2	49.63	170.03
Vegetables	18.26	311.76	341.46	802.39	2735.9
**Viet Nam**					
Cereals	420231.35	1484182.13	139001.14	763572.59	0
Fiber	2091.5	4798.39	346.26	1258.98	0
Fruit	3899.31	9040.8	655.72	2468.18	0
Livestock	132393.38	203083.87	8124.82	76525.83	0
Oil crops	30543.54	74590.47	5807.47	11173.98	0
Pulses	10710.99	23161.15	1611.09	2317.81	0
Roots & tubers	35734.75	63891.6	3478.66	5278.92	0
Sugar crops	0	0	0	0	0
Vegetables	650.62	1518.4	111.02	329.72	0
**Yemen**					
Cereals	11684.79	50024.89	16448.43	8439.48	1437.48
Fiber	1038.86	4089.86	1272.16	647.85	109.88
Fruit	364.89	1730.78	598.93	311.43	53.05
Livestock	11905.92	28957.5	5252.59	2311.75	374.79
Oil crops	609.59	2194.3	636.65	321.22	54.15
Pulses	251.48	990.03	307.95	156.83	26.6
Roots & tubers	465.44	1993.04	652.37	336.01	57.08
Sugar crops	0	0	0	0	0
Vegetables	368.01	1972.57	724.49	379.08	64.82
**Zambia**					
Cereals	22293.54	55212.95	7178.94	14241.13	0
Fiber	5334.31	14425.88	1971.75	21785.04	0
Fruit	9.38	15.11	1.42	2.67	0
Livestock	6591.53	11386.66	1132.09	3393.65	0
Oil crops	4811	16347.12	2412.5	2280.15	0
Pulses	435.54	726.5	70.4	166.98	0
Roots & tubers	5099.16	7235.5	541.89	350.77	0
Sugar crops	0	0	0	0	0
Vegetables	146.7	241.19	23.03	56.23	0
**Zimbabwe**					
Cereals	17890.23	81979.52	13088	4235.74	0
Fiber	8123.75	48647.39	8168.89	4499	0
Fruit	153.88	1057.3	181.37	20.92	0
Livestock	15355.03	24045.43	2199.8	147.6	0
Oil crops	8577.44	19371.43	2405.62	1037.66	0
Pulses	1019.48	3753.55	569.48	122.57	0
Roots & tubers	851.73	1836.15	221.96	122.56	0
Sugar crops	0	0	0	0	0

Vegetables	2.54	144.01	27.62	0.42	0

	Vitamin A production (T)				
	< 2 ha	2 - 20 ha	20 - 50 ha	50 - 200 ha	> 200 ha
**Afghanistan**					
Cereals	0.01	0.02	0	0.01	0
Fiber	0	0	0	0	0
Fruit	0	0.01	0	0	0
Livestock	0.26	0.33	0.03	0.01	0
Oil crops	0	0	0	0	0
Pulses	0	0	0	0	0
Roots & tubers	0	0	0	0	0
Sugar crops	0	0	0	0	0
Vegetables	0	0	0	0	0
**Albania**					
Cereals	0	0.01	0	0	0
Fiber	0	0	0	0	0
Fruit	0	0	0	0	0
Livestock	0.08	0.24	0.05	0.04	0
Oil crops	0	0	0	0	0
Pulses	0	0	0	0	0
Roots & tubers	0	0	0	0	0
Sugar crops	0	0	0	0	0
Vegetables	0.01	0.03	0.01	0.01	0
**Algeria**					
Cereals	0	0	0	0	0
Fiber	0	0	0	0	0
Fruit	0.01	0.05	0.01	0.01	0.01
Livestock	0.12	0.51	0.16	0.13	0.12
Oil crops	0.01	0.02	0	0.01	0.01
Pulses	0	0	0	0	0
Roots & tubers	0	0	0	0	0
Sugar crops	0	0	0	0	0
Vegetables	0.03	0.17	0.06	0.03	0.01
**Angola**					
Cereals	0.02	0.03	0	0	0
Fiber	0	0	0	0	0
Fruit	0.01	0.01	0	0	0
Livestock	0.03	0.04	0	0.01	0
Oil crops	0	0	0	0	0
Pulses	0	0	0	0	0
Roots & tubers	1.21	2.33	0.26	0.19	0
Sugar crops	0	0	0	0	0
Vegetables	0	0	0	0	0
**Argentina**					
Cereals	0	0.02	0.04	0.2	1.35

Fiber	0	0	0	0	0
Fruit	0	0.01	0.01	0.03	0.2
Livestock	0.02	0.33	0.33	0.7	2.91
Oil crops	0	0.01	0.01	0.06	0.43
Pulses	0	0	0	0	0.01
Roots & tubers	0.01	0.13	0.15	0.38	1.13
Sugar crops	0	0	0	0	0
Vegetables	0	0.01	0.01	0.04	0.21
**Armenia**					
Cereals	0	0	0	0	0
Fiber	0	0	0	0	0
Fruit	0	0.01	0	0	0
Livestock	0.03	0.14	0.05	0.03	0
Oil crops	0	0	0	0	0
Pulses	0	0	0	0	0
Roots & tubers	0	0	0	0	0
Sugar crops	0	0	0	0	0
Vegetables	0.01	0.06	0.03	0.01	0
**Australia**					
Cereals	0	0	0	0.01	0.09
Fiber	0	0	0	0	0
Fruit	0	0	0	0.01	0.1
Livestock	0	0.01	0.03	0.28	3.45
Oil crops	0	0	0	0	0.02
Pulses	0	0	0	0	0.02
Roots & tubers	0	0	0	0.02	0.21
Sugar crops	0	0	0	0	0
Vegetables	0	0	0	0.01	0.13
**Austria**					
Cereals	0.01	0.05	0.03	0.06	0.01
Fiber	0	0	0	0	0
Fruit	0	0.01	0	0.01	0
Livestock	0.06	0.36	0.25	0.5	0.06
Oil crops	0	0	0	0	0
Pulses	0	0	0	0	0
Roots & tubers	0	0	0	0	0
Sugar crops	0	0.02	0.01	0.01	0
Vegetables	0	0.01	0	0	0
**Azerbaijan**					
Cereals	0	0.01	0	0	0
Fiber	0	0	0	0	0
Fruit	0	0	0	0	0
Livestock	0.07	0.25	0.07	0.06	0.06
Oil crops	0	0	0	0	0
Pulses	0	0	0	0	0
Roots & tubers	0	0	0	0	0

Sugar crops	0	0	0	0	0
Vegetables	0.03	0.09	0.02	0.02	0.01
**Bangladesh**					
Cereals	0.01	0.03	0	0.01	0
Fiber	0	0	0	0	0
Fruit	0.01	0.02	0	0.01	0
Livestock	0.5	0.92	0.1	0.14	0
Oil crops	0	0	0	0	0
Pulses	0	0.01	0	0	0
Roots & tubers	0.39	0.83	0.1	0.25	0
Sugar crops	0	0	0	0	0
Vegetables	0.01	0.03	0	0	0
**Belarus**					
Cereals	0	0.02	0.01	0.02	0.01
Fiber	0	0	0	0	0
Fruit	0	0	0	0	0
Livestock	0.16	0.62	0.28	0.86	0.32
Oil crops	0	0	0	0	0
Pulses	0	0	0	0	0
Roots & tubers	0	0	0	0	0
Sugar crops	0	0.01	0.01	0.03	0.01
Vegetables	0.01	0.03	0.01	0.03	0.01
**Belgium**					
Cereals	0.01	0.03	0.01	0.01	0
Fiber	0	0	0	0	0
Fruit	0	0	0	0	0
Livestock	0.08	0.37	0.19	0.56	0.19
Oil crops	0	0	0	0	0
Pulses	0	0	0	0	0
Roots & tubers	0	0	0	0	0
Sugar crops	0.01	0.02	0.01	0.04	0.01
Vegetables	0.01	0.03	0.01	0.03	0.01
**Belize**					
Cereals	0	0	0	0	0
Fiber	0	0	0	0	0
Fruit	0	0.01	0.02	0.01	0
Livestock	0	0	0	0	0
Oil crops	0	0	0	0	0
Pulses	0	0	0	0	0
Roots & tubers	0	0	0	0	0
Sugar crops	0	0	0	0	0
Vegetables	0	0	0	0	0
**Benin**					
Cereals	0.03	0.04	0	0	0
Fiber	0	0	0	0	0
Fruit	0	0	0	0	0

Livestock	0.01	0.02	0	0	0
Oil crops	0	0	0	0	0
Pulses	0	0	0	0	0
Roots & tubers	0.2	0.25	0.02	0	0
Sugar crops	0	0	0	0	0
Vegetables	0.02	0.03	0	0	0
**Bhutan**					
Cereals	0	0	0	0	0
Fiber	0	0	0	0	0
Fruit	0	0	0	0	0
Livestock	0.01	0.01	0	0	0
Oil crops	0	0	0	0	0
Pulses	0	0	0	0	0
Roots & tubers	0	0	0	0	0
Sugar crops	0	0	0	0	0
Vegetables	0	0	0	0	0
**Bolivia (Plurinational State of)**					
Cereals	0	0.01	0.01	0.01	0.04
Fiber	0	0	0	0	0
Fruit	0	0.05	0.04	0.04	0.05
Livestock	0	0.03	0.03	0.07	0.14
Oil crops	0	0	0	0	0.01
Pulses	0	0	0	0	0
Roots & tubers	0	0.01	0	0.01	0.02
Sugar crops	0	0	0	0	0
Vegetables	0	0	0	0.01	0.03
**Bosnia and Herzegovina**					
Cereals	0.01	0.03	0.01	0.02	0
Fiber	0	0	0	0	0
Fruit	0	0	0	0	0
Livestock	0.03	0.11	0.05	0.08	0.01
Oil crops	0	0	0	0	0
Pulses	0	0	0	0	0
Roots & tubers	0	0	0	0	0
Sugar crops	0	0	0	0	0
Vegetables	0	0.01	0	0	0
**Botswana**					
Cereals	0	0	0	0	0
Fiber	0	0	0	0	0
Fruit	0	0	0	0	0
Livestock	0	0.02	0	0.01	0
Oil crops	0	0	0	0	0
Pulses	0	0	0	0	0
Roots & tubers	0	0	0	0	0
Sugar crops	0	0	0	0	0

Vegetables	0	0	0	0	0
**Brazil**					
Cereals	0.01	0.15	0.17	0.5	2.85
Fiber	0	0	0	0	0
Fruit	0.01	0.18	0.17	0.28	1.06
Livestock	0.08	1.24	1.25	2.51	8.89
Oil crops	0	0	0.01	0.05	0.48
Pulses	0	0.01	0.01	0.01	0.02
Roots & tubers	0.03	0.44	0.41	0.62	1.4
Sugar crops	0	0	0	0	0
Vegetables	0	0.07	0.08	0.22	0.93
**Brunei Darussalam**					
Cereals	0	0	0	0	0
Fiber	0	0	0	0	0
Fruit	0	0	0	0	0
Livestock	0	0.01	0	0	0
Oil crops	0	0	0	0	0
Pulses	0	0	0	0	0
Roots & tubers	0	0	0	0	0
Sugar crops	0	0	0	0	0
Vegetables	0	0	0	0	0
**Bulgaria**					
Cereals	0	0.02	0.01	0.05	0.02
Fiber	0	0	0	0	0
Fruit	0	0	0	0	0
Livestock	0.06	0.24	0.09	0.23	0.07
Oil crops	0	0	0	0.01	0
Pulses	0	0	0	0	0
Roots & tubers	0	0	0	0	0
Sugar crops	0	0	0	0	0
Vegetables	0	0.02	0.01	0.02	0.01
**Burkina Faso**					
Cereals	0.01	0.02	0	0.03	0
Fiber	0	0	0	0	0
Fruit	0	0	0	0	0
Livestock	0.08	0.1	0.01	0.01	0
Oil crops	0	0	0	0	0
Pulses	0	0	0	0	0
Roots & tubers	0.09	0.18	0.02	0.08	0
Sugar crops	0	0	0	0	0
Vegetables	0	0	0	0	0
**Burundi**					
Cereals	0	0.01	0	0	0
Fiber	0	0	0	0	0
Fruit	0.01	0.02	0	0	0
Livestock	0.01	0.01	0	0	0

Oil crops	0	0	0	0	0
Pulses	0	0	0	0	0
Roots & tubers	1.92	2.32	0.12	0	0
Sugar crops	0	0	0	0	0
Vegetables	0	0	0	0	0
**Cambodia**					
Cereals	0.01	0.02	0	0	0
Fiber	0	0	0	0	0
Fruit	0	0.01	0	0	0
Livestock	0.01	0.03	0	0	0
Oil crops	0	0	0	0	0
Pulses	0	0	0	0	0
Roots & tubers	0.07	0.14	0.01	0	0
Sugar crops	0	0	0	0	0
Vegetables	0	0	0	0	0
**Cameroon**					
Cereals	0.04	0.05	0	0.01	0
Fiber	0	0	0	0	0
Fruit	0.28	0.45	0.04	0.04	0
Livestock	0.05	0.07	0	0	0
Oil crops	0	0	0	0	0
Pulses	0	0	0	0	0
Roots & tubers	0.34	0.59	0.06	0.14	0
Sugar crops	0	0	0	0	0
Vegetables	0.08	0.11	0.01	0	0
**Canada**					
Cereals	0	0.04	0.09	0.4	0.42
Fiber	0	0	0	0	0
Fruit	0	0	0	0.01	0
Livestock	0	0.49	0.75	1.52	0.84
Oil crops	0	0.01	0.02	0.05	0.05
Pulses	0	0.02	0.02	0.08	0.08
Roots & tubers	0	0	0	0	0
Sugar crops	0	0	0	0	0.01
Vegetables	0	0.03	0.05	0.16	0.08
**Central African Republic**					
Cereals	0	0.01	0	0	0
Fiber	0	0	0	0	0
Fruit	0.01	0.02	0	0	0
Oil crops	0	0	0	0	0
Pulses	0	0	0	0	0
Roots & tubers	0.01	0.02	0	0	0
Sugar crops	0	0	0	0	0
Vegetables	0	0	0	0	0
**Chad**					
Cereals	0	0.01	0	0.01	0

Fiber	0	0	0	0	0
Fruit	0	0	0	0	0
Livestock	0.03	0.05	0	0.01	0
Oil crops	0	0	0	0	0
Pulses	0	0	0	0	0
Roots & tubers	0.13	0.21	0.02	0.08	0
Sugar crops	0	0	0	0	0
Vegetables	0	0	0	0	0
**Chile**					
Cereals	0	0.01	0.01	0.02	0.06
Fiber	0	0	0	0	0
Fruit	0	0.01	0.01	0.02	0.07
Livestock	0	0.07	0.09	0.24	0.76
Oil crops	0	0	0	0	0
Pulses	0	0	0	0	0
Roots & tubers	0	0	0	0.01	0.03
Sugar crops	0	0	0	0.01	0.02
Vegetables	0	0.04	0.04	0.08	0.27
**China**					
Cereals	5.64	3.98	0	2.94	0
Fiber	0	0	0	0	0
Fruit	0.95	0.4	0	0.21	0
Livestock	33.1	13.77	0	5.69	0
Oil crops	0.14	0.09	0	0.05	0
Pulses	0.06	0.03	0	0.02	0
Roots & tubers	320.96	91.56	0	29.62	0
Sugar crops	0.04	0.05	0	0.04	0
Vegetables	7.66	3.59	0	1.52	0
**Colombia**					
Cereals	0	0	0.01	0.02	0.11
Fiber	0	0	0	0	0
Fruit	0	0.08	0.1	0.25	0.77
Livestock	0.02	0.38	0.37	0.64	1.74
Oil crops	0	0	0	0	0
Pulses	0	0	0	0	0
Roots & tubers	0	0	0	0.01	0.02
Sugar crops	0	0	0	0	0
Vegetables	0	0.02	0.02	0.04	0.09
**Congo**					
Cereals	0	0	0	0	0
Fiber	0	0	0	0	0
Fruit	0.01	0.02	0	0	0
Livestock	0	0	0	0	0
Oil crops	0	0	0	0	0
Pulses	0	0	0	0	0
Roots & tubers	0.01	0.02	0	0	0

Sugar crops	0	0	0	0	0
Vegetables	0	0	0	0	0
**Costa Rica**					
Cereals	0	0	0	0	0
Fiber	0	0	0	0	0
Fruit	0	0.01	0.03	0.03	0.05
Livestock	0	0.02	0.08	0.09	0.17
Oil crops	0	0	0	0	0
Pulses	0	0	0	0	0
Roots & tubers	0	0	0	0	0
Sugar crops	0	0	0	0	0
Vegetables	0	0	0	0	0.01
**Croatia**					
Cereals	0.01	0.05	0.02	0.05	0.02
Fiber	0	0	0	0	0
Fruit	0	0	0	0	0
Livestock	0.03	0.1	0.04	0.15	0.06
Oil crops	0	0	0	0	0
Pulses	0	0	0	0	0
Roots & tubers	0	0	0	0	0
Sugar crops	0	0.01	0	0.01	0
Vegetables	0	0	0	0	0
**Cuba**					
Cereals	0	0.01	0.01	0	0.01
Fiber	0	0	0	0	0
Fruit	0.02	0.06	0.06	0.05	0.08
Livestock	0.01	0.04	0.07	0.06	0.1
Oil crops	0	0	0	0	0
Pulses	0	0	0	0	0
Roots & tubers	0.21	0.48	0.46	0.33	0.52
Sugar crops	0	0	0	0	0
Vegetables	0.03	0.06	0.06	0.04	0.07
**Cyprus**					
Cereals	0	0	0	0	0
Fiber	0	0	0	0	0
Fruit	0	0	0	0	0
Livestock	0.01	0.05	0.02	0.01	0
Oil crops	0	0	0	0	0
Pulses	0	0	0	0	0
Roots & tubers	0	0	0	0	0
Sugar crops	0	0	0	0	0
Vegetables	0	0	0	0	0
**Czech Republic**					
Cereals	0.01	0.03	0.01	0.03	0.01
Fiber	0	0	0	0	0
Fruit	0	0	0	0	0

Livestock	0.07	0.29	0.15	0.51	0.19
Oil crops	0	0	0	0	0
Pulses	0	0	0	0	0
Roots & tubers	0	0	0	0	0
Sugar crops	0	0.02	0.01	0.02	0.01
Vegetables	0	0	0	0	0
**Democratic Republic of the Congo**					
Cereals	0.03	0.05	0.01	0.01	0
Fiber	0	0	0	0	0
Fruit	0.15	0.23	0.02	0.07	0
Livestock	0	0.01	0	0	0
Oil crops	0	0	0	0	0
Pulses	0	0	0	0	0
Roots & tubers	0.39	0.64	0.06	0.23	0
Sugar crops	0	0	0	0	0
Vegetables	0	0.01	0	0	0
**Denmark**					
Cereals	0	0.01	0.01	0.02	0.01
Fiber	0	0	0	0	0
Fruit	0	0	0	0	0
Livestock	0.05	0.38	0.29	0.78	0.21
Oil crops	0	0	0	0	0
Pulses	0	0	0	0	0
Roots & tubers	0	0	0	0	0
Sugar crops	0	0.01	0.01	0.02	0.01
Vegetables	0	0	0	0	0
**Djibouti**					
Cereals	0	0	0	0	0
Fiber	0	0	0	0	0
Fruit	0	0	0	0	0
Livestock	0	0	0	0	0
Oil crops	0	0	0	0	0
Pulses	0	0	0	0	0
Roots & tubers	0	0	0	0	0
Sugar crops	0	0	0	0	0
Vegetables	0	0	0	0	0
**Dominica**					
Cereals	0	0	0	0	0
Fiber	0	0	0	0	0
Fruit	0	0	0	0	0
Livestock	0	0	0	0	0
Oil crops	0	0	0	0	0
Pulses	0	0	0	0	0
Roots & tubers	0	0	0	0	0
Sugar crops	0	0	0	0	0

Vegetables	0	0	0	0	0
**Ecuador**					
Cereals	0	0.01	0.01	0.02	0.03
Fiber	0	0	0	0	0
Fruit	0	0.05	0.04	0.08	0.18
Livestock	0.02	0.26	0.25	0.39	0.9
Oil crops	0	0	0	0	0
Pulses	0	0	0	0	0
Roots & tubers	0	0	0	0	0.01
Sugar crops	0	0	0	0	0
Vegetables	0	0.01	0.01	0.01	0.01
**Egypt**					
Cereals	0.07	0.31	0.12	0.06	0.01
Fiber	0	0	0	0	0
Fruit	0.04	0.18	0.07	0.03	0.01
Livestock	0.28	1.31	0.49	0.24	0.04
Oil crops	0.01	0.04	0.01	0.01	0
Pulses	0	0	0	0	0
Roots & tubers	0.22	1.01	0.37	0.18	0.03
Sugar crops	0.01	0.04	0.01	0.01	0
Vegetables	0.38	1.76	0.65	0.32	0.06
**El Salvador**					
Cereals	0.01	0.02	0.02	0.01	0
Fiber	0	0	0	0	0
Fruit	0.01	0.01	0.01	0	0
Livestock	0.04	0.09	0.1	0.04	0.02
Oil crops	0	0	0	0	0
Pulses	0	0	0	0	0
Roots & tubers	0	0	0	0	0
Sugar crops	0	0	0	0	0
Vegetables	0	0.01	0	0	0
**Equatorial Guinea**					
Cereals	0	0	0	0	0
Fiber	0	0	0	0	0
Fruit	0.01	0.01	0	0	0
Livestock	0	0	0	0	0
Oil crops	0	0	0	0	0
Pulses	0	0	0	0	0
Roots & tubers	0.16	0.2	0.01	0	0
Sugar crops	0	0	0	0	0
Vegetables	0	0	0	0	0
**Eritrea**					
Cereals	0	0	0	0	0
Fiber	0	0	0	0	0
Fruit	0	0	0	0	0
Livestock	0.01	0.03	0	0	0

Oil crops	0	0	0	0	0
Pulses	0	0	0	0	0
Roots & tubers	0	0	0	0	0
Sugar crops	0	0	0	0	0
Vegetables	0	0	0	0	0
**Estonia**					
Cereals	0	0	0	0	0
Fiber	0	0	0	0	0
Fruit	0	0	0	0	0
Livestock	0	0.03	0.03	0.13	0.05
Oil crops	0	0	0	0	0
Pulses	0	0	0	0	0
Roots & tubers	0	0	0	0	0
Sugar crops	0	0	0	0	0
Vegetables	0	0	0	0	0
**Ethiopia**					
Cereals	0.14	0.19	0.01	0	0
Fiber	0	0	0	0	0
Fruit	0	0	0	0	0
Livestock	0.32	0.52	0.05	0	0
Oil crops	0	0	0	0	0
Pulses	0.01	0.01	0	0	0
Roots & tubers	0.66	1.25	0.13	0.22	0
Sugar crops	0	0	0	0	0
Vegetables	0	0.01	0	0	0
**Finland**					
Cereals	0	0	0	0.01	0
Fiber	0	0	0	0	0
Fruit	0	0	0	0	0
Livestock	0.04	0.25	0.16	0.38	0.08
Oil crops	0	0	0	0	0
Pulses	0	0	0	0	0
Roots & tubers	0	0	0	0	0
Sugar crops	0	0	0	0.01	0
Vegetables	0	0	0	0.01	0
**France**					
Cereals	0.09	0.37	0.18	0.48	0.14
Fiber	0	0	0	0	0
Fruit	0.01	0.07	0.04	0.1	0.03
Livestock	0.87	3.46	1.58	3.59	0.82
Oil crops	0	0.01	0.01	0.03	0.01
Pulses	0	0.02	0.01	0.03	0.01
Roots & tubers	0	0	0	0	0
Sugar crops	0	0.04	0.04	0.27	0.13
Vegetables	0.01	0.06	0.03	0.11	0.04
**Gabon**					

Cereals	0	0	0	0	0
Fiber	0	0	0	0	0
Fruit	0.04	0.05	0	0	0
Livestock	0	0	0	0	0
Oil crops	0	0	0	0	0
Pulses	0	0	0	0	0
Roots & tubers	0.01	0.02	0	0	0
Sugar crops	0	0	0	0	0
Vegetables	0	0	0	0	0
**Gambia**					
Cereals	0	0	0	0	0
Fiber	0	0	0	0	0
Fruit	0	0	0	0	0
Livestock	0	0	0	0	0
Oil crops	0	0	0	0	0
Pulses	0	0	0	0	0
Roots & tubers	0	0	0	0	0
Sugar crops	0	0	0	0	0
Vegetables	0	0	0	0	0
**Georgia**					
Cereals	0	0.02	0.01	0	0
Fiber	0	0	0	0	0
Fruit	0	0	0	0	0
Livestock	0.03	0.15	0.05	0.03	0
Oil crops	0	0	0	0	0
Pulses	0	0	0	0	0
Roots & tubers	0	0	0	0	0
Sugar crops	0	0	0	0	0
Vegetables	0	0.02	0.01	0	0
**Germany**					
Cereals	0.05	0.16	0.05	0.15	0.05
Fiber	0	0	0	0	0
Fruit	0.01	0.03	0.01	0.02	0
Livestock	0.35	1.99	1.29	5.15	2.08
Oil crops	0	0.01	0.01	0.02	0.01
Pulses	0	0	0	0.01	0
Roots & tubers	0	0	0	0	0
Sugar crops	0.01	0.08	0.06	0.17	0.05
Vegetables	0	0.01	0	0.01	0
**Ghana**					
Cereals	0.04	0.06	0	0	0
Fiber	0	0	0	0	0
Fruit	0.45	0.62	0.05	0	0
Livestock	0.02	0.04	0	0	0
Oil crops	0	0	0	0	0
Pulses	0	0	0	0	0

Roots & tubers	0.31	0.48	0.04	0	0
Sugar crops	0	0	0	0	0
Vegetables	0.03	0.04	0	0	0
**Greece**					
Cereals	0.02	0.08	0.04	0.06	0.01
Fiber	0	0	0	0	0
Fruit	0.01	0.05	0.02	0.03	0
Livestock	0.13	0.49	0.18	0.26	0.02
Oil crops	0.04	0.17	0.07	0.1	0.01
Pulses	0	0	0	0	0
Roots & tubers	0	0.01	0	0.01	0
Sugar crops	0	0.01	0	0.01	0
Vegetables	0.07	0.26	0.1	0.16	0.02
**Grenada**					
Cereals	0	0	0	0	0
Fiber	0	0	0	0	0
Fruit	0	0	0	0	0
Livestock	0	0	0	0	0
Oil crops	0	0	0	0	0
Pulses	0	0	0	0	0
Roots & tubers	0	0	0	0	0
Sugar crops	0	0	0	0	0
Vegetables	0	0	0	0	0
**Guatemala**					
Cereals	0.01	0.02	0.03	0.02	0.03
Fiber	0	0	0	0	0
Fruit	0.01	0.02	0.04	0.03	0.03
Livestock	0.04	0.11	0.16	0.09	0.08
Oil crops	0	0	0	0	0
Pulses	0	0	0	0	0
Roots & tubers	0	0	0	0	0
Sugar crops	0	0	0	0	0
Vegetables	0.01	0.02	0.04	0.02	0.03
**Guinea**					
Cereals	0.01	0.03	0	0	0
Fiber	0	0	0	0	0
Fruit	0.05	0.11	0.01	0	0
Livestock	0.03	0.04	0	0	0
Oil crops	0	0	0	0	0
Pulses	0	0.01	0	0	0
Roots & tubers	0.3	0.63	0.07	0	0
Sugar crops	0	0	0	0	0
Vegetables	0	0	0	0	0
**Guinea-Bissau**					
Cereals	0	0	0	0	0
Fiber	0	0	0	0	0

Fruit	0.01	0.01	0	0	0
Livestock	0	0.01	0	0	0
Oil crops	0	0	0	0	0
Pulses	0	0	0	0	0
Roots & tubers	0	0	0	0	0
Sugar crops	0	0	0	0	0
Vegetables	0	0	0	0	0
**Guyana**					
Cereals	0	0	0	0	0
Fiber	0	0	0	0	0
Fruit	0	0	0	0	0
Livestock	0	0	0	0	0.01
Oil crops	0	0	0	0	0
Pulses	0	0	0	0	0
Roots & tubers	0	0	0	0	0.01
Sugar crops	0	0	0	0	0
Vegetables	0	0	0	0	0
**Haiti**					
Cereals	0	0.01	0.01	0	0
Fiber	0	0	0	0	0
Fruit	0.02	0.04	0.03	0.01	0.01
Livestock	0.01	0.01	0.01	0.01	0
Oil crops	0	0	0	0	0
Pulses	0	0	0	0	0
Roots & tubers	0.17	0.37	0.33	0.14	0.06
Sugar crops	0	0	0	0	0
Vegetables	0	0	0	0	0
**Honduras**					
Cereals	0	0.01	0.01	0.01	0.02
Fiber	0	0	0	0	0
Fruit	0.01	0.01	0.01	0.01	0.03
Livestock	0.04	0.08	0.05	0.05	0.13
Oil crops	0	0	0	0	0
Pulses	0	0	0	0	0
Roots & tubers	0	0.01	0	0	0.01
Sugar crops	0	0	0	0	0
Vegetables	0.01	0.01	0.01	0.01	0.02
**Hungary**					
Cereals	0.05	0.19	0.08	0.22	0.07
Fiber	0	0	0	0	0
Fruit	0	0.01	0	0.01	0
Livestock	0.06	0.29	0.15	0.39	0.11
Oil crops	0	0.01	0	0.01	0
Pulses	0	0	0	0	0
Roots & tubers	0	0	0	0	0
Sugar crops	0	0.01	0.01	0.01	0

Vegetables	0.01	0.03	0.01	0.02	0.01
**Iceland**					
Cereals	0	0	0	0	0
Fiber	0	0	0	0	0
Fruit	0	0	0	0	0
Livestock	0	0	0	0	0
Oil crops	0	0	0	0	0
Pulses	0	0	0	0	0
Roots & tubers	0	0	0	0	0
Sugar crops	0	0	0	0	0
Vegetables	0	0	0	0	0
**India**					
Cereals	0.43	0.84	0.1	0.02	0
Fiber	0	0	0	0	0
Fruit	0.24	0.54	0.07	0.01	0
Livestock	11.02	24.01	2.95	0.57	0
Oil crops	0.05	0.12	0.02	0	0
Pulses	0.05	0.12	0.02	0	0
Roots & tubers	1.78	3.44	0.4	0.08	0
Sugar crops	0	0	0	0	0
Vegetables	1.43	2.01	0.18	0.04	0
**Indonesia**					
Cereals	0.42	0.58	0.02	0.04	0
Fiber	0	0	0	0	0
Fruit	0.08	0.15	0.01	0.08	0
Livestock	1.03	1.36	0.04	0.04	0
Oil crops	0	0	0	0	0
Pulses	0	0	0	0	0
Roots & tubers	3.17	4.42	0.15	1.95	0
Sugar crops	0	0	0	0	0
Vegetables	0.06	0.11	0.01	0.06	0
**Iran (Islamic Republic of)**					
Cereals	0.05	0.11	0.01	0.04	0
Fiber	0	0	0	0	0
Fruit	0.1	0.22	0.03	0.08	0
Livestock	0.66	2.16	0.3	0.9	0
Oil crops	0	0.01	0	0	0
Pulses	0	0	0	0	0
Roots & tubers	0	0	0	0	0
Sugar crops	0.01	0.05	0.01	0.02	0
Vegetables	0.45	0.99	0.12	0.4	0
**Iraq**					
Cereals	0.01	0.02	0.01	0	0
Fiber	0	0	0	0	0
Fruit	0	0.02	0.01	0	0
Livestock	0.02	0.1	0.03	0.02	0.02

Oil crops	0	0	0	0	0
Pulses	0	0	0	0	0
Roots & tubers	0	0	0	0	0
Sugar crops	0	0	0	0	0
Vegetables	0.04	0.21	0.08	0.04	0.01
**Ireland**					
Cereals	0	0	0	0.01	0
Fiber	0	0	0	0	0
Fruit	0	0	0	0	0
Livestock	0.16	0.68	0.33	0.63	0.09
Oil crops	0	0	0	0	0
Pulses	0	0	0	0	0
Roots & tubers	0	0	0	0	0
Sugar crops	0	0	0	0.01	0
Vegetables	0	0	0	0	0
**Israel**					
Cereals	0	0.01	0	0	0
Fiber	0	0	0	0	0
Fruit	0.02	0.1	0.03	0.02	0
Livestock	0.19	0.79	0.27	0.13	0.02
Oil crops	0	0.01	0	0	0
Pulses	0	0	0	0	0
Roots & tubers	0.04	0.16	0.05	0.03	0
Sugar crops	0	0	0	0	0
Vegetables	0.04	0.19	0.07	0.03	0.01
**Italy**					
Cereals	0.03	0.23	0.18	0.37	0.05
Fiber	0	0	0	0	0
Fruit	0.02	0.11	0.07	0.22	0.07
Livestock	0.25	1.55	1.04	2.16	0.31
Oil crops	0.04	0.14	0.04	0.22	0.12
Pulses	0	0	0	0	0
Roots & tubers	0	0.02	0.01	0.03	0.01
Sugar crops	0	0.03	0.02	0.05	0.01
Vegetables	0.12	0.67	0.41	1.07	0.28
**Ivory Coast**					
Cereals	0	0	0	0	0
Fiber	0	0	0	0	0
Fruit	0	0	0	0	0
Livestock	0	0.04	0.01	0	0
Oil crops	0	0	0	0	0
Pulses	0	0	0	0	0
Roots & tubers	0	0	0	0	0
Sugar crops	0	0	0	0	0
Vegetables	0	0	0	0	0
**Jamaica**					

Cereals	0	0	0	0	0
Fiber	0	0	0	0	0
Fruit	0.01	0.01	0	0	0
Livestock	0.04	0.07	0.01	0.01	0
Oil crops	0	0	0	0	0
Pulses	0	0	0	0	0
Roots & tubers	0.05	0.08	0.01	0	0
Sugar crops	0	0	0	0	0
Vegetables	0	0	0	0	0
**Japan**					
Cereals	0	0	0	0	0
Fiber	0	0	0	0	0
Fruit	0.01	0.01	0.01	0	0
Livestock	2.82	2.29	0.7	0.86	0
Oil crops	0	0	0	0	0
Pulses	0	0	0	0	0
Roots & tubers	3.19	1.6	0.27	0.08	0
Sugar crops	0.02	0.03	0.01	0.01	0
Vegetables	0.08	0.12	0.05	0.04	0
**Jordan**					
Cereals	0	0	0	0	0
Fiber	0	0	0	0	0
Fruit	0	0	0	0	0
Livestock	0.03	0.12	0.04	0.02	0
Oil crops	0	0.01	0	0	0
Pulses	0	0	0	0	0
Roots & tubers	0	0	0	0	0
Sugar crops	0	0	0	0	0
Vegetables	0.02	0.11	0.04	0.02	0
**Kazakhstan**					
Cereals	0	0.03	0.01	0.01	0.01
Fiber	0	0	0	0	0
Fruit	0	0	0	0	0
Livestock	0.11	0.75	0.32	0.31	0.37
Oil crops	0	0	0	0	0
Pulses	0	0	0	0	0
Roots & tubers	0	0	0	0	0
Sugar crops	0	0	0	0	0
Vegetables	0.01	0.09	0.04	0.03	0.02
**Kenya**					
Cereals	0.06	0.13	0.01	0.05	0
Fiber	0	0	0	0	0
Fruit	0.01	0.02	0	0.01	0
Livestock	0.32	0.69	0.08	0.26	0
Oil crops	0	0	0	0	0
Pulses	0	0	0	0	0

Roots & tubers	0.87	1.88	0.22	0.8	0
Sugar crops	0	0	0	0	0
Vegetables	0.05	0.1	0.01	0.05	0
**Kuwait**					
Cereals	0	0	0	0	0
Fiber	0	0	0	0	0
Fruit	0	0	0	0	0
Livestock	0	0.03	0.02	0.01	0
Oil crops	0	0	0	0	0
Pulses	0	0	0	0	0
Roots & tubers	0	0	0	0	0
Sugar crops	0	0	0	0	0
Vegetables	0	0.01	0.01	0	0
**Kyrgyzstan**					
Cereals	0	0.01	0.01	0.01	0.01
Fiber	0	0	0	0	0
Fruit	0	0	0	0	0
Livestock	0.06	0.22	0.06	0.05	0.04
Oil crops	0	0	0	0	0
Pulses	0	0	0	0	0
Roots & tubers	0	0	0	0	0
Sugar crops	0	0	0	0	0
Vegetables	0	0.03	0.01	0.01	0.02
**Lao People's Democratic Republic**					
Cereals	0.01	0.03	0	0	0
Fiber	0	0	0	0	0
Fruit	0	0.01	0	0	0
Livestock	0.01	0.02	0	0	0
Oil crops	0	0	0	0	0
Pulses	0	0	0	0	0
Roots & tubers	0.19	0.4	0.02	0.01	0
Sugar crops	0	0	0	0	0
Vegetables	0	0	0	0	0
**Latvia**					
Cereals	0	0	0	0	0
Fiber	0	0	0	0	0
Fruit	0	0	0	0	0
Livestock	0.01	0.06	0.04	0.16	0.06
Oil crops	0	0	0	0	0
Pulses	0	0	0	0	0
Roots & tubers	0	0	0	0	0
Sugar crops	0	0	0	0	0
Vegetables	0	0	0	0	0
**Lebanon**					
Cereals	0	0	0	0	0

Fiber	0	0	0	0	0
Fruit	0	0.01	0	0	0.01
Livestock	0.01	0.05	0.02	0.04	0.07
Oil crops	0	0.01	0	0	0
Pulses	0	0	0	0	0
Roots & tubers	0	0	0	0	0
Sugar crops	0	0	0	0	0
Vegetables	0.01	0.05	0.02	0.02	0.02
**Lesotho**					
Cereals	0	0	0	0	0
Fiber	0	0	0	0	0
Fruit	0	0	0	0	0
Livestock	0	0.01	0	0	0
Oil crops	0	0	0	0	0
Pulses	0	0	0	0	0
Roots & tubers	0	0	0	0	0
Sugar crops	0	0	0	0	0
Vegetables	0	0	0	0	0
**Liberia**					
Cereals	0	0	0	0	0
Fiber	0	0	0	0	0
Fruit	0	0.01	0	0	0
Livestock	0	0.01	0	0	0
Oil crops	0	0	0	0	0
Pulses	0	0	0	0	0
Roots & tubers	0.02	0.07	0.01	0	0
Sugar crops	0	0	0	0	0
Vegetables	0	0	0	0	0
**Libya**					
Cereals	0	0	0	0	0
Fiber	0	0	0	0	0
Fruit	0	0.01	0	0	0
Livestock	0.01	0.1	0.04	0.03	0.03
Oil crops	0	0.01	0	0	0.01
Pulses	0	0	0	0	0
Roots & tubers	0	0	0	0	0
Sugar crops	0	0	0	0	0
Vegetables	0.01	0.03	0.01	0.01	0.02
**Lithuania**					
Cereals	0	0	0	0	0
Fiber	0	0	0	0	0
Fruit	0	0	0	0	0
Livestock	0.03	0.16	0.09	0.33	0.12
Oil crops	0	0	0	0	0
Pulses	0	0	0	0	0
Roots & tubers	0	0	0	0	0

Sugar crops	0	0	0	0.01	0
Vegetables	0	0	0	0	0
**Luxembourg**					
Cereals	0	0	0	0	0
Fiber	0	0	0	0	0
Fruit	0	0	0	0	0
Livestock	0	0.01	0.02	0.05	0.01
Oil crops	0	0	0	0	0
Pulses	0	0	0	0	0
Roots & tubers	0	0	0	0	0
Sugar crops	0	0	0	0	0
Vegetables	0	0	0	0	0
**Madagascar**					
Cereals	0.02	0.02	0	0	0
Fiber	0	0	0	0	0
Fruit	0.01	0.01	0	0	0
Livestock	0.1	0.12	0.01	0	0
Oil crops	0	0	0	0	0
Pulses	0	0	0	0	0
Roots & tubers	1.99	2.41	0.14	0	0
Sugar crops	0	0	0	0	0
Vegetables	0.01	0.01	0	0	0
**Malawi**					
Cereals	0.07	0.11	0.01	0.01	0
Fiber	0	0	0	0	0
Fruit	0.04	0.07	0.01	0	0
Livestock	0.02	0.02	0	0	0
Oil crops	0	0	0	0	0
Pulses	0	0	0	0	0
Roots & tubers	0.01	0.01	0	0	0
Sugar crops	0	0	0	0	0
Vegetables	0.01	0.01	0	0	0
**Malaysia**					
Cereals	0	0	0	0	0
Fiber	0	0	0	0	0
Fruit	0	0.01	0	0	0
Livestock	0.19	0.64	0.06	0.11	0
Oil crops	0	0	0	0	0
Pulses	0	0	0	0	0
Roots & tubers	0.02	0.06	0	0.07	0
Sugar crops	0	0	0	0	0
Vegetables	0	0.01	0	0	0
**Mali**					
Cereals	0.02	0.04	0	0	0
Fiber	0	0	0	0	0
Fruit	0	0	0	0	0

Livestock	0.15	0.3	0.03	0	0
Oil crops	0	0	0	0	0
Pulses	0	0	0	0	0
Roots & tubers	0.38	0.6	0.05	0	0
Sugar crops	0	0	0	0	0
Vegetables	0.01	0.03	0	0	0
**Mauritania**					
Cereals	0	0	0	0	0
Fiber	0	0	0	0	0
Fruit	0	0	0	0	0
Livestock	0.06	0.09	0.01	0	0
Oil crops	0	0	0	0	0
Pulses	0	0	0	0	0
Roots & tubers	0	0.01	0	0	0
Sugar crops	0	0	0	0	0
Vegetables	0	0	0	0	0
**Mauritius**					
Cereals	0	0	0	0	0
Fiber	0	0	0	0	0
Fruit	0	0	0	0	0
Livestock	0	0	0	0	0
Oil crops	0	0	0	0	0
Pulses	0	0	0	0	0
Roots & tubers	0	0	0	0	0
Sugar crops	0	0	0	0	0
Vegetables	0	0	0	0	0
**Mexico**					
Cereals	0.34	0.67	0.37	0.21	0.24
Fiber	0	0	0	0	0
Fruit	0.02	0.08	0.15	0.1	0.14
Livestock	0.68	1.76	2.16	1.27	1.46
Oil crops	0	0	0	0	0
Pulses	0	0.01	0	0	0
Roots & tubers	0.04	0.08	0.07	0.05	0.09
Sugar crops	0	0	0	0	0
Vegetables	0.07	0.18	0.26	0.22	0.4
**Mongolia**					
Cereals	0	0	0	0	0
Fiber	0	0	0	0	0
Fruit	0	0	0	0	0
Livestock	0.04	0.04	0.01	0.04	0
Oil crops	0	0	0	0	0
Pulses	0	0	0	0	0
Roots & tubers	0	0	0	0	0
Sugar crops	0	0	0	0	0
Vegetables	0	0	0	0	0

**Montenegro**					
Cereals	0	0	0	0	0
Fiber	0	0	0	0	0
Fruit	0	0	0	0	0
Livestock	0.1	0.37	0.12	0.14	0.01
Oil crops	0	0	0	0	0
Pulses	0	0	0	0	0
Roots & tubers	0	0	0	0	0
Sugar crops	0	0	0	0	0
Vegetables	0	0	0	0	0
**Morocco**					
Cereals	0	0.01	0	0	0
Fiber	0	0	0	0	0
Fruit	0.01	0.04	0.01	0.01	0.01
Livestock	0.18	0.55	0.14	0.08	0.05
Oil crops	0.01	0.05	0.01	0.01	0.02
Pulses	0	0	0	0	0
Roots & tubers	0.01	0.02	0.01	0.01	0.01
Sugar crops	0.01	0.02	0.01	0.01	0.01
Vegetables	0.07	0.23	0.06	0.05	0.05
**Mozambique**					
Cereals	0.03	0.07	0.01	0	0
Fiber	0	0	0	0	0
Fruit	0	0	0	0	0
Livestock	0.01	0.03	0	0	0
Oil crops	0	0	0	0	0
Pulses	0.01	0.01	0	0	0
Roots & tubers	0.87	1.85	0.22	0.59	0
Sugar crops	0	0	0	0	0
Vegetables	0.01	0.02	0	0.01	0
**Myanmar**					
Cereals	0.03	0.05	0	0	0
Fiber	0	0	0	0	0
Fruit	0.09	0.15	0.01	0	0
Livestock	0.35	0.53	0.02	0.02	0
Oil crops	0	0.01	0	0	0
Pulses	0.01	0.02	0	0	0
Roots & tubers	0.1	0.17	0.01	0	0
Sugar crops	0	0	0	0	0
Vegetables	0	0	0	0	0
**Namibia**					
Cereals	0	0	0	0	0
Fiber	0	0	0	0	0
Fruit	0	0	0	0	0
Livestock	0.01	0.02	0	0.01	0
Oil crops	0	0	0	0	0

Pulses	0	0	0	0	0
Roots & tubers	0	0	0	0	0
Sugar crops	0	0	0	0	0
Vegetables	0	0	0	0	0
**Nepal**					
Cereals	0.05	0.09	0.01	0	0
Fiber	0	0	0	0	0
Fruit	0	0	0	0	0
Livestock	0.17	0.31	0.03	0.01	0
Oil crops	0	0	0	0	0
Pulses	0	0	0	0	0
Roots & tubers	0	0	0	0	0
Sugar crops	0	0	0	0	0
Vegetables	0	0	0	0	0
**Netherlands**					
Cereals	0	0.01	0	0.01	0
Fiber	0	0	0	0	0
Fruit	0	0	0	0	0
Livestock	0.14	1.07	0.81	2.2	0.58
Oil crops	0	0	0	0	0
Pulses	0	0	0	0	0
Roots & tubers	0	0	0	0	0
Sugar crops	0	0.01	0.01	0.04	0.02
Vegetables	0.01	0.04	0.03	0.12	0.05
**New Zealand**					
Cereals	0	0	0	0	0.02
Fiber	0	0	0	0	0
Fruit	0	0	0	0	0.01
Livestock	0.01	0.18	0.26	1.08	3.59
Oil crops	0	0	0	0	0
Pulses	0	0	0	0	0
Roots & tubers	0	0	0	0.01	0.07
Sugar crops	0	0	0	0	0
Vegetables	0	0	0	0.01	0.03
**Nicaragua**					
Cereals	0.01	0.01	0.01	0.01	0.01
Fiber	0	0	0	0	0
Fruit	0	0.01	0.01	0	0
Livestock	0.03	0.07	0.07	0.05	0.06
Oil crops	0	0	0	0	0
Pulses	0	0	0	0	0
Roots & tubers	0	0	0	0	0
Sugar crops	0	0	0	0	0
Vegetables	0	0	0	0	0
**Niger**					
Cereals	0	0	0	0	0

Fiber	0	0	0	0	0
Fruit	0	0	0	0	0
Livestock	0.12	0.17	0.01	0.02	0
Oil crops	0	0	0	0	0
Pulses	0	0	0	0	0
Roots & tubers	0.17	0.23	0.02	0.04	0
Sugar crops	0	0	0	0	0
Vegetables	0.02	0.02	0	0	0
**Nigeria**					
Cereals	0.17	0.32	0.04	0.03	0
Fiber	0	0	0	0	0
Fruit	0.29	0.58	0.07	0.09	0
Livestock	0.15	0.34	0.04	0.44	0
Oil crops	0	0	0	0	0
Pulses	0	0.01	0	0	0
Roots & tubers	4.97	10.1	1.14	1.68	0
Sugar crops	0	0	0	0	0
Vegetables	0.33	0.39	0.02	0	0
**Norway**					
Cereals	0	0	0	0	0
Fiber	0	0	0	0	0
Fruit	0	0	0	0	0
Livestock	0.01	0.14	0.13	0.3	0.04
Oil crops	0	0	0	0	0
Pulses	0	0	0	0	0
Roots & tubers	0	0	0	0	0
Sugar crops	0	0	0	0	0
Vegetables	0	0	0	0	0
**Oman**					
Cereals	0	0	0	0	0
Fiber	0	0	0	0	0
Fruit	0	0.01	0	0	0
Livestock	0.01	0.04	0.02	0.01	0
Oil crops	0	0	0	0	0
Pulses	0	0	0	0	0
Roots & tubers	0	0	0	0	0
Sugar crops	0	0	0	0	0
Vegetables	0	0.01	0	0	0
**Pakistan**					
Cereals	0.08	0.17	0.02	0	0
Fiber	0	0	0	0	0
Fruit	0.04	0.11	0.01	0	0
Livestock	3.78	6.51	0.71	0.14	0
Oil crops	0	0.01	0	0	0
Pulses	0.01	0.01	0	0	0
Roots & tubers	0.01	0.03	0	0	0

Sugar crops	0	0	0	0	0
Vegetables	0.05	0.11	0.01	0	0
**Panama**					
Cereals	0	0	0	0	0
Fiber	0	0	0	0	0
Fruit	0.01	0.02	0.02	0.01	0.01
Livestock	0.03	0.06	0.03	0.01	0.01
Oil crops	0	0	0	0	0
Pulses	0	0	0	0	0
Roots & tubers	0	0	0	0	0
Sugar crops	0	0	0	0	0
Vegetables	0	0	0	0	0
**Papua New Guinea**					
Cereals	0	0	0	0	0
Fiber	0	0	0	0	0
Fruit	0.01	0.01	0	0	0
Livestock	0.01	0	0	0	0
Oil crops	0	0	0	0	0
Pulses	0	0	0	0	0
Roots & tubers	0.87	1.22	0.46	0.25	0
Sugar crops	0	0	0	0	0
Vegetables	0	0	0	0	0
**Paraguay**					
Cereals	0	0.01	0.01	0.02	0.09
Fiber	0	0	0	0	0
Fruit	0	0	0	0.01	0.03
Livestock	0	0.04	0.04	0.07	0.15
Oil crops	0	0	0	0.01	0.04
Pulses	0	0	0	0	0
Roots & tubers	0	0.03	0.03	0.11	0.56
Sugar crops	0	0	0	0	0
Vegetables	0	0	0	0.01	0.02
**Peru**					
Cereals	0	0.03	0.02	0.03	0.03
Fiber	0	0	0	0	0
Fruit	0.01	0.14	0.13	0.17	0.23
Livestock	0.02	0.27	0.24	0.27	0.23
Oil crops	0	0	0	0	0
Pulses	0	0	0	0	0
Roots & tubers	0.01	0.21	0.2	0.25	0.31
Sugar crops	0	0	0	0	0
Vegetables	0	0.02	0.02	0.02	0
**Philippines**					
Cereals	0.05	0.22	0.02	0.22	0
Fiber	0	0	0	0	0
Fruit	0.03	0.09	0.01	0.04	0

Livestock	0.27	0.51	0.03	0.01	0
Oil crops	0	0	0	0	0
Pulses	0	0	0	0	0
Roots & tubers	0.49	1.31	0.11	1.03	0
Sugar crops	0	0	0	0	0
Vegetables	0.01	0.03	0	0.02	0
**Poland**					
Cereals	0.01	0.04	0.02	0.09	0.04
Fiber	0	0	0	0	0
Fruit	0	0.02	0.01	0.02	0.01
Livestock	0.47	1.71	0.67	1.69	0.53
Oil crops	0	0	0	0.01	0
Pulses	0	0.01	0	0.01	0
Roots & tubers	0	0	0	0	0
Sugar crops	0.01	0.04	0.02	0.08	0.03
Vegetables	0.02	0.08	0.03	0.09	0.03
**Portugal**					
Cereals	0	0.02	0.01	0.02	0
Fiber	0	0	0	0	0
Fruit	0.01	0.02	0.01	0.02	0.01
Livestock	0.12	0.4	0.11	0.24	0.08
Oil crops	0	0.01	0	0.02	0.01
Pulses	0	0	0	0	0
Roots & tubers	0.01	0.02	0.01	0.04	0.02
Sugar crops	0	0	0	0	0
Vegetables	0.03	0.1	0.04	0.17	0.08
**Qatar**					
Cereals	0	0	0	0	0
Fiber	0	0	0	0	0
Fruit	0	0	0	0	0
Livestock	0	0	0	0	0
Oil crops	0	0	0	0	0
Pulses	0	0	0	0	0
Roots & tubers	0	0	0	0	0
Sugar crops	0	0	0	0	0
Vegetables	0	0	0	0	0
**Republic of Korea**					
Cereals	0	0	0	0	0
Fiber	0	0	0	0	0
Fruit	0.01	0.01	0	0	0
Livestock	0.71	0.96	0.03	0.01	0
Oil crops	0	0	0	0	0
Pulses	0	0	0	0	0
Roots & tubers	0.66	0.88	0.03	0.01	0
Sugar crops	0	0	0	0	0
Vegetables	0.07	0.1	0	0	0

**Romania**					
Cereals	0.03	0.14	0.08	0.3	0.12
Fiber	0	0	0	0	0
Fruit	0	0.01	0	0.02	0.01
Livestock	0.16	0.69	0.34	1.13	0.42
Oil crops	0	0	0	0.01	0
Pulses	0	0	0	0	0
Roots & tubers	0	0	0	0	0
Sugar crops	0	0	0	0	0
Vegetables	0.01	0.06	0.03	0.11	0.04
**Russian Federation**					
Cereals	0.02	0.11	0.06	0.22	0.09
Fiber	0	0	0	0	0
Fruit	0	0.01	0	0.03	0.02
Livestock	0.68	3.03	1.6	6.16	2.51
Oil crops	0	0.02	0.01	0.06	0.03
Pulses	0	0.02	0.01	0.03	0.01
Roots & tubers	0	0	0	0	0
Sugar crops	0.02	0.07	0.03	0.19	0.1
Vegetables	0.04	0.18	0.09	0.36	0.16
**Rwanda**					
Cereals	0	0.01	0	0	0
Fiber	0	0	0	0	0
Fruit	0.34	0.56	0.05	0	0
Livestock	0.03	0.04	0	0	0
Oil crops	0	0	0	0	0
Pulses	0	0	0	0	0
Roots & tubers	1.64	2.43	0.2	0	0
Sugar crops	0	0	0	0	0
Vegetables	0.01	0.01	0	0	0
**Saint Lucia**					
Cereals	0	0	0	0	0
Fiber	0	0	0	0	0
Fruit	0	0	0	0	0
Livestock	0	0	0	0	0
Oil crops	0	0	0	0	0
Pulses	0	0	0	0	0
Roots & tubers	0	0	0	0	0
Sugar crops	0	0	0	0	0
Vegetables	0	0	0	0	0
**Saint Vincent and the Grenadines**					
Cereals	0	0	0	0	0
Fiber	0	0	0	0	0
Fruit	0	0	0	0	0
Livestock	0	0	0	0	0

Oil crops	0	0	0	0	0
Pulses	0	0	0	0	0
Roots & tubers	0	0	0	0	0
Sugar crops	0	0	0	0	0
Vegetables	0	0	0	0	0
**Saudi Arabia**					
Cereals	0	0.01	0	0	0
Fiber	0	0	0	0	0
Fruit	0.01	0.04	0.01	0.01	0
Livestock	0.12	0.48	0.16	0.08	0.01
Oil crops	0	0	0	0	0
Pulses	0	0	0	0	0
Roots & tubers	0	0	0	0	0
Sugar crops	0	0	0	0	0
Vegetables	0.03	0.11	0.03	0.01	0
**Senegal**					
Cereals	0.01	0.01	0	0	0
Fiber	0	0	0	0	0
Fruit	0	0	0	0	0
Livestock	0.02	0.06	0.01	0	0
Oil crops	0	0	0	0	0
Pulses	0	0	0	0	0
Roots & tubers	0.07	0.09	0.01	0	0
Sugar crops	0	0	0	0	0
Vegetables	0.02	0.03	0	0	0
**Serbia**					
Cereals	0.04	0.14	0.05	0.15	0.05
Fiber	0	0	0	0	0
Fruit	0	0.01	0	0	0
Oil crops	0	0	0	0	0
Pulses	0	0	0	0	0
Roots & tubers	0	0	0	0	0
Sugar crops	0.01	0.02	0.01	0.01	0
Vegetables	0.01	0.02	0.01	0.02	0.01
**Sierra Leone**					
Cereals	0	0	0	0	0
Fiber	0	0	0	0	0
Fruit	0	0.01	0	0	0
Livestock	0.01	0.01	0	0	0
Oil crops	0	0	0	0	0
Pulses	0	0.01	0	0	0
Roots & tubers	0.16	0.35	0.04	0	0
Sugar crops	0	0	0	0	0
Vegetables	0	0	0	0	0
**Slovakia**					
Cereals	0.01	0.02	0.01	0.03	0.01

Fiber	0	0	0	0	0
Fruit	0	0	0	0	0
Livestock	0.02	0.11	0.07	0.23	0.08
Oil crops	0	0	0	0	0
Pulses	0	0	0	0	0
Roots & tubers	0	0	0	0	0
Sugar crops	0	0.01	0	0.01	0
Vegetables	0	0.01	0	0.01	0
**Slovenia**					
Cereals	0	0.01	0	0	0
Fiber	0	0	0	0	0
Fruit	0	0	0	0	0
Livestock	0.04	0.14	0.04	0.03	0
Oil crops	0	0	0	0	0
Pulses	0	0	0	0	0
Roots & tubers	0	0	0	0	0
Sugar crops	0	0	0	0	0
Vegetables	0	0	0	0	0
**Somalia**					
Cereals	0	0.01	0	0	0
Fiber	0	0	0	0	0
Fruit	0	0	0	0	0
Livestock	0.19	0.39	0.04	0	0
Oil crops	0	0	0	0	0
Pulses	0	0	0	0	0
Roots & tubers	0	0	0	0	0
Sugar crops	0	0	0	0	0
Vegetables	0	0	0	0	0
**South Africa**					
Cereals	0.02	0.19	0.03	0.48	0
Fiber	0	0	0	0	0
Fruit	0.05	0.14	0.02	0.06	0
Livestock	0.22	0.7	0.1	0.86	0
Oil crops	0	0	0	0	0
Pulses	0	0	0	0	0
Roots & tubers	0.03	0.12	0.02	0.09	0
Sugar crops	0	0	0	0	0
Vegetables	0.03	0.1	0.01	0.04	0
**Spain**					
Cereals	0.02	0.1	0.05	0.16	0.05
Fiber	0	0	0	0	0
Fruit	0.03	0.13	0.05	0.17	0.06
Livestock	0.36	1.39	0.59	1.33	0.35
Oil crops	0.02	0.12	0.07	0.43	0.21
Pulses	0	0	0	0.01	0
Roots & tubers	0.01	0.03	0.01	0.05	0.02

Sugar crops	0	0.01	0.01	0.05	0.02
Vegetables	0.1	0.4	0.18	0.67	0.27
**Sri Lanka**					
Cereals	0	0	0	0	0
Fiber	0	0	0	0	0
Fruit	0.04	0.09	0.01	0.04	0
Livestock	0.06	0.08	0.01	0.01	0
Oil crops	0	0	0	0	0
Pulses	0	0	0	0	0
Roots & tubers	0.05	0.12	0.01	0.04	0
Sugar crops	0	0	0	0	0
Vegetables	0.01	0.01	0	0.01	0
**Sudan (former)**					
Cereals	0	0	0	0	0
Fiber	0	0	0	0	0
Fruit	0.01	0.03	0.01	0.01	0.02
Livestock	0.37	1.34	0.39	0.34	0.37
Oil crops	0	0	0	0	0
Pulses	0	0	0	0	0
Roots & tubers	0.11	0.39	0.11	0.13	0.18
Sugar crops	0	0	0	0	0
Vegetables	0.03	0.09	0.03	0.03	0.04
**Suriname**					
Cereals	0	0	0	0	0
Fiber	0	0	0	0	0
Fruit	0	0	0	0	0
Livestock	0	0	0	0	0.01
Oil crops	0	0	0	0	0
Pulses	0	0	0	0	0
Roots & tubers	0	0	0	0	0
Sugar crops	0	0	0	0	0
Vegetables	0	0	0	0	0
**Swaziland**					
Cereals	0	0	0	0	0
Fiber	0	0	0	0	0
Fruit	0	0.01	0	0	0
Livestock	0	0.01	0	0	0
Oil crops	0	0	0	0	0
Pulses	0	0	0	0	0
Roots & tubers	0	0.01	0	0	0
Sugar crops	0	0	0	0	0
Vegetables	0	0	0	0	0
**Sweden**					
Cereals	0	0	0	0.01	0
Fiber	0	0	0	0	0
Fruit	0	0	0	0	0

Livestock	0.03	0.26	0.19	0.56	0.17
Oil crops	0	0	0	0	0
Pulses	0	0	0	0	0
Roots & tubers	0	0	0	0	0
Sugar crops	0	0.01	0.01	0.01	0
Vegetables	0	0	0	0	0
**Switzerland**					
Cereals	0	0	0	0.01	0
Fiber	0	0	0	0	0
Fruit	0	0	0	0	0
Livestock	0.05	0.37	0.29	0.6	0.07
Oil crops	0	0	0	0	0
Pulses	0	0	0	0	0
Roots & tubers	0	0	0	0	0
Sugar crops	0	0.01	0	0.01	0
Vegetables	0	0	0	0	0
**Syrian Arab Republic**					
Cereals	0.01	0.01	0	0	0
Fiber	0	0	0	0	0
Fruit	0.01	0.03	0.01	0.01	0.01
Livestock	0.22	0.67	0.17	0.12	0.11
Oil crops	0.02	0.06	0.02	0.01	0.01
Pulses	0	0	0	0	0
Roots & tubers	0	0	0	0	0
Sugar crops	0	0.01	0	0	0
Vegetables	0.06	0.2	0.06	0.05	0.04
**Tajikistan**					
Cereals	0	0.01	0	0	0
Fiber	0	0	0	0	0
Fruit	0	0	0	0	0
Livestock	0.02	0.08	0.03	0.03	0.04
Oil crops	0	0	0	0	0
Pulses	0	0	0	0	0
Roots & tubers	0	0	0	0	0
Sugar crops	0	0	0	0	0
Vegetables	0.01	0.04	0.01	0.01	0.01
**Thailand**					
Cereals	0.06	0.24	0.02	0.01	0
Fiber	0	0	0	0	0
Fruit	0.05	0.12	0.01	0	0
Livestock	0.26	1.33	0.14	0.06	0
Oil crops	0	0	0	0	0
Pulses	0	0.01	0	0	0
Roots & tubers	0.04	0.13	0.01	0.01	0
Sugar crops	0	0	0	0	0
Vegetables	0.01	0.04	0	0	0

**The former Yugoslav Republic of Macedonia**					
Cereals	0	0	0	0	0
Fiber	0	0	0	0	0
Fruit	0	0	0	0	0
Livestock	0.01	0.06	0.03	0.04	0
Oil crops	0	0	0	0	0
Pulses	0	0	0	0	0
Roots & tubers	0	0	0	0	0
Sugar crops	0	0	0	0	0
Vegetables	0	0.02	0.01	0.02	0
**Togo**					
Cereals	0.01	0.03	0	0	0
Fiber	0	0	0	0	0
Fruit	0	0	0	0	0
Livestock	0.01	0.01	0	0	0
Oil crops	0	0	0	0	0
Pulses	0	0	0	0	0
Roots & tubers	0.02	0.03	0	0	0
Sugar crops	0	0	0	0	0
Vegetables	0	0	0	0	0
**Trinidad and Tobago**					
Cereals	0	0	0	0	0
Fiber	0	0	0	0	0
Fruit	0	0	0	0	0
Livestock	0	0	0	0	0
Oil crops	0	0	0	0	0
Pulses	0	0	0	0	0
Roots & tubers	0	0	0	0	0
Sugar crops	0	0	0	0	0
Vegetables	0	0	0	0	0
**Tunisia**					
Cereals	0	0	0	0	0
Fiber	0	0	0	0	0
Fruit	0	0.02	0.01	0.01	0.01
Livestock	0.05	0.25	0.09	0.06	0.04
Oil crops	0.02	0.07	0.03	0.03	0.03
Pulses	0	0	0	0	0
Roots & tubers	0	0	0	0	0
Sugar crops	0	0	0	0	0
Vegetables	0.02	0.09	0.03	0.08	0.14
**Turkey**					
Cereals	0.08	0.2	0.04	0.04	0.06
Fiber	0	0	0	0	0
Fruit	0.06	0.17	0.04	0.03	0.04
Livestock	0.95	2.94	0.74	0.49	0.35

Oil crops	0.04	0.12	0.03	0.02	0.02
Pulses	0	0	0	0	0
Roots & tubers	0	0	0	0	0
Sugar crops	0.04	0.12	0.02	0.02	0.01
Vegetables	0.64	1.9	0.46	0.39	0.42
**Turkmenistan**					
Cereals	0	0	0	0	0
Fiber	0	0	0	0	0
Fruit	0	0	0	0	0
Livestock	0.01	0.2	0.09	0.13	0.2
Oil crops	0	0	0	0	0
Pulses	0	0	0	0	0
Roots & tubers	0	0	0	0	0
Sugar crops	0	0	0	0	0
Vegetables	0	0.04	0.02	0.02	0.03
**Uganda**					
Cereals	0.04	0.06	0.01	0	0
Fiber	0	0	0	0	0
Fruit	1.09	2.02	0.21	0	0
Livestock	0.1	0.26	0.03	0	0
Oil crops	0	0	0	0	0
Pulses	0	0	0	0	0
Roots & tubers	3.99	8.48	0.99	0	0
Sugar crops	0	0	0	0	0
Vegetables	0	0.01	0	0	0
**Ukraine**					
Cereals	0.05	0.19	0.08	0.26	0.1
Fiber	0	0	0	0	0
Fruit	0	0.01	0	0.01	0
Livestock	0.46	1.74	0.73	2.06	0.69
Oil crops	0.01	0.02	0.01	0.04	0.01
Pulses	0	0.01	0	0.02	0.01
Roots & tubers	0	0	0	0	0
Sugar crops	0.01	0.06	0.03	0.12	0.05
Vegetables	0.04	0.16	0.07	0.23	0.09
**United Arab Emirates**					
Cereals	0	0	0	0	0
Fiber	0	0	0	0	0
Fruit	0.01	0.03	0.01	0	0
Livestock	0	0.02	0.01	0	0
Oil crops	0	0	0	0	0
Pulses	0	0	0	0	0
Roots & tubers	0	0	0	0	0
Sugar crops	0	0	0	0	0
Vegetables	0.01	0.02	0.01	0	0
**United Kingdom**					

Cereals	0	0.01	0.01	0.02	0.01
Fiber	0	0	0	0	0
Fruit	0	0	0	0	0
Livestock	0.28	1.75	1.1	2.41	0.51
Oil crops	0	0	0	0.01	0
Pulses	0	0.01	0.01	0.04	0.01
Roots & tubers	0	0	0	0	0
Sugar crops	0	0.02	0.02	0.06	0.02
Vegetables	0	0.01	0	0.02	0.01
**United Republic of Tanzania**					
Cereals	0.07	0.19	0.02	0	0
Fiber	0	0	0	0	0
Fruit	0.07	0.19	0.03	0	0
Livestock	0.18	0.37	0.04	0	0
Oil crops	0	0	0	0	0
Pulses	0.01	0.01	0	0	0
Roots & tubers	1.87	4.61	0.59	0	0
Sugar crops	0	0	0	0	0
Vegetables	0.01	0.04	0.01	0	0
**United States of America**					
Cereals	0	1.05	2.33	10.87	10.67
Fiber	0	0	0	0	0
Fruit	0	0.14	0.18	0.41	0.47
Livestock	0.01	3.98	6.37	18.14	12.39
Oil crops	0	0.03	0.08	0.37	0.37
Pulses	0	0	0.01	0.03	0.02
Roots & tubers	0	0.22	0.51	1.87	1.27
Sugar crops	0	0.01	0.03	0.2	0.23
Vegetables	0	0.28	0.56	2.23	1.82
**Uruguay**					
Cereals	0	0	0	0.01	0.02
Fiber	0	0	0	0	0
Fruit	0	0	0	0	0.01
Livestock	0	0.02	0.03	0.11	0.48
Oil crops	0	0	0	0	0.01
Pulses	0	0	0	0	0
Roots & tubers	0	0.01	0.02	0.07	0.24
Sugar crops	0	0	0	0	0
Vegetables	0	0	0	0	0.01
**Uzbekistan**					
Cereals	0	0.01	0	0	0
Fiber	0	0	0	0	0
Fruit	0	0.02	0.01	0.01	0.01
Livestock	0.12	0.87	0.36	0.25	0.18
Oil crops	0	0	0	0	0
Pulses	0	0	0	0	0

Roots & tubers	0	0	0	0	0
Sugar crops	0	0	0	0	0
Vegetables	0.04	0.25	0.1	0.09	0.1
**Venezuela (Bolivarian Republic of)**					
Cereals	0	0.02	0.02	0.04	0.12
Fiber	0	0	0	0	0
Fruit	0	0.01	0.01	0.03	0.15
Livestock	0.01	0.14	0.14	0.2	0.5
Oil crops	0	0	0	0	0
Pulses	0	0	0	0	0
Roots & tubers	0	0.01	0.01	0.02	0.05
Sugar crops	0	0	0	0	0
Vegetables	0	0.01	0.01	0.01	0.05
**Viet Nam**					
Cereals	0.12	0.2	0.01	0	0
Fiber	0	0	0	0	0
Fruit	0.04	0.08	0.01	0.02	0
Livestock	0.18	0.29	0.01	0.06	0
Oil crops	0	0	0	0	0
Pulses	0	0.01	0	0	0
Roots & tubers	1.83	4.08	0.29	1.25	0
Sugar crops	0	0	0	0	0
Vegetables	0	0	0	0	0
**Yemen**					
Cereals	0	0	0	0	0
Fiber	0	0	0	0	0
Fruit	0	0.01	0	0	0
Livestock	0.05	0.13	0.02	0.01	0
Oil crops	0	0	0	0	0
Pulses	0	0	0	0	0
Roots & tubers	0	0	0	0	0
Sugar crops	0	0	0	0	0
Vegetables	0.01	0.05	0.02	0.01	0
**Zambia**					
Cereals	0.02	0.06	0.01	0.02	0
Fiber	0	0	0	0	0
Fruit	0	0	0	0	0
Livestock	0.02	0.05	0.01	0.01	0
Oil crops	0	0	0	0	0
Pulses	0	0	0	0	0
Roots & tubers	0.13	0.22	0.02	0.05	0
Sugar crops	0	0	0	0	0
Vegetables	0	0	0	0	0
**Zimbabwe**					
Cereals	0.01	0.07	0.01	0	0

Fiber	0	0	0	0	0
Fruit	0	0.01	0	0	0
Livestock	0.07	0.11	0.01	0	0
Oil crops	0	0	0	0	0
Pulses	0	0	0	0	0
Roots & tubers	0	0.01	0	0	0
Sugar crops	0	0	0	0	0
Vegetables	0	0.01	0	0	0

	**Vitamin B12 production (T)**				
	**< 2 ha**	**2 - 20 ha**	**20 - 50 ha**	**50 - 200 ha**	**> 200 ha**
**Afghanistan**					
Cereals	0	0	0	0	0
Fiber	0	0	0	0	0
Fruit	0	0	0	0	0
Livestock	0	0.01	0	0	0
Oil crops	0	0	0	0	0
Pulses	0	0	0	0	0
Roots & tubers	0	0	0	0	0
Sugar crops	0	0	0	0	0
Vegetables	0	0	0	0	0
**Albania**					
Cereals	0	0	0	0	0
Fiber	0	0	0	0	0
Fruit	0	0	0	0	0
Livestock	0	0	0	0	0
Oil crops	0	0	0	0	0
Pulses	0	0	0	0	0
Roots & tubers	0	0	0	0	0
Sugar crops	0	0	0	0	0
Vegetables	0	0	0	0	0
**Algeria**					
Cereals	0	0	0	0	0
Fiber	0	0	0	0	0
Fruit	0	0	0	0	0
Livestock	0	0.01	0	0	0
Oil crops	0	0	0	0	0
Pulses	0	0	0	0	0
Roots & tubers	0	0	0	0	0
Sugar crops	0	0	0	0	0
Vegetables	0	0	0	0	0
**Angola**					
Cereals	0	0	0	0	0
Fiber	0	0	0	0	0
Fruit	0	0	0	0	0

Livestock	0	0	0	0	0
Oil crops	0	0	0	0	0
Pulses	0	0	0	0	0
Roots & tubers	0	0	0	0	0
Sugar crops	0	0	0	0	0
Vegetables	0	0	0	0	0
**Argentina**					
Cereals	0	0	0	0	0
Fiber	0	0	0	0	0
Fruit	0	0	0	0	0
Livestock	0	0.01	0.01	0.02	0.06
Oil crops	0	0	0	0	0
Pulses	0	0	0	0	0
Roots & tubers	0	0	0	0	0
Sugar crops	0	0	0	0	0
Vegetables	0	0	0	0	0
**Armenia**					
Cereals	0	0	0	0	0
Fiber	0	0	0	0	0
Fruit	0	0	0	0	0
Livestock	0	0	0	0	0
Oil crops	0	0	0	0	0
Pulses	0	0	0	0	0
Roots & tubers	0	0	0	0	0
Sugar crops	0	0	0	0	0
Vegetables	0	0	0	0	0
**Australia**					
Cereals	0	0	0	0	0
Fiber	0	0	0	0	0
Fruit	0	0	0	0	0
Livestock	0	0	0	0.01	0.07
Oil crops	0	0	0	0	0
Pulses	0	0	0	0	0
Roots & tubers	0	0	0	0	0
Sugar crops	0	0	0	0	0
Vegetables	0	0	0	0	0
**Austria**					
Cereals	0	0	0	0	0
Fiber	0	0	0	0	0
Fruit	0	0	0	0	0
Livestock	0	0.01	0	0.01	0
Oil crops	0	0	0	0	0
Pulses	0	0	0	0	0
Roots & tubers	0	0	0	0	0
Sugar crops	0	0	0	0	0
Vegetables	0	0	0	0	0

**Azerbaijan**					
Cereals	0	0	0	0	0
Fiber	0	0	0	0	0
Fruit	0	0	0	0	0
Livestock	0	0	0	0	0
Oil crops	0	0	0	0	0
Pulses	0	0	0	0	0
Roots & tubers	0	0	0	0	0
Sugar crops	0	0	0	0	0
Vegetables	0	0	0	0	0
**Bangladesh**					
Cereals	0	0	0	0	0
Fiber	0	0	0	0	0
Fruit	0	0	0	0	0
Livestock	0.01	0.01	0	0	0
Oil crops	0	0	0	0	0
Pulses	0	0	0	0	0
Roots & tubers	0	0	0	0	0
Sugar crops	0	0	0	0	0
Vegetables	0	0	0	0	0
**Belarus**					
Cereals	0	0	0	0	0
Fiber	0	0	0	0	0
Fruit	0	0	0	0	0
Livestock	0	0.01	0	0.01	0
Oil crops	0	0	0	0	0
Pulses	0	0	0	0	0
Roots & tubers	0	0	0	0	0
Sugar crops	0	0	0	0	0
Vegetables	0	0	0	0	0
**Belgium**					
Cereals	0	0	0	0	0
Fiber	0	0	0	0	0
Fruit	0	0	0	0	0
Livestock	0	0.01	0	0.01	0
Oil crops	0	0	0	0	0
Pulses	0	0	0	0	0
Roots & tubers	0	0	0	0	0
Sugar crops	0	0	0	0	0
Vegetables	0	0	0	0	0
**Belize**					
Cereals	0	0	0	0	0
Fiber	0	0	0	0	0
Fruit	0	0	0	0	0
Livestock	0	0	0	0	0
Oil crops	0	0	0	0	0

Pulses	0	0	0	0	0
Roots & tubers	0	0	0	0	0
Sugar crops	0	0	0	0	0
Vegetables	0	0	0	0	0
**Benin**					
Cereals	0	0	0	0	0
Fiber	0	0	0	0	0
Fruit	0	0	0	0	0
Livestock	0	0	0	0	0
Oil crops	0	0	0	0	0
Pulses	0	0	0	0	0
Roots & tubers	0	0	0	0	0
Sugar crops	0	0	0	0	0
Vegetables	0	0	0	0	0
**Bhutan**					
Cereals	0	0	0	0	0
Fiber	0	0	0	0	0
Fruit	0	0	0	0	0
Livestock	0	0	0	0	0
Oil crops	0	0	0	0	0
Pulses	0	0	0	0	0
Roots & tubers	0	0	0	0	0
Sugar crops	0	0	0	0	0
Vegetables	0	0	0	0	0
**Bolivia (Plurinational State of)**					
Cereals	0	0	0	0	0
Fiber	0	0	0	0	0
Fruit	0	0	0	0	0
Livestock	0	0	0	0	0
Oil crops	0	0	0	0	0
Pulses	0	0	0	0	0
Roots & tubers	0	0	0	0	0
Sugar crops	0	0	0	0	0
Vegetables	0	0	0	0	0
**Bosnia and Herzegovina**					
Cereals	0	0	0	0	0
Fiber	0	0	0	0	0
Fruit	0	0	0	0	0
Livestock	0	0	0	0	0
Oil crops	0	0	0	0	0
Pulses	0	0	0	0	0
Roots & tubers	0	0	0	0	0
Sugar crops	0	0	0	0	0
Vegetables	0	0	0	0	0
**Botswana**					

Cereals	0	0	0	0	0
Fiber	0	0	0	0	0
Fruit	0	0	0	0	0
Livestock	0	0	0	0	0
Oil crops	0	0	0	0	0
Pulses	0	0	0	0	0
Roots & tubers	0	0	0	0	0
Sugar crops	0	0	0	0	0
Vegetables	0	0	0	0	0
**Brazil**					
Cereals	0	0	0	0	0
Fiber	0	0	0	0	0
Fruit	0	0	0	0	0
Livestock	0	0.03	0.02	0.05	0.17
Oil crops	0	0	0	0	0
Pulses	0	0	0	0	0
Roots & tubers	0	0	0	0	0
Sugar crops	0	0	0	0	0
Vegetables	0	0	0	0	0
**Brunei Darussalam**					
Cereals	0	0	0	0	0
Fiber	0	0	0	0	0
Fruit	0	0	0	0	0
Livestock	0	0	0	0	0
Oil crops	0	0	0	0	0
Pulses	0	0	0	0	0
Roots & tubers	0	0	0	0	0
Sugar crops	0	0	0	0	0
Vegetables	0	0	0	0	0
**Bulgaria**					
Cereals	0	0	0	0	0
Fiber	0	0	0	0	0
Fruit	0	0	0	0	0
Livestock	0	0	0	0	0
Oil crops	0	0	0	0	0
Pulses	0	0	0	0	0
Roots & tubers	0	0	0	0	0
Sugar crops	0	0	0	0	0
Vegetables	0	0	0	0	0
**Burkina Faso**					
Cereals	0	0	0	0	0
Fiber	0	0	0	0	0
Fruit	0	0	0	0	0
Livestock	0	0	0	0	0
Oil crops	0	0	0	0	0
Pulses	0	0	0	0	0

Roots & tubers	0	0	0	0	0
Sugar crops	0	0	0	0	0
Vegetables	0	0	0	0	0
**Burundi**					
Cereals	0	0	0	0	0
Fiber	0	0	0	0	0
Fruit	0	0	0	0	0
Livestock	0	0	0	0	0
Oil crops	0	0	0	0	0
Pulses	0	0	0	0	0
Roots & tubers	0	0	0	0	0
Sugar crops	0	0	0	0	0
Vegetables	0	0	0	0	0
**Cambodia**					
Cereals	0	0	0	0	0
Fiber	0	0	0	0	0
Fruit	0	0	0	0	0
Livestock	0	0	0	0	0
Oil crops	0	0	0	0	0
Pulses	0	0	0	0	0
Roots & tubers	0	0	0	0	0
Sugar crops	0	0	0	0	0
Vegetables	0	0	0	0	0
**Cameroon**					
Cereals	0	0	0	0	0
Fiber	0	0	0	0	0
Fruit	0	0	0	0	0
Livestock	0	0	0	0	0
Oil crops	0	0	0	0	0
Pulses	0	0	0	0	0
Roots & tubers	0	0	0	0	0
Sugar crops	0	0	0	0	0
Vegetables	0	0	0	0	0
**Canada**					
Cereals	0	0	0	0	0
Fiber	0	0	0	0	0
Fruit	0	0	0	0	0
Livestock	0	0.01	0.01	0.03	0.02
Oil crops	0	0	0	0	0
Pulses	0	0	0	0	0
Roots & tubers	0	0	0	0	0
Sugar crops	0	0	0	0	0
Vegetables	0	0	0	0	0
**Central African Republic**					
Cereals	0	0	0	0	0
Fiber	0	0	0	0	0

Fruit	0	0	0	0	0
Oil crops	0	0	0	0	0
Pulses	0	0	0	0	0
Roots & tubers	0	0	0	0	0
Sugar crops	0	0	0	0	0
Vegetables	0	0	0	0	0
**Chad**					
Cereals	0	0	0	0	0
Fiber	0	0	0	0	0
Fruit	0	0	0	0	0
Livestock	0	0	0	0	0
Oil crops	0	0	0	0	0
Pulses	0	0	0	0	0
Roots & tubers	0	0	0	0	0
Sugar crops	0	0	0	0	0
Vegetables	0	0	0	0	0
**Chile**					
Cereals	0	0	0	0	0
Fiber	0	0	0	0	0
Fruit	0	0	0	0	0
Livestock	0	0	0	0	0.01
Oil crops	0	0	0	0	0
Pulses	0	0	0	0	0
Roots & tubers	0	0	0	0	0
Sugar crops	0	0	0	0	0
Vegetables	0	0	0	0	0
**China**					
Cereals	0	0	0	0	0
Fiber	0	0	0	0	0
Fruit	0	0	0	0	0
Livestock	0.45	0.19	0	0.08	0
Oil crops	0	0	0	0	0
Pulses	0	0	0	0	0
Roots & tubers	0	0	0	0	0
Sugar crops	0	0	0	0	0
Vegetables	0	0	0	0	0
**Colombia**					
Cereals	0	0	0	0	0
Fiber	0	0	0	0	0
Fruit	0	0	0	0	0
Livestock	0	0	0	0.01	0.02
Oil crops	0	0	0	0	0
Pulses	0	0	0	0	0
Roots & tubers	0	0	0	0	0
Sugar crops	0	0	0	0	0
Vegetables	0	0	0	0	0

**Congo**					
Cereals	0	0	0	0	0
Fiber	0	0	0	0	0
Fruit	0	0	0	0	0
Livestock	0	0	0	0	0
Oil crops	0	0	0	0	0
Pulses	0	0	0	0	0
Roots & tubers	0	0	0	0	0
Sugar crops	0	0	0	0	0
Vegetables	0	0	0	0	0
**Costa Rica**					
Cereals	0	0	0	0	0
Fiber	0	0	0	0	0
Fruit	0	0	0	0	0
Livestock	0	0	0	0	0
Oil crops	0	0	0	0	0
Pulses	0	0	0	0	0
Roots & tubers	0	0	0	0	0
Sugar crops	0	0	0	0	0
Vegetables	0	0	0	0	0
**Croatia**					
Cereals	0	0	0	0	0
Fiber	0	0	0	0	0
Fruit	0	0	0	0	0
Livestock	0	0	0	0	0
Oil crops	0	0	0	0	0
Pulses	0	0	0	0	0
Roots & tubers	0	0	0	0	0
Sugar crops	0	0	0	0	0
Vegetables	0	0	0	0	0
**Cuba**					
Cereals	0	0	0	0	0
Fiber	0	0	0	0	0
Fruit	0	0	0	0	0
Livestock	0	0	0	0	0
Oil crops	0	0	0	0	0
Pulses	0	0	0	0	0
Roots & tubers	0	0	0	0	0
Sugar crops	0	0	0	0	0
Vegetables	0	0	0	0	0
**Cyprus**					
Cereals	0	0	0	0	0
Fiber	0	0	0	0	0
Fruit	0	0	0	0	0
Livestock	0	0	0	0	0
Oil crops	0	0	0	0	0

Pulses	0	0	0	0	0
Roots & tubers	0	0	0	0	0
Sugar crops	0	0	0	0	0
Vegetables	0	0	0	0	0
**Czech Republic**					
Cereals	0	0	0	0	0
Fiber	0	0	0	0	0
Fruit	0	0	0	0	0
Livestock	0	0	0	0.01	0
Oil crops	0	0	0	0	0
Pulses	0	0	0	0	0
Roots & tubers	0	0	0	0	0
Sugar crops	0	0	0	0	0
Vegetables	0	0	0	0	0
**Democratic Republic of the Congo**					
Cereals	0	0	0	0	0
Fiber	0	0	0	0	0
Fruit	0	0	0	0	0
Livestock	0	0	0	0	0
Oil crops	0	0	0	0	0
Pulses	0	0	0	0	0
Roots & tubers	0	0	0	0	0
Sugar crops	0	0	0	0	0
Vegetables	0	0	0	0	0
**Denmark**					
Cereals	0	0	0	0	0
Fiber	0	0	0	0	0
Fruit	0	0	0	0	0
Livestock	0	0.01	0	0.01	0
Oil crops	0	0	0	0	0
Pulses	0	0	0	0	0
Roots & tubers	0	0	0	0	0
Sugar crops	0	0	0	0	0
Vegetables	0	0	0	0	0
**Djibouti**					
Cereals	0	0	0	0	0
Fiber	0	0	0	0	0
Fruit	0	0	0	0	0
Livestock	0	0	0	0	0
Oil crops	0	0	0	0	0
Pulses	0	0	0	0	0
Roots & tubers	0	0	0	0	0
Sugar crops	0	0	0	0	0
Vegetables	0	0	0	0	0
**Dominica**					

Cereals	0	0	0	0	0
Fiber	0	0	0	0	0
Fruit	0	0	0	0	0
Livestock	0	0	0	0	0
Oil crops	0	0	0	0	0
Pulses	0	0	0	0	0
Roots & tubers	0	0	0	0	0
Sugar crops	0	0	0	0	0
Vegetables	0	0	0	0	0
**Ecuador**					
Cereals	0	0	0	0	0
Fiber	0	0	0	0	0
Fruit	0	0	0	0	0
Livestock	0	0	0	0	0.01
Oil crops	0	0	0	0	0
Pulses	0	0	0	0	0
Roots & tubers	0	0	0	0	0
Sugar crops	0	0	0	0	0
Vegetables	0	0	0	0	0
**Egypt**					
Cereals	0	0	0	0	0
Fiber	0	0	0	0	0
Fruit	0	0	0	0	0
Livestock	0	0.02	0.01	0	0
Oil crops	0	0	0	0	0
Pulses	0	0	0	0	0
Roots & tubers	0	0	0	0	0
Sugar crops	0	0	0	0	0
Vegetables	0	0	0	0	0
**El Salvador**					
Cereals	0	0	0	0	0
Fiber	0	0	0	0	0
Fruit	0	0	0	0	0
Livestock	0	0	0	0	0
Oil crops	0	0	0	0	0
Pulses	0	0	0	0	0
Roots & tubers	0	0	0	0	0
Sugar crops	0	0	0	0	0
Vegetables	0	0	0	0	0
**Equatorial Guinea**					
Cereals	0	0	0	0	0
Fiber	0	0	0	0	0
Fruit	0	0	0	0	0
Livestock	0	0	0	0	0
Oil crops	0	0	0	0	0
Pulses	0	0	0	0	0

Roots & tubers	0	0	0	0	0
Sugar crops	0	0	0	0	0
Vegetables	0	0	0	0	0
**Eritrea**					
Cereals	0	0	0	0	0
Fiber	0	0	0	0	0
Fruit	0	0	0	0	0
Livestock	0	0	0	0	0
Oil crops	0	0	0	0	0
Pulses	0	0	0	0	0
Roots & tubers	0	0	0	0	0
Sugar crops	0	0	0	0	0
Vegetables	0	0	0	0	0
**Estonia**					
Cereals	0	0	0	0	0
Fiber	0	0	0	0	0
Fruit	0	0	0	0	0
Livestock	0	0	0	0	0
Oil crops	0	0	0	0	0
Pulses	0	0	0	0	0
Roots & tubers	0	0	0	0	0
Sugar crops	0	0	0	0	0
Vegetables	0	0	0	0	0
**Ethiopia**					
Cereals	0	0	0	0	0
Fiber	0	0	0	0	0
Fruit	0	0	0	0	0
Livestock	0.01	0.01	0	0	0
Oil crops	0	0	0	0	0
Pulses	0	0	0	0	0
Roots & tubers	0	0	0	0	0
Sugar crops	0	0	0	0	0
Vegetables	0	0	0	0	0
**Finland**					
Cereals	0	0	0	0	0
Fiber	0	0	0	0	0
Fruit	0	0	0	0	0
Livestock	0	0	0	0	0
Oil crops	0	0	0	0	0
Pulses	0	0	0	0	0
Roots & tubers	0	0	0	0	0
Sugar crops	0	0	0	0	0
Vegetables	0	0	0	0	0
**France**					
Cereals	0	0	0	0	0
Fiber	0	0	0	0	0

Fruit	0	0	0	0	0
Livestock	0.01	0.05	0.02	0.05	0.01
Oil crops	0	0	0	0	0
Pulses	0	0	0	0	0
Roots & tubers	0	0	0	0	0
Sugar crops	0	0	0	0	0
Vegetables	0	0	0	0	0
**Gabon**					
Cereals	0	0	0	0	0
Fiber	0	0	0	0	0
Fruit	0	0	0	0	0
Livestock	0	0	0	0	0
Oil crops	0	0	0	0	0
Pulses	0	0	0	0	0
Roots & tubers	0	0	0	0	0
Sugar crops	0	0	0	0	0
Vegetables	0	0	0	0	0
**Gambia**					
Cereals	0	0	0	0	0
Fiber	0	0	0	0	0
Fruit	0	0	0	0	0
Livestock	0	0	0	0	0
Oil crops	0	0	0	0	0
Pulses	0	0	0	0	0
Roots & tubers	0	0	0	0	0
Sugar crops	0	0	0	0	0
Vegetables	0	0	0	0	0
**Georgia**					
Cereals	0	0	0	0	0
Fiber	0	0	0	0	0
Fruit	0	0	0	0	0
Livestock	0	0	0	0	0
Oil crops	0	0	0	0	0
Pulses	0	0	0	0	0
Roots & tubers	0	0	0	0	0
Sugar crops	0	0	0	0	0
Vegetables	0	0	0	0	0
**Germany**					
Cereals	0	0	0	0	0
Fiber	0	0	0	0	0
Fruit	0	0	0	0	0
Livestock	0.01	0.03	0.02	0.07	0.03
Oil crops	0	0	0	0	0
Pulses	0	0	0	0	0
Roots & tubers	0	0	0	0	0
Sugar crops	0	0	0	0	0

Vegetables	0	0	0	0	0
**Ghana**					
Cereals	0	0	0	0	0
Fiber	0	0	0	0	0
Fruit	0	0	0	0	0
Livestock	0	0	0	0	0
Oil crops	0	0	0	0	0
Pulses	0	0	0	0	0
Roots & tubers	0	0	0	0	0
Sugar crops	0	0	0	0	0
Vegetables	0	0	0	0	0
**Greece**					
Cereals	0	0	0	0	0
Fiber	0	0	0	0	0
Fruit	0	0	0	0	0
Livestock	0	0.01	0	0	0
Oil crops	0	0	0	0	0
Pulses	0	0	0	0	0
Roots & tubers	0	0	0	0	0
Sugar crops	0	0	0	0	0
Vegetables	0	0	0	0	0
**Grenada**					
Cereals	0	0	0	0	0
Fiber	0	0	0	0	0
Fruit	0	0	0	0	0
Livestock	0	0	0	0	0
Oil crops	0	0	0	0	0
Pulses	0	0	0	0	0
Roots & tubers	0	0	0	0	0
Sugar crops	0	0	0	0	0
Vegetables	0	0	0	0	0
**Guatemala**					
Cereals	0	0	0	0	0
Fiber	0	0	0	0	0
Fruit	0	0	0	0	0
Livestock	0	0	0	0	0
Oil crops	0	0	0	0	0
Pulses	0	0	0	0	0
Roots & tubers	0	0	0	0	0
Sugar crops	0	0	0	0	0
Vegetables	0	0	0	0	0
**Guinea**					
Cereals	0	0	0	0	0
Fiber	0	0	0	0	0
Fruit	0	0	0	0	0
Livestock	0	0	0	0	0

Oil crops	0	0	0	0	0
Pulses	0	0	0	0	0
Roots & tubers	0	0	0	0	0
Sugar crops	0	0	0	0	0
Vegetables	0	0	0	0	0
**Guinea-Bissau**					
Cereals	0	0	0	0	0
Fiber	0	0	0	0	0
Fruit	0	0	0	0	0
Livestock	0	0	0	0	0
Oil crops	0	0	0	0	0
Pulses	0	0	0	0	0
Roots & tubers	0	0	0	0	0
Sugar crops	0	0	0	0	0
Vegetables	0	0	0	0	0
**Guyana**					
Cereals	0	0	0	0	0
Fiber	0	0	0	0	0
Fruit	0	0	0	0	0
Livestock	0	0	0	0	0
Oil crops	0	0	0	0	0
Pulses	0	0	0	0	0
Roots & tubers	0	0	0	0	0
Sugar crops	0	0	0	0	0
Vegetables	0	0	0	0	0
**Haiti**					
Cereals	0	0	0	0	0
Fiber	0	0	0	0	0
Fruit	0	0	0	0	0
Livestock	0	0	0	0	0
Oil crops	0	0	0	0	0
Pulses	0	0	0	0	0
Roots & tubers	0	0	0	0	0
Sugar crops	0	0	0	0	0
Vegetables	0	0	0	0	0
**Honduras**					
Cereals	0	0	0	0	0
Fiber	0	0	0	0	0
Fruit	0	0	0	0	0
Livestock	0	0	0	0	0
Oil crops	0	0	0	0	0
Pulses	0	0	0	0	0
Roots & tubers	0	0	0	0	0
Sugar crops	0	0	0	0	0
Vegetables	0	0	0	0	0
**Hungary**					

Cereals	0	0	0	0	0
Fiber	0	0	0	0	0
Fruit	0	0	0	0	0
Livestock	0	0	0	0	0
Oil crops	0	0	0	0	0
Pulses	0	0	0	0	0
Roots & tubers	0	0	0	0	0
Sugar crops	0	0	0	0	0
Vegetables	0	0	0	0	0
**Iceland**					
Cereals	0	0	0	0	0
Fiber	0	0	0	0	0
Fruit	0	0	0	0	0
Livestock	0	0	0	0	0
Oil crops	0	0	0	0	0
Pulses	0	0	0	0	0
Roots & tubers	0	0	0	0	0
Sugar crops	0	0	0	0	0
Vegetables	0	0	0	0	0
**India**					
Cereals	0	0	0	0	0
Fiber	0	0	0	0	0
Fruit	0	0	0	0	0
Livestock	0.12	0.27	0.03	0.01	0
Oil crops	0	0	0	0	0
Pulses	0	0	0	0	0
Roots & tubers	0	0	0	0	0
Sugar crops	0	0	0	0	0
Vegetables	0	0	0	0	0
**Indonesia**					
Cereals	0	0	0	0	0
Fiber	0	0	0	0	0
Fruit	0	0	0	0	0
Livestock	0.01	0.01	0	0	0
Oil crops	0	0	0	0	0
Pulses	0	0	0	0	0
Roots & tubers	0	0	0	0	0
Sugar crops	0	0	0	0	0
Vegetables	0	0	0	0	0
**Iran (Islamic Republic of)**					
Cereals	0	0	0	0	0
Fiber	0	0	0	0	0
Fruit	0	0	0	0	0
Livestock	0.01	0.03	0	0.01	0
Oil crops	0	0	0	0	0
Pulses	0	0	0	0	0

Roots & tubers	0	0	0	0	0
Sugar crops	0	0	0	0	0
Vegetables	0	0	0	0	0
**Iraq**					
Cereals	0	0	0	0	0
Fiber	0	0	0	0	0
Fruit	0	0	0	0	0
Livestock	0	0	0	0	0
Oil crops	0	0	0	0	0
Pulses	0	0	0	0	0
Roots & tubers	0	0	0	0	0
Sugar crops	0	0	0	0	0
Vegetables	0	0	0	0	0
**Ireland**					
Cereals	0	0	0	0	0
Fiber	0	0	0	0	0
Fruit	0	0	0	0	0
Livestock	0	0.01	0	0.01	0
Oil crops	0	0	0	0	0
Pulses	0	0	0	0	0
Roots & tubers	0	0	0	0	0
Sugar crops	0	0	0	0	0
Vegetables	0	0	0	0	0
**Israel**					
Cereals	0	0	0	0	0
Fiber	0	0	0	0	0
Fruit	0	0	0	0	0
Livestock	0	0.01	0	0	0
Oil crops	0	0	0	0	0
Pulses	0	0	0	0	0
Roots & tubers	0	0	0	0	0
Sugar crops	0	0	0	0	0
Vegetables	0	0	0	0	0
**Italy**					
Cereals	0	0	0	0	0
Fiber	0	0	0	0	0
Fruit	0	0	0	0	0
Livestock	0	0.02	0.01	0.03	0
Oil crops	0	0	0	0	0
Pulses	0	0	0	0	0
Roots & tubers	0	0	0	0	0
Sugar crops	0	0	0	0	0
Vegetables	0	0	0	0	0
**Ivory Coast**					
Cereals	0	0	0	0	0
Fiber	0	0	0	0	0

Fruit	0	0	0	0	0
Livestock	0	0	0	0	0
Oil crops	0	0	0	0	0
Pulses	0	0	0	0	0
Roots & tubers	0	0	0	0	0
Sugar crops	0	0	0	0	0
Vegetables	0	0	0	0	0
**Jamaica**					
Cereals	0	0	0	0	0
Fiber	0	0	0	0	0
Fruit	0	0	0	0	0
Livestock	0	0	0	0	0
Oil crops	0	0	0	0	0
Pulses	0	0	0	0	0
Roots & tubers	0	0	0	0	0
Sugar crops	0	0	0	0	0
Vegetables	0	0	0	0	0
**Japan**					
Cereals	0	0	0	0	0
Fiber	0	0	0	0	0
Fruit	0	0	0	0	0
Livestock	0.02	0.02	0.01	0.01	0
Oil crops	0	0	0	0	0
Pulses	0	0	0	0	0
Roots & tubers	0	0	0	0	0
Sugar crops	0	0	0	0	0
Vegetables	0	0	0	0	0
**Jordan**					
Cereals	0	0	0	0	0
Fiber	0	0	0	0	0
Fruit	0	0	0	0	0
Livestock	0	0	0	0	0
Oil crops	0	0	0	0	0
Pulses	0	0	0	0	0
Roots & tubers	0	0	0	0	0
Sugar crops	0	0	0	0	0
Vegetables	0	0	0	0	0
**Kazakhstan**					
Cereals	0	0	0	0	0
Fiber	0	0	0	0	0
Fruit	0	0	0	0	0
Livestock	0	0.01	0	0	0
Oil crops	0	0	0	0	0
Pulses	0	0	0	0	0
Roots & tubers	0	0	0	0	0
Sugar crops	0	0	0	0	0

Vegetables	0	0	0	0	0
**Kenya**					
Cereals	0	0	0	0	0
Fiber	0	0	0	0	0
Fruit	0	0	0	0	0
Livestock	0.01	0.01	0	0	0
Oil crops	0	0	0	0	0
Pulses	0	0	0	0	0
Roots & tubers	0	0	0	0	0
Sugar crops	0	0	0	0	0
Vegetables	0	0	0	0	0
**Kuwait**					
Cereals	0	0	0	0	0
Fiber	0	0	0	0	0
Fruit	0	0	0	0	0
Livestock	0	0	0	0	0
Oil crops	0	0	0	0	0
Pulses	0	0	0	0	0
Roots & tubers	0	0	0	0	0
Sugar crops	0	0	0	0	0
Vegetables	0	0	0	0	0
**Kyrgyzstan**					
Cereals	0	0	0	0	0
Fiber	0	0	0	0	0
Fruit	0	0	0	0	0
Livestock	0	0	0	0	0
Oil crops	0	0	0	0	0
Pulses	0	0	0	0	0
Roots & tubers	0	0	0	0	0
Sugar crops	0	0	0	0	0
Vegetables	0	0	0	0	0
**Lao People's Democratic Republic**					
Cereals	0	0	0	0	0
Fiber	0	0	0	0	0
Fruit	0	0	0	0	0
Livestock	0	0	0	0	0
Oil crops	0	0	0	0	0
Pulses	0	0	0	0	0
Roots & tubers	0	0	0	0	0
Sugar crops	0	0	0	0	0
Vegetables	0	0	0	0	0
**Latvia**					
Cereals	0	0	0	0	0
Fiber	0	0	0	0	0
Fruit	0	0	0	0	0

Livestock	0	0	0	0	0
Oil crops	0	0	0	0	0
Pulses	0	0	0	0	0
Roots & tubers	0	0	0	0	0
Sugar crops	0	0	0	0	0
Vegetables	0	0	0	0	0
**Lebanon**					
Cereals	0	0	0	0	0
Fiber	0	0	0	0	0
Fruit	0	0	0	0	0
Livestock	0	0	0	0	0
Oil crops	0	0	0	0	0
Pulses	0	0	0	0	0
Roots & tubers	0	0	0	0	0
Sugar crops	0	0	0	0	0
Vegetables	0	0	0	0	0
**Lesotho**					
Cereals	0	0	0	0	0
Fiber	0	0	0	0	0
Fruit	0	0	0	0	0
Livestock	0	0	0	0	0
Oil crops	0	0	0	0	0
Pulses	0	0	0	0	0
Roots & tubers	0	0	0	0	0
Sugar crops	0	0	0	0	0
Vegetables	0	0	0	0	0
**Liberia**					
Cereals	0	0	0	0	0
Fiber	0	0	0	0	0
Fruit	0	0	0	0	0
Livestock	0	0	0	0	0
Oil crops	0	0	0	0	0
Pulses	0	0	0	0	0
Roots & tubers	0	0	0	0	0
Sugar crops	0	0	0	0	0
Vegetables	0	0	0	0	0
**Libya**					
Cereals	0	0	0	0	0
Fiber	0	0	0	0	0
Fruit	0	0	0	0	0
Livestock	0	0	0	0	0
Oil crops	0	0	0	0	0
Pulses	0	0	0	0	0
Roots & tubers	0	0	0	0	0
Sugar crops	0	0	0	0	0
Vegetables	0	0	0	0	0

**Lithuania**					
Cereals	0	0	0	0	0
Fiber	0	0	0	0	0
Fruit	0	0	0	0	0
Livestock	0	0	0	0	0
Oil crops	0	0	0	0	0
Pulses	0	0	0	0	0
Roots & tubers	0	0	0	0	0
Sugar crops	0	0	0	0	0
Vegetables	0	0	0	0	0
**Luxembourg**					
Cereals	0	0	0	0	0
Fiber	0	0	0	0	0
Fruit	0	0	0	0	0
Livestock	0	0	0	0	0
Oil crops	0	0	0	0	0
Pulses	0	0	0	0	0
Roots & tubers	0	0	0	0	0
Sugar crops	0	0	0	0	0
Vegetables	0	0	0	0	0
**Madagascar**					
Cereals	0	0	0	0	0
Fiber	0	0	0	0	0
Fruit	0	0	0	0	0
Livestock	0	0	0	0	0
Oil crops	0	0	0	0	0
Pulses	0	0	0	0	0
Roots & tubers	0	0	0	0	0
Sugar crops	0	0	0	0	0
Vegetables	0	0	0	0	0
**Malawi**					
Cereals	0	0	0	0	0
Fiber	0	0	0	0	0
Fruit	0	0	0	0	0
Livestock	0	0	0	0	0
Oil crops	0	0	0	0	0
Pulses	0	0	0	0	0
Roots & tubers	0	0	0	0	0
Sugar crops	0	0	0	0	0
Vegetables	0	0	0	0	0
**Malaysia**					
Cereals	0	0	0	0	0
Fiber	0	0	0	0	0
Fruit	0	0	0	0	0
Livestock	0	0	0	0	0
Oil crops	0	0	0	0	0

Pulses	0	0	0	0	0
Roots & tubers	0	0	0	0	0
Sugar crops	0	0	0	0	0
Vegetables	0	0	0	0	0
**Mali**					
Cereals	0	0	0	0	0
Fiber	0	0	0	0	0
Fruit	0	0	0	0	0
Livestock	0	0	0	0	0
Oil crops	0	0	0	0	0
Pulses	0	0	0	0	0
Roots & tubers	0	0	0	0	0
Sugar crops	0	0	0	0	0
Vegetables	0	0	0	0	0
**Mauritania**					
Cereals	0	0	0	0	0
Fiber	0	0	0	0	0
Fruit	0	0	0	0	0
Livestock	0	0	0	0	0
Oil crops	0	0	0	0	0
Pulses	0	0	0	0	0
Roots & tubers	0	0	0	0	0
Sugar crops	0	0	0	0	0
Vegetables	0	0	0	0	0
**Mauritius**					
Cereals	0	0	0	0	0
Fiber	0	0	0	0	0
Fruit	0	0	0	0	0
Livestock	0	0	0	0	0
Oil crops	0	0	0	0	0
Pulses	0	0	0	0	0
Roots & tubers	0	0	0	0	0
Sugar crops	0	0	0	0	0
Vegetables	0	0	0	0	0
**Mexico**					
Cereals	0	0	0	0	0
Fiber	0	0	0	0	0
Fruit	0	0	0	0	0
Livestock	0.01	0.02	0.02	0.02	0.02
Oil crops	0	0	0	0	0
Pulses	0	0	0	0	0
Roots & tubers	0	0	0	0	0
Sugar crops	0	0	0	0	0
Vegetables	0	0	0	0	0
**Mongolia**					
Cereals	0	0	0	0	0

Fiber	0	0	0	0	0
Fruit	0	0	0	0	0
Livestock	0	0	0	0	0
Oil crops	0	0	0	0	0
Pulses	0	0	0	0	0
Roots & tubers	0	0	0	0	0
Sugar crops	0	0	0	0	0
Vegetables	0	0	0	0	0
**Montenegro**					
Cereals	0	0	0	0	0
Fiber	0	0	0	0	0
Fruit	0	0	0	0	0
Livestock	0	0.01	0	0	0
Oil crops	0	0	0	0	0
Pulses	0	0	0	0	0
Roots & tubers	0	0	0	0	0
Sugar crops	0	0	0	0	0
Vegetables	0	0	0	0	0
**Morocco**					
Cereals	0	0	0	0	0
Fiber	0	0	0	0	0
Fruit	0	0	0	0	0
Livestock	0	0.01	0	0	0
Oil crops	0	0	0	0	0
Pulses	0	0	0	0	0
Roots & tubers	0	0	0	0	0
Sugar crops	0	0	0	0	0
Vegetables	0	0	0	0	0
**Mozambique**					
Cereals	0	0	0	0	0
Fiber	0	0	0	0	0
Fruit	0	0	0	0	0
Livestock	0	0	0	0	0
Oil crops	0	0	0	0	0
Pulses	0	0	0	0	0
Roots & tubers	0	0	0	0	0
Sugar crops	0	0	0	0	0
Vegetables	0	0	0	0	0
**Myanmar**					
Cereals	0	0	0	0	0
Fiber	0	0	0	0	0
Fruit	0	0	0	0	0
Livestock	0	0.01	0	0	0
Oil crops	0	0	0	0	0
Pulses	0	0	0	0	0
Roots & tubers	0	0	0	0	0

Sugar crops	0	0	0	0	0
Vegetables	0	0	0	0	0
**Namibia**					
Cereals	0	0	0	0	0
Fiber	0	0	0	0	0
Fruit	0	0	0	0	0
Livestock	0	0	0	0	0
Oil crops	0	0	0	0	0
Pulses	0	0	0	0	0
Roots & tubers	0	0	0	0	0
Sugar crops	0	0	0	0	0
Vegetables	0	0	0	0	0
**Nepal**					
Cereals	0	0	0	0	0
Fiber	0	0	0	0	0
Fruit	0	0	0	0	0
Livestock	0	0.01	0	0	0
Oil crops	0	0	0	0	0
Pulses	0	0	0	0	0
Roots & tubers	0	0	0	0	0
Sugar crops	0	0	0	0	0
Vegetables	0	0	0	0	0
**Netherlands**					
Cereals	0	0	0	0	0
Fiber	0	0	0	0	0
Fruit	0	0	0	0	0
Livestock	0	0.01	0.01	0.03	0.01
Oil crops	0	0	0	0	0
Pulses	0	0	0	0	0
Roots & tubers	0	0	0	0	0
Sugar crops	0	0	0	0	0
Vegetables	0	0	0	0	0
**New Zealand**					
Cereals	0	0	0	0	0
Fiber	0	0	0	0	0
Fruit	0	0	0	0	0
Livestock	0	0	0	0.01	0.05
Oil crops	0	0	0	0	0
Pulses	0	0	0	0	0
Roots & tubers	0	0	0	0	0
Sugar crops	0	0	0	0	0
Vegetables	0	0	0	0	0
**Nicaragua**					
Cereals	0	0	0	0	0
Fiber	0	0	0	0	0
Fruit	0	0	0	0	0

Livestock	0	0	0	0	0
Oil crops	0	0	0	0	0
Pulses	0	0	0	0	0
Roots & tubers	0	0	0	0	0
Sugar crops	0	0	0	0	0
Vegetables	0	0	0	0	0
**Niger**					
Cereals	0	0	0	0	0
Fiber	0	0	0	0	0
Fruit	0	0	0	0	0
Livestock	0	0	0	0	0
Oil crops	0	0	0	0	0
Pulses	0	0	0	0	0
Roots & tubers	0	0	0	0	0
Sugar crops	0	0	0	0	0
Vegetables	0	0	0	0	0
**Nigeria**					
Cereals	0	0	0	0	0
Fiber	0	0	0	0	0
Fruit	0	0	0	0	0
Livestock	0.01	0.01	0	0	0
Oil crops	0	0	0	0	0
Pulses	0	0	0	0	0
Roots & tubers	0	0	0	0	0
Sugar crops	0	0	0	0	0
Vegetables	0	0	0	0	0
**Norway**					
Cereals	0	0	0	0	0
Fiber	0	0	0	0	0
Fruit	0	0	0	0	0
Livestock	0	0	0	0	0
Oil crops	0	0	0	0	0
Pulses	0	0	0	0	0
Roots & tubers	0	0	0	0	0
Sugar crops	0	0	0	0	0
Vegetables	0	0	0	0	0
**Oman**					
Cereals	0	0	0	0	0
Fiber	0	0	0	0	0
Fruit	0	0	0	0	0
Livestock	0	0	0	0	0
Oil crops	0	0	0	0	0
Pulses	0	0	0	0	0
Roots & tubers	0	0	0	0	0
Sugar crops	0	0	0	0	0
Vegetables	0	0	0	0	0

**Pakistan**					
Cereals	0	0	0	0	0
Fiber	0	0	0	0	0
Fruit	0	0	0	0	0
Livestock	0.05	0.08	0.01	0	0
Oil crops	0	0	0	0	0
Pulses	0	0	0	0	0
Roots & tubers	0	0	0	0	0
Sugar crops	0	0	0	0	0
Vegetables	0	0	0	0	0
**Panama**					
Cereals	0	0	0	0	0
Fiber	0	0	0	0	0
Fruit	0	0	0	0	0
Livestock	0	0	0	0	0
Oil crops	0	0	0	0	0
Pulses	0	0	0	0	0
Roots & tubers	0	0	0	0	0
Sugar crops	0	0	0	0	0
Vegetables	0	0	0	0	0
**Papua New Guinea**					
Cereals	0	0	0	0	0
Fiber	0	0	0	0	0
Fruit	0	0	0	0	0
Livestock	0	0	0	0	0
Oil crops	0	0	0	0	0
Pulses	0	0	0	0	0
Roots & tubers	0	0	0	0	0
Sugar crops	0	0	0	0	0
Vegetables	0	0	0	0	0
**Paraguay**					
Cereals	0	0	0	0	0
Fiber	0	0	0	0	0
Fruit	0	0	0	0	0
Livestock	0	0	0	0	0
Oil crops	0	0	0	0	0
Pulses	0	0	0	0	0
Roots & tubers	0	0	0	0	0
Sugar crops	0	0	0	0	0
Vegetables	0	0	0	0	0
**Peru**					
Cereals	0	0	0	0	0
Fiber	0	0	0	0	0
Fruit	0	0	0	0	0
Livestock	0	0	0	0	0
Oil crops	0	0	0	0	0

Pulses	0	0	0	0	0
Roots & tubers	0	0	0	0	0
Sugar crops	0	0	0	0	0
Vegetables	0	0	0	0	0
**Philippines**					
Cereals	0	0	0	0	0
Fiber	0	0	0	0	0
Fruit	0	0	0	0	0
Livestock	0	0.01	0	0	0
Oil crops	0	0	0	0	0
Pulses	0	0	0	0	0
Roots & tubers	0	0	0	0	0
Sugar crops	0	0	0	0	0
Vegetables	0	0	0	0	0
**Poland**					
Cereals	0	0	0	0	0
Fiber	0	0	0	0	0
Fruit	0	0	0	0	0
Livestock	0.01	0.02	0.01	0.02	0.01
Oil crops	0	0	0	0	0
Pulses	0	0	0	0	0
Roots & tubers	0	0	0	0	0
Sugar crops	0	0	0	0	0
Vegetables	0	0	0	0	0
**Portugal**					
Cereals	0	0	0	0	0
Fiber	0	0	0	0	0
Fruit	0	0	0	0	0
Livestock	0	0	0	0	0
Oil crops	0	0	0	0	0
Pulses	0	0	0	0	0
Roots & tubers	0	0	0	0	0
Sugar crops	0	0	0	0	0
Vegetables	0	0	0	0	0
**Qatar**					
Cereals	0	0	0	0	0
Fiber	0	0	0	0	0
Fruit	0	0	0	0	0
Livestock	0	0	0	0	0
Oil crops	0	0	0	0	0
Pulses	0	0	0	0	0
Roots & tubers	0	0	0	0	0
Sugar crops	0	0	0	0	0
Vegetables	0	0	0	0	0
**Republic of Korea**					
Cereals	0	0	0	0	0

Fiber	0	0	0	0	0
Fruit	0	0	0	0	0
Livestock	0.01	0.01	0	0	0
Oil crops	0	0	0	0	0
Pulses	0	0	0	0	0
Roots & tubers	0	0	0	0	0
Sugar crops	0	0	0	0	0
Vegetables	0	0	0	0	0
**Romania**					
Cereals	0	0	0	0	0
Fiber	0	0	0	0	0
Fruit	0	0	0	0	0
Livestock	0	0.01	0	0.01	0
Oil crops	0	0	0	0	0
Pulses	0	0	0	0	0
Roots & tubers	0	0	0	0	0
Sugar crops	0	0	0	0	0
Vegetables	0	0	0	0	0
**Russian Federation**					
Cereals	0	0	0	0	0
Fiber	0	0	0	0	0
Fruit	0	0	0	0	0
Livestock	0.01	0.03	0.02	0.08	0.03
Oil crops	0	0	0	0	0
Pulses	0	0	0	0	0
Roots & tubers	0	0	0	0	0
Sugar crops	0	0	0	0	0
Vegetables	0	0	0	0	0
**Rwanda**					
Cereals	0	0	0	0	0
Fiber	0	0	0	0	0
Fruit	0	0	0	0	0
Livestock	0	0	0	0	0
Oil crops	0	0	0	0	0
Pulses	0	0	0	0	0
Roots & tubers	0	0	0	0	0
Sugar crops	0	0	0	0	0
Vegetables	0	0	0	0	0
**Saint Lucia**					
Cereals	0	0	0	0	0
Fiber	0	0	0	0	0
Fruit	0	0	0	0	0
Livestock	0	0	0	0	0
Oil crops	0	0	0	0	0
Pulses	0	0	0	0	0
Roots & tubers	0	0	0	0	0

Sugar crops	0	0	0	0	0
Vegetables	0	0	0	0	0
**Saint Vincent and the Grenadines**					
Cereals	0	0	0	0	0
Fiber	0	0	0	0	0
Fruit	0	0	0	0	0
Livestock	0	0	0	0	0
Oil crops	0	0	0	0	0
Pulses	0	0	0	0	0
Roots & tubers	0	0	0	0	0
Sugar crops	0	0	0	0	0
Vegetables	0	0	0	0	0
**Saudi Arabia**					
Cereals	0	0	0	0	0
Fiber	0	0	0	0	0
Fruit	0	0	0	0	0
Livestock	0	0.01	0	0	0
Oil crops	0	0	0	0	0
Pulses	0	0	0	0	0
Roots & tubers	0	0	0	0	0
Sugar crops	0	0	0	0	0
Vegetables	0	0	0	0	0
**Senegal**					
Cereals	0	0	0	0	0
Fiber	0	0	0	0	0
Fruit	0	0	0	0	0
Livestock	0	0	0	0	0
Oil crops	0	0	0	0	0
Pulses	0	0	0	0	0
Roots & tubers	0	0	0	0	0
Sugar crops	0	0	0	0	0
Vegetables	0	0	0	0	0
**Serbia**					
Cereals	0	0	0	0	0
Fiber	0	0	0	0	0
Fruit	0	0	0	0	0
Oil crops	0	0	0	0	0
Pulses	0	0	0	0	0
Roots & tubers	0	0	0	0	0
Sugar crops	0	0	0	0	0
Vegetables	0	0	0	0	0
**Sierra Leone**					
Cereals	0	0	0	0	0
Fiber	0	0	0	0	0
Fruit	0	0	0	0	0

Livestock	0	0	0	0	0
Oil crops	0	0	0	0	0
Pulses	0	0	0	0	0
Roots & tubers	0	0	0	0	0
Sugar crops	0	0	0	0	0
Vegetables	0	0	0	0	0
**Slovakia**					
Cereals	0	0	0	0	0
Fiber	0	0	0	0	0
Fruit	0	0	0	0	0
Livestock	0	0	0	0	0
Oil crops	0	0	0	0	0
Pulses	0	0	0	0	0
Roots & tubers	0	0	0	0	0
Sugar crops	0	0	0	0	0
Vegetables	0	0	0	0	0
**Slovenia**					
Cereals	0	0	0	0	0
Fiber	0	0	0	0	0
Fruit	0	0	0	0	0
Livestock	0	0	0	0	0
Oil crops	0	0	0	0	0
Pulses	0	0	0	0	0
Roots & tubers	0	0	0	0	0
Sugar crops	0	0	0	0	0
Vegetables	0	0	0	0	0
**Somalia**					
Cereals	0	0	0	0	0
Fiber	0	0	0	0	0
Fruit	0	0	0	0	0
Livestock	0	0	0	0	0
Oil crops	0	0	0	0	0
Pulses	0	0	0	0	0
Roots & tubers	0	0	0	0	0
Sugar crops	0	0	0	0	0
Vegetables	0	0	0	0	0
**South Africa**					
Cereals	0	0	0	0	0
Fiber	0	0	0	0	0
Fruit	0	0	0	0	0
Livestock	0.01	0.01	0	0.01	0
Oil crops	0	0	0	0	0
Pulses	0	0	0	0	0
Roots & tubers	0	0	0	0	0
Sugar crops	0	0	0	0	0
Vegetables	0	0	0	0	0

**Spain**					
Cereals	0	0	0	0	0
Fiber	0	0	0	0	0
Fruit	0	0	0	0	0
Livestock	0.01	0.02	0.01	0.02	0.01
Oil crops	0	0	0	0	0
Pulses	0	0	0	0	0
Roots & tubers	0	0	0	0	0
Sugar crops	0	0	0	0	0
Vegetables	0	0	0	0	0
**Sri Lanka**					
Cereals	0	0	0	0	0
Fiber	0	0	0	0	0
Fruit	0	0	0	0	0
Livestock	0	0	0	0	0
Oil crops	0	0	0	0	0
Pulses	0	0	0	0	0
Roots & tubers	0	0	0	0	0
Sugar crops	0	0	0	0	0
Vegetables	0	0	0	0	0
**Sudan (former)**					
Cereals	0	0	0	0	0
Fiber	0	0	0	0	0
Fruit	0	0	0	0	0
Livestock	0.01	0.02	0.01	0	0.01
Oil crops	0	0	0	0	0
Pulses	0	0	0	0	0
Roots & tubers	0	0	0	0	0
Sugar crops	0	0	0	0	0
Vegetables	0	0	0	0	0
**Suriname**					
Cereals	0	0	0	0	0
Fiber	0	0	0	0	0
Fruit	0	0	0	0	0
Livestock	0	0	0	0	0
Oil crops	0	0	0	0	0
Pulses	0	0	0	0	0
Roots & tubers	0	0	0	0	0
Sugar crops	0	0	0	0	0
Vegetables	0	0	0	0	0
**Swaziland**					
Cereals	0	0	0	0	0
Fiber	0	0	0	0	0
Fruit	0	0	0	0	0
Livestock	0	0	0	0	0
Oil crops	0	0	0	0	0

Pulses	0	0	0	0	0
Roots & tubers	0	0	0	0	0
Sugar crops	0	0	0	0	0
Vegetables	0	0	0	0	0
**Sweden**					
Cereals	0	0	0	0	0
Fiber	0	0	0	0	0
Fruit	0	0	0	0	0
Livestock	0	0	0	0.01	0
Oil crops	0	0	0	0	0
Pulses	0	0	0	0	0
Roots & tubers	0	0	0	0	0
Sugar crops	0	0	0	0	0
Vegetables	0	0	0	0	0
**Switzerland**					
Cereals	0	0	0	0	0
Fiber	0	0	0	0	0
Fruit	0	0	0	0	0
Livestock	0	0	0	0.01	0
Oil crops	0	0	0	0	0
Pulses	0	0	0	0	0
Roots & tubers	0	0	0	0	0
Sugar crops	0	0	0	0	0
Vegetables	0	0	0	0	0
**Syrian Arab Republic**					
Cereals	0	0	0	0	0
Fiber	0	0	0	0	0
Fruit	0	0	0	0	0
Livestock	0	0.01	0	0	0
Oil crops	0	0	0	0	0
Pulses	0	0	0	0	0
Roots & tubers	0	0	0	0	0
Sugar crops	0	0	0	0	0
Vegetables	0	0	0	0	0
**Tajikistan**					
Cereals	0	0	0	0	0
Fiber	0	0	0	0	0
Fruit	0	0	0	0	0
Livestock	0	0	0	0	0
Oil crops	0	0	0	0	0
Pulses	0	0	0	0	0
Roots & tubers	0	0	0	0	0
Sugar crops	0	0	0	0	0
Vegetables	0	0	0	0	0
**Thailand**					
Cereals	0	0	0	0	0

Fiber	0	0	0	0	0
Fruit	0	0	0	0	0
Livestock	0	0.01	0	0	0
Oil crops	0	0	0	0	0
Pulses	0	0	0	0	0
Roots & tubers	0	0	0	0	0
Sugar crops	0	0	0	0	0
Vegetables	0	0	0	0	0
**The former Yugoslav Republic of Macedonia**					
Cereals	0	0	0	0	0
Fiber	0	0	0	0	0
Fruit	0	0	0	0	0
Livestock	0	0	0	0	0
Oil crops	0	0	0	0	0
Pulses	0	0	0	0	0
Roots & tubers	0	0	0	0	0
Sugar crops	0	0	0	0	0
Vegetables	0	0	0	0	0
**Togo**					
Cereals	0	0	0	0	0
Fiber	0	0	0	0	0
Fruit	0	0	0	0	0
Livestock	0	0	0	0	0
Oil crops	0	0	0	0	0
Pulses	0	0	0	0	0
Roots & tubers	0	0	0	0	0
Sugar crops	0	0	0	0	0
Vegetables	0	0	0	0	0
**Trinidad and Tobago**					
Cereals	0	0	0	0	0
Fiber	0	0	0	0	0
Fruit	0	0	0	0	0
Livestock	0	0	0	0	0
Oil crops	0	0	0	0	0
Pulses	0	0	0	0	0
Roots & tubers	0	0	0	0	0
Sugar crops	0	0	0	0	0
Vegetables	0	0	0	0	0
**Tunisia**					
Cereals	0	0	0	0	0
Fiber	0	0	0	0	0
Fruit	0	0	0	0	0
Livestock	0	0	0	0	0
Oil crops	0	0	0	0	0
Pulses	0	0	0	0	0

Roots & tubers	0	0	0	0	0
Sugar crops	0	0	0	0	0
Vegetables	0	0	0	0	0
**Turkey**					
Cereals	0	0	0	0	0
Fiber	0	0	0	0	0
Fruit	0	0	0	0	0
Livestock	0.01	0.03	0.01	0.01	0
Oil crops	0	0	0	0	0
Pulses	0	0	0	0	0
Roots & tubers	0	0	0	0	0
Sugar crops	0	0	0	0	0
Vegetables	0	0	0	0	0
**Turkmenistan**					
Cereals	0	0	0	0	0
Fiber	0	0	0	0	0
Fruit	0	0	0	0	0
Livestock	0	0	0	0	0
Oil crops	0	0	0	0	0
Pulses	0	0	0	0	0
Roots & tubers	0	0	0	0	0
Sugar crops	0	0	0	0	0
Vegetables	0	0	0	0	0
**Uganda**					
Cereals	0	0	0	0	0
Fiber	0	0	0	0	0
Fruit	0	0	0	0	0
Livestock	0	0	0	0	0
Oil crops	0	0	0	0	0
Pulses	0	0	0	0	0
Roots & tubers	0	0	0	0	0
Sugar crops	0	0	0	0	0
Vegetables	0	0	0	0	0
**Ukraine**					
Cereals	0	0	0	0	0
Fiber	0	0	0	0	0
Fruit	0	0	0	0	0
Livestock	0.01	0.02	0.01	0.02	0.01
Oil crops	0	0	0	0	0
Pulses	0	0	0	0	0
Roots & tubers	0	0	0	0	0
Sugar crops	0	0	0	0	0
Vegetables	0	0	0	0	0
**United Arab Emirates**					
Cereals	0	0	0	0	0
Fiber	0	0	0	0	0

Fruit	0	0	0	0	0
Livestock	0	0	0	0	0
Oil crops	0	0	0	0	0
Pulses	0	0	0	0	0
Roots & tubers	0	0	0	0	0
Sugar crops	0	0	0	0	0
Vegetables	0	0	0	0	0
**United Kingdom**					
Cereals	0	0	0	0	0
Fiber	0	0	0	0	0
Fruit	0	0	0	0	0
Livestock	0	0.02	0.01	0.03	0.01
Oil crops	0	0	0	0	0
Pulses	0	0	0	0	0
Roots & tubers	0	0	0	0	0
Sugar crops	0	0	0	0	0
Vegetables	0	0	0	0	0
**United Republic of Tanzania**					
Cereals	0	0	0	0	0
Fiber	0	0	0	0	0
Fruit	0	0	0	0	0
Livestock	0	0.01	0	0	0
Oil crops	0	0	0	0	0
Pulses	0	0	0	0	0
Roots & tubers	0	0	0	0	0
Sugar crops	0	0	0	0	0
Vegetables	0	0	0	0	0
**United States of America**					
Cereals	0	0	0	0	0
Fiber	0	0	0	0	0
Fruit	0	0	0	0	0
Livestock	0	0.05	0.08	0.26	0.2
Oil crops	0	0	0	0	0
Pulses	0	0	0	0	0
Roots & tubers	0	0	0	0	0
Sugar crops	0	0	0	0	0
Vegetables	0	0	0	0	0
**Uruguay**					
Cereals	0	0	0	0	0
Fiber	0	0	0	0	0
Fruit	0	0	0	0	0
Livestock	0	0	0	0	0.01
Oil crops	0	0	0	0	0
Pulses	0	0	0	0	0
Roots & tubers	0	0	0	0	0
Sugar crops	0	0	0	0	0

Vegetables	0	0	0	0	0
**Uzbekistan**					
Cereals	0	0	0	0	0
Fiber	0	0	0	0	0
Fruit	0	0	0	0	0
Livestock	0	0.01	0.01	0	0
Oil crops	0	0	0	0	0
Pulses	0	0	0	0	0
Roots & tubers	0	0	0	0	0
Sugar crops	0	0	0	0	0
Vegetables	0	0	0	0	0
**Venezuela (Bolivarian Republic of)**					
Cereals	0	0	0	0	0
Fiber	0	0	0	0	0
Fruit	0	0	0	0	0
Livestock	0	0	0	0	0.01
Oil crops	0	0	0	0	0
Pulses	0	0	0	0	0
Roots & tubers	0	0	0	0	0
Sugar crops	0	0	0	0	0
Vegetables	0	0	0	0	0
**Viet Nam**					
Cereals	0	0	0	0	0
Fiber	0	0	0	0	0
Fruit	0	0	0	0	0
Livestock	0.01	0.01	0	0	0
Oil crops	0	0	0	0	0
Pulses	0	0	0	0	0
Roots & tubers	0	0	0	0	0
Sugar crops	0	0	0	0	0
Vegetables	0	0	0	0	0
**Yemen**					
Cereals	0	0	0	0	0
Fiber	0	0	0	0	0
Fruit	0	0	0	0	0
Livestock	0	0	0	0	0
Oil crops	0	0	0	0	0
Pulses	0	0	0	0	0
Roots & tubers	0	0	0	0	0
Sugar crops	0	0	0	0	0
Vegetables	0	0	0	0	0
**Zambia**					
Cereals	0	0	0	0	0
Fiber	0	0	0	0	0
Fruit	0	0	0	0	0

Livestock	0	0	0	0	0
Oil crops	0	0	0	0	0
Pulses	0	0	0	0	0
Roots & tubers	0	0	0	0	0
Sugar crops	0	0	0	0	0
Vegetables	0	0	0	0	0
**Zimbabwe**					
Cereals	0	0	0	0	0
Fiber	0	0	0	0	0
Fruit	0	0	0	0	0
Livestock	0	0	0	0	0
Oil crops	0	0	0	0	0
Pulses	0	0	0	0	0
Roots & tubers	0	0	0	0	0
Sugar crops	0	0	0	0	0
Vegetables	0	0	0	0	0

	**Zinc production (T)**				
	**< 2 ha**	**2 - 20 ha**	**20 - 50 ha**	**50 - 200 ha**	**> 200 ha**
**Afghanistan**					
Cereals	39.12	96.06	12.43	41.1	0
Fiber	0.05	1.61	0.29	0.05	0
Fruit	0.03	0.19	0.03	0.01	0
Livestock	6.32	8.17	0.75	0.13	0
Oil crops	1.2	1.52	0.13	0.02	0
Pulses	0.1	0.21	0.03	0.05	0
Roots & tubers	0.17	0.37	0.05	0.07	0
Sugar crops	0.01	0.01	0	0	0
Vegetables	0	0	0	0	0
**Albania**					
Cereals	2.55	7.98	1.99	1.95	0.09
Fiber	0	0.02	0.01	0.02	0
Fruit	0.01	0.05	0.02	0.02	0
Livestock	1.41	4.12	0.82	0.62	0.03
Oil crops	0.03	0.08	0.02	0.01	0
Pulses	0.12	0.36	0.07	0.06	0
Roots & tubers	0.06	0.18	0.05	0.06	0
Sugar crops	0.02	0.06	0.02	0.02	0
Vegetables	0.05	0.17	0.05	0.06	0
**Algeria**					
Cereals	15.67	58.43	17.34	19.89	26.97
Fiber	0	0	0	0	0
Fruit	0.33	1.43	0.5	0.26	0.09
Livestock	2.86	10.86	3.26	2.79	2.88
Oil crops	0.23	0.76	0.2	0.27	0.41
Pulses	0.03	0.09	0.03	0.03	0.05

Roots & tubers	0.6	1.95	0.51	0.52	0.65
Sugar crops	0	0	0	0	0
Vegetables	0.3	1.22	0.38	0.27	0.19
**Angola**					
Cereals	3.51	7.1	0.8	0.46	0
Fiber	0.06	0.13	0.02	0.02	0
Fruit	0.35	0.67	0.07	0.05	0
Livestock	1.82	2.82	0.24	0.22	0
Oil crops	0.21	0.39	0.04	0.03	0
Pulses	0.68	1.57	0.19	0.19	0
Roots & tubers	7.44	17.5	2.18	1.57	0
Sugar crops	0.01	0.03	0	0	0
Vegetables	0.01	0.01	0	0	0
**Argentina**					
Cereals	0.46	12.06	19.95	112.7	831.4
Fiber	0.08	1.34	1.46	3.98	21.37
Fruit	0	0.03	0.07	0.4	2.59
Livestock	0.59	10.28	11.48	28.64	105.26
Oil crops	0.84	23.11	39.29	237.55	1857.36
Pulses	0.01	0.25	0.41	1.62	6.62
Roots & tubers	0.01	0.29	0.4	1.24	3.44
Sugar crops	0.01	0.13	0.2	0.67	1.49
Vegetables	0	0.04	0.06	0.3	1.76
**Armenia**					
Cereals	0.96	7.08	2.92	1.54	0.27
Fiber	0	0	0	0	0
Fruit	0.01	0.1	0.04	0.02	0
Livestock	0.48	2.49	0.92	0.47	0.08
Oil crops	0	0	0	0	0
Pulses	0.01	0.07	0.03	0.01	0
Roots & tubers	0.09	0.68	0.28	0.15	0.03
Sugar crops	0.01	0.03	0.01	0.01	0
Vegetables	0.04	0.3	0.12	0.07	0.01
**Australia**					
Cereals	0	1.67	7.15	66.71	897.35
Fiber	0	0.09	0.4	3.92	69.35
Fruit	0	0	0.01	0.13	1.76
Livestock	0	0.34	1.36	12.39	129.94
Oil crops	0	0.13	0.55	4.94	50.91
Pulses	0	0.01	0.06	0.61	12.1
Roots & tubers	0	0.01	0.03	0.28	2.5
Sugar crops	0	0	0.03	0.27	3.41
Vegetables	0	0	0.01	0.08	0.87
**Austria**					
Cereals	11.09	44.35	20.08	36.17	4.34
Fiber	0	0	0	0	0

Fruit	0.05	0.17	0.06	0.1	0.01
Livestock	1.41	8.63	5.86	11.85	1.44
Oil crops	1.22	4.57	1.85	3.17	0.37
Pulses	0.27	1.07	0.49	0.88	0.11
Roots & tubers	0.19	0.66	0.24	0.38	0.04
Sugar crops	0.87	3.13	1.19	1.97	0.23
Vegetables	0.02	0.08	0.04	0.07	0.01
**Azerbaijan**					
Cereals	11.34	36.45	9.82	7.8	7.82
Fiber	1.32	4.25	1.14	0.91	0.92
Fruit	0.02	0.06	0.02	0.01	0.01
Livestock	1.4	4.57	1.23	1.16	1.39
Oil crops	0.06	0.2	0.06	0.04	0.04
Pulses	0.05	0.29	0.11	0.07	0.03
Roots & tubers	0.35	1.13	0.3	0.24	0.24
Sugar crops	0.05	0.16	0.04	0.03	0.03
Vegetables	0.16	0.51	0.14	0.09	0.06
**Bangladesh**					
Cereals	123.88	270.45	33.31	64.57	0
Fiber	0.79	1.77	0.22	0.24	0
Fruit	0.26	0.58	0.07	0.16	0
Livestock	8.13	15.11	1.69	2.63	0
Oil crops	2.98	8.53	1.17	2.01	0
Pulses	0.59	1.35	0.17	0.37	0
Roots & tubers	2.88	6.03	0.73	2.19	0
Sugar crops	0.15	0.31	0.04	0.09	0
Vegetables	0.34	0.74	0.09	0.17	0
**Belarus**					
Cereals	12.83	48.64	20.24	58.79	20.62
Fiber	0	0	0	0	0
Fruit	0	0.02	0.02	0.06	0.02
Livestock	2.6	10.71	5.01	15.44	5.52
Oil crops	0.7	2.65	1.11	3.17	1.09
Pulses	0.32	1.21	0.51	1.45	0.5
Roots & tubers	0.34	3.02	2.45	9.11	3.39
Sugar crops	0.16	1.49	1.24	4.84	1.85
Vegetables	0.05	0.2	0.09	0.23	0.07
**Belgium**					
Cereals	5.8	22.82	10.26	36.02	14.22
Fiber	0	0	0	0	0
Fruit	0.01	0.04	0.02	0.04	0.01
Livestock	3.74	14.14	5.72	14.07	4.26
Oil crops	0	0.09	0.1	0.92	0.5
Pulses	0.01	0.04	0.02	0.06	0.01
Roots & tubers	0.82	2.73	0.87	1.49	0.3
Sugar crops	0.95	3.99	1.95	6.38	2.35

Vegetables	0.04	0.14	0.06	0.15	0.05
**Belize**					
Cereals	0.02	0.14	0.36	0.16	0.07
Fiber	0	0	0	0	0
Fruit	0	0.04	0.11	0.05	0.02
Livestock	0.02	0.04	0.06	0.03	0.03
Oil crops	0	0.01	0.01	0.01	0
Pulses	0	0.02	0.06	0.03	0.01
Roots & tubers	0	0	0	0	0
Sugar crops	0	0.02	0.05	0.02	0.01
Vegetables	0	0	0	0	0
**Benin**					
Cereals	5.31	8.65	0.82	0	0
Fiber	3.37	10.73	1.54	0.01	0
Fruit	0.03	0.06	0.01	0	0
Livestock	0.49	0.84	0.08	0	0
Oil crops	0.51	0.73	0.06	0	0
Pulses	1.05	1.81	0.18	0	0
Roots & tubers	5.36	6.72	0.43	0	0
Sugar crops	0	0	0	0	0
Vegetables	0.08	0.13	0.01	0	0
**Bhutan**					
Cereals	1.03	1.5	0.14	0.03	0
Fiber	0	0	0	0	0
Fruit	0.01	0.01	0	0	0
Livestock	0.14	0.2	0.02	0	0
Oil crops	0.03	0.04	0	0	0
Pulses	0.09	0.11	0.01	0	0
Roots & tubers	0.05	0.07	0.01	0	0
Sugar crops	0	0	0	0	0
Vegetables	0	0	0	0	0
**Bolivia (Plurinational State of)**					
Cereals	0.15	2.44	2.56	5.48	18.25
Fiber	0.02	0.32	0.36	0.99	4.79
Fruit	0.01	0.15	0.13	0.16	0.21
Livestock	0.1	1.63	1.65	2.94	6.53
Oil crops	0.07	1.57	2.43	11.29	68.01
Pulses	0.01	0.15	0.14	0.23	0.62
Roots & tubers	0.04	0.54	0.5	0.66	0.95
Sugar crops	0	0.02	0.02	0.09	0.46
Vegetables	0	0.02	0.02	0.04	0.16
**Bosnia and Herzegovina**					
Cereals	2.64	10.05	4.32	7.93	1.02
Fiber	0	0	0	0	0
Fruit	0	0.01	0.01	0.01	0
Livestock	0.41	1.59	0.68	1.17	0.14

Oil crops	0.05	0.23	0.12	0.23	0.03
Pulses	0.05	0.2	0.09	0.16	0.02
Roots & tubers	0.09	0.36	0.15	0.27	0.04
Sugar crops	0	0	0	0	0
Vegetables	0.01	0.04	0.02	0.03	0
**Botswana**					
Cereals	0.07	0.33	0.05	0.17	0
Fiber	0.02	0.09	0.01	0.02	0
Fruit	0	0	0	0	0
Livestock	0.16	0.91	0.15	0.63	0
Oil crops	0.02	0.07	0.01	0.04	0
Pulses	0.01	0.03	0.01	0.03	0
Roots & tubers	0	0	0	0	0
Sugar crops	0	0	0	0	0
Vegetables	0	0	0	0	0
**Brazil**					
Cereals	1.91	33.93	39.2	125.87	739.98
Fiber	0.05	1.76	3.21	22.11	186.43
Fruit	0.15	2.36	2.23	3.54	10.42
Livestock	2.98	48.11	47.87	91.78	325.5
Oil crops	0.86	22.73	37.63	259.6	2334.33
Pulses	0.81	12.67	11.78	17.08	43
Roots & tubers	0.74	11.75	11.27	17.84	42.42
Sugar crops	0.02	0.68	1.17	6.23	40.23
Vegetables	0.04	0.71	0.71	1.36	4.42
**Brunei Darussalam**					
Cereals	0	0.01	0	0	0
Fiber	0	0	0	0	0
Fruit	0	0	0	0	0
Livestock	0	0.2	0.03	0.01	0
Oil crops	0	0	0	0	0
Pulses	0	0	0	0	0
Roots & tubers	0	0.01	0	0	0
Sugar crops	0	0	0	0	0
Vegetables	0	0	0	0	0
**Bulgaria**					
Cereals	5.71	32.47	21.24	74.27	26.84
Fiber	0.02	0.04	0.01	0	0
Fruit	0.02	0.08	0.03	0.07	0.02
Livestock	0.89	3.48	1.48	3.51	0.98
Oil crops	0.43	3.78	3.11	13.64	5.65
Pulses	0.02	0.07	0.04	0.12	0.04
Roots & tubers	0.06	0.24	0.13	0.29	0.05
Sugar crops	0	0.01	0.01	0.03	0.01
Vegetables	0.02	0.07	0.03	0.11	0.04
**Burkina Faso**					

Cereals	14.59	29.15	3.24	8.56	0
Fiber	9.72	18.01	1.96	7.31	0
Fruit	0	0	0	0	0
Livestock	2.74	3.91	0.31	0.74	0
Oil crops	0.25	0.79	0.11	0.87	0
Pulses	0.08	0.12	0.01	0.03	0
Roots & tubers	0.05	0.1	0.01	0.05	0
Sugar crops	0	0.02	0	0.02	0
Vegetables	0	0.01	0	0	0
**Burundi**					
Cereals	2.04	2.46	0.13	0	0
Fiber	0.13	0.16	0.01	0	0
Fruit	0.7	0.84	0.04	0	0
Livestock	0.32	0.39	0.02	0	0
Oil crops	0.06	0.07	0	0	0
Pulses	2.96	3.57	0.18	0	0
Roots & tubers	1.6	1.93	0.1	0	0
Sugar crops	0.01	0.01	0	0	0
Vegetables	0	0	0	0	0
**Cambodia**					
Cereals	21.04	48.58	3.33	1.36	0
Fiber	0.01	0.01	0	0	0
Fruit	0.05	0.11	0.01	0	0
Livestock	1.66	2.95	0.14	0.05	0
Oil crops	3.89	6.54	0.34	0.13	0
Pulses	0.5	0.85	0.04	0.01	0
Roots & tubers	1.64	2.93	0.15	0.06	0
Sugar crops	0.01	0.01	0	0	0
Vegetables	0	0	0	0	0
**Cameroon**					
Cereals	11.91	18.12	1.54	1.23	0
Fiber	6.05	10.06	0.96	0.33	0
Fruit	1.03	1.6	0.14	0.21	0
Livestock	2.66	3.26	0.22	0	0
Oil crops	0.37	0.59	0.05	0.02	0
Pulses	2.88	3.49	0.2	0	0
Roots & tubers	2.47	4.4	0.44	1.64	0
Sugar crops	0.04	0.06	0	0.01	0
Vegetables	0.36	0.53	0.04	0.02	0
**Canada**					
Cereals	0.54	124.17	195.25	614.35	653.89
Fiber	0	0	0	0	0
Fruit	0	0.01	0.03	0.09	0.05
Livestock	0.05	13.17	21.03	51.47	31.88
Oil crops	0.25	55.75	85.41	237.85	233.68
Pulses	0.04	8.63	13.28	41.23	45.33

Roots & tubers	0.01	1.24	2.03	4.52	2.74
Sugar crops	0	0.06	0.09	0.69	1.17
Vegetables	0	0.13	0.26	0.76	0.44
**Central African Republic**					
Cereals	1.36	1.82	0.12	0	0
Fiber	0.08	0.09	0.01	0	0
Fruit	0.07	0.12	0.01	0	0
Oil crops	1.31	1.77	0.13	0	0
Pulses	0.09	0.16	0.02	0	0
Roots & tubers	1.04	1.31	0.08	0	0
Sugar crops	0	0.01	0	0	0
Vegetables	0	0	0	0	0
**Chad**					
Cereals	9.95	17.63	1.8	8.02	0
Fiber	2.61	4.89	0.52	0.84	0
Fruit	0.01	0.03	0	0.02	0
Livestock	1.69	2.56	0.23	0.49	0
Oil crops	0.92	1.46	0.13	0.33	0
Pulses	0.75	1.33	0.13	0.43	0
Roots & tubers	0.45	0.86	0.09	0.25	0
Sugar crops	0.01	0.02	0	0	0
Vegetables	0.01	0.02	0	0.01	0
**Chile**					
Cereals	0.54	8.8	9.03	18.36	61.85
Fiber	0	0	0	0	0
Fruit	0.01	0.19	0.19	0.38	1.33
Livestock	0.17	2.83	2.97	6.15	17.92
Oil crops	0.01	0.21	0.21	0.42	1.47
Pulses	0.01	0.13	0.12	0.19	0.63
Roots & tubers	0.01	0.16	0.18	0.43	1.66
Sugar crops	0.03	0.53	0.53	1.04	3.65
Vegetables	0.01	0.21	0.21	0.41	1.44
**China**					
Cereals	5090.79	2372.95	0	1181.91	0
Fiber	531.09	382.61	0	291.34	0
Fruit	14.46	7.12	0	2.4	0
Livestock	972.53	395.74	0	169.7	0
Oil crops	701.88	432.93	0	249.09	0
Pulses	45.25	21.97	0	15.58	0
Roots & tubers	240.91	71.92	0	23.95	0
Sugar crops	12.48	12.77	0	6.42	0
Vegetables	49.12	23.01	0	9.76	0
**Colombia**					
Cereals	0.11	2.19	2.9	9.38	35.72
Fiber	0.06	0.95	0.92	1.54	3.99
Fruit	0.02	0.42	0.45	1.02	3.11

Livestock	0.48	7.68	7.53	13.13	37.37
Oil crops	0.02	0.27	0.27	0.56	2.54
Pulses	0.03	0.54	0.59	1.27	2.87
Roots & tubers	0.08	1.22	1.21	2.15	5.74
Sugar crops	0.03	0.46	0.46	0.83	1.98
Vegetables	0.01	0.16	0.15	0.26	0.66
**Congo**					
Cereals	0.11	0.17	0.01	0	0
Fiber	0	0	0	0	0
Fruit	0.03	0.09	0.01	0.01	0
Livestock	0.07	0.14	0.02	0.06	0
Oil crops	0.01	0.02	0	0	0
Pulses	0.09	0.16	0.02	0.03	0
Roots & tubers	1	1.85	0.19	0.07	0
Sugar crops	0.02	0.03	0	0	0
Vegetables	0	0	0	0	0
**Costa Rica**					
Cereals	0	0.16	0.59	0.55	0.94
Fiber	0	0	0.01	0.01	0.02
Fruit	0	0.24	0.9	0.84	1.43
Livestock	0.02	0.51	1.73	1.75	3.54
Oil crops	0	0.01	0.04	0.04	0.07
Pulses	0	0.02	0.07	0.06	0.11
Roots & tubers	0	0.03	0.13	0.12	0.2
Sugar crops	0	0.03	0.12	0.11	0.18
Vegetables	0	0.01	0.03	0.03	0.06
**Croatia**					
Cereals	5.62	20.35	7.98	22.87	7.94
Fiber	0	0	0	0	0
Fruit	0.02	0.05	0.02	0.05	0.02
Livestock	0.48	1.86	0.81	2.87	1.16
Oil crops	0.95	3.3	1.19	3.3	1.15
Pulses	0.01	0.04	0.02	0.05	0.02
Roots & tubers	0.06	0.21	0.07	0.2	0.07
Sugar crops	0.35	1.27	0.49	1.41	0.5
Vegetables	0.01	0.03	0.01	0.03	0.01
**Cuba**					
Cereals	0.99	2.25	2.15	1.56	2.44
Fiber	0	0	0	0	0
Fruit	0.1	0.23	0.23	0.18	0.3
Livestock	0.38	1.15	1.82	1.4	2.38
Oil crops	0.07	0.16	0.15	0.11	0.17
Pulses	0.26	0.58	0.55	0.4	0.63
Roots & tubers	0.33	0.75	0.72	0.52	0.82
Sugar crops	0.12	0.28	0.26	0.19	0.3
Vegetables	0.13	0.3	0.28	0.21	0.32

**Cyprus**					
Cereals	0	0	0	0	0
Fiber	0	0	0	0	0
Fruit	0	0	0	0	0
Livestock	0.25	1.14	0.42	0.2	0.04
Oil crops	0	0	0	0	0
Pulses	0	0	0	0	0
Roots & tubers	0	0	0	0	0
Sugar crops	0	0	0	0	0
Vegetables	0	0	0	0	0
**Czech Republic**					
Cereals	18.47	68.89	29	88.61	31.24
Fiber	0	0	0	0	0
Fruit	0.01	0.04	0.01	0.03	0.01
Livestock	1.09	5.29	3.06	9.72	3.27
Oil crops	3.37	12.95	5.74	18.19	6.49
Pulses	0.25	0.87	0.32	0.83	0.27
Roots & tubers	0.09	0.43	0.27	0.81	0.23
Sugar crops	0.82	2.8	0.98	2.87	1.08
Vegetables	0.01	0.04	0.02	0.04	0.01
**Democratic Republic of the Congo**					
Cereals	6.31	10.72	1.04	2.67	0
Fiber	0.53	0.99	0.1	0.29	0
Fruit	0.5	0.81	0.07	0.23	0
Livestock	0.53	1	0.1	0.09	0
Oil crops	0.36	0.61	0.06	0.11	0
Pulses	0.97	1.59	0.15	0.37	0
Roots & tubers	12.19	22.91	2.42	6.17	0
Sugar crops	0.04	0.08	0.01	0.03	0
Vegetables	0.04	0.08	0.01	0.03	0
**Denmark**					
Cereals	6.81	56.68	43.87	147.66	51.33
Fiber	0	0	0	0	0
Fruit	0	0	0	0.01	0
Livestock	1.26	10.22	7.79	24.95	8.31
Oil crops	0.5	4.14	3.2	10.71	3.7
Pulses	0.02	0.2	0.15	0.51	0.18
Roots & tubers	0.07	0.61	0.47	1.6	0.56
Sugar crops	0.15	1.22	0.94	3.17	1.1
Vegetables	0	0.02	0.02	0.05	0.02
**Djibouti**					
Cereals	0	0	0	0	0
Fiber	0	0	0	0	0
Fruit	0	0	0	0	0
Livestock	0	0	0	0	0

Oil crops	0	0	0	0	0
Pulses	0	0	0	0	0
Roots & tubers	0	0	0	0	0
Sugar crops	0	0	0	0	0
Vegetables	0	0	0	0	0
**Dominica**					
Cereals	0	0	0	0	0
Fiber	0	0	0	0	0
Fruit	0	0	0.01	0.01	0.01
Livestock	0	0.01	0.02	0.01	0.01
Oil crops	0	0.01	0.02	0.01	0.02
Pulses	0	0	0	0	0
Roots & tubers	0	0	0.01	0.01	0.01
Sugar crops	0	0	0	0	0
Vegetables	0	0	0	0	0
**Ecuador**					
Cereals	0.33	5.11	4.85	7.08	12.5
Fiber	0	0.03	0.03	0.05	0.1
Fruit	0.1	1.49	1.31	1.45	2.17
Livestock	0.29	4.55	4.35	6.76	15.47
Oil crops	0	0.1	0.14	0.5	1.61
Pulses	0.01	0.09	0.09	0.12	0.22
Roots & tubers	0.01	0.11	0.11	0.23	0.52
Sugar crops	0.01	0.09	0.09	0.16	0.4
Vegetables	0	0.02	0.02	0.03	0.04
**Egypt**					
Cereals	60.11	278.04	103.13	50.03	8.87
Fiber	4.25	19.68	7.3	3.54	0.63
Fruit	0.99	4.57	1.69	0.82	0.15
Livestock	6.45	29.84	11.07	5.37	0.95
Oil crops	0.77	3.58	1.33	0.64	0.11
Pulses	0.18	0.85	0.31	0.15	0.03
Roots & tubers	0.81	3.75	1.39	0.67	0.12
Sugar crops	1.58	7.31	2.71	1.32	0.23
Vegetables	1.78	8.22	3.05	1.48	0.26
**El Salvador**					
Cereals	1.63	4.06	4.47	1.89	0.82
Fiber	0.01	0.03	0.03	0.01	0.01
Fruit	0.02	0.04	0.03	0.01	0.01
Livestock	0.67	1.52	1.36	0.57	0.25
Oil crops	0.12	0.26	0.2	0.09	0.04
Pulses	0.1	0.45	0.9	0.38	0.17
Roots & tubers	0.01	0.03	0.02	0.01	0
Sugar crops	0.1	0.2	0.11	0.05	0.02
Vegetables	0.01	0.02	0.01	0.01	0
**Equatorial Guinea**					

Cereals	0	0	0	0	0
Fiber	0	0	0	0	0
Fruit	0.03	0.03	0	0	0
Livestock	0.01	0.01	0	0	0
Oil crops	0.02	0.02	0	0	0
Pulses	0	0	0	0	0
Roots & tubers	0.14	0.17	0.01	0	0
Sugar crops	0	0	0	0	0
Vegetables	0	0	0	0	0
**Eritrea**					
Cereals	2.43	4.93	0.55	0	0
Fiber	0	0	0	0	0
Fruit	0	0	0	0	0
Livestock	0.52	0.92	0.09	0	0
Oil crops	0.24	0.59	0.08	0	0
Pulses	0.07	0.18	0.02	0	0
Roots & tubers	0.01	0.01	0	0	0
Sugar crops	0	0	0	0	0
Vegetables	0	0	0	0	0
**Estonia**					
Cereals	0.32	2.95	2.42	13.1	6.18
Fiber	0	0	0	0	0
Fruit	0	0	0	0	0
Livestock	0.03	0.48	0.48	2.12	0.85
Oil crops	0.06	0.54	0.45	2.6	1.26
Pulses	0	0.03	0.02	0.13	0.06
Roots & tubers	0	0.05	0.04	0.21	0.1
Sugar crops	0	0	0	0	0
Vegetables	0	0	0	0.01	0
**Ethiopia**					
Cereals	95.46	178.59	18.85	19.61	0
Fiber	1.48	2.79	0.3	0.5	0
Fruit	0.07	0.14	0.01	0.02	0
Livestock	9.34	15.34	1.43	0.43	0
Oil crops	3.86	6.75	0.67	1.04	0
Pulses	4.42	6.71	0.56	0.68	0
Roots & tubers	0.72	1.35	0.14	0.24	0
Sugar crops	0.07	0.14	0.01	0.03	0
Vegetables	0.1	0.19	0.02	0.03	0
**Finland**					
Cereals	3.91	30.69	23.88	60.92	13.66
Fiber	0	0	0	0	0
Fruit	0	0	0	0	0
Livestock	0.73	4.45	3.01	7.05	1.41
Oil crops	0.18	1.39	1.07	2.73	0.62
Pulses	0.01	0.08	0.06	0.15	0.03

Roots & tubers	0.04	0.33	0.26	0.68	0.16
Sugar crops	0.08	0.58	0.45	1.15	0.26
Vegetables	0	0.02	0.02	0.04	0.01
**France**					
Cereals	65.18	349.52	226.26	934.28	378.35
Fiber	0	0	0	0	0
Fruit	0.29	1.5	0.85	2.02	0.51
Livestock	15.83	65.5	31.91	74.09	16.7
Oil crops	9.13	44.23	26.43	125.95	56.26
Pulses	1.93	8.64	4.62	14.79	5.11
Roots & tubers	0.14	1.93	1.92	7.77	2.84
Sugar crops	0.41	7.07	7.57	47.21	22.4
Vegetables	0.08	0.35	0.19	0.59	0.2
**Gabon**					
Cereals	0.2	0.26	0.02	0	0
Fiber	0	0	0	0	0
Fruit	0.11	0.14	0.01	0	0
Livestock	0.05	0.13	0.02	0	0
Oil crops	0.04	0.06	0	0	0
Pulses	0	0	0	0	0
Roots & tubers	0.42	0.57	0.04	0	0
Sugar crops	0.01	0.01	0	0	0
Vegetables	0	0	0	0	0
**Gambia**					
Cereals	0.98	1.83	0.19	0	0
Fiber	0.01	0.02	0	0	0
Fruit	0	0	0	0	0
Livestock	0.06	0.15	0.02	0	0
Oil crops	0.07	0.12	0.01	0	0
Pulses	0.01	0.02	0	0	0
Roots & tubers	0.01	0.01	0	0	0
Sugar crops	0	0	0	0	0
Vegetables	0	0	0	0	0
**Georgia**					
Cereals	1.1	5.8	2.15	1.11	0.19
Fiber	0	0	0	0	0
Fruit	0.01	0.1	0.04	0.02	0
Livestock	0.57	2.79	1	0.51	0.09
Oil crops	0.08	0.41	0.15	0.08	0.01
Pulses	0.04	0.21	0.08	0.04	0.01
Roots & tubers	0.06	0.34	0.13	0.07	0.01
Sugar crops	0	0	0	0	0
Vegetables	0.02	0.11	0.04	0.02	0
**Germany**					
Cereals	56.14	297.91	183.02	631.38	230.65
Fiber	0	0	0	0	0

Fruit	0.18	0.58	0.16	0.27	0.07
Livestock	8.75	47.51	29.6	110.21	42.93
Oil crops	9.11	48.11	30.09	121.22	49.19
Pulses	0.51	2.31	1.22	4.11	1.52
Roots & tubers	0.77	4.59	3.05	11.36	4.38
Sugar crops	1.74	13.83	10.36	29.38	8.54
Vegetables	0.04	0.17	0.08	0.26	0.09
**Ghana**					
Cereals	10.43	14.71	1.11	0	0
Fiber	0.41	0.74	0.08	0	0
Fruit	1.23	1.69	0.13	0	0
Livestock	1	1.42	0.11	0	0
Oil crops	0.6	1.09	0.11	0	0
Pulses	1.76	2.12	0.11	0	0
Roots & tubers	14.82	20.53	1.54	0	0
Sugar crops	0.01	0.01	0	0	0
Vegetables	0.13	0.21	0.02	0	0
**Greece**					
Cereals	12.44	49.56	21.5	35.58	3.68
Fiber	6.47	25.8	11.5	20.16	2.32
Fruit	0.14	0.53	0.22	0.35	0.03
Livestock	2.24	8.75	3.53	5.13	0.4
Oil crops	0.57	2.19	0.88	1.36	0.13
Pulses	0.07	0.25	0.09	0.14	0.01
Roots & tubers	0.18	0.75	0.35	0.6	0.07
Sugar crops	0.52	2	0.82	1.33	0.14
Vegetables	0.31	1.16	0.45	0.72	0.07
**Grenada**					
Cereals	0	0	0	0	0
Fiber	0	0	0	0	0
Fruit	0	0	0	0	0
Livestock	0	0	0	0	0
Oil crops	0	0	0	0	0
Pulses	0	0	0	0	0
Roots & tubers	0	0	0	0	0
Sugar crops	0	0	0	0	0
Vegetables	0	0	0	0	0
**Guatemala**					
Cereals	1.05	3.29	5.6	3.83	4.84
Fiber	0.01	0.05	0.09	0.05	0.05
Fruit	0.1	0.38	0.72	0.42	0.42
Livestock	0.71	1.97	2.71	1.47	1.36
Oil crops	0.25	0.91	1.71	0.98	1
Pulses	0.22	0.68	1.12	0.72	0.84
Roots & tubers	0.05	0.19	0.35	0.2	0.21
Sugar crops	0.09	0.35	0.66	0.38	0.39

Vegetables	0.03	0.12	0.23	0.13	0.14
**Guinea**					
Cereals	8.59	17.23	1.94	0.01	0
Fiber	0.8	1.63	0.19	0	0
Fruit	0.19	0.4	0.05	0	0
Livestock	0.89	1.51	0.15	0	0
Oil crops	0.09	0.16	0.02	0	0
Pulses	0.12	0.42	0.06	0	0
Roots & tubers	1.17	2.16	0.23	0	0
Sugar crops	0.01	0.02	0	0	0
Vegetables	0	0	0	0	0
**Guinea-Bissau**					
Cereals	1.2	1.45	0.08	0	0
Fiber	0.14	0.17	0.01	0	0
Fruit	0.02	0.03	0	0	0
Livestock	0.23	0.28	0.02	0	0
Oil crops	0.11	0.14	0.01	0	0
Pulses	0.01	0.01	0	0	0
Roots & tubers	0.05	0.06	0	0	0
Sugar crops	0	0	0	0	0
Vegetables	0	0	0	0	0
**Guyana**					
Cereals	0	0.05	0.11	0.67	4.14
Fiber	0	0	0	0	0
Fruit	0	0	0	0	0.02
Livestock	0	0.01	0.02	0.08	0.3
Oil crops	0	0.02	0.03	0.1	0.32
Pulses	0	0	0	0	0.01
Roots & tubers	0	0	0	0.01	0.05
Sugar crops	0	0.01	0.01	0.05	0.23
Vegetables	0	0	0	0	0
**Haiti**					
Cereals	0.96	2.16	1.91	0.81	0.35
Fiber	0.01	0.03	0.03	0.01	0
Fruit	0.08	0.19	0.17	0.07	0.03
Livestock	0.44	0.95	0.73	0.31	0.14
Oil crops	0.08	0.17	0.15	0.07	0.03
Pulses	0.2	0.46	0.41	0.17	0.08
Roots & tubers	0.33	0.74	0.66	0.28	0.12
Sugar crops	0.02	0.04	0.03	0.01	0.01
Vegetables	0	0.01	0	0	0
**Honduras**					
Cereals	0.88	1.78	1.18	1.45	3.71
Fiber	0.01	0.02	0.02	0.02	0.05
Fruit	0.09	0.2	0.15	0.16	0.39
Livestock	0.78	1.6	1.1	1.01	2.12

Oil crops	0.02	0.05	0.04	0.04	0.1
Pulses	0.1	0.23	0.2	0.35	1.02
Roots & tubers	0.01	0.03	0.02	0.02	0.04
Sugar crops	0.05	0.1	0.08	0.1	0.24
Vegetables	0.03	0.06	0.04	0.04	0.11
**Hungary**					
Cereals	25.78	101.4	43.52	116.53	38.18
Fiber	0	0	0	0	0
Fruit	0.03	0.14	0.07	0.19	0.07
Livestock	1.1	5.91	3.46	8.23	2.04
Oil crops	3.14	13.7	6.83	20.77	7.28
Pulses	0.1	0.5	0.27	0.73	0.23
Roots & tubers	0.12	0.45	0.17	0.42	0.13
Sugar crops	0.72	2.57	0.96	2.09	0.55
Vegetables	0.05	0.18	0.05	0.13	0.04
**Iceland**					
Cereals	0	0	0	0	0
Fiber	0	0	0	0	0
Fruit	0	0	0	0	0
Livestock	0	0	0	0	0
Oil crops	0	0	0	0	0
Pulses	0	0	0	0	0
Roots & tubers	0	0	0	0	0
Sugar crops	0	0	0	0	0
Vegetables	0	0	0	0	0
**India**					
Cereals	1684.6	2985.99	334.9	63.11	0
Fiber	160.42	473.8	65.45	12.37	0
Fruit	6.89	15.61	1.95	0.37	0
Livestock	152.18	337.98	41.9	8.05	0
Oil crops	278.38	613.12	75.79	14.53	0
Pulses	31.88	77.63	9.98	1.92	0
Roots & tubers	29.05	51.63	5.79	1.09	0
Sugar crops	7.15	18.96	2.54	0.48	0
Vegetables	11.36	18.56	1.94	0.38	0
**Indonesia**					
Cereals	269.75	376.72	13.92	116.69	0
Fiber	0.09	0.16	0.01	0.08	0
Fruit	1.87	3.29	0.18	1.7	0
Livestock	20.02	29.58	1.2	2.44	0
Oil crops	42.99	67.03	3.01	26.85	0
Pulses	2.25	3.97	0.21	2.05	0
Roots & tubers	23.74	33.9	1.24	3.8	0
Sugar crops	0.74	1.3	0.07	0.68	0
Vegetables	0.58	1.02	0.05	0.53	0
**Iran (Islamic Republic of)**					

Cereals	191.46	378.04	43.72	150.89	0
Fiber	6.13	10.12	1.05	1.9	0
Fruit	1.98	4.49	0.55	1.39	0
Livestock	12.48	39.05	5.42	15.42	0
Oil crops	2.93	20.16	3.29	2.9	0
Pulses	1.43	3.2	0.4	1.16	0
Roots & tubers	2.02	4.07	0.47	2.92	0
Sugar crops	1.85	8.26	1.25	4.46	0
Vegetables	2.46	5.31	0.64	2.44	0
**Iraq**					
Cereals	21.46	68.46	17.93	10.01	4.25
Fiber	0.2	0.92	0.34	0.17	0.03
Fruit	0.24	1.03	0.35	0.19	0.07
Livestock	0.51	2.31	0.79	0.68	0.71
Oil crops	0.07	0.29	0.1	0.05	0.01
Pulses	0.03	0.09	0.03	0.01	0.01
Roots & tubers	0.15	0.8	0.29	0.2	0.15
Sugar crops	0	0.02	0.01	0	0
Vegetables	0.2	0.95	0.36	0.17	0.03
**Ireland**					
Cereals	2.19	14.25	10.15	31.53	9.88
Fiber	0	0	0	0	0
Fruit	0	0	0	0.01	0
Livestock	3.44	14.83	7.3	16.38	3.75
Oil crops	0.03	0.22	0.15	0.48	0.15
Pulses	0.02	0.11	0.07	0.23	0.07
Roots & tubers	0.03	0.18	0.13	0.4	0.13
Sugar crops	0.07	0.68	0.58	1.85	0.57
Vegetables	0	0.01	0	0.01	0
**Israel**					
Cereals	1.61	9.59	3.79	1.94	0.34
Fiber	0.64	3.34	1.27	0.64	0.11
Fruit	0.13	0.56	0.19	0.09	0.02
Livestock	3.65	14.99	5.03	2.48	0.43
Oil crops	0.12	0.55	0.2	0.1	0.02
Pulses	0	0.02	0.01	0	0
Roots & tubers	0.32	1.49	0.54	0.27	0.05
Sugar crops	0	0	0	0	0
Vegetables	0.2	0.91	0.33	0.16	0.03
**Italy**					
Cereals	18.06	114	78.83	234.41	70.99
Fiber	0	0	0	0	0
Fruit	0.26	1.54	1.02	3.41	1.21
Livestock	5.55	35.9	24.61	49.82	6.59
Oil crops	1.53	9.74	6.67	17.07	4.2
Pulses	0.09	0.47	0.29	0.74	0.19

Roots & tubers	0.26	1.11	0.56	1.51	0.45
Sugar crops	0.71	5.29	3.94	9.36	1.89
Vegetables	0.52	2.84	1.76	4.59	1.21
**Ivory Coast**					
Cereals	0	0	0	0	0
Fiber	0	0	0	0	0
Fruit	0	0	0	0	0
Livestock	0.33	1.41	0.22	0.1	0
Oil crops	0	0	0	0	0
Pulses	0	0	0	0	0
Roots & tubers	0	0	0	0	0
Sugar crops	0	0	0	0	0
Vegetables	0	0	0	0	0
**Jamaica**					
Cereals	0.01	0.01	0	0	0
Fiber	0	0	0	0	0
Fruit	0.06	0.1	0.01	0	0
Livestock	0.62	1.06	0.16	0.07	0.03
Oil crops	0.58	0.98	0.08	0.03	0.02
Pulses	0.01	0.02	0	0	0
Roots & tubers	0.12	0.21	0.02	0.01	0
Sugar crops	0.06	0.1	0.01	0	0
Vegetables	0.01	0.02	0	0	0
**Japan**					
Cereals	50.49	66.9	26.71	17.07	0
Fiber	0	0	0	0	0
Fruit	0.14	0.2	0.08	0.06	0
Livestock	43.06	36.02	11.31	14.65	0
Oil crops	3.45	4.48	1.78	1.44	0
Pulses	0.71	1.01	0.42	0.32	0
Roots & tubers	3.2	3.3	1.19	0.87	0
Sugar crops	3.32	4.63	1.88	1.46	0
Vegetables	0.85	1.21	0.5	0.38	0
**Jordan**					
Cereals	0.24	1.12	0.42	0.2	0.04
Fiber	0	0	0	0	0
Fruit	0.01	0.06	0.02	0.01	0
Livestock	0.36	1.67	0.62	0.3	0.05
Oil crops	0.03	0.13	0.05	0.02	0
Pulses	0	0	0	0	0
Roots & tubers	0.04	0.17	0.06	0.03	0.01
Sugar crops	0	0	0	0	0
Vegetables	0.11	0.49	0.18	0.09	0.02
**Kazakhstan**					
Cereals	8.06	153.26	72.72	149.89	260.75
Fiber	1.29	9.81	4.16	4.9	6.67

Fruit	0.01	0.04	0.02	0.01	0
Livestock	2.32	16.58	6.95	6.52	7.36
Oil crops	0.37	5.24	2.43	2.65	3.31
Pulses	0.04	0.43	0.19	0.3	0.47
Roots & tubers	0.2	1.97	0.87	0.98	1.27
Sugar crops	0.03	0.41	0.19	0.14	0.1
Vegetables	0.09	0.61	0.25	0.18	0.15
**Kenya**					
Cereals	14.95	31.18	3.59	13	0
Fiber	0.31	0.66	0.08	0.31	0
Fruit	0.34	0.74	0.09	0.32	0
Livestock	7.53	16.21	1.91	6.03	0
Oil crops	0.38	0.79	0.09	0.34	0
Pulses	3.1	6.17	0.69	2.05	0
Roots & tubers	1.88	4.08	0.48	1.75	0
Sugar crops	0.11	0.25	0.03	0.11	0
Vegetables	0.22	0.49	0.06	0.22	0
**Kuwait**					
Cereals	0	0.05	0.02	0.01	0
Fiber	0	0	0	0	0
Fruit	0	0.04	0.02	0.01	0
Livestock	0	1.29	0.65	0.37	0.06
Oil crops	0	0	0	0	0
Pulses	0	0	0	0	0
Roots & tubers	0	0.03	0.01	0.01	0
Sugar crops	0	0	0	0	0
Vegetables	0	0.06	0.03	0.02	0
**Kyrgyzstan**					
Cereals	3.35	17.06	6.13	8.31	12.32
Fiber	0.42	2.52	0.96	1.15	1.57
Fruit	0	0.02	0.01	0.01	0.01
Livestock	1.33	4.96	1.47	1.1	0.95
Oil crops	0.15	0.77	0.27	0.27	0.32
Pulses	0.05	0.85	0.4	0.22	0.04
Roots & tubers	0.19	0.98	0.35	0.48	0.72
Sugar crops	0.04	0.21	0.08	0.11	0.17
Vegetables	0.03	0.16	0.06	0.08	0.11
**Lao People's Democratic Republic**					
Cereals	12.76	21.7	1.06	0.39	0
Fiber	0.17	0.24	0.01	0	0
Fruit	0.07	0.13	0.01	0	0
Livestock	0.82	1.43	0.07	0.03	0
Oil crops	0.44	0.64	0.02	0.01	0
Pulses	0.08	0.14	0.01	0	0
Roots & tubers	0.3	0.45	0.02	0.01	0

Sugar crops	0.01	0.02	0	0	0
Vegetables	0	0	0	0	0
**Latvia**					
Cereals	0.85	7.04	5.42	23.17	9.79
Fiber	0	0	0	0	0
Fruit	0	0	0	0.01	0
Livestock	0.12	0.91	0.68	2.4	0.88
Oil crops	0.15	1.21	0.92	3.75	1.53
Pulses	0	0.01	0.01	0.04	0.01
Roots & tubers	0.02	0.2	0.15	0.68	0.29
Sugar crops	0.01	0.12	0.1	0.46	0.2
Vegetables	0	0.01	0	0.02	0.01
**Lebanon**					
Cereals	0.61	2.8	0.99	1	1.26
Fiber	0	0	0	0	0
Fruit	0.03	0.14	0.04	0.05	0.07
Livestock	0.29	1.42	0.52	0.85	1.4
Oil crops	0.02	0.11	0.04	0.02	0.01
Pulses	0.01	0.04	0.01	0.02	0.02
Roots & tubers	0.03	0.29	0.13	0.22	0.36
Sugar crops	0.01	0.05	0.02	0.02	0.03
Vegetables	0.04	0.21	0.08	0.08	0.11
**Lesotho**					
Cereals	0.46	1.08	0.13	0	0
Fiber	0	0	0	0	0
Fruit	0	0	0	0	0
Livestock	0.25	0.53	0.06	0	0
Oil crops	0	0	0	0	0
Pulses	0.04	0.08	0.01	0	0
Roots & tubers	0.05	0.14	0.02	0	0
Sugar crops	0	0	0	0	0
Vegetables	0	0	0	0	0
**Liberia**					
Cereals	0.57	1.28	0.15	0	0
Fiber	0	0	0	0	0
Fruit	0.04	0.11	0.01	0	0
Livestock	0.06	0.2	0.03	0	0
Oil crops	0.05	0.14	0.02	0	0
Pulses	0.01	0.02	0	0	0
Roots & tubers	0.43	1.02	0.13	0	0
Sugar crops	0.01	0.02	0	0	0
Vegetables	0	0	0	0	0
**Libya**					
Cereals	0.64	2.8	0.91	1.27	1.92
Fiber	0	0	0	0	0
Fruit	0.05	0.24	0.08	0.11	0.17

Livestock	0.23	1.78	0.75	0.63	0.62
Oil crops	0.03	0.12	0.04	0.05	0.08
Pulses	0.02	0.08	0.03	0.04	0.06
Roots & tubers	0.05	0.22	0.07	0.1	0.16
Sugar crops	0	0	0	0	0
Vegetables	0.05	0.21	0.07	0.1	0.15
**Lithuania**					
Cereals	3.47	18.01	10.95	36.59	12.92
Fiber	0	0	0	0	0
Fruit	0	0.01	0	0.01	0
Livestock	0.46	2.37	1.43	5.42	2.15
Oil crops	0.42	2.33	1.48	5.06	1.84
Pulses	0.08	0.3	0.13	0.36	0.12
Roots & tubers	0.08	0.35	0.19	0.59	0.2
Sugar crops	0.03	0.34	0.29	1.05	0.36
Vegetables	0	0.01	0	0.01	0
**Luxembourg**					
Cereals	0	0.78	0.99	2.65	0.42
Fiber	0	0	0	0	0
Fruit	0	0	0	0.01	0
Livestock	0	0.24	0.31	0.83	0.13
Oil crops	0	0.13	0.16	0.44	0.07
Pulses	0	0.01	0.01	0.02	0
Roots & tubers	0	0.01	0.01	0.02	0
Sugar crops	0	0	0	0	0
Vegetables	0	0	0	0	0
**Madagascar**					
Cereals	19.39	23.47	1.37	0	0
Fiber	0.34	0.42	0.02	0	0
Fruit	0.17	0.21	0.01	0	0
Livestock	3.55	4.32	0.25	0	0
Oil crops	0.21	0.26	0.02	0	0
Pulses	1.24	1.5	0.08	0	0
Roots & tubers	4.78	5.78	0.33	0	0
Sugar crops	0.08	0.16	0.02	0	0
Vegetables	0.03	0.03	0	0	0
**Malawi**					
Cereals	12.78	18.9	1.66	1.84	0
Fiber	1.26	1.93	0.17	0.09	0
Fruit	0.23	0.37	0.04	0.02	0
Livestock	0.83	1.03	0.06	0.12	0
Oil crops	1.24	1.51	0.08	0	0
Pulses	1.43	2	0.16	0.08	0
Roots & tubers	4.86	6.87	0.56	0.4	0
Sugar crops	0.09	0.14	0.01	0.01	0
Vegetables	0.05	0.08	0.01	0	0

**Malaysia**					
Cereals	1.61	11.26	1.28	11.7	0
Fiber	0	0	0	0	0
Fruit	0.13	0.29	0.02	0.13	0
Livestock	2.69	9.15	0.79	4.45	0
Oil crops	0.71	1.56	0.11	0.7	0
Pulses	0	0	0	0	0
Roots & tubers	0.05	0.13	0.01	0.04	0
Sugar crops	0.01	0.02	0	0.01	0
Vegetables	0.02	0.03	0	0.02	0
**Mali**					
Cereals	17.62	32.17	3.37	0.01	0
Fiber	7.99	14.73	1.55	0.01	0
Fruit	0.04	0.07	0.01	0	0
Livestock	3.52	7.11	0.81	0	0
Oil crops	0.16	0.26	0.03	0	0
Pulses	0	0	0	0	0
Roots & tubers	0.3	0.65	0.08	0	0
Sugar crops	0.01	0.02	0	0	0
Vegetables	0.06	0.14	0.02	0	0
**Mauritania**					
Cereals	0.89	1.46	0.13	0	0
Fiber	0	0	0	0	0
Fruit	0.03	0.05	0.01	0	0
Livestock	1.43	2.19	0.18	0	0
Oil crops	0	0	0	0	0
Pulses	0.19	0.29	0.02	0	0
Roots & tubers	0.01	0.01	0	0	0
Sugar crops	0	0	0	0	0
Vegetables	0	0	0	0	0
**Mauritius**					
Cereals	0	0	0	0	0
Fiber	0	0	0	0	0
Fruit	0	0	0	0	0
Livestock	0	0	0	0	0
Oil crops	0	0	0	0	0
Pulses	0	0	0	0	0
Roots & tubers	0	0	0	0	0
Sugar crops	0	0	0	0	0
Vegetables	0	0	0	0	0
**Mexico**					
Cereals	66.98	147.09	129.65	90.57	131.34
Fiber	0.01	0.43	1.61	5.22	17.29
Fruit	0.55	1.28	1.3	0.83	1.14
Livestock	15.74	37.98	41.47	26.02	33.16
Oil crops	2.72	5.39	3.07	1.64	1.73

Pulses	5.85	10.95	4.6	2.94	4.09
Roots & tubers	0.35	0.86	0.97	0.66	0.9
Sugar crops	0.58	1.4	1.5	0.82	0.85
Vegetables	0.5	1.22	1.41	1.21	2.19
**Mongolia**					
Cereals	0.7	0.98	0.39	2.43	0
Fiber	0	0	0	0	0
Fruit	0	0	0	0	0
Livestock	1.88	1.87	0.63	2.15	0
Oil crops	0.01	0.24	0.13	0.07	0
Pulses	0	0	0	0.01	0
Roots & tubers	0.03	0.05	0.02	0.12	0
Sugar crops	0	0	0	0	0
Vegetables	0	0	0	0	0
**Montenegro**					
Cereals	0.04	0.14	0.04	0.05	0
Fiber	0	0	0	0	0
Fruit	0	0.02	0	0.01	0
Livestock	2.61	9.28	3.07	3.65	0.17
Oil crops	0	0	0	0	0
Pulses	0.01	0.04	0.01	0.01	0
Roots & tubers	0.04	0.13	0.04	0.05	0
Sugar crops	0	0	0	0	0
Vegetables	0.01	0.02	0.01	0.01	0
**Morocco**					
Cereals	24.41	87.92	25.76	25.88	32.09
Fiber	0	0.01	0	0	0
Fruit	0.16	0.54	0.15	0.15	0.17
Livestock	3.65	11.56	3.01	2.09	1.66
Oil crops	0.31	1	0.27	0.25	0.29
Pulses	0.15	0.54	0.16	0.13	0.13
Roots & tubers	0.47	1.58	0.43	0.38	0.41
Sugar crops	1	3.46	1	0.96	1.16
Vegetables	0.45	1.51	0.42	0.34	0.35
**Mozambique**					
Cereals	5.13	13.97	1.89	1.81	0
Fiber	1.09	1.7	0.15	6.1	0
Fruit	0.04	0.11	0.02	0.03	0
Livestock	1.06	2.32	0.28	0	0
Oil crops	0.96	2.03	0.24	0.7	0
Pulses	1.63	2.84	0.29	0.27	0
Roots & tubers	4.21	10.39	1.3	0.27	0
Sugar crops	0.04	0.15	0.02	0	0
Vegetables	0.07	0.13	0.01	0.04	0
**Myanmar**					
Cereals	119.33	218.8	12.09	4.66	0

Fiber	3.81	6.42	0.31	0.74	0
Fruit	0.22	0.37	0.02	0.01	0
Livestock	8.41	13.49	0.6	0.92	0
Oil crops	27.01	45.54	2.12	0.76	0
Pulses	24.36	40.86	1.93	0.69	0
Roots & tubers	0.65	1.1	0.05	0.02	0
Sugar crops	0.31	0.49	0.02	0.01	0
Vegetables	0.5	0.83	0.04	0.01	0
**Namibia**					
Cereals	0.49	1.61	0.24	0.21	0
Fiber	0	0	0	0	0
Fruit	0	0.01	0	0	0
Livestock	0.69	1.17	0.12	0.35	0
Oil crops	0	0	0	0	0
Pulses	0.02	0.08	0.01	0.05	0
Roots & tubers	0	0	0	0	0
Sugar crops	0	0	0	0	0
Vegetables	0	0.01	0	0	0
**Nepal**					
Cereals	42.97	80.88	9.21	1.79	0
Fiber	0	0	0	0	0
Fruit	0.03	0.05	0	0	0
Livestock	4.54	8.18	0.91	0.18	0
Oil crops	0.32	0.6	0.07	0.01	0
Pulses	0.32	0.55	0.06	0.01	0
Roots & tubers	1.34	2.44	0.27	0.05	0
Sugar crops	0.08	0.15	0.02	0	0
Vegetables	0	0	0	0	0
**Netherlands**					
Cereals	1.54	9.08	5.94	28.61	13.13
Fiber	0	0	0	0	0
Fruit	0	0.02	0.01	0.06	0.03
Livestock	3.23	21.89	15.36	38.34	9.11
Oil crops	0	0.04	0.04	0.29	0.14
Pulses	0.01	0.05	0.03	0.14	0.06
Roots & tubers	0.23	1.73	1.34	7.53	3.59
Sugar crops	0.41	2.35	1.52	7.44	3.43
Vegetables	0.08	0.45	0.29	1.26	0.55
**New Zealand**					
Cereals	0.02	0.52	0.8	4.01	20.42
Fiber	0	0	0	0	0
Fruit	0	0.01	0.01	0.04	0.22
Livestock	0.11	3.11	4.48	19.47	73.73
Oil crops	0	0	0	0.02	0.11
Pulses	0	0.02	0.03	0.17	0.86
Roots & tubers	0	0.02	0.03	0.17	0.88

Sugar crops	0	0	0	0	0
Vegetables	0	0	0	0.02	0.12
**Nicaragua**					
Cereals	1.76	3.73	2.92	1.74	1.87
Fiber	0.03	0.06	0.05	0.03	0.03
Fruit	0.02	0.05	0.04	0.02	0.03
Livestock	0.7	1.59	1.55	1.02	1.24
Oil crops	0.09	0.2	0.16	0.09	0.1
Pulses	1.12	2.09	0.87	0.46	0.42
Roots & tubers	0.06	0.12	0.1	0.06	0.06
Sugar crops	0.06	0.13	0.1	0.06	0.07
Vegetables	0	0.01	0.01	0	0
**Niger**					
Cereals	23.03	31.93	2.42	7.08	0
Fiber	0.27	0.36	0.03	0	0
Fruit	0.01	0.02	0	0.01	0
Livestock	4.77	6.38	0.45	0.42	0
Oil crops	1.27	1.75	0.13	0.28	0
Pulses	0.17	0.23	0.02	0.03	0
Roots & tubers	0.23	0.32	0.02	0.06	0
Sugar crops	0.01	0.01	0	0	0
Vegetables	0.26	0.35	0.03	0.06	0
**Nigeria**					
Cereals	141.52	245.5	24.22	7.93	0
Fiber	9.93	18.76	2	0.01	0
Fruit	0.87	1.75	0.2	0.27	0
Livestock	11.17	19.38	1.95	4.54	0
Oil crops	11.86	21.94	2.29	1.89	0
Pulses	0.24	0.42	0.04	0	0
Roots & tubers	56.61	115.4	13.01	17.68	0
Sugar crops	0.05	0.06	0	0	0
Vegetables	1.83	2.63	0.21	0.17	0
**Norway**					
Cereals	1.74	12.83	9.11	17.01	1.71
Fiber	0	0	0	0	0
Fruit	0	0	0	0	0
Livestock	0.25	2.88	2.65	6.03	0.82
Oil crops	0.02	0.15	0.11	0.2	0.02
Pulses	0	0	0	0	0
Roots & tubers	0.03	0.22	0.16	0.3	0.03
Sugar crops	0	0	0	0	0
Vegetables	0	0.01	0	0.01	0
**Oman**					
Cereals	0.03	0.14	0.05	0.02	0
Fiber	0	0	0	0	0
Fruit	0.13	0.59	0.22	0.11	0.02

Livestock	0.1	0.46	0.17	0.08	0.01
Oil crops	0	0	0	0	0
Pulses	0	0	0	0	0
Roots & tubers	0	0.01	0	0	0
Sugar crops	0	0	0	0	0
Vegetables	0.01	0.04	0.01	0.01	0
**Pakistan**					
Cereals	286.35	674.99	85.5	16.48	0
Fiber	92.75	241.1	31.5	6.11	0
Fruit	0.83	1.96	0.25	0.05	0
Livestock	61.37	106.32	11.66	2.23	0
Oil crops	8.15	18.74	2.36	0.45	0
Pulses	2.03	4.64	0.58	0.11	0
Roots & tubers	1.3	2.79	0.34	0.07	0
Sugar crops	1.37	3.29	0.42	0.08	0
Vegetables	0.93	2.31	0.3	0.06	0
**Panama**					
Cereals	0.55	1.27	1.23	0.55	0.32
Fiber	0	0	0	0	0
Fruit	0.07	0.18	0.22	0.1	0.07
Livestock	0.6	1.39	1.32	0.59	0.34
Oil crops	0.01	0.03	0.04	0.02	0.01
Pulses	0.02	0.04	0.05	0.03	0.02
Roots & tubers	0.02	0.05	0.06	0.03	0.02
Sugar crops	0.01	0.04	0.07	0.03	0.01
Vegetables	0.01	0.02	0.02	0.01	0.01
**Papua New Guinea**					
Cereals	0.07	0.08	0.03	0.01	0
Fiber	0	0	0	0	0
Fruit	0.27	0.39	0.15	0.08	0
Livestock	0.61	0.49	0.13	0.06	0
Oil crops	1.56	2.28	0.88	0.48	0
Pulses	0.01	0.01	0	0	0
Roots & tubers	0.67	0.95	0.36	0.2	0
Sugar crops	0.01	0.01	0.01	0	0
Vegetables	0	0	0	0	0
**Paraguay**					
Cereals	0.14	2.5	2.78	7.85	41.28
Fiber	0.07	1.08	1.08	1.96	5.47
Fruit	0	0.01	0.02	0.04	0.18
Livestock	0.1	1.54	1.53	2.8	8.26
Oil crops	0.16	3.76	6.01	29.87	194.96
Pulses	0.02	0.29	0.28	0.43	0.97
Roots & tubers	0.03	0.52	0.64	2.08	10.73
Sugar crops	0	0.03	0.04	0.09	0.21
Vegetables	0	0.01	0.02	0.04	0.1

**Peru**					
Cereals	0.8	12.36	11.37	14.48	18.37
Fiber	0.18	2.74	2.53	3.23	4.04
Fruit	0.03	0.44	0.41	0.54	0.7
Livestock	0.34	5.2	4.7	5.51	5.81
Oil crops	0.01	0.08	0.07	0.09	0.12
Pulses	0.06	0.91	0.83	0.96	0.85
Roots & tubers	0.13	2.08	1.95	2.68	3.9
Sugar crops	0.01	0.15	0.14	0.19	0.25
Vegetables	0.02	0.28	0.25	0.29	0.29
**Philippines**					
Cereals	44.8	115.64	9.2	82.53	0
Fiber	0.04	0.09	0.01	0.04	0
Fruit	1.53	3.94	0.31	1.98	0
Livestock	11.8	27.49	1.89	3.1	0
Oil crops	16.76	43.26	3.39	21.74	0
Pulses	0.2	0.5	0.04	0.25	0
Roots & tubers	1.46	4.01	0.32	0.75	0
Sugar crops	0.77	2.02	0.15	0.22	0
Vegetables	0.09	0.22	0.02	0.11	0
**Poland**					
Cereals	37.57	151.05	69.57	238.27	92.36
Fiber	0	0	0	0	0
Fruit	0.06	0.21	0.08	0.23	0.08
Livestock	8.66	32.48	13.34	36.48	12.03
Oil crops	1.72	11.42	8.55	43.87	19.89
Pulses	0.29	1.07	0.43	1.15	0.38
Roots & tubers	2.67	9.08	2.98	6.15	1.69
Sugar crops	1.44	6.74	3.79	14.59	5.83
Vegetables	0.18	0.67	0.26	0.71	0.24
**Portugal**					
Cereals	1.17	4.68	2.13	8.67	3.79
Fiber	0	0	0	0	0
Fruit	0.11	0.35	0.11	0.23	0.07
Livestock	2.3	7.93	2.71	6.04	1.78
Oil crops	0.03	0.11	0.06	0.32	0.16
Pulses	0.01	0.02	0.01	0.04	0.02
Roots & tubers	0.18	0.64	0.21	0.3	0.04
Sugar crops	0.02	0.14	0.12	0.55	0.22
Vegetables	0.12	0.45	0.19	0.77	0.35
**Qatar**					
Cereals	0	0	0	0	0
Fiber	0	0	0	0	0
Fruit	0	0	0	0	0
Livestock	0.05	0.23	0.09	0.04	0.01
Oil crops	0	0	0	0	0

Pulses	0	0	0	0	0
Roots & tubers	0	0	0	0	0
Sugar crops	0	0	0	0	0
Vegetables	0	0	0	0	0
**Republic of Korea**					
Cereals	31.2	42.92	1.32	0.37	0
Fiber	0	0	0	0	0
Fruit	0.16	0.21	0.01	0	0
Livestock	17.01	23.18	0.7	0.19	0
Oil crops	3.74	4.99	0.14	0.04	0
Pulses	0.11	0.15	0	0	0
Roots & tubers	0.91	1.23	0.04	0.01	0
Sugar crops	0	0	0	0	0
Vegetables	0.94	1.3	0.04	0.01	0
**Romania**					
Cereals	16.94	76.84	42.78	164.18	64.57
Fiber	0	0	0	0	0
Fruit	0.02	0.12	0.07	0.35	0.15
Livestock	2.93	11.88	5.52	17.64	6.45
Oil crops	1.86	9.62	6.11	26.29	10.94
Pulses	0.14	0.58	0.26	0.71	0.23
Roots & tubers	0.68	2.75	1.24	2.89	0.76
Sugar crops	0.19	0.71	0.29	0.86	0.32
Vegetables	0.09	0.38	0.19	0.7	0.27
**Russian Federation**					
Cereals	121.65	518.07	262.64	1278.94	598.01
Fiber	0	0	0	0	0
Fruit	0.02	0.11	0.07	0.46	0.23
Livestock	10.68	50.15	28.25	110.03	44.36
Oil crops	7.05	33.37	19.28	112.75	55.91
Pulses	1.9	8.19	4.11	16.53	7.08
Roots & tubers	5.69	22.35	9.78	31.72	12.12
Sugar crops	3.47	13.11	5.68	33.33	17.12
Vegetables	0.31	1.27	0.61	2.54	1.11
**Rwanda**					
Cereals	2.26	3.66	0.34	0	0
Fiber	0	0	0	0	0
Fruit	0.86	1.42	0.14	0	0
Livestock	0.78	1.08	0.08	0	0
Oil crops	0.63	0.8	0.05	0	0
Pulses	2.89	4.53	0.41	0	0
Roots & tubers	2.59	3.73	0.3	0	0
Sugar crops	0	0	0	0	0
Vegetables	0.03	0.03	0	0	0
**Saint Lucia**					
Cereals	0	0	0	0	0

Fiber	0	0	0	0	0
Fruit	0	0	0	0	0
Livestock	0	0	0	0	0
Oil crops	0	0	0	0	0
Pulses	0	0	0	0	0
Roots & tubers	0	0	0	0	0
Sugar crops	0	0	0	0	0
Vegetables	0	0	0	0	0
**Saint Vincent and the Grenadines**					
Cereals	0	0	0	0	0
Fiber	0	0	0	0	0
Fruit	0	0	0	0	0
Livestock	0	0	0	0	0
Oil crops	0	0	0	0	0
Pulses	0	0	0	0	0
Roots & tubers	0	0	0	0	0
Sugar crops	0	0	0	0	0
Vegetables	0	0	0	0	0
**Saudi Arabia**					
Cereals	16.97	65.24	21.05	10.23	1.78
Fiber	0	0	0	0	0
Fruit	0.65	2.31	0.69	0.34	0.06
Livestock	2.15	8.64	2.78	1.4	0.24
Oil crops	0.12	0.24	0.03	0.01	0
Pulses	0.01	0.05	0.02	0.01	0
Roots & tubers	0.15	0.56	0.18	0.09	0.02
Sugar crops	0	0	0	0	0
Vegetables	0.15	0.49	0.13	0.06	0.01
**Senegal**					
Cereals	4.71	10	1.19	0.01	0
Fiber	0.6	1.96	0.29	0	0
Fruit	0.02	0.03	0	0	0
Livestock	1.15	2.28	0.26	0	0
Oil crops	0.7	0.86	0.06	0	0
Pulses	0	0	0	0	0
Roots & tubers	0.33	0.41	0.03	0	0
Sugar crops	0.02	0.06	0.01	0	0
Vegetables	0.1	0.2	0.02	0	0
**Serbia**					
Cereals	14.3	52.44	20.53	53.73	17.41
Fiber	0	0	0	0	0
Fruit	0.04	0.13	0.04	0.09	0.03
Oil crops	2.22	8.53	3.64	10.22	3.41
Pulses	0.25	0.84	0.28	0.68	0.22
Roots & tubers	0.15	0.56	0.23	0.66	0.23

Sugar crops	1.1	3.48	0.98	2.27	0.78
Vegetables	0.05	0.17	0.06	0.14	0.04
**Sierra Leone**					
Cereals	2.87	6.41	0.78	0	0
Fiber	0	0	0	0	0
Fruit	0.01	0.02	0	0	0
Livestock	0.18	0.27	0.02	0	0
Oil crops	0.06	0.14	0.02	0	0
Pulses	0.16	0.49	0.07	0	0
Roots & tubers	1.21	2.91	0.37	0	0
Sugar crops	0	0	0	0	0
Vegetables	0.01	0.02	0	0	0
**Slovakia**					
Cereals	7.06	27.01	11.7	35.47	12.45
Fiber	0	0	0	0	0
Fruit	0.01	0.02	0.01	0.01	0
Livestock	0.36	1.89	1.15	3.76	1.31
Oil crops	1.52	5.64	2.34	7.11	2.52
Pulses	0.07	0.25	0.11	0.33	0.12
Roots & tubers	0.04	0.17	0.09	0.25	0.07
Sugar crops	0.22	0.94	0.48	1.44	0.47
Vegetables	0.01	0.04	0.02	0.05	0.02
**Slovenia**					
Cereals	1.88	6.28	1.85	2.05	0.1
Fiber	0	0	0	0	0
Fruit	0.02	0.06	0.02	0.02	0
Livestock	0.9	2.88	0.77	0.79	0.04
Oil crops	0.07	0.23	0.07	0.07	0
Pulses	0.04	0.13	0.04	0.05	0
Roots & tubers	0.04	0.14	0.04	0.05	0
Sugar crops	0.07	0.24	0.07	0.08	0
Vegetables	0	0.01	0	0	0
**Somalia**					
Cereals	1.59	2.37	0.19	0	0
Fiber	0.15	0.23	0.02	0	0
Fruit	0.03	0.05	0	0	0
Livestock	3.55	6.71	0.72	0	0
Oil crops	1.5	2.29	0.19	0	0
Pulses	0.18	0.3	0.03	0	0
Roots & tubers	0.09	0.14	0.01	0	0
Sugar crops	0.01	0.01	0	0	0
Vegetables	0	0.01	0	0	0
**South Africa**					
Cereals	12.22	64.44	10.63	126.86	0
Fiber	0.22	1.2	0.2	0.67	0
Fruit	0.55	1.37	0.18	0.57	0

Livestock	8.95	23.42	3.1	20.45	0
Oil crops	3.77	12.62	1.84	9.42	0
Pulses	0.08	0.63	0.11	0.78	0
Roots & tubers	0.47	2.01	0.32	1.38	0
Sugar crops	0.11	1.13	0.2	0.6	0
Vegetables	0.17	0.67	0.1	0.4	0
**Spain**					
Cereals	28.29	129.82	70.13	236.43	86.87
Fiber	0.21	1.25	0.9	6.54	3.42
Fruit	0.53	1.96	0.78	2.2	0.78
Livestock	13.65	49.1	18.1	36.81	8.99
Oil crops	0.91	4.26	2.43	12.59	5.94
Pulses	0.38	1.53	0.71	2.57	1.04
Roots & tubers	0.36	1.59	0.78	2.12	0.68
Sugar crops	0.4	2.55	1.82	8.03	3.39
Vegetables	0.5	2.02	0.93	3.39	1.38
**Sri Lanka**					
Cereals	9.66	18.28	2.1	6.07	0
Fiber	0	0	0	0	0
Fruit	0.11	0.25	0.03	0.12	0
Livestock	1.03	1.54	0.15	0.22	0
Oil crops	3.33	6.19	0.71	1.9	0
Pulses	0.05	0.11	0.01	0.06	0
Roots & tubers	0.21	0.45	0.05	0.19	0
Sugar crops	0.02	0.04	0.01	0.03	0
Vegetables	0.06	0.14	0.02	0.07	0
**Sudan (former)**					
Cereals	8.48	46.42	17.06	20.19	27.33
Fiber	1.76	6.66	2	2.41	3.37
Fruit	0.23	0.84	0.24	0.3	0.42
Livestock	7	26.84	8.18	7.22	7.75
Oil crops	3.77	11.38	2.75	3	4
Pulses	0.14	0.5	0.15	0.17	0.22
Roots & tubers	0.16	0.57	0.16	0.19	0.26
Sugar crops	0.09	0.31	0.09	0.1	0.13
Vegetables	0.32	1.14	0.32	0.36	0.49
**Suriname**					
Cereals	0	0.01	0.03	0.24	1.62
Fiber	0	0	0	0	0
Fruit	0	0	0	0.01	0.07
Livestock	0	0	0	0.02	0.16
Oil crops	0	0	0	0	0.03
Pulses	0	0	0	0	0
Roots & tubers	0	0	0	0	0.01
Sugar crops	0	0	0	0	0.01
Vegetables	0	0	0	0	0

**Swaziland**					
Cereals	0.24	0.49	0.06	0.02	0
Fiber	0.03	0.09	0.01	0.01	0
Fruit	0.01	0.03	0	0	0
Livestock	0.19	0.41	0.05	0.12	0
Oil crops	0	0	0	0	0
Pulses	0.01	0.02	0	0	0
Roots & tubers	0.01	0.01	0	0	0
Sugar crops	0.14	0.31	0.04	0.01	0
Vegetables	0	0	0	0	0
**Sweden**					
Cereals	4.22	34.16	25.96	74.81	21.96
Fiber	0	0	0	0	0
Fruit	0	0	0	0	0
Livestock	0.58	4.64	3.51	10.53	3.26
Oil crops	0.31	2.38	1.72	4.7	1.33
Pulses	0.04	0.38	0.31	0.88	0.24
Roots & tubers	0.07	0.49	0.34	0.76	0.16
Sugar crops	0.25	1.75	1.18	2.42	0.43
Vegetables	0	0.01	0.01	0.03	0.01
**Switzerland**					
Cereals	1.71	9.27	6.07	13.03	1.78
Fiber	0	0	0	0	0
Fruit	0.01	0.05	0.03	0.07	0.01
Livestock	0.87	6.72	5.09	10.31	1.21
Oil crops	0.31	1.23	0.59	1.15	0.16
Pulses	0.03	0.18	0.12	0.27	0.04
Roots & tubers	0.02	0.24	0.21	0.46	0.06
Sugar crops	0.08	0.89	0.8	1.79	0.24
Vegetables	0	0.01	0.01	0.02	0
**Syrian Arab Republic**					
Cereals	41.4	118.17	28.06	17.41	12.15
Fiber	7.18	24.09	6.88	5.23	5
Fruit	0.09	0.3	0.09	0.07	0.07
Livestock	3	11.91	3.83	2.35	1.34
Oil crops	0.36	1.17	0.32	0.24	0.22
Pulses	0.05	0.18	0.05	0.04	0.04
Roots & tubers	0.21	0.66	0.18	0.13	0.12
Sugar crops	0.64	1.93	0.49	0.28	0.16
Vegetables	0.26	0.91	0.27	0.2	0.19
**Tajikistan**					
Cereals	3.23	14.37	4.81	4.19	4.36
Fiber	2.74	12.06	4.01	3.55	3.76
Fruit	0.01	0.05	0.02	0.02	0.02
Livestock	0.35	1.68	0.58	0.64	0.83
Oil crops	0.01	0.06	0.02	0.02	0.01

Pulses	0.05	0.22	0.07	0.06	0.06
Roots & tubers	0.13	0.6	0.2	0.18	0.19
Sugar crops	0	0	0	0	0
Vegetables	0.07	0.3	0.1	0.09	0.09
**Thailand**					
Cereals	94.6	269.32	22.38	9.69	0
Fiber	0.15	0.46	0.04	0.02	0
Fruit	0.95	2.4	0.19	0.08	0
Livestock	7.47	29.74	2.91	1.3	0
Oil crops	5.95	16.93	1.43	0.62	0
Pulses	1.17	2.38	0.15	0.06	0
Roots & tubers	12.12	45.1	4.34	1.94	0
Sugar crops	1.52	3.51	0.25	0.11	0
Vegetables	0.08	0.2	0.02	0.01	0
**The former Yugoslav Republic of Macedonia**					
Cereals	1.06	5.57	3.41	6.79	0.83
Fiber	0	0	0	0	0
Fruit	0.01	0.07	0.04	0.08	0.01
Livestock	0.25	0.94	0.36	0.56	0.06
Oil crops	0.01	0.07	0.04	0.07	0.01
Pulses	0.01	0.08	0.04	0.09	0.01
Roots & tubers	0.02	0.13	0.08	0.15	0.02
Sugar crops	0	0.03	0.02	0.03	0
Vegetables	0.02	0.08	0.05	0.09	0.01
**Togo**					
Cereals	4.16	7.66	0.8	0	0
Fiber	1.35	1.64	0.09	0	0
Fruit	0.01	0.02	0	0	0
Livestock	0.39	0.59	0.05	0	0
Oil crops	0.07	0.12	0.01	0	0
Pulses	0.53	1.01	0.11	0	0
Roots & tubers	1.12	2.04	0.21	0	0
Sugar crops	0	0	0	0	0
Vegetables	0	0.01	0	0	0
**Trinidad and Tobago**					
Cereals	0	0	0.02	0.02	0.03
Fiber	0	0	0	0	0
Fruit	0	0	0	0	0.01
Livestock	0	0	0	0	0
Oil crops	0	0.01	0.03	0.03	0.04
Pulses	0	0	0	0	0
Roots & tubers	0	0	0	0	0
Sugar crops	0	0	0.02	0.02	0.03
Vegetables	0	0	0	0	0
**Tunisia**					

Cereals	8	32.39	10.28	10.32	12.57
Fiber	0.01	0.06	0.02	0.02	0.03
Fruit	0.13	0.36	0.08	0.06	0.06
Livestock	0.98	4.86	1.73	1.23	0.93
Oil crops	0.2	0.98	0.34	0.35	0.42
Pulses	0.05	0.22	0.07	0.08	0.1
Roots & tubers	0.09	0.3	0.08	0.11	0.17
Sugar crops	0	0	0	0	0
Vegetables	0.1	0.47	0.16	0.33	0.59
**Turkey**					
Cereals	171.18	550.81	145.64	127.17	140.01
Fiber	19.01	70.66	21.81	16.01	14.01
Fruit	0.75	2.23	0.54	0.46	0.49
Livestock	14.14	45	11.68	7.85	5.76
Oil crops	4.69	18.35	5.76	3.21	1.21
Pulses	0.89	2.84	0.75	0.48	0.32
Roots & tubers	2.1	5.13	0.92	0.6	0.49
Sugar crops	7.87	21.05	4.34	2.7	1.81
Vegetables	3.1	9.33	2.27	1.81	1.79
**Turkmenistan**					
Cereals	2.58	43.04	20.22	25.11	34.42
Fiber	1.09	18.08	8.49	10.64	14.69
Fruit	0	0.08	0.04	0.05	0.06
Livestock	0.4	5.01	2.29	2.95	4.18
Oil crops	0	0	0	0	0
Pulses	0.01	0.14	0.06	0.08	0.11
Roots & tubers	0.01	0.17	0.08	0.09	0.11
Sugar crops	0.01	0.22	0.1	0.13	0.17
Vegetables	0.01	0.21	0.1	0.12	0.17
**Uganda**					
Cereals	13.45	23.37	2.35	0.01	0
Fiber	1.14	2.26	0.25	0	0
Fruit	2.87	5.38	0.58	0	0
Livestock	3.53	8.16	1.01	0.01	0
Oil crops	10.14	14.44	1.16	0	0
Pulses	4.12	7.45	0.78	0	0
Roots & tubers	6.94	13.37	1.44	0.01	0
Sugar crops	0.08	0.15	0.02	0	0
Vegetables	0.11	0.2	0.02	0	0
**Ukraine**					
Cereals	77.13	297.84	128.6	392.64	141.31
Fiber	0	0	0	0	0
Fruit	0.03	0.13	0.06	0.19	0.07
Livestock	7.58	28.38	11.56	31.99	10.77
Oil crops	13.88	53.96	23.47	75.74	28.65
Pulses	0.88	4.21	2.41	8.51	3.17

Roots & tubers	2.71	11.83	5.99	16.51	5.04
Sugar crops	2.49	11.31	6.02	21.28	8.19
Vegetables	0.25	0.97	0.42	1.39	0.54
**United Arab Emirates**					
Cereals	0	0	0	0	0
Fiber	0	0	0	0	0
Fruit	0.37	1.7	0.63	0.31	0.05
Livestock	0.05	0.25	0.09	0.04	0.01
Oil crops	0	0	0	0	0
Pulses	0	0	0	0	0
Roots & tubers	0	0.01	0	0	0
Sugar crops	0	0	0	0	0
Vegetables	0.02	0.1	0.04	0.02	0
**United Kingdom**					
Cereals	38.18	188.15	107.98	326.19	106.56
Fiber	0	0	0	0	0
Fruit	0	0.02	0.01	0.04	0.01
Livestock	6.25	35.62	21.46	47.95	10.8
Oil crops	5.01	24.34	13.83	40.34	12.64
Pulses	0.23	1.95	1.53	4.92	1.62
Roots & tubers	0.24	2.22	1.83	6.25	2.14
Sugar crops	0.36	3.33	2.76	10.27	3.78
Vegetables	0.02	0.13	0.1	0.32	0.11
**United Republic of Tanzania**					
Cereals	21.93	55.16	7.13	0.04	0
Fiber	3.31	9.2	1.25	0.01	0
Fruit	0.85	2.33	0.31	0	0
Livestock	5.5	10.6	1.13	0.01	0
Oil crops	4.24	11.43	1.53	0.01	0
Pulses	6.85	13.23	1.43	0.01	0
Roots & tubers	4.68	13.87	1.94	0.01	0
Sugar crops	0.05	0.16	0.02	0	0
Vegetables	0.07	0.2	0.03	0	0
**United States of America**					
Cereals	0.58	295.23	627.61	2938.13	2958.77
Fiber	0.03	24.69	58.47	296.42	314.17
Fruit	0	0.91	1.44	4.31	3.7
Livestock	0.3	87.8	148.46	461.51	340.31
Oil crops	0.25	157.55	363.29	1748.46	1766.88
Pulses	0.01	3.15	5.99	24.1	20.66
Roots & tubers	0.01	3.85	6.66	21.49	12.68
Sugar crops	0	2.22	6.1	35.95	42.44
Vegetables	0	1.44	2.87	11.37	9.3
**Uruguay**					
Cereals	0.07	1.64	2.57	9.97	39.18
Fiber	0	0	0	0	0

Fruit	0	0.01	0.01	0.04	0.14
Livestock	0.03	0.66	1.06	4.45	20.58
Oil crops	0.01	0.37	0.6	3.71	30.42
Pulses	0	0.01	0.01	0.04	0.13
Roots & tubers	0	0.01	0.02	0.08	0.35
Sugar crops	0	0	0	0	0.02
Vegetables	0	0	0	0.02	0.09
**Uzbekistan**					
Cereals	19.07	113.8	44.19	40.84	45.05
Fiber	16.03	95.61	37.13	34.3	37.83
Fruit	0.05	0.3	0.12	0.11	0.12
Livestock	2.56	19.27	7.98	5.88	4.54
Oil crops	0.05	0.28	0.11	0.09	0.1
Pulses	0.01	0.05	0.02	0.02	0.02
Roots & tubers	0.17	0.98	0.38	0.35	0.39
Sugar crops	0	0	0	0	0
Vegetables	0.24	1.41	0.55	0.5	0.56
**Venezuela (Bolivarian Republic of)**					
Cereals	0.24	3.98	4.26	9.64	34.16
Fiber	0.01	0.11	0.12	0.28	0.96
Fruit	0	0.07	0.08	0.23	0.9
Livestock	0.22	3.51	3.41	5.89	17.75
Oil crops	0.02	0.36	0.4	0.93	3.26
Pulses	0.01	0.1	0.1	0.19	0.49
Roots & tubers	0.01	0.17	0.19	0.47	1.72
Sugar crops	0	0.08	0.08	0.19	0.66
Vegetables	0	0.05	0.06	0.14	0.46
**Viet Nam**					
Cereals	70.59	237.57	21.78	116.93	0
Fiber	0.39	0.88	0.06	0.23	0
Fruit	0.49	1.13	0.08	0.32	0
Livestock	17.93	26.71	1.02	9.56	0
Oil crops	5.3	12.71	0.97	2.08	0
Pulses	1.51	3.27	0.23	0.34	0
Roots & tubers	8.57	15.16	0.81	1	0
Sugar crops	0.31	0.91	0.08	0.34	0
Vegetables	0.1	0.23	0.02	0.05	0
**Yemen**					
Cereals	2.22	9.92	3.35	1.72	0.29
Fiber	0.19	0.75	0.23	0.12	0.02
Fruit	0.05	0.24	0.08	0.04	0.01
Livestock	1.73	4.08	0.69	0.3	0.05
Oil crops	0.27	0.96	0.28	0.14	0.02
Pulses	0.04	0.15	0.05	0.02	0
Roots & tubers	0.07	0.29	0.09	0.05	0.01

Sugar crops	0	0	0	0	0
Vegetables	0.06	0.34	0.13	0.07	0.01
**Zambia**					
Cereals	5.16	12.9	1.69	3.22	0
Fiber	0.98	2.66	0.36	4.01	0
Fruit	0	0	0	0	0
Livestock	1.1	1.75	0.16	0.43	0
Oil crops	0.66	2.25	0.33	0.35	0
Pulses	0.07	0.12	0.01	0.03	0
Roots & tubers	1.25	1.77	0.13	0.08	0
Sugar crops	0.08	0.12	0.01	0.03	0
Vegetables	0.03	0.04	0	0.01	0
**Zimbabwe**					
Cereals	4.47	19.18	3.02	0.99	0
Fiber	1.5	8.96	1.5	0.83	0
Fruit	0.02	0.11	0.02	0	0
Livestock	2.33	3.73	0.35	0.03	0
Oil crops	1.22	2.71	0.33	0.15	0
Pulses	0.14	0.52	0.08	0.02	0
Roots & tubers	0.21	0.4	0.04	0.03	0
Sugar crops	0.09	0.2	0.03	0.02	0
Vegetables	0	0.03	0.01	0	0
